# Health-system challenges, resilience strategies, and actionable priorities for non-communicable disease care in humanitarian crises: A scoping review

**DOI:** 10.1371/journal.pgph.0006035

**Published:** 2026-08-18

**Authors:** Pirhossein Kolivand, Sahar Kargar, Fereshte Karimi, Masoud Behzadifar, Samad Azari, Ahad Bakhtiari

**Affiliations:** 1 Department of Health Economics, Faculty of Medicine, Shahed University, Tehran, Iran; 2 Research Center for Emergency and Disaster Resilience, Red Crescent Society of the Islamic Republic of Iran, Tehran, Iran; 3 Health Equity Research Center (HERC), Tehran University of Medical Sciences, Tehran, Iran; 4 Iranian Research Network for Social Determinants of Health (IRNSDH), Tehran, Iran; 5 Social Determinants of Health Research Center, Lorestan University of Medical Sciences, Khorramabad, Iran; 6 Health Management and Economics Research Center, Health Management Research Institute, Iran University of Medical Sciences, Tehran, Iran; University of New South Wales, AUSTRALIA

## Abstract

Humanitarian crises disrupt the continuous care required for non-communicable diseases (NCDs), yet evidence on effective health-system responses remains fragmented. We conducted a scoping review, reported in accordance with PRISMA-ScR, to map health-system challenges, implemented and recommended strategies, resilience functions, and evidence gaps for cardiovascular diseases, diabetes, cancer, and chronic respiratory diseases in humanitarian settings. PubMed/MEDLINE, Scopus, Web of Science, relevant organizational websites, and reference lists were searched. English-language empirical studies, reviews, programme reports, guidelines, policy documents, and operational frameworks addressing continuity of NCD care during crises were eligible. Evidence was synthesized across nine operational domains and characterized according to patient or clinical outcomes, service or implementation outcomes, descriptive or recommendation-only evidence, and economic information. Thirty-seven sources published between 2013 and 2025 were included. Country-specific empirical evidence was concentrated in Asia and the Middle East. Service delivery had the strongest outcome-based evidence, whereas financing, coordination, governance, and transformative system reforms were supported mainly by descriptive or recommendation-based sources. No full comparative economic evaluation was identified. Reported challenges involved disrupted access and referral, failures of medicines and infrastructure, workforce and information constraints, and financial and social barriers. Implemented strategies included integrated primary care, mobile and telemedicine services, structured follow-up, medicine-continuity measures, electronic monitoring, and community and workforce interventions. Empirical evidence predominantly addressed absorptive and adaptive responses; transformative strategies were mainly proposed in guidance and policy documents and were rarely evaluated. Three cross-cutting priorities emerged from the synthesis: a minimum emergency NCD service and medicine package; continuity mechanisms for patients at highest risk from treatment interruption; and simplified, integrated NCD care within emergency and primary-care services. Future research should prospectively evaluate these priorities using standardized implementation, clinical, equity, and economic indicators and should involve affected communities and frontline practitioners in intervention design and assessment.

## Introduction

Non-communicable diseases (NCDs) are the leading causes of morbidity and mortality worldwide, accounting for more than 60% of the global burden of disease and approximately 73% of all deaths [1]. Nearly 80% of NCD-related deaths occur in low- and middle-income countries, where health systems may face substantial structural and resource constraints even under stable conditions [2]. Cardiovascular diseases, diabetes, cancers, and chronic respiratory diseases constitute the four major NCD groups prioritized by the World Health Organization (WHO) because of their major contributions to premature mortality, disability, and long-term health-care needs [3]. In many countries, including those in the Middle East, NCDs account for a substantial proportion of deaths and impose considerable social and economic burdens on individuals, families, and health systems [4].

At the same time, the frequency, scale, duration and complexity of humanitarian emergencies have increased markedly in recent decades [5]. Armed conflicts, forced displacement, natural disasters, and complex emergencies have affected hundreds of millions of people, resulting in large-scale population movements and severe disruption of social structures and health systems [5,6]. Humanitarian crises can damage health infrastructure, interrupt supply chains, reduce the availability of essential medicines and technologies, displace health-care personnel, and weaken referral and information systems [7]. Humanitarian health responses have traditionally prioritized trauma, infectious diseases, and acute malnutrition [8]. However, the increasing burden of NCDs among crisis-affected populations has exposed the limitations of emergency responses designed primarily around acute and short-term health needs [9].

Individuals living with NCDs are particularly vulnerable during humanitarian crises because many require continuous access to medicines, diagnostic monitoring, regular follow-up, and coordinated care across different levels of the health system. Even brief interruptions in routine services may lead to acute complications, disease progression, avoidable hospitalization, disability, or premature death. Evidence from diverse humanitarian settings has documented increased cardiovascular events, deterioration of glycaemic control, exacerbations of chronic respiratory diseases, and interruptions or delays in cancer diagnosis and treatment following emergencies [9]. This vulnerability is likely to become increasingly important as population ageing, demographic transitions, and established NCD risk factors increase the prevalence of chronic conditions in populations subsequently affected by conflict, displacement, disasters, and other emergencies [10].

Global policy frameworks, including the WHO Global Action Plan for the Prevention and Control of NCDs and the Sustainable Development Goals, recognize the need to reduce premature NCD mortality and strengthen health systems [11,12]. Nevertheless, NCD prevention and care remain insufficiently incorporated into many humanitarian preparedness, response, and recovery arrangement [10]. Existing emergency guidelines, health kits, financing mechanisms, and coordination structures provide varying levels of attention to continuity of NCD care, but implementation remains fragmented and uneven across diseases and settings [13]. Difficult decisions concerning the allocation of limited resources, particularly for specialized and resource-intensive services such as cancer care, also remain insufficiently addressed [14]. Strengthening NCD care in humanitarian settings requires more than the inclusion of chronic diseases in emergency plans. It requires health systems that can maintain essential services during an initial shock, modify delivery arrangements as conditions change, and undertake longer-term institutional reforms to address recurrent vulnerabilities. These functions can be understood in terms of absorptive, adaptive, and transformative resilience capacities [9]. Examples may include maintaining emergency stocks of essential medicines, adopting mobile or remote models of care, expanding task-sharing, establishing patient-held records or registries, protecting NCD financing, and integrating chronic care into primary health care and humanitarian coordination mechanisms [9,15]. However, the feasibility and evidence supporting these strategies may vary substantially according to the type and duration of the crisis, baseline health-system capacity, available resources, and the needs of affected populations.

Although previous studies, reviews, and international guidance have described NCD-related needs and possible responses in humanitarian settings, the evidence remains dispersed across different diseases, crisis types, geographic regions, and source types [9,11,12,15]. Existing literature has often emphasized broad recommendations without clearly distinguishing between documented preparedness challenges, interventions that have been implemented, strategies proposed in guidance documents, and actions supported by direct patient or service outcomes. In addition, limited attention has been given to comparing the strength and directness of evidence across preparedness domains, identifying where economic evidence is available, and translating broad calls for “integration” into measurable priorities for countries and humanitarian organizations.

Accordingly, this scoping review aimed to map the health-system challenges affecting the continuity of care for cardiovascular diseases, diabetes, cancer, and chronic respiratory diseases in humanitarian settings; synthesize the interventions and strategies reported or recommended to address these challenges; and identify actionable priorities for strengthening health-system resilience across preparedness, response, and recovery. Specifically, the review sought to organize the available evidence across major operational domains, examine how reported actions contribute to absorptive, adaptive, or transformative resilience, and identify important gaps in implementation, patient-outcome, and economic evidence. The review did not seek to directly measure or rank the overall preparedness of individual countries or humanitarian organizations.

## Methods

### Study design

This study was conducted as a scoping review to identify and map health-system challenges, interventions, strategies, and policy options related to the continuity of care for major non-communicable diseases (NCDs) in humanitarian settings. A scoping review methodology was selected because the available evidence was heterogeneous in terms of source type, study design, disease group, crisis context, and reported actions and outcomes. The review was conducted using a structured scoping review process and is reported in accordance with the Preferred Reporting Items for Systematic Reviews and Meta-Analyses extension for Scoping Reviews (PRISMA-ScR) [16,17].

### Review the objective and research question

The review aimed to map the health-system challenges affecting the continuity of care for cardiovascular diseases, diabetes, cancer, and chronic respiratory diseases during humanitarian crises; identify interventions and strategies that had been implemented or recommended; and highlight gaps and actionable priorities for strengthening health-system resilience across preparedness, response, and recovery. The primary review question was:

- What health-system challenges, interventions, and recommended strategies have been reported for maintaining or strengthening care for major NCDs in humanitarian settings?

### Information sources and search strategy

A systematic literature search was conducted in PubMed/MEDLINE, Scopus, and Web of Science. The search combined terms relating to three main concepts:

1. Major NCDs, including cardiovascular diseases, hypertension, diabetes, cancer, chronic respiratory diseases, asthma, and chronic obstructive pulmonary disease;
2. Humanitarian and crisis settings, including armed conflict, forced displacement, refugee settings, natural disasters, earthquakes, floods, storms, complex emergencies, and public health emergencies;
3. Health-system responses, including preparedness, interventions, care, service delivery, treatment, continuity of care, access, policy, and emergency response.

Controlled vocabulary terms, where available, and free-text terms were combined using Boolean operators. The search syntax was adapted to the requirements of each database. The complete search strategies for PubMed, Scopus, and Web of Science are presented in S1 Table.

The database search was supplemented by manual searches of the websites of relevant international and humanitarian organizations, including the WHO and its regional offices, the Organisation for Economic Co-operation and Development. Reference lists of relevant reviews, guidelines, policy documents, and technical reports were also examined for additional eligible sources.

### Eligibility criteria

#### Inclusion criteria.

Sources were eligible if they:

- Were published in English between January 1, 2000, and September 23, 2025;
- Addressed one or more of the four major NCD groups: cardiovascular diseases, diabetes, cancer, or chronic respiratory diseases;
- Examined populations, services, health systems, or organizations affected by humanitarian or crisis conditions;
- Addressed preparedness, response, recovery, continuity of care, service disruption, health-system interventions, operational actions, or policy options;
- Were original quantitative, qualitative, or mixed-methods studies, implementation or programme evaluations, review articles, guidelines, technical reports, policy documents, or operational frameworks.

Humanitarian contexts included armed conflict, forced displacement, refugee crises, earthquakes, floods, storms, other natural disasters, complex emergencies, and relevant public health emergencies.

#### Exclusion criteria.

Sources were excluded if they:

- Were published in languages other than English;
- Were letters, editorials, commentaries, or opinion pieces without substantive empirical, operational, or policy content;
- Focused exclusively on clinical outcomes without addressing service disruption, continuity of care, health-system preparedness, an intervention, or a policy response;
- Addressed only communicable diseases, trauma, acute malnutrition, or conditions outside the prespecified NCD scope;
- Did not concern a humanitarian, emergency, disaster, displacement, or crisis setting;

Mental health conditions, dialysis-dependent kidney disease, and other chronic conditions were not treated as independent target conditions unless they were discussed in the context of an eligible source addressing at least one of the four prespecified NCD groups.

### Record management and study selection

All retrieved records were imported into EndNote. Duplicate records were identified and removed. Two reviewers (AB, SK) independently screened the titles and abstracts of retrieved records against the eligibility criteria. Sources considered potentially relevant were assessed in full text by the same reviewers. Disagreements were resolved through discussion, and a third reviewer (MB) was consulted when consensus could not be reached. Reasons for full-text exclusion were recorded and summarized in the study-selection flow diagram. The selection process was reported using a PRISMA-ScR-compatible flow diagram.

### Data extraction and synthesis

Data were extracted by one reviewer (AB) using a piloted standardized form and independently verified by a second reviewer (SK). Discrepancies were resolved through discussion and, when necessary, consultation with a third reviewer (MB). Data were extracted using a structured form developed in Microsoft Excel. The form captured general source characteristics, including:

- Author or issuing organization;
- Year of publication;
- Source type or study design;
- Country or geographic setting;
- Target population;
- Type of humanitarian crisis;
- NCD group addressed.

Content-related data included:

- Reported challenges to NCD care;
- Actions, interventions, strategies, or policy recommendations;
- Access to services and continuity of care;
- Risk-factor prevention and community-level interventions;
- Emergency preparedness and operational planning;
- Availability of medicines, supplies, technologies, and equipment;
- Reported implementation, service, or patient outcomes, where explicitly provided.

Following initial screening to remove duplicates and irrelevant records, full texts were assessed for eligibility. Disagreements were resolved through discussion, and when necessary, a third reviewer was consulted to reach a consensus. The included records consisted of different source types, including original research, reviews, guidelines, reports, and policy documents. They are therefore referred to collectively as “included sources” rather than as independent studies. Counts of included sources should not be interpreted as counts of independent interventions or independent effect estimates.

### Narrative and thematic synthesis

The included sources were synthesized narratively because of substantial heterogeneity in study design, crisis setting, target population, interventions, and reported outcomes. No meta-analysis or inferential statistical testing was undertaken.

The original analysis used inductive coding to identify recurring challenges, actions, and recommendations relevant to NCD care in humanitarian settings. During thematic synthesis, similar codes were grouped into broader categories and compared with established health-system functions, including planning and governance, service delivery, workforce capacity, medicines and technologies, financing, and health information systems.

This process resulted in nine operational domains:

1. Strategic and operational planning;
2. Service delivery;
3. Medicines, supplies, and logistics;
4. Training and capacity building;
5. Financing and budget planning;
6. Community engagement and support;
7. Intersectoral coordination and partnerships;
8. Monitoring, evaluation, and learning;
9. Health information systems and digital technologies.

The analytic approach is therefore described as predominantly inductive, followed by deductive comparison and organization using established health-system concepts. The nine domains are presented as an operational synthesis of the included evidence rather than as an entirely new health-system taxonomy. Sources could contribute to more than one NCD group, crisis type, or operational domain. Accordingly, these categories were not mutually exclusive, and totals across categories could exceed the total number of included sources.

### Derivation of cross-cutting priorities

The cross-cutting priorities were derived through a structured, post hoc synthesis of the source-level extraction and evidence-profile tables. Two reviewers (AB and SK) independently identified candidate actions that recurred across NCD groups, crisis contexts, and operational domains. Their candidate lists were compared and consolidated through discussion, with consultation of a third reviewer (MB) when agreement could not be reached.

No fixed source-count threshold, formal thematic-saturation criterion, or inter-coder reliability statistic was applied because the included source types and operational-domain assignments were heterogeneous and non-mutually exclusive. Candidate actions were retained as cross-cutting priorities when they: (1) addressed a recurrent and documented continuity-of-care failure; (2) were supported by more than one form of evidence, including empirical or operational evidence and guidance or policy sources; (3) were relevant across multiple NCD or crisis contexts; and (4) could be translated into measurable implementation indicators. The resulting priorities were evidence-informed and were not formally ranked.

### Characterization of evidence supporting the identified domains

To clarify the nature and directness of the evidence supporting the identified domains, the included sources were additionally examined for whether they reported: (1) direct patient or clinical outcomes; (2) service-delivery, health-system, or implementation outcomes; or (3) descriptive findings or recommendations without an evaluated outcome. Sources were also checked for explicitly reported economic evidence, including formal economic evaluations, costing analyses, budget-impact estimates, or descriptive cost information. These classifications were used only to characterize the available evidence and were not treated as a formal assessment of methodological quality or certainty of evidence.

## Results

### Selection of sources of evidence

**Fig 1:**
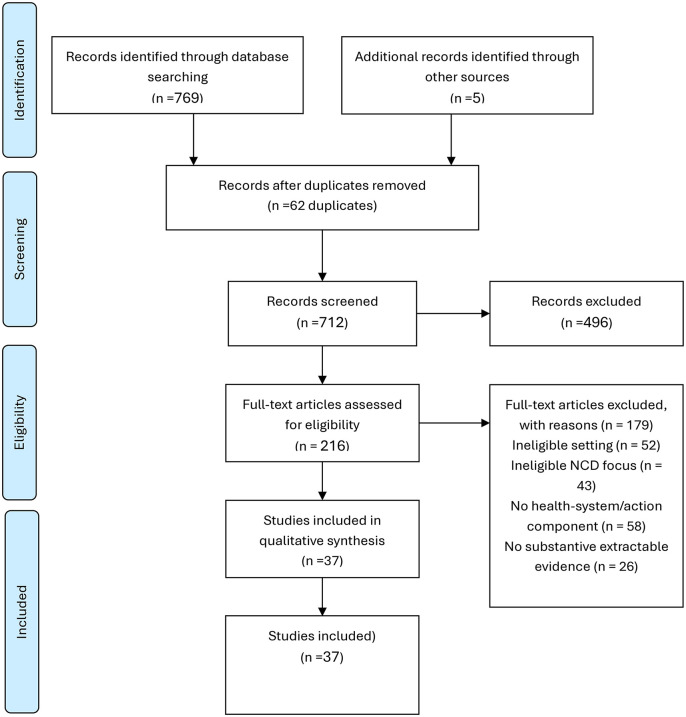
PRISMA-ScR flow diagram of source identification, screening, eligibility assessment, and inclusion.

The database searches identified 774 records. After removal of 62 duplicates, 712 records underwent title and abstract screening, of which 496 were excluded. The full texts of 216 records were subsequently assessed for eligibility. At this stage, 179 records were excluded because they did not address an eligible humanitarian or crisis setting, did not examine one of the four prespecified NCD groups, did not report a health-system, continuity-of-care, preparedness, intervention, operational, or policy component, represented an ineligible publication type without substantive empirical or operational content, or did not meet the language criterion. Thirty-seven sources met the eligibility criteria and were included in the final synthesis [13^,^^18^–^53^]. The source-selection process is presented in Fig 1, PRISMA-ScR flow diagram.

### Characteristics and distribution of the included evidence

The 37 included sources were published between 2013 and 2025. Twenty-one sources (56.8%) were published between 2022 and 2025, indicating that more than half of the included evidence was produced during the most recent four years of the review period. The evidence base comprised eight empirical primary studies, including observational, qualitative, and health-service assessments; one modelling and costing study; 16 systematic or narrative reviews, perspectives, and experience-based syntheses; and 12 operational guidelines, strategic frameworks, policy documents, or organizational reports. Because the included records represented heterogeneous forms of evidence, they are referred to collectively as “sources” rather than as independent studies or intervention evaluations. Their detailed characteristics are presented in S2 Table.

Ten sources examined a specific country or a clearly defined cross-border setting. Nine of these focused on Asian or Middle Eastern settings, including Japan [18,22,40], Lebanon [32,44], Pakistan [37], Iran [42], Thailand [43], and the Türkiye–Syria earthquake setting [48]. One empirical source was conducted in the United States during the COVID-19 pandemic [38]. The remaining 27 sources were global, regional, or multicountry reviews, frameworks, guidelines, and reports. Thus, although the evidence covered multiple world regions, directly observed country-level evidence was concentrated predominantly in Asia and the Middle East, with relatively little dedicated empirical research from Africa and Latin America.

The humanitarian contexts were heterogeneous and frequently overlapping. They included armed conflict, forced displacement and refugee settings; earthquakes, floods, storms, hurricanes, and other natural disasters; COVID-19 and other public-health emergencies; and economic or political crises affecting the availability and continuity of health services. Several sources addressed more than one crisis type, particularly global reviews and all-hazards operational guidance, and the crisis categories were therefore not mutually exclusive.

The four prespecified NCD groups were also represented unevenly and frequently appeared together within the same source. Cardiovascular disease or hypertension was addressed in 31 sources, diabetes in 30, cancer in 20, and chronic respiratory diseases, including asthma and chronic obstructive pulmonary disease, in 18. Mental-health conditions and psychosocial distress were discussed in several sources as comorbidities, determinants of treatment continuation, or components of integrated NCD care, but were not treated as an independent target disease group. The full distribution of sources by design, setting, crisis context, NCD focus, implementation status, and type of evidence is provided in S3 and S4 Tables.

### Evidence profile and directness across operational domains

The 37 included sources contributed to one or more operational domains; therefore, domain totals were non-mutually exclusive. Service delivery was the most frequently represented domain (n = 36), followed by medicines, supplies, and logistics (n = 32); strategic and operational planning and community engagement and support (n = 30 each); training and capacity building (n = 29); health information systems and digital technologies (n = 25); intersectoral coordination and partnerships (n = 24); financing and budget planning (n = 22); and monitoring, evaluation, and learning (n = 18). Source-level classifications are presented in S3 and S4 Tables.

The directness of evidence varied substantially across domains. Service delivery had the strongest outcome-based profile, including seven empirical primary studies, nine sources reporting patient or clinical outcomes, and 13 reporting service, system, or implementation outcomes. Medicines, supplies, and logistics also had relatively direct evidence, with four empirical primary studies, seven patient- or clinical-outcome sources, and 12 service or implementation sources. Evidence relating to information systems and digital technologies was mixed: four empirical studies, seven patient-outcome sources, and eight service or implementation sources were identified, but 10 sources remained descriptive or recommendation-based.

Governance- and system-enabling domains were supported by less direct evidence. Strategic and operational planning included five patient-outcome and 11 service or implementation sources, but 14 of 30 sources were descriptive or recommendation-based. In financing, only two empirical primary studies, three patient-outcome sources, and six service or implementation sources were identified, while 13 of 22 sources were descriptive or recommendation-based. Intersectoral coordination showed a similar pattern, with three empirical studies, three patient-outcome sources, eight service or implementation sources, and 13 descriptive or recommendation-based sources. Training and community engagement also contained implemented examples, but substantial proportions of their evidence consisted of programme descriptions, expert perspectives, or recommendations rather than evaluated outcomes.

Economic evidence was limited to costing, affordability, expenditure, or resource-use information. Such information was reported in four financing sources, three service-delivery sources, two medicines and logistics sources, and one strategic-planning source. The most detailed analysis estimated the cost of adding essential NCD medicines to emergency health kits [13]. No full comparative cost-effectiveness, cost-utility, or cost-benefit evaluation was identified.

Overall, direct outcome evidence was concentrated in service delivery, medicine continuity, monitoring, and selected digital-health interventions, whereas financing, coordination, and strategic governance were supported predominantly by indirect, descriptive, or recommendation-based evidence. The classifications in Supplementary S2 Table represent an evidence profile and an assessment of directness; they are not a formal grading of methodological quality, risk of bias, or certainty of evidence.

### Reported health-system challenges affecting continuity of NCD care

Across the included sources, challenges to NCD care clustered into four interconnected pathways: disruption of service continuity and access; failure of medicines, technologies, and infrastructure; workforce, coordination, and information constraints; and financial, social, and patient-level barriers. These challenges recurred across cardiovascular disease, diabetes, cancer, and chronic respiratory disease and often overlapped within the same crisis setting.

#### Disruption of continuity and access.

Interruptions in follow-up, referral, diagnosis, and treatment were among the most consistently reported challenges. Facility damage, displacement, travel restrictions, and reallocation of services reduced access to both primary and specialist care. Cancer services were especially vulnerable because treatment depended on specialized facilities, equipment, and repeated visits. Following Hurricane Katrina, one hospital reported a 70% reduction in patient volume, 34% of patients remained displaced for more than six months, and the average interval from initial presentation to cancer diagnosis increased by approximately two months. Elsewhere, 79 patients experienced radiotherapy delays of 7–14 days, while more than 100 paediatric oncology patients required relocation to hospitals in other states [26].

Routine cardiometabolic care was also disrupted. During the COVID-19 response in Bangkok, fewer than one-quarter of facilities maintained essential diabetes services for more than 95% of their target population. Diabetes follow-up coverage declined from 18.4% to 12.0%, and home-visit coverage from 6.5% to 4.1% across pandemic waves [43]. During recovery from the Kumamoto earthquake, 31.2% of respondents with hypertension were noncompliant with treatment, with cost, lack of time, and low perceived need for consultation among the reported reasons [40]. Conflict and displacement additionally produced fragmented referral pathways, delayed investigations, and discontinuity between humanitarian, governmental, and private providers [39].

#### Medicine, technology, and infrastructure failures.

Shortages of medicines, diagnostics, power, transport, and disease-specific equipment were reported across all four NCD groups. Among Syrian refugees with type 1 diabetes in Iraq, 63.6% reported symptomatic hypoglycaemia, 36.6% experienced at least one episode of diabetic ketoacidosis, mean HbA1c was 9.3%, and 81.8% had HbA1c values above 8.0%, indicating marked instability of treatment and monitoring under displacement conditions [28].

Cardiovascular care was affected by interrupted antihypertensive treatment and reduced monitoring. In conflict-affected Kosovo, only 5.5% of patients with hypertension were receiving regular treatment, while after the Sichuan earthquake, 43.4% of 762 assessed patients had blood pressure in the hypertensive range [36]. Respiratory care was constrained by shortages of inhalers, oxygen, nebulizers, and reliable electricity. Oncology services experienced flooding of medicine-storage areas, loss of refrigerated medicines, damage to radiotherapy equipment, and closure of pharmacies and treatment units [26].

Infrastructure failures also affected clinical information. Paper records, treatment protocols, pathology material, radiation plans, and medication histories were lost or inaccessible after flooding, evacuation, or facility closure. Electricity, telephone, and internet outages limited communication, while damaged roads and disrupted transport delayed access to functioning facilities [18,26].

#### Workforce, coordination, and information constraints.

The included sources described shortages, reassignment, displacement, and uneven preparedness of health workers. During the second COVID-19 wave in Bangkok, 77.8% of facilities reported that personnel responsible for diabetes care had been partially or fully reassigned to pandemic response, reducing follow-up, counselling, and community outreach [43]. In facilities dependent on one nurse or a small number of trained staff, reassignment could interrupt routine diabetes services almost completely.

Qualitative and review evidence identified limited NCD-specific training, inconsistent use of protocols, and uncertainty among non-specialist personnel. General practitioners, nurses, emergency staff, and volunteers were often required to manage insulin adjustment, hypertension, cardiovascular emergencies, or cancer-related needs without standardized procedures or adequate specialist support [18,33,42].

Coordination failures further fragmented care. Referral responsibilities between humanitarian agencies, public facilities, private providers, and specialist centres were often unclear, and records were not consistently shared. Patients in Lebanon frequently carried prescriptions and laboratory results between facilities, increasing duplication, delay, and the risk of incomplete treatment histories [44]. Communication-system failure after disasters also hindered clinician handover, patient tracing, and coordination of transfers [26].

#### Financial, social, and patient-level barriers.

Affordability was a major determinant of interrupted care. Reduced income, user fees, transport expenses, diagnostic costs, and medicine prices limited patients’ ability to maintain treatment even when services remained available. In the Kumamoto cohort, pandemic-related income reduction was associated with hypertension-treatment noncompliance (adjusted OR 3.23, 95% CI 2.27–4.58), while poor self-rated health was also associated with noncompliance (adjusted OR 2.49, 95% CI 1.72–3.61) [40].

Housing instability, displacement, and food insecurity created additional barriers. Irregular or nutritionally unsuitable food complicated diabetes and hypertension management; in shelters, high-carbohydrate and high-salt foods contributed to poor glycaemic and blood-pressure control, while uncertain meal timing complicated medication adjustment [18]. Psychological distress also affected adherence. After the Great East Japan earthquake, severe psychological distress was associated with fourfold higher odds of withdrawing from hypertension treatment (OR 4.0, 95% CI 1.3–10.6) [22].

Qualitative evidence from Lebanon showed that patients and families often compensated for service fragmentation by searching for medicines, financing consultations, transporting patients, and carrying records between providers. Some households had to choose between food, medicines, laboratory tests, and clinical visits, while family networks became essential for obtaining insulin and maintaining contact with services [44]. These findings indicate that continuity failures reflected both health-system disruption and household-level financial, social, and psychological vulnerability.

### Implemented strategies and evaluated outcomes

Implemented responses were identified mainly in service delivery, medicine continuity, information systems, telemedicine, workforce development, and community-based support. Most were multicomponent programmes or operational adaptations rather than controlled evaluations of a single strategy. Consequently, reported outcomes generally reflected the combined effects of service organization, medicines, workforce inputs, follow-up, and information systems.

#### Service-delivery adaptations.

The most consistently implemented service model was the integration of standardized NCD care into existing primary or outpatient services. In clinics serving Palestinian refugees in Jordan, structured hypertension and diabetes algorithms combined scheduled follow-up, lifestyle counselling, pharmacological treatment, complication screening, and electronic cohort monitoring. Among 4,130 patients with hypertension, 76% remained in care, and 74% of those with recorded measurements achieved blood pressure below 140/90 mmHg. Among 12,550 patients with diabetes, 78% remained in care; 65% achieved the postprandial glucose target, 63% achieved the cholesterol target, and 87% achieved the blood-pressure target. However, implementation was incomplete for some components, including foot and ophthalmological assessment [19].

A multicomponent heart-failure disease-management programme was implemented in conflict-affected Georgia. The programme included free outpatient services, physician training, patient education, equipment provision, and protocol-based pharmacological management. Of 400 enrolled adults, 337 completed follow-up. Mean ejection fraction increased by 4.1 percentage points, systolic blood pressure decreased by 30.9 mmHg, and diastolic blood pressure decreased by 17.8 mmHg. Hospital admissions declined by 52.5% and emergency-room visits by 40.7%, with all reported changes reaching p < 0.001. Because the programme incorporated several simultaneous components, the contribution of any single component could not be isolated [19].

Remote service delivery was also used when in-person access was restricted. During the COVID-19 pandemic in the United States, a hypertension telemedicine programme combined video consultations, repeated home blood-pressure measurements, medication adjustment, and lifestyle assessment. Among 569 patients, mean systolic and diastolic blood pressure decreased by 9.7 and 6.8 mmHg, respectively. Among those initially uncontrolled, 55.7% achieved blood-pressure control; 77% experienced an improvement in systolic or diastolic pressure, and 84% received a new antihypertensive prescription. Patient acceptability was high, with 96.6% rating the video visit as 4 or 5 on a five-point scale [38].

In Pakistan, mobile, doorstep, and teleconsultation services were used following floods and earthquakes to extend diabetes care beyond functioning facilities. The programme combined clinical assessment, medicine provision, diabetes education, and diabetic-foot services and reached approximately 1,800 people with diabetes. The operational reports demonstrated reach and feasibility, although changes in glycaemic control, complications, hospital admission, or mortality were not formally reported [37].

Dedicated NCD clinic days were implemented in the Bidibidi refugee settlement, supported by village health teams and peer “buddy groups.” Clinic attendance reportedly increased by 75%, and 65% of enrolled patients showed improvement in condition control within six months. These outcomes were reported for the NCD programme collectively rather than separately for hypertension, diabetes, or other conditions [50].

#### Medicine and logistics interventions.

Several sources described operational adaptations intended to maintain access to medicines and equipment during supply-chain or infrastructure failure. Following the Great East Japan earthquake, diabetes services distributed practical insulin-use guidance, information on storage during power outages, and advice on dose adjustment when meals and glucose monitoring were unpredictable. Visual medication catalogues were used to help patients identify medicines when prescriptions and records had been destroyed, and printed notices were posted at health facilities when internet and telephone communication failed. These measures were implemented as emergency adaptations, but their effects on glycaemic control, treatment interruption, or acute complications were not formally evaluated [18].

Oncology services used emergency relocation and medicine-preservation procedures during hurricanes. After Hurricane Katrina, oncology medicines, investigational products, treatment protocols, and paediatric patients were transferred to functioning facilities. More than 100 children with cancer were relocated to hospitals in other states, and damaged radiotherapy services required patients to be replanned or transferred. These actions enabled partial continuation of treatment but were implemented reactively after substantial service interruption; no comparative evaluation of alternative relocation or stock-preservation strategies was reported [26].

Following the Marmara earthquake, haemodialysis capacity was progressively restored through recovery of equipment, personnel, and functioning centres. Weekly dialysis sessions fell from 1,093 before the earthquake to 520 immediately afterwards, then increased to 616 after one month and 729 after three months. The number of patients receiving dialysis declined from 439 before the earthquake to 175 immediately afterwards and subsequently increased to 239 at one month and 288 at three months. Functioning dialysis machines increased from 74 at one month to 79 at three months, while staffing increased from 86 to 94 personnel. These data documented partial system recovery but did not evaluate a predefined logistics intervention against a comparator [19].

During the COVID-19 response in Bangkok, medicine and clinical-supply availability for hypertension were largely maintained despite workforce disruption. Regular supply levels were reported by 75.3% of facilities during the first wave and 74.1% during the second wave; the proportion reporting inadequate supplies increased from 3.7% to 6.2%. The findings indicate relative preservation of basic hypertension commodities, although diabetes services requiring more intensive monitoring and follow-up were less resilient [43].

In Cox’s Bazar, local NCD programmes combined essential medicine procurement with decentralized screening and care. Antihypertensive and other NCD medicines were incorporated into secured supply arrangements, alongside diagnostic tools and local workforce training. The programme demonstrated operational deployment of an emergency NCD supply package, but source-specific stock-out rates, refill continuity, and patient outcomes were not reported [50].

#### Information, monitoring, and digital adaptations.

Electronic medical records were used to register cohorts, schedule follow-up, monitor clinical targets, and identify missed care in refugee health services. In the Jordanian hypertension and diabetes programmes, EMR-supported follow-up accompanied retention rates of 76% and 78%, respectively, and enabled routine measurement of blood pressure, glucose, cholesterol, renal function, and complications [19]. In UNRWA diabetes clinics, documentation of postprandial glucose increased from 42% among 2,851 patients to 99% among 9,691 patients one year later, while the proportion meeting the glucose-control target increased from 50% to 65%. These findings indicate improved monitoring and measured control after the introduction of structured electronic follow-up, although the contribution of the information system could not be separated from concurrent clinical changes [28].

Digital follow-up was also implemented during the COVID-19 pandemic. The United States telemedicine programme used home blood-pressure readings and video visits to support medication adjustment and achieved measurable improvements in blood-pressure control [38]. In Bangkok, mHealth platforms allowed patients with diabetes to upload glucose readings and receive remote consultations. Their use demonstrated operational feasibility during restricted face-to-face care, but the study did not separately report the number of users, adherence to digital follow-up, or changes in glycaemic outcomes attributable to the applications [43].

Emergency communication systems were introduced in oncology settings after conventional telephone and internet systems failed. Online platforms and clinician-to-clinician handovers supported communication between displaced patients, referring providers, and receiving oncology centres. Treatment plans and other documentation were transferred where available, but the effects on referral completion, treatment delay, or clinical outcomes were not quantified [26].

#### Community and workforce interventions.

A structured Hurricane Action Plan was implemented for families of children receiving cancer treatment. The programme included evacuation planning, medicine checklists, treatment documentation, and patient and caregiver education. After implementation, 78% of families reported having an evacuation plan, 71% had stored at least two weeks of medicines, and 43% carried a treatment plan during displacement. Overall, 92% of families rated the plan as extremely useful. This was one of the clearest examples of a preparedness intervention linked to measurable family-level implementation outcomes [26].

Community-health-worker outreach, village health teams, buddy groups, and household-based follow-up were implemented in several settings. In Bidibidi, village health teams and buddy groups supported attendance, adherence, and follow-up within dedicated NCD clinic days, accompanying a 75% increase in attendance and improvement in condition control among 65% of enrolled patients within six months [50]. In Pakistan, community-oriented and doorstep services expanded access to approximately 1,800 people with diabetes, although clinical outcomes were not reported separately [37].

Workforce training was commonly delivered as part of broader programmes. The Georgia heart-failure programme included physician training and was associated with improvements in cardiac function, blood pressure, hospitalization, and emergency visits, but the independent effect of training could not be determined [19]. In Cox’s Bazar, local health workers were trained to conduct blood-pressure screening, recognize NCD presentations, and initiate basic management; the training enabled decentralized delivery but was not accompanied by pre- and post-training competency measures or patient-level effectiveness outcomes [50].

Community-based psychosocial screening was implemented after the Great East Japan earthquake through household surveys and outreach. The programme identified patients who had discontinued hypertension treatment and demonstrated that severe psychological distress was associated with fourfold higher odds of treatment withdrawal. The study established the feasibility and relevance of community detection, although it did not evaluate whether subsequent psychosocial referral or support restored treatment continuity [22].

Overall, the implemented strategies demonstrated that continuity of NCD care could be partially maintained through integrated clinics, remote care, structured follow-up, medicine and equipment recovery, electronic monitoring, community outreach, and workforce adaptation. However, most interventions were multicomponent, observational, or documented through operational experience. Few used comparison groups, prospective implementation outcomes, long-term follow-up, or economic evaluation, limiting attribution of effects to individual strategies.

### Recommended strategies without evaluated implementation

Operational guidelines, policy briefs, and strategic frameworks repeatedly recommended that NCD care be incorporated into all-hazards emergency preparedness, response, and recovery plans rather than being treated as a secondary activity after acute needs had been addressed. Common recommendations included establishment of a minimum emergency NCD service and medicine package, protection of dedicated NCD financing, development of standardized clinical and referral protocols, maintenance of patient registries or patient-held records, and use of interoperable information systems to preserve continuity across facilities and organizations [10,29,46,47,52].

The same sources also emphasized multisectoral coordination among health authorities, humanitarian agencies, logistics providers, community organizations, and specialist services, together with formal involvement of patients and affected communities in planning, communication, and service evaluation. Additional recommendations included workforce preparedness, task-sharing, multi-month medicine dispensing, protected supply chains, and routine monitoring of service coverage and medicine availability [45,49–51].

These strategies were predominantly presented in guidance, policy, and framework documents and were not accompanied by evaluated implementation or patient outcomes. Therefore, they represent areas of policy consensus and proposed system design rather than evidence of demonstrated effectiveness in humanitarian settings.

### Contribution of reported strategies to health-system resilience

The reported strategies contributed to three complementary dimensions of health-system resilience: absorptive, adaptive, and transformative capacity. These categories were applied as an interpretive crosswalk across the operational domains rather than as mutually exclusive classifications, because several interventions served more than one resilience function.

#### Absorptive strategies.

Were intended to maintain essential NCD functions during the immediate shock without substantially changing the existing model of care. Reported examples included emergency medicine stocks, backup electricity and refrigeration, continuation of pre-crisis treatment, identification of high-risk patients, emergency referral arrangements, and temporary transfer of patients, medicines, or services to functioning facilities. Following the Great East Japan earthquake, continued insulin access, emergency storage guidance, and simplified treatment instructions were used to reduce interruption of diabetes care during electricity, communication, and supply failures [18,26,41]. In cancer care, medicines and paediatric oncology patients were relocated to operational hospitals after Hurricane Katrina, while damaged radiotherapy services were transferred or reorganized to permit partial treatment continuation [26,48]. These actions primarily preserved existing functions during acute disruption and therefore represented absorptive responses.

#### Adaptive strategies.

Altered how services were organized or delivered in response to prolonged disruption or reduced access. These included mobile and doorstep clinics, telemedicine, task-sharing, flexible treatment protocols, home delivery of medicines, electronic or patient-held records, and modified follow-up arrangements. Telemedicine-supported hypertension management during the COVID-19 pandemic combined remote consultation, home blood-pressure monitoring, and medication adjustment and was associated with improved blood-pressure control [31,38]. Mobile, doorstep, and teleconsultation services extended diabetes care to approximately 1,800 people following floods and earthquakes in Pakistan [37]. Electronic cohort monitoring, standardized follow-up, and integrated primary-care models were also used to maintain hypertension and diabetes services among refugee populations [19,28,41]. These approaches modified routine delivery mechanisms while maintaining the underlying objectives of chronic-disease care.

#### Transformative strategies.

Involved longer-term changes to governance, financing, information systems, workforce organization, and community participation. Commonly proposed measures included formal integration of NCDs into national all-hazards emergency plans, protected and sustained financing, interoperable information systems, multisectoral governance structures, standardized NCD indicators, institutionalized task-sharing, and routine participation of affected communities in planning and evaluation [10,29,45,46,49,52]. Unlike many absorptive and adaptive actions, these measures sought to modify the underlying organization of the health system rather than only preserve or temporarily adjust existing services.

Overall, empirical evidence was concentrated mainly on absorptive and adaptive strategies, particularly medicine continuity, service reorganization, telemedicine, electronic monitoring, and community outreach. Transformative strategies were identified primarily in operational guidelines, strategic frameworks, and policy documents and were rarely accompanied by evaluated implementation, longitudinal system outcomes, or direct patient outcomes. The evidence therefore demonstrated the feasibility and potential benefits of selected short- and medium-term adaptations more clearly than the effectiveness of longer-term structural reforms.

### Evidence gaps, geographic distribution, and transferability

The evidence map showed substantial gaps in both the directness and applicability of the available evidence. Across the nine operational domains, direct patient or clinical outcomes were reported less frequently than process, service-delivery, descriptive, or recommendation-based findings. Several apparently effective actions were reported as components of multicomponent programmes, making it difficult to attribute observed outcomes to a specific intervention. For example, improvements in hypertension, diabetes, and heart-failure outcomes occurred within programmes combining protocols, medicines, training, follow-up, and information systems rather than evaluations of individual implementation strategies [19,28]. Similarly, qualitative studies identified operational barriers and feasible care models but did not assess comparative effectiveness [33,42,44].

Formal implementation evaluations were uncommon. Most sources did not report implementation fidelity, reach, adoption, acceptability, sustainability, or maintenance using standardized definitions. Evidence regarding scalability beyond the original setting was also limited, particularly for mobile services, telemedicine, task-sharing, and community-based models [37,38,50]. Although several sources emphasized patient and community engagement, few documented formal co-design with people living with NCDs, caregivers, or frontline practitioners, and few evaluated whether participation affected service use or outcomes [30,49].

Economic evidence was particularly limited. Available sources provided costing, affordability, expenditure, or medicine-price information, but no full comparative cost-effectiveness, cost-utility, or cost-benefit evaluation was identified. The most detailed analysis estimated the quantities and costs of adding NCD medicines to an emergency health kit but did not compare costs and outcomes with alternative strategies [13].

Country-specific empirical evidence was concentrated mainly in Asia and the Middle East, including Japan, Lebanon, Pakistan, Iran, Thailand, and the Türkiye–Syria earthquake setting [18,22,37,42–44,48]. Dedicated empirical evidence from Africa and Latin America was limited, although these regions appeared within global reviews and operational reports. Few sources systematically compared the feasibility, resource requirements, or effects of the same strategy across lower-resource humanitarian settings and higher-resource emergency contexts. Consequently, transferability could not be assumed for interventions dependent on stable electricity, digital connectivity, specialist workforces, or established referral systems.

Evidence supporting transformative resilience was also limited. Longer-term reforms such as protected financing, interoperable information systems, multisector governance, institutionalized community participation, and formal integration of NCDs into national emergency systems were described mainly in strategic frameworks and guidance documents rather than evaluated programmes [10,23,29,45,47,52]. Across all domains, the absence of standardized implementation and outcome indicators restricted comparison between settings and synthesis of programme performance.

### Cross-cutting evidence-informed priorities

Application of the predefined synthesis criteria yielded three cross-cutting priorities supported across multiple NCD groups, crisis contexts, and evidence types. These priorities arose from the narrative synthesis and should not be interpreted as a formally scored or ranked set of recommendations.

#### Establish a minimum emergency NCD service and medicine package.

The package should include tracer medicines, essential diagnostics, cold-chain arrangements, backup power, and predefined multi-month medicine supplies. This priority was supported by repeated evidence of medicine interruption, equipment failure, and treatment discontinuity across diabetes, cardiovascular, respiratory, and cancer care [13,18,26,36]. Suggested indicators are the proportion of facilities with all tracer medicines available, the number of stock-out days, cold-chain or backup-power availability, and the proportion of eligible patients receiving multi-month refills.

#### Establish continuity mechanisms for patients at the highest risk of harm from treatment interruption.

These mechanisms should combine advanced identification or registries, patient-held records, documented referral and evacuation pathways, multi-month dispensing, and structured follow-up. Evidence from hypertension, diabetes, and oncology settings showed that displacement, lost records, fragmented referrals, and psychological distress increased treatment interruption and reliance on patients and families to navigate care [8,22,26,44]. Suggested indicators are the proportion of eligible high-risk patients registered, the proportion with accessible clinical information, uninterrupted-treatment rates, follow-up completion, and referral completion.

#### Integrate simplified NCD care into emergency and primary-care delivery.

This priority includes context-adapted protocols, task-sharing, mobile or outreach services, telemedicine where feasible, and standardized monitoring. Integrated clinics and remote-care programmes demonstrated measurable improvements in retention, blood-pressure control, monitoring, and service reach, although effects were usually generated by multicomponent models [19,31,37,38]. Suggested indicators are the proportion of facilities with simplified protocols, staff training coverage, NCD service coverage, referral completion, and selected clinical outcomes such as blood-pressure or glycaemic control.

## Discussion

This scoping review maps how health-system challenges, implemented responses, and proposed strategies for maintaining NCD care have been documented across humanitarian crises. It does not directly measure or rank the preparedness of individual countries, health systems, or humanitarian organizations. Similarly, the nine operational domains should not be interpreted as a new preparedness taxonomy, because they overlap substantially with established health-system and humanitarian frameworks. The principal contribution of this review lies instead in distinguishing implemented and evaluated actions from descriptive experiences and unevaluated recommendations, characterizing the directness of the available evidence, and examining how reported strategies contribute to absorptive, adaptive, and transformative resilience.

The evidence map revealed a clear imbalance between policy consensus and evaluated effectiveness. Direct patient and clinical outcomes were concentrated in a relatively small group of service-delivery, medicine-continuity, monitoring, and digital-health interventions. In contrast, financing, governance, intersectoral coordination, community participation, and longer-term system transformation were supported mainly by descriptive reports, expert perspectives, operational frameworks, and policy guidance. Previous reviews have identified a similar pattern: the literature increasingly describes context-adapted models for NCD care in crises, but comparatively few programmes have been evaluated using direct patient outcomes, implementation measures, comparison groups, or economic analyses [19,54]. A broader review of humanitarian health interventions also found that evidence on the effectiveness of NCD interventions remained relatively underdeveloped compared with several other humanitarian health fields [55].

Interpreting the reported strategies through absorptive, adaptive, and transformative resilience provides a clearer analytical framework than treating all preparedness actions as equivalent. Absorptive strategies preserve existing services during the immediate shock, for example, through emergency medicine stocks, backup power, temporary transfers, emergency referral arrangements, and continuation of pre-crisis treatment. Adaptive strategies modify the mode of service delivery when routine systems cannot function normally, including mobile clinics, telemedicine, task-sharing, home delivery, modified follow-up, and patient-held records. Transformative strategies seek longer-term changes in financing, governance, information systems, workforce organization, and community participation.

The concentration of empirical evidence on absorptive and adaptive actions is understandable. Such strategies can often be introduced within a defined programme and assessed through immediate measures such as attendance, medicine availability, treatment retention, service coverage, or disease control. Transformative changes require longer time horizons, institutional authority, sustained financing, and coordination across multiple organizations. They are therefore more difficult both to implement and to evaluate. Reviews of health-system resilience in fragile and conflict-affected settings have similarly reported limited empirical investigation of how absorption, adaptation, and transformation develop over time, with many publications remaining conceptual or descriptive [56].

Temporary adaptation should not, however, be equated automatically with health-system strengthening. Mobile clinics, donated medicines, parallel humanitarian services, and reliance on family networks may preserve access during acute disruption while leaving underlying weaknesses in financing, referral, governance, and workforce capacity unchanged. Conversely, technologically advanced interventions are not necessarily the most resilient. Simplified protocols, multi-month dispensing, paper-based patient-held records, and clearly assigned referral responsibilities may be more reliable than electronic systems in settings with unstable electricity, connectivity, or security. Operational guidance therefore, appropriately emphasizes adapting service models to the crisis phase, baseline health-system capacity, available infrastructure, security conditions, and expected duration of disruption rather than applying a uniform model across settings [57].

The available evidence supports integrated and simplified delivery of NCD care but does not identify one universally preferred model. Structured primary-care programmes, remote consultations, mobile services, community outreach, electronic monitoring, and multicomponent disease-management programmes were associated with improvements in selected clinical and service outcomes. For example, integrated diabetes and hypertension care among Syrian refugees in Lebanon combined consolidated consultations, laboratory assessment, medicine provision, task-sharing, and reduced visit frequency, illustrating how several continuity functions can be delivered within one primary-care model [58].

Most reported programmes were multicomponent, however, and the observed outcomes cannot be attributed confidently to any single element. Improvements may have reflected the combined effects of medicines, protocols, staff training, follow-up systems, patient education, and information management. This limits causal interpretation but does not make the evidence operationally irrelevant. Humanitarian interventions are inherently complex, and the relevant implementation question is frequently whether an integrated package can be delivered, accepted, maintained, and adapted under changing conditions.

Future evaluations should therefore specify the programme theory, core and adaptable components, target population, contextual determinants, and the mechanisms through which outcomes are expected to occur. They should also report implementation measures such as reach, adoption, fidelity, acceptability, feasibility, equity, cost, and maintenance alongside clinical outcomes. The emphasis on iterative testing of integrated NCD models is particularly relevant because it treats adaptation and implementation as outcomes requiring evaluation rather than as undocumented background processes [49].

The findings also indicate that patient and household capacity should not be treated as an unlimited substitute for health-system capacity. Families often searched for medicines, financed care, transported patients, preserved records, and navigated fragmented referral systems. Yet psychological distress, unstable housing, food insecurity, displacement, and loss of income could make continued treatment impossible even when some services remained available [22,44]. Person-centred preparedness should therefore support self-management without transferring disproportionate responsibility for continuity to patients and caregivers. Education, patient-held records, peer support, and community-health-worker involvement require functioning referral pathways, affordable medicines, and accessible services to be effective [5,44].

The economic evidence requires cautious interpretation. The included sources provided information on medicine prices, programme expenditure, affordability barriers, quantities of required supplies, and resource use. Tonelli et al. demonstrated that estimating the quantities and costs of incorporating essential NCD medicines into an emergency health kit was technically feasible [13]. This type of costing is useful for planning and budget preparation, but it is not equivalent to demonstrating cost-effectiveness.

No full comparative cost-effectiveness, cost-utility, or cost-benefit evaluation was identified. Consequently, the present review cannot determine whether one intervention, delivery model, or operational domain provides better value for money than an alternative. This gap is important because humanitarian decision-makers must allocate limited resources across trauma, communicable diseases, nutrition, maternal and child health, mental health, and chronic care. Future studies should therefore examine budget impact, affordability, opportunity costs, distributional effects, and patient outcomes over relevant time horizons. Priority candidates for economic evaluation include multi-month dispensing, decentralized care, telemedicine, essential diagnostic packages, emergency medicine stocks, and referral pathways for resource-intensive cancer or cardiovascular treatment.

The evidence does not support direct transfer of a single service model across all crisis settings. Most country-specific empirical evidence was generated in Asia and the Middle East, with relatively limited dedicated research from Africa and Latin America. A more defensible approach is to distinguish a minimum core package from context-dependent extensions. The minimum package may include essential tracer medicines, basic diagnostics, simplified treatment protocols, identification of patients at high risk from interruption, accessible clinical records, and documented referral arrangements. Additional components, such as interoperable electronic systems, advanced telemedicine, specialist networks, or complex oncology pathways, would depend on pre-crisis infrastructure, connectivity, workforce, financing, security, and the duration of the emergency. This layered approach avoids assuming that models developed in well-resourced disaster settings are feasible in protracted displacement or conflict, while also avoiding the assumption that lower-resource settings can provide only acute or minimal NCD care.

Several requirements identified in this review, including medicine continuity, accessible clinical information, referral pathways, psychosocial support, and protection from catastrophic expenditure, are also relevant to other chronic conditions requiring uninterrupted care, such as severe mental illness and dialysis-dependent kidney disease. Nevertheless, these conditions involve distinct clinical, technological, and infrastructure requirements and were not systematically examined in the present review. Cross-condition transferability should therefore be assessed directly rather than inferred from the findings concerning the four included NCD groups.

Prioritizing continuity of NCD care should not be framed as displacing immediate life-saving responses for trauma, communicable diseases, nutrition, or maternal and child health. Rather, phased and context-sensitive allocation should protect a minimum set of NCD functions during the acute response to prevent avoidable decompensation and additional demand for emergency care, while expanding chronic-care services as response capacity stabilizes. Decisions should explicitly consider local disease burden, crisis phase, available delivery capacity, equity, and the opportunity costs of alternative resource uses.

The synthesis supports replacing general calls for “integration” with a limited set of operational and measurable priorities. The first is the establishment of a minimum emergency NCD service and medicine package, monitored through tracer-medicine availability, stock-out days, diagnostic capacity, backup arrangements, and provision of multi-month refills. The second is establishment of continuity mechanisms for patients at highest risk from treatment interruption, including advance identification, accessible records, documented referral or evacuation pathways, and structured follow-up. The third is integration of simplified NCD care into primary and emergency services through context-adapted protocols, task-sharing, mobile or remote alternatives, and standardized service and clinical indicators.

These priorities should not be interpreted as a formally ranked hierarchy. They were not derived through stakeholder voting, Delphi consensus, or quantitative priority-setting methods. Rather, they emerged from their recurrence across disease groups and crisis types, their relationship to documented continuity failures, and their potential to be monitored through practical indicators. Their relevance, feasibility, and equity implications should be assessed with patients, caregivers, frontline practitioners, national authorities, and humanitarian organizations before large-scale implementation.

### Strengths and limitations

A key strength of this review is its inclusion of diverse evidence sources, including empirical studies, programme reports, reviews, operational guidance, and policy documents, reflecting the way implementation knowledge is generated in humanitarian settings. The synthesis also distinguishes patient and clinical outcomes, service and implementation outcomes, descriptive evidence, and recommendation-only sources, while separately identifying the limited economic evidence.

Several limitations affect interpretation. Restriction to English-language sources may have excluded local-language and unpublished operational evidence. Country-specific evidence was concentrated mainly in Asia and the Middle East, with limited evidence from Africa and Latin America, reducing geographic transferability. No formal risk-of-bias or certainty assessment was undertaken; therefore, the evidence profile reflects evidence type and directness rather than methodological quality or certainty.

The evidence-directness classification was based on the outcomes and descriptions reported by the included sources and was not accompanied by independent verification of those claims or formal appraisal of the methodological robustness of the underlying evidence. Accordingly, classification as patient or clinical outcome evidence indicates the type of outcome reported and should not be interpreted as evidence of high methodological quality, causal validity, or certainty.

The synthesis also combined acute-onset, recovery, and protracted crisis settings without systematically stratifying findings by crisis phase. The feasibility, relative priority, and expected effects of individual strategies may therefore differ across phases of an emergency.

Heterogeneity in crises, diseases, interventions, source types, and outcomes prevented quantitative comparison of effects. The review also did not systematically compare feasibility across lower- and higher-resource settings, assess equity, or involve patients and practitioners in co-design or priority setting. Some reviews may have included overlapping primary evidence, and the operational domains were non-mutually exclusive. Although most sources were recent, some empirical evidence predated current developments in digital health and humanitarian coordination. Finally, the three priorities represent an evidence-informed narrative synthesis, not a formal consensus or ranking exercise.

## Conclusion

This review identifies a persistent gap between the growing policy consensus on NCD care in humanitarian crises and the limited evidence showing how recommended strategies are implemented, sustained, scaled, and translated into patient benefit. The evidence is most developed for selected absorptive and adaptive actions, including continuity of medicines, integrated primary care, mobile or remote delivery, structured follow-up, and preservation of clinical information. Transformative measures involving financing, governance, interoperable systems, and institutionalized community participation remain largely unevaluated.

The immediate implication is not simply to call for greater integration of NCD care, but to operationalize and prospectively test three measurable components: a minimum emergency NCD service and medicine package, continuity mechanisms for patients at highest risk from treatment interruption, and simplified integrated NCD care within emergency and primary-care services. These components should be adapted to local capacity and crisis phase and evaluated using standardized implementation, service, clinical, equity, and economic indicators. Advancing the field will require moving from guidance and isolated operational experience toward context-sensitive implementation research conducted with affected communities and frontline practitioners.

## Supporting information

S1 Table Database-specific search strategies. (DOCX)

S2 Table Characteristics and principal contributions of the included evidence sources. (DOCX)

S3 Table Evidence profile and directness of evidence across operational domains. (DOCX)

S4 Table Key findings by operational domain. (DOCX)

S1 File Completed PRISMA Extension for Scoping Reviews checklist, adapted from Tricco AC, Lillie E, Zarin W, et al. PRISMA Extension for Scoping Reviews (PRISMA-ScR): Checklist and Explanation. Ann Intern Med. 2018;169:467–473. Distributed under the Creative Commons Attribution 4.0 International License (CC BY 4.0).
(DOCX)
