# Supplementary material for: Health-system challenges, resilience strategies, and actionable priorities for non-communicable disease care in humanitarian crises: A scoping review
Source: PLOS Glob Public Health. 2026 Aug 18;6(8):e0006035. doi: 10.1371/journal.pgph.0006035 (PMC13485051; doi:10.1371/journal.pgph.0006035)
Supplement: Supplementary file 1 [file pgph.0006035.s001.docx]

**Table S1. Database-specific search strategies**

| Database | search strategies | Identified |
| --- | --- | --- |
| PubMed/MEDLINE | ( ( NCDs[Title] OR "non-communicable"[Title] OR noncommunicable[Title] OR "cardiovascular disease"[Title] OR CVD[Title] OR hypertension[Title] OR cancer[Title] OR "diabetes mellitus"[Title] OR diabetes[Title] ) AND ( humanitarian[Title] OR emergencies[Title] OR crisis[Title] OR disaster[Title] OR earthquake[Title] ) AND ( intervention[Title] OR care[Title] OR response[Title] OR services[Title] OR "services delivery"[Title] OR treatment[Title] ) ) AND ("2000/01/01"[Date - Publication] : "2025/12/31"[Date - Publication]) AND English[Language] AND ( "Journal Article"[Publication Type] OR Review[Publication Type] ) | **130** |
| Scopuse | TITLE ( ncds OR "non-communicable" OR noncommunicable OR "cardiovascular disease" OR cvd OR hypertension OR cancer OR "diabetes mellitus" OR diabetes ) AND TITLE ( humanitarian OR emergencies OR crisis OR disaster OR earthquake ) AND TITLE ( intervention OR care OR response OR services OR "services delivery" OR treatment ) AND PUBYEAR > 1999 AND PUBYEAR < 2026 AND ( LIMIT-TO ( DOCTYPE , "ar" ) OR LIMIT-TO ( DOCTYPE , "re" ) ) AND ( LIMIT-TO ( LANGUAGE , "English" ) ) | **497** |
| Web of Science | TI=( ncds OR "non-communicable" OR noncommunicable OR "cardiovascular disease" OR cvd OR hypertension OR cancer OR "diabetes mellitus" OR diabetes ) AND TI=( humanitarian OR emergencies OR crisis OR disaster OR earthquake ) AND TI=( intervention OR care OR response OR services OR "services delivery" OR treatment ) AND PY=(2000-2025) AND LA=(English) AND DT=(Article OR Review) | **147** |
| Total | | **774** |
