# Supplementary material for: Health-system challenges, resilience strategies, and actionable priorities for non-communicable disease care in humanitarian crises: A scoping review
Source: PLOS Glob Public Health. 2026 Aug 18;6(8):e0006035. doi: 10.1371/journal.pgph.0006035 (PMC13485051; doi:10.1371/journal.pgph.0006035)
Supplement: Supplementary file 2 [file pgph.0006035.s002.docx]

**Table S2. Characteristics and principal contributions of the included evidence sources** (1-37)

| Source | Country/region and humanitarian context | Source type/design | NCD focus | Main challenge addressed | Implemented or recommended strategy | Evidence/outcome type | Principal finding relevant to preparedness and resilience |
| --- | --- | --- | --- | --- | --- | --- | --- |
| Kishimoto & Noda, 2013 | Japan; Great East Japan earthquake, tsunami, and Fukushima nuclear accident | Narrative clinical review/experience report | Diabetes primarily; hypertension and respiratory disease also discussed | Loss of medicines and records, cold-chain and power failure, irregular meals, and unsuitable shelter conditions. | Described/recommended emergency kits, flexible insulin dosing, printed guidance, medication-identification tools, and manuals for non-specialists. | Descriptive and operational evidence | Patient preparedness and context-adapted protocols may preserve immediate continuity of care (absorptive/adaptive resilience); no comparative evaluation was reported. |
| Ruby et al., 2015 | Multiple conflict, refugee, and earthquake settings, including Jordan, Georgia, Afghanistan, India, and Türkiye | Systematic review | Hypertension, heart failure, diabetes, chronic kidney disease, thalassemia, and arthritis | Sparse, heterogeneous intervention evidence and major disruptions in chronic-care services. | Reviewed implemented disease-management programmes, EMR follow-up, free outpatient care, staff training, and restoration of dialysis services. | Patient/clinical and service outcomes | Some programmes improved blood-pressure control, heart-failure measures, retention, and service delivery, but the evidence base was small and heterogeneous. |
| Ryan et al., 2015 | Multiple countries affected by cyclones, floods, storms, hurricanes, tsunamis, and related disasters | Systematic review | Cardiovascular disease, diabetes, cancer, asthma, and COPD | Medication and service interruption, transport and power failure, environmental exposure, and increased physical stress. | Recommended medicine stockpiles, backup power, continuity plans, responder training, alternative access routes, and safer shelter environments. | Descriptive associations and recommendations | Weather-related disasters repeatedly exacerbated NCDs and interrupted care, supporting anticipatory supply, infrastructure, and cross-sector preparedness; evaluated interventions were limited. |
| Tonelli et al., 2016 | Global modelling for natural disasters and complex emergencies, using country-specific burden estimates | Modelling and costing study | Hypertension, cardiovascular disease, diabetes, asthma, and COPD | The Interagency Emergency Health Kit was oriented mainly toward acute conditions and omitted sustained NCD care. | Proposed an expanded 90-day kit covering acute and stable NCD treatment, with country-specific medicine quantities. | Modelled medicine requirements and costs | Including many essential NCD medicines appeared operationally and financially feasible, although the study was not a comparative cost-effectiveness or clinical-effectiveness evaluation. |
| Nakaya et al., 2017 | Japan; Great East Japan earthquake and tsunami | Observational survey study | Hypertension | Post-disaster psychological distress contributed to withdrawal from hypertension treatment. | Recommended psychological-distress screening, community outreach, and integration of mental-health support into chronic-care follow-up. | Patient-level association | Severe psychological distress was associated with approximately fourfold higher odds of withdrawing from hypertension treatment, demonstrating the importance of psychosocial continuity. |
| Man et al., 2018 | Multiple natural disasters, including hurricanes, earthquakes, tsunamis, floods, bushfires, and tornadoes | Systematic review | Cancer | Damage to oncology infrastructure and medicine stocks, patient displacement, communication failure, and treatment delays. | Described relocation and referral arrangements, emergency communication platforms, digital backups, and family-oriented hurricane action plans. | Patient/service outcomes and descriptive evidence | Natural disasters caused substantial interruptions in diagnosis and treatment; structured action plans improved family preparedness but broader effectiveness evidence remained limited. |
| Boulle et al., 2019 | Conflict and displacement settings, including Iraq, Ukraine, Syria, Lebanon, Jordan, and South Sudan | Narrative review | Diabetes | Insulin and cold-chain constraints, limited diagnostics, food insecurity, weak records, and insufficiently trained staff. | Described NCD kits, mobile clinics, point-of-care testing, simplified DKA protocols, telemedicine, and patient-held records. | Implementation/process and descriptive evidence | Adapted diabetes models were operationally feasible in several crises, supporting integration into primary care, but comparative outcome evidence was scarce. |
| Kehlenbrink et al., 2019 | Low- and middle-income humanitarian settings affected by conflict, forced migration, natural disasters, and food insecurity | Evidence synthesis/narrative review | Type 1, type 2, and gestational diabetes; cardiovascular comorbidity | High diabetes burden, poor glycaemic control, disrupted supplies, and inconsistent monitoring among displaced populations. | Reviewed structured clinics, electronic cohort monitoring, continuity of medicines, and adjustment of glycaemic targets in food-insecure settings. | Clinical, service, and descriptive evidence | Refugee and conflict-affected populations frequently had poor control and acute complications, while structured monitoring systems improved documentation and some care indicators. |
| Gyawali et al., 2021 | Global humanitarian, conflict, refugee, and COVID-19 contexts | Perspective/commentary | Major NCDs and mental ill-health | Physical NCD care and mental-health services were organized and funded in separate silos. | Recommended tiered integration of mental-health and psychosocial support, task-sharing, community support, and less fragmented financing. | Recommendation-based evidence | Integrated care was proposed to address multimorbidity and improve continuity, but no intervention-effectiveness evaluation was presented. |
| Jaung et al., 2021 | Multiple conflict, displacement, refugee, and earthquake settings | Systematic review | Hypertension and diabetes | Models were commonly physician-led and facility-based, with variable continuity, follow-up, and standardization. | Reviewed integrated primary-care clinics, mobile services, standardized pathways, cohort monitoring, patient education, and staff training. | Service/implementation outcomes and some clinical outcomes | Integrated primary care and longitudinal monitoring were recurrent components of stronger models, although outcome evidence was heterogeneous and often limited. |
| El Karak et al., 2021 | Lebanon; economic collapse, COVID-19 pandemic, and Beirut port explosion | Narrative review/commentary | Cancer | Destroyed hospitals and medicine stocks, rising costs, delayed screening, and interrupted oncology treatment. | Described medicine donations, transfer of patients and services, infrastructure restoration, and WHO–Ministry response planning. | Descriptive service evidence | Overlapping crises severely destabilized oncology care, underscoring the need for protected supply systems, referral capacity, and explicit recovery planning. |
| Ansbro et al., 2022 | Global expert perspectives across conflict, displacement, natural disasters, food insecurity, and protracted crises | Qualitative interview study | Hypertension and diabetes | Inconsistent protocols, insulin and cold-chain constraints, limited workforce skills, short-term funding, and discontinuous records. | Experts recommended integrated primary care, simplified protocols, task-sharing, patient-held records, and community education. | Qualitative implementation/process evidence | Context-adapted, phased care models were considered necessary, but the findings represent expert experience rather than comparative effectiveness evidence. |
| Kehlenbrink et al., 2022 | Low- and middle-income humanitarian settings affected by conflict, displacement, and other crises | Narrative review/perspective | Diabetes, with mental-health comorbidity | Diabetes was often excluded from humanitarian responses, and displaced populations were poorly connected to national services. | Recommended integration into primary care, differentiated delivery, resilient supply chains, information continuity, and mental-health integration. | Descriptive and recommendation-based evidence | The review framed diabetes inclusion as a health-system requirement; most supporting evidence was operational rather than comparative. |
| Odhaib et al., 2022 | Pakistan; 2005 earthquake and 2022 floods | Narrative country-experience report | Diabetes, with cardiovascular and renal-care considerations | Rapid depletion of medicines and supplies, non-standardized mobile care, and preventable diabetic-foot complications. | Described mobile clinics, an ICRC–health department diabetic-foot programme, staff training, and provision of essential supplies. | Implementation/process and descriptive evidence | Targeted workforce training and organized foot-care services were feasible during disaster response, although robust patient-outcome evaluation was limited. |
| Taylor et al., 2022 | United States; COVID-19 pandemic | Observational telemedicine programme evaluation | Hypertension with multiple cardiometabolic comorbidities | Pandemic restrictions reduced access to in-person hypertension care. | Implemented video consultations, guideline-based medication adjustment, repeated BP monitoring, and lifestyle and mental-health screening. | Patient clinical and service outcomes | Among 569 patients, telemedicine was associated with mean reductions of 9.7 mmHg in systolic and 6.8 mmHg in diastolic BP, supporting adaptive digital care. |
| Al-Nimer, 2023 | Five Arab countries affected by war, displacement, political instability, and economic crisis | Systematic review | Diabetes, with hypertension and heart disease | Medicine shortages, unaffordability, poor adherence, and fragmented clinical monitoring. | Reviewed affordable formularies, eHealth monitoring, MSF clinic models, regular consultations, laboratory follow-up, and staff training. | Descriptive, service, and implementation evidence | Multiple patient- and system-level barriers constrained diabetes care; integrated low-cost models were promising, but the evidence was heterogeneous. |
| Ide-Okochi et al., 2023 | Japan; recovery from the Kumamoto earthquake during the COVID-19 pandemic | Cross-sectional observational study | Hypertension | Treatment noncompliance associated with financial strain, reduced income, housing conditions, and poor perceived health. | Recommended long-term follow-up, financial protection, and targeted support for socially vulnerable survivors. | Patient behavioural outcomes and adjusted associations | Treatment noncompliance affected 31.2% of respondents; pandemic-related income reduction was strongly associated with noncompliance (adjusted OR 3.23). |
| Moslehi & Shirazi, 2023 | Iran; natural and human-made disasters | Qualitative study | Cardiovascular disease | Interrupted medicine access, unsuitable diets, limited services, psychological stress, and absent operational protocols. | Participants recommended advance planning, supply continuity, workforce training, referral arrangements, and family and psychosocial support. | Qualitative implementation/process evidence | Cardiovascular continuity was perceived as dependent on coordinated planning, trained personnel, and psychosocial support; no effectiveness outcomes were evaluated. |
| Pitayarangsarit et al., 2023 | Bangkok, Thailand; COVID-19 pandemic | Cross-sectional health-service assessment | Hypertension and diabetes | Workforce reassignment and reduced continuity of medicines, counselling, home visits, and routine NCD services. | Facilities reorganized clinics and used alternative delivery arrangements while attempting to preserve medicine and supply systems. | Service/system and clinical-control indicators | Hypertension services were more resilient than diabetes services; fewer than one-quarter of facilities maintained near-universal diabetes service coverage. |
| Willis et al., 2023 | Lebanon; displacement, economic collapse, COVID-19, and Beirut explosion | Qualitative patient study | Hypertension and diabetes | Medicine shortages, unaffordable care, fragmented services, and substantial patient navigation burden. | Recommended case management, shared information systems, standardized protocols, and strengthened family and social support. | Qualitative patient-experience evidence | Patient accounts showed that financial and organizational fragmentation directly undermined treatment continuity and monitoring. |
| Aebischer Perone et al., 2018 | Global humanitarian and disaster settings | Operational guideline | Multiple NCDs, including hypertension, diabetes, respiratory disease, neurological disorders, and mental illness | Field teams lacked a consistent operational approach to designing and managing NCD projects. | Recommended needs assessment, essential medicines, triage and referral, standardized tools, workforce training, patient tracking, and community-health-worker involvement. | Recommendation-based guidance | Provides a comprehensive project-cycle framework for absorptive and adaptive NCD capacity, but does not report evaluated outcomes. |
| WHO, 2024 | Eastern Mediterranean Region; humanitarian and all-hazards emergencies | Regional framework for action | Multiple NCDs | NCD care remained insufficiently embedded in national emergency governance and operational systems. | Recommended national SOPs, NCD kits, workforce training, financing, community engagement, surveillance, digital health, and designated focal points. | Recommendation-based policy guidance | Provides a transformative governance framework for regional integration of NCDs; it does not present intervention-specific effectiveness evidence. |
| Sert et al., 2024 | Türkiye and Syria; 2023 earthquakes | Qualitative interview study | Cancer | Acute trauma response displaced attention from cancer treatment, creating access, continuity, psychosocial, and future-care concerns. | Highlighted roles for cancer-patient associations, coordinated referrals, treatment-continuity planning, and psychosocial support. | Qualitative descriptive evidence | Interviews with 15 association volunteers generated three themes and 11 subthemes, indicating that oncology continuity must be explicitly incorporated into disaster response. |
| Leff et al., 2022 | Low- and middle-income countries affected by armed conflict, displacement, earthquakes, and typhoons | Review of interventions | Hypertension, cardiovascular disease, diabetes, cancer, and neuropsychiatric disorders | Few rigorously evaluated NCD interventions and persistent access and donor-financing barriers. | Reviewed free medicines, integrated outpatient services, training, telemedicine, emergency kits, patient education, and evacuation of high-risk patients. | Implementation/service outcomes and selected patient outcomes | Feasible interventions were reported across several contexts, but studies were small, heterogeneous, and rarely designed for comparative effectiveness. |
| Cooper, 2018 | Global humanitarian crises; bootcamp stakeholder report | Workshop/advocacy report | Cancer, diabetes, cardiovascular disease, and chronic respiratory disease | NCDs were frequently excluded from emergency plans, technical guidance, financing, and information systems. | Recommended national plans, global guidance, multi-month medicines, portable records, workforce training, pooled funding, partnerships, and community co-design. | Stakeholder recommendations | Presents a broad advocacy agenda and proposed transformative actions; the recommendations were not formally evaluated. |
| IFRC, 2025 | Global conflict, displacement, natural and climate-related disasters, and health-system collapse | Advocacy brief | Major NCDs and mental-health conditions | Persistent neglect, fragmented financing and coordination, disrupted supply chains, and inadequate data. | Called for resilient primary care, protected supply chains, sustainable financing, coordination, community engagement, information systems, and capacity building. | Recommendation-based evidence | Defines current advocacy priorities for humanitarian NCD care but provides no direct patient or implementation-effectiveness evaluation. |
| UN Secretary-General, 2025 | Global; COVID-19, humanitarian emergencies, displacement, and climate-related pressures | UN progress report | Broad NCD and mental-health agenda | Disrupted essential services and chronic underinvestment in NCD and mental-health systems. | Recommended integration into emergency plans, protected services and supplies, financial protection, workforce development, community care, and digital innovation. | Global descriptive and policy evidence | Identifies continuing implementation gaps and system-strengthening priorities, rather than reporting intervention-specific causal effects. |
| Pratap, 2024 | Global fragile, conflict-affected, displaced, and protracted-crisis settings | Strategic framework | Multiple NCDs and mental-health disorders | Fragmented access and inadequate continuity across preparedness, response, and recovery. | Recommended emergency kits, cash mechanisms, service mapping, patient navigation, registries, self-care support, and NCD-specific emergency protocols. | Recommendation-based framework | Offers a comprehensive continuum-of-care model with absorptive, adaptive, and transformative actions, but no effectiveness data. |
| Vijayasingham et al., 2024 | Fragile and humanitarian settings affected by displacement, conflict, economic crisis, disasters, and outbreaks | Implementation perspective/framework paper | Multiple NCDs, multimorbidity, and common mental disorders | Siloed services, fragmented records, weak supply systems, and short-term financing. | Proposed person-centred integrated models, service co-location, theory-of-change design, RE-AIM/PRISM evaluation, shared records, and pooled budgets. | Implementation and methodological evidence | Emphasizes iterative testing and implementation evaluation of integrated models; it is not a direct clinical-effectiveness study. |
| Kehlenbrink et al., 2023 | Global humanitarian settings | Narrative review | Diabetes | Diabetes exclusion was attributed to changing humanitarian paradigms, service complexity, social and cultural barriers, and inadequate financing. | Outlined opportunities for explicit inclusion, context-adapted service delivery, community and cultural adaptation, and sustainable financing. | Descriptive and recommendation-based evidence | Defines four major causes of inequity in humanitarian diabetes care and proposes corrective actions without formally evaluating their effects. |
| Cooper, 2018 | Global humanitarian settings, with examples from Dadaab, Syria, Yemen, Libya, and refugee-hosting countries | Advocacy article/report | Multiple NCDs, including diabetes, cardiovascular disease, respiratory disease, cancer, and mental illness | Emergency kits and services inadequately covered NCDs; insulin storage, medicine affordability, and service access were recurrent barriers. | Described the WHO NCD kit, clay-pot insulin storage, humanitarian pricing, mobile clinics, and civil-society advocacy. | Implementation examples and recommendations | Field adaptations demonstrate operational feasibility, although the evidence is largely descriptive rather than comparative. |
| NCD Alliance, 2025 | Global case examples, including Cox’s Bazar, Bidibidi, and Burundi | Case-study/policy article | Multiple NCDs | Undiagnosed disease, weak medicine supply, limited local capacity, and poor follow-up. | Described community screening, integration into primary care, local workforce training, reliable supplies, buddy groups, and patient self-monitoring. | Implementation examples | Case examples illustrate adaptive community-based models and continuity mechanisms, but no comparative evaluation was reported. |
| WHO, 2024 | Global all-hazards emergencies, including disasters, conflict, displacement, outbreaks, and climate events | Operational manual | Major NCDs and selected additional chronic conditions | NCDs lacked detailed, standardized integration into all-hazards preparedness and response. | Recommended risk assessment, core medicine and technology lists, buffer stocks, primary care and referral protocols, workforce preparation, information systems, and community engagement. | Recommendation-based operational guidance | Provides detailed operational standards across emergency phases; it does not present direct outcome evidence. |
| WHO, 2025 | Global all-hazards emergencies | Policy brief | Cardiovascular disease, diabetes, chronic respiratory disease, cancer, and renal failure | National policies, supplies, referral systems, and data mechanisms insufficiently incorporated NCD continuity. | Recommended national protocols, essential lists, pre-positioning and buffer stocks, referral and evacuation pathways, and information-system support. | Recommendation-based policy guidance | Translates all-hazards integration into high-level policy actions, without evaluating implementation or patient outcomes. |
| Ngaruiya et al., 2022 | Multiple conflict, refugee, earthquake, famine, and dust-storm settings | Systematic review | Cardiovascular disease, diabetes, cancer, kidney disease, asthma, and COPD | Low treatment coverage, disrupted services and supplies, psychosocial stress, and weak coordination. | Reviewed medication continuity, counselling, supply reserves, tailored services, workforce interventions, and intersectoral coordination. | Patient/service outcomes and descriptive evidence | Disasters consistently worsened chronic-disease access and outcomes, but the underlying evidence was predominantly observational and heterogeneous. |
| UNHCR, 2020 | Global conflict, displacement, and humanitarian emergencies | Operational guide | Multiple NCDs, epilepsy, and mental-health conditions | NCD care was insufficiently integrated into humanitarian planning, primary care, and routine information systems. | Recommended inclusion in humanitarian plans, pre-positioned medicines, integrated primary care, workforce training, referral systems, community support, and NCD indicators. | Recommendation-based operational guidance | Provides a practical integration framework across humanitarian programme functions; no direct intervention evaluation was reported. |
| WHO, 2016 | Global natural, technological, public-health, conflict, displacement, and food-security emergencies | Technical guidance | Multiple NCDs and selected chronic conditions | Abrupt treatment interruption and acute exacerbations create preventable morbidity and mortality during emergencies. | Recommended essential medicines, continuation of pre-emergency treatment, primary-care stabilization and referral, diagnostic access, and palliative symptom relief. | Recommendation-based technical guidance | Defines minimum immediate priorities for maintaining NCD care and absorptive capacity, but does not evaluate intervention effectiveness. |

**Abbreviations:** BP, blood pressure; COPD, chronic obstructive pulmonary disease; DKA, diabetic ketoacidosis; EMR, electronic medical record; NCD, non-communicable disease; SOP, standard operating procedure.

**Data related to each article included in the study.**

1. Article Title: Diabetes care: After the Great East Japan Earthquake

Author, Publication year: Kishimoto, 2013

The article focuses on a specific combined disaster (earthquake, tsunami, and nuclear plant crisis), but it does stratify its recommendations and insights specifically for these events, Including:

- Earthquake (Great East Japan Earthquake)
- Tsunami
- Nuclear plant disaster (Fukushima)

𝐃𝐈𝐒𝐄𝐀𝐒𝐄: TYPE 1 DIABETES

𝐃𝐑𝐔𝐆 𝐒𝐔𝐏𝐏𝐋𝐘

A. Patients with type 1 diabetes were instructed to store insulin injection kits and related supplies not only at home but also in offices or schools. This is because insulin discontinuation, especially for those with type 1 diabetes, can lead to life-threatening conditions such as diabetic ketoacidosis. The unpredictable nature of earthquakes requires that patients have access to insulin at multiple sites, as they might not be at home during the event. Insulin injections were described as indispensable for survival.

B. Emergency bags were recommended to contain insulin, antidiabetic agents, SMBG kits, glucose, and glucagon kits. These components are critical for maintaining glycemic control during the first few days when access to healthcare and pharmacy services is disrupted. Inability to monitor or adjust insulin can lead to hyperglycemia or hypoglycemia, both of which may escalate rapidly without appropriate tools.

𝐒𝐄𝐑𝐕𝐈𝐂𝐄 𝐏𝐑𝐎𝐕𝐈𝐒𝐈𝐎𝐍

C. It was recommended that patients inject insulin even without alcohol swabs or fresh needles, reuse needles when necessary, and estimate dosages based on previous experience if SMBG kits were unavailable. This is because complete avoidance of insulin injections due to lack of ideal conditions would cause worse outcomes than using less-than-ideal methods.

D. Patients were advised to inject insulin after meals instead of the usual preprandial timing to avoid hypoglycemia if meal amounts were uncertain. During disasters, meal regularity and volume are unpredictable; postprandial injection allows better matching of insulin with carbohydrate intake.

𝐒𝐔𝐏𝐏𝐎𝐑𝐓

E. Patients were urged not to internalize emotional distress and instead speak with others, as emotional stress can worsen blood glucose control. Emotional instability and anxiety during disasters were reported to have impaired diabetic management, and interpersonal support was suggested as a way to mitigate this effect.

𝐒𝐓𝐑𝐀𝐓𝐄𝐆𝐈𝐂 / 𝐎𝐏𝐄𝐑𝐀𝐓𝐈𝐎𝐍𝐀𝐋 𝐏𝐋𝐀𝐍𝐒

F. The Japan IDDM Network published a specialized manual advising patients to maintain blood glucose at a relatively higher level than normal to avoid hypoglycemia during inconsistent food supply. The manual instructed them to remain calm, adjust insulin dosing after meals, and prioritize prevention of hypoglycemia over tight control.

𝐓𝐑𝐀𝐈𝐍𝐈𝐍𝐆 𝐎𝐅 𝐅𝐎𝐑𝐂𝐄𝐒

G. Non-specialist doctors received guidance on insulin administration during disasters through manuals. Since many shelter physicians lacked diabetes specialization, structured guidance was required to prevent incorrect insulin use, dosage miscalculations, and consequent acute complications.

𝐒𝐓𝐎𝐑𝐀𝐆𝐄 𝐑𝐄𝐒𝐎𝐔𝐑𝐂𝐄𝐒

H. Insulin storage guidelines were shared by pharmaceutical companies to ensure cold-chain preservation during blackouts. Since cold storage failure could degrade insulin, immediate guidance on how to store insulin in home refrigerators without power was essential for preserving its efficacy.

𝐈𝐍𝐅𝐎𝐑𝐌𝐀𝐓𝐈𝐎𝐍 𝐃𝐈𝐒𝐒𝐄𝐌𝐈𝐍𝐀𝐓𝐈𝐎𝐍

I. Due to failure of electricity, Internet, and phone services, it was recommended to distribute printed notices via public boards at health centers. Digital communication was unreliable, and elderly patients were unfamiliar with the Internet, making analog systems the only viable solution in many contexts.

𝐃𝐈𝐒𝐄𝐀𝐒𝐄: TYPE 2 DIABETES

𝐃𝐑𝐔𝐆 𝐒𝐔𝐏𝐏𝐋𝐘

A. Patients using sulfonylureas or glinides were advised to reduce dosage to one-half or one-third in cases of decreased food intake to prevent hypoglycemia. These drugs stimulate insulin secretion regardless of food, posing significant risk during caloric restriction typical in shelters.

B. Discontinuation of biguanides, thiazolidinediones, or alpha-glucosidase inhibitors was deemed relatively safe, indicating these medications’ lower risk profiles for acute deterioration.

𝐒𝐄𝐑𝐕𝐈𝐂𝐄 𝐏𝐑𝐎𝐕𝐈𝐒𝐈𝐎𝐍

C. For patients on insulin, personalized adjustments were necessary based on their pathophysiological condition and meal availability. Uniform regimens could not be applied because of differing insulin sensitivities and baseline glycemic levels among patients.

𝐒𝐔𝐏𝐏𝐎𝐑𝐓

D. Physicians used visual medication catalogues with photographs to help patients identify lost or unreadable medication records. Many records were damaged by seawater or destroyed, making this visual aid critical for accurate re-prescription.

𝐄𝐃𝐔𝐂𝐀𝐓𝐈𝐎𝐍 / 𝐏𝐑𝐄𝐏𝐀𝐑𝐄𝐃𝐍𝐄𝐒𝐒

E. Patients were encouraged to maintain higher glucose levels than normal to avoid hypoglycemia, especially in the elderly who had decreased appetite due to grief. Strict glycemic control was discouraged, as loss of appetite made hypoglycemia a more immediate danger.

𝐒𝐓𝐑𝐀𝐓𝐄𝐆𝐈𝐂 𝐏𝐋𝐀𝐍𝐒

F. Guidelines were developed specifically for diabetes medication use in shelters, targeted especially at non-endocrinologist physicians. This ensured informed decision-making despite lack of diabetes-specific training among many first responders.

𝐍𝐔𝐓𝐑𝐈𝐓𝐈𝐎𝐍 𝐌𝐀𝐍𝐀𝐆𝐄𝐌𝐄𝐍𝐓

G. Food in shelters, such as sweet buns, rice balls, and juice, led to severe postprandial hyperglycemia. Patients had no control over meals and no option to balance carbohydrate intake. Lack of vegetables and fresh food also contributed to poor glycemic control.

𝐃𝐈𝐒𝐄𝐀𝐒𝐄: HYPERTENSION

𝐃𝐑𝐔𝐆 𝐒𝐔𝐏𝐏𝐋𝐘

A. Many hypertensive patients discontinued medication due to lost supplies, contributing to aggravated blood pressure.

𝐒𝐄𝐑𝐕𝐈𝐂𝐄 𝐏𝐑𝐎𝐕𝐈𝐒𝐈𝐎𝐍

B. Stress, sleeplessness, and anxiety in shelters were directly linked to worsened hypertension. Sleep disturbances were common due to overcrowded, noisy shelters, and stress from uncertainty about housing, income, and family safety.

𝐍𝐔𝐓𝐑𝐈𝐓𝐈𝐎𝐍 𝐌𝐀𝐍𝐀𝐆𝐄𝐌𝐄𝐍𝐓

C. High-salt food like instant noodles and salty soup exacerbated hypertension. People were encouraged to drink the soup to reduce sewage production, further increasing sodium intake. Managing salt intake was impossible in shelters without control over food preparation.

𝐃𝐈𝐒𝐄𝐀𝐒𝐄: RESPIRATORY DISEASES

𝐃𝐑𝐔𝐆 𝐒𝐔𝐏𝐏𝐋𝐘

A. Many patients experienced asthma and hay fever due to dust and allergens in shelters. Medication continuity for inhalers and antihistamines was essential to prevent acute episodes.

𝐄𝐍𝐕𝐈𝐑𝐎𝐍𝐌𝐄𝐍𝐓𝐀𝐋 𝐂𝐎𝐍𝐓𝐑𝐎𝐋

B. Dust exposure, combined with crowded shelters, triggered upper respiratory inflammation. The lack of ventilation and proximity of individuals created an environment conducive to flare-ups.

𝐈𝐍𝐅𝐄𝐂𝐓𝐈𝐎𝐍 𝐏𝐑𝐄𝐕𝐄𝐍𝐓𝐈𝐎𝐍

C. Use of masks, gargling, and handwashing were necessary but challenging in shelters with no running water. Bottled water or wet wipes substituted for hygiene in resource-poor environments.

2. Article Title: The Effectiveness of Interventions for Non-Communicable Diseases in Humanitarian Crises: A Systematic Review

Author, Publication year: Ruby, 2015

The article includes both armed conflict and natural disaster (earthquake) settings but does not stratify strategies or measures clearly by disaster type. But it has stratified those by Disease type.

- List of the NCD diseases considered in this article:

Hypertension, Heart Failure, Diabetes Mellitus, Chronic Kidney Disease, Thalassemia, Arthritis (Osteoarthritis and Rheumatoid Arthritis)

- List all of the crises and disasters considered in this article

(Only non–inside health system crises like natural disasters, conflict, displacement)

- Armed conflict (Afghanistan, Georgia, Jordan, India, etc.)
- Earthquake (Marmara region of Turkey)
- Humanitarian refugee settings (Tibetan refugees in India, Palestinian refugees in Jordan)

**Disease:**Hypertension

***SERVICE PROVISION***

**A.** A standardized hypertension treatment algorithm was implemented, which included lifestyle and dietary counseling, a graduated introduction of antihypertensive medications, periodic screening for hypertension-related complications, and associated conditions such as diabetes mellitus and hyperlipidemia. This algorithm was applied within a structured primary care environment serving Palestinian refugees in Jordan. Patients were scheduled for quarterly follow-ups to ensure adherence and continuity of care. The intervention framework was embedded within existing clinical services and structured around an Electronic Medical Record (EMR) system, allowing for structured cohort follow-up.

**B.** Among 4,130 hypertensive patients enrolled via EMRs over a 2.5-year period, 76% remained under clinical care. Of those who had their blood pressure checked, 74% had readings under the target threshold of 140/90 mmHg. In a sub-cohort of 226 patients followed for 12–15 months, 76% maintained target blood pressure levels. These results suggest a high rate of blood pressure control among patients who were actively monitored, demonstrating that clinical management protocols, when consistently applied, yielded substantial control of hypertension.

**C.** Screening for comorbid conditions revealed varied success. While cholesterol screening was nearly universal (100%), diabetes screening through glucose testing was extremely limited (3%). The disproportionate implementation of screening for associated conditions points to potential limitations in either operational scope or resource availability, despite the integrated nature of the hypertension protocol.

***USE OF INFORMATION SYSTEMS***

**A.** The Electronic Medical Record system facilitated cohort registration, clinical tracking, and outcome monitoring. It allowed for efficient retrieval of patients' longitudinal data, particularly in measuring outcomes such as blood pressure trends, medication adherence, and appointment compliance. This system enabled program managers to assess not only clinical effectiveness but also service delivery metrics like missed appointments and patient retention rates.

**B.** Data extracted from EMRs enabled stratification by risk factors and allowed for comparative analysis of adherence to target outcomes. For instance, patient retention in care after initial enrollment and their attainment of clinical targets were directly measured. This structure provided the operational backbone necessary to translate standardized guidelines into measurable health outcomes.

***TRAINING OF FORCES***

**A.** Though not explicitly disaggregated in the article, implementation of structured care protocols and EMR-based cohort monitoring presupposes the need for trained clinical personnel to execute diagnostic, therapeutic, and data management tasks. These would include nurses, primary care physicians, and IT support, all of whom must be familiar with hypertension guidelines, EMR interfaces, and referral protocols to sustain the care model described.

**Disease:** Heart Failure

***SERVICE PROVISION***

**A.** A two-year Heart Failure Disease Management Programme (HF-DMP) was instituted in Georgia within a conflict-affected general population. The intervention integrated physician training, equipment provision, patient education, and delivery of free outpatient services. Its design accounted for protracted crisis conditions and aimed to improve clinical outcomes through systematic, protocol-based care. Patient inclusion reached 400 adults with heart failure, of whom 337 completed the follow-up period.

**B.** The clinical outcomes demonstrate significant improvement attributable to the HF-DMP. Ejection fraction (EF), a cardinal marker of heart failure status, increased by 4.1 ± 2.6% (p < 0.001). Systolic blood pressure was reduced by 30.9 ± 20.0 mmHg (p < 0.001), and diastolic pressure fell by 17.8 ± 13.0 mmHg (p < 0.001). Hospital admissions decreased by 52.5% (p < 0.001), and emergency room visits were reduced by 40.7% (p < 0.001). These values indicate statistically significant improvement in patient outcomes directly tied to structured care delivery under the HF-DMP.

**C.** The program also led to shifts in functional capacity classification (NYHA). Class I and II patients increased by 13.7% and 19.2% respectively, while Class III and IV patients decreased by 26.0% and 6.8%. This pattern reflects enhanced cardiac functional status in the patient cohort. Furthermore, beta-blocker utilization increased by 73.3% (p < 0.001), reflecting adherence to pharmacologic treatment standards.

***DRUG SUPPLY***

**A.** The rise in beta-blocker usage—an essential pharmacologic intervention in heart failure management—suggests that logistical systems were sufficient to ensure medication availability throughout the conflict. This ensured continuity in pharmacotherapy, contributing directly to improved clinical markers.

***TRAINING OF FORCES***

**A.** The intervention included physician training as a core component. This training focused on clinical management of heart failure, interpretation of diagnostic parameters such as ejection fraction and blood pressure, and correct usage of pharmacological interventions. The significant changes in NYHA classification and blood pressure control further indicate that training improved provider competency in disease-specific care delivery.

***SUPPORT***

**A.** Staff salaries were supported during the intervention, ensuring stability of service provision amidst the disruptions of armed conflict. This measure prevented attrition of critical health personnel and maintained continuous access to care.

**Disease:** Diabetes Mellitus (Type 1 and Type 2)

***SERVICE PROVISION***

**A.** A standardized diabetes management algorithm was deployed across multiple clinics serving Palestinian refugees in Jordan. It comprised dietary and lifestyle counseling, graduated pharmacotherapy including insulin where needed, quarterly follow-up appointments, and structured screening for complications (e.g., nephropathy, foot problems) and comorbidities (e.g., hypertension, dyslipidemia).

**B.** Among 12,550 diabetes patients across six clinics, 78% remained in care. Screening uptake was high for cholesterol (99%), creatinine (99%), and BMI (100%), with 65% achieving PPBG targets (≤ 180 mg/dL), 63% achieving cholesterol goals (< 200 mg/dL), and 87% meeting blood pressure targets (< 140/90 mmHg). These outcomes demonstrate that structured protocols improved glycemic and cardiovascular risk factor control.

**C.** However, adherence to screening for diabetes-related complications was inconsistent. For example, only 3% of one patient subgroup had foot assessments and virtually no patients had documented ophthalmologic referrals, pointing to implementation gaps despite a structured protocol. Such gaps indicate operational weaknesses that must be addressed to ensure comprehensive diabetes management during crises.

***USE OF INFORMATION SYSTEMS***

**A.** Electronic Medical Records were central to cohort monitoring, enabling analysis of care coverage, target achievement, and risk factor distribution across large patient populations. EMR data were used to stratify patients by gender, smoking status, physical activity, and presence of complications.

**B.** Males had a significantly higher likelihood of smoking (OR M:F = 7.4; CI: 6.6–8.2; p < 0.001), being physically inactive (OR M:F = 1.8; CI: 1.6–1.9; p < 0.001), and having complications (OR M:F = 1.6; CI: 1.4–1.8; p < 0.001). Females were more likely to be obese (OR M:F = 0.34; CI: 0.32–0.37; p < 0.001). These data enabled targeted responses and informed clinical prioritization.

***TRAINING OF FORCES***

**A.** Although not quantified, the use of standardized protocols and EMRs implies the need for trained personnel to interpret clinical metrics, apply therapeutic algorithms, and manage patient follow-up. Successful implementation across multiple clinics indicates

**Disease:** Chronic Kidney Disease

***SERVICE PROVISION***

**A.** Following the Marmara earthquake in Turkey, haemodialysis (HD) services for patients with chronic kidney disease were disrupted due to structural damage to health facilities. Eight HD centres were assessed. Immediately after the earthquake, the number of dialysis sessions per week declined from 1,093 to 520, indicating a sudden drop in service delivery. One month after the disaster, services partially recovered to 616 sessions/week and further improved to 729 sessions/week by three months post-event. This reflects the necessity of maintaining functional dialysis services, even during acute infrastructural disruption, to prevent life-threatening metabolic imbalance in CKD patients reliant on dialysis.

**B.** The number of patients receiving dialysis fell from 439 pre-earthquake to 175 one week post-earthquake, 239 after one month, and 288 after three months. This reduction clearly illustrates how crisis-related service interruptions severely limit access to essential renal replacement therapy, which is non-deferrable for individuals with end-stage renal disease. Without timely restoration of these services, patients are at significant risk of fluid overload, electrolyte imbalance, and mortality.

***CREATION OF STORAGE RESOURCES***

**A.** The number of functioning HD machines declined from 95 pre-earthquake to 74 one month after the disaster, then increased to 79 by three months. The degradation in equipment availability restricted capacity to meet patient demand. The ability to return machine availability close to baseline demonstrates the importance of having reserve equipment or mobile dialysis units stored in secure, earthquake-resistant facilities, which can be rapidly deployed to mitigate the collapse of fixed-site infrastructure.

***SUPPORT***

**A.** The total HD personnel fell from 112 pre-disaster to 86 after one month, and then increased to 94 by the three-month point. The initial drop in staff availability directly contributed to the reduction in dialysis sessions and indicates that staff reallocation, attrition, or injury must be accounted for in preparedness planning. Retaining skilled dialysis personnel is critical to sustaining renal services in crisis periods.

***STRATEGIC/OPERATIONAL PLANNING***

**A.** The authors emphasized the necessity of disaster planning and patient education to reduce adverse clinical outcomes during crises. Despite the increase in once-weekly dialysis rates from 2.3% pre-earthquake to 7.2% one week after, the interdialytic weight gain of patients did not increase (2.9 ± 1.1 kg pre-earthquake versus 2.6 ± 1.1 kg post-earthquake). This finding suggests that well-informed patients likely restricted fluid intake appropriately in response to the reduced dialysis frequency, thus preventing fluid overload. Hence, strategic plans must include structured patient education on managing intake and recognizing danger signs during limited dialysis access scenarios.

**Disease:** Thalassemia

***SERVICE PROVISION***

**A.** A case series conducted in Afghanistan provided palliative surgical intervention (splenectomy) to 45 pediatric patients aged 13 months to 11 years with thalassemia. In the absence of curative options such as bone marrow transplantation, which are logistically and financially impractical in crisis settings, splenectomy was implemented to reduce transfusion frequency and mitigate complications.

**B.** Following splenectomy, mean haemoglobin levels rose from 5.4 g/dL to 8.7 g/dL, and hematocrit increased from 16.5% to 26.3%, reflecting substantial improvement in anemia severity. Additionally, the interval between blood transfusions extended from every 24 days pre-operatively to approximately every 50 days post-operatively, effectively halving transfusion dependency and conserving limited blood bank resources under crisis conditions.

***DRUG SUPPLY***

**A.** Postoperative care and transfusions continued to be necessary, albeit at reduced frequency. This shift implies a lowered burden on the pharmaceutical and transfusion infrastructure. By prolonging the interval between transfusions, surgical palliation indirectly reduces the demand for transfusion-related drugs such as chelation therapy and immunosuppressants required to manage iron overload and transfusion reactions.

***SUPPORT***

**A.** Among the cohort, two patients died pre-operatively, and three post-operative complications were observed: one case each of respiratory distress, transfusion reaction, and post-transfusion congestive heart failure. This demonstrates that although surgical palliation can be effective, it must be accompanied by access to emergency respiratory and cardiac support services to manage perioperative and transfusion-related complications in fragile pediatric patients.

***STRATEGIC/OPERATIONAL PLANNING***

**A.** The article explicitly identified that curative strategies are unfeasible in crisis contexts due to limited infrastructure and resource constraints. As a result, splenectomy was advocated as the most viable palliative strategy in such settings. Planning frameworks for NCD care in humanitarian emergencies must therefore prioritize intermediate or supportive interventions that can significantly enhance patient quality of life without requiring sophisticated tertiary care capacities.

**Disease:** Arthritis (Osteoarthritis and Rheumatoid Arthritis)

***SERVICE PROVISION***

**A.** A randomized controlled trial compared traditional Tibetan treatments with Western pharmacologic management for 28 Tibetan refugees suffering from arthritis (24 osteoarthritis, 4 rheumatoid arthritis) in northern India. Interventions lasted for three months. Traditional therapy included herbal pills, dietary restrictions, and behavioral advice, whereas the Western treatment group received NSAIDs such as ibuprofen or indomethacin.

**B.** Limb mobility improved more in the traditional treatment group, with 12 out of 14 patient pairs showing greater improvement compared to their matched counterparts. Mean improvement on the mobility scale was 1.39 points (SD 0.59) for the traditional group versus 0.57 points (SD 0.33) for the Western medicine group. This suggests that in humanitarian refugee settings where sustained access to pharmacological interventions is uncertain, traditional therapies may offer a viable, culturally acceptable alternative that still yields measurable physical benefits.

***DRUG SUPPLY***

**A.** Western pharmacologic agents (NSAIDs) demonstrated slightly superior outcomes for pain control based on visual analogue scale scores (numerical values not specified), but the lack of medication continuity in such settings and potential side effects make them less ideal as a sole intervention. The findings underscore the necessity of securing reliable drug supplies if pharmacological treatment is to be pursued for inflammatory joint conditions in displaced populations.

***SUPPORT***

**A.** The feasibility of conducting an RCT in a refugee setting using traditional and biomedical interventions confirms that even under constrained conditions, research and service delivery for arthritis are possible. This also implies that logistic support—such as community-based monitoring and patient tracking—was in place, which can serve as a model for wider implementation in similarly unstable environments.

3. Article Title: Identifying and Describing the Impact of Cyclone, Storm and Flood Related Disasters on Treatment Management, Care and Exacerbations of Non-communicable Diseases and the Implications for Public Health

Author, Publication year: Ryan, 2015

The article discusses cyclone, flood, and storm-related disasters as a combined category throughout, and does not stratify specific strategies or measures by disaster type. But the article does stratify the impact and relevant considerations by disease type

List of the NCD diseases considered in this article:

Cardiovascular diseases (including coronary heart disease, stroke, hypertension), Chronic respiratory diseases (including asthma, COPD), Diabetes, Cancer

- List all of the crises and disasters considered in this article

(Only non–inside health system crises like natural disasters, conflict, displacement)

Main focus (included in analysis):

Cyclones

Floods

Storms

Hurricanes

Tsunamis

Blizzards, snowstorms, ice storms

Mudslides

Typhoons

Windstorms

Tornadoes

**Disease:** CARDIOVASCULAR DISEASES *(including coronary heart disease, stroke, hypertension)*

***Area: Drug Supply***

**A. Ensure continued access to medication, care and medical supplies to prevent exacerbations, complications (e.g., hypertension, myocardial infarction), and death**
Following cyclone, flood, and storm-related disasters, individuals with cardiovascular diseases experience interruptions in access to prescribed medications and cardiovascular treatment regimens due to damaged transport routes, health service disruption, and loss of medical infrastructure. The interruption of antihypertensive medications and anticoagulants places patients at elevated risk of blood pressure instability and thromboembolic events. These disruptions may persist for extended periods post-disaster, as observed in Hurricane Katrina, where cardiovascular complications were documented years after the event.

***Area: Service Provision***

**B. Promote adherence and continuity of medical regimens to counter medical noncompliance during and after disasters**
Disasters result in structural breakdowns in service delivery systems and cause displacement, leading to patients' inability to attend follow-up appointments or refill prescriptions. This noncompliance is not primarily behavioral but structurally induced, as patients cannot access healthcare providers or facilities. The lack of ongoing monitoring and treatment intensifies risks of acute cardiovascular events. The absence of service continuity contributes to higher rates of myocardial infarction and cerebrovascular events in the aftermath.

**C. Reduce physical workload burdens on affected individuals (e.g., cleanup and reconstruction) to minimize cardiovascular strain**
Post-disaster environments necessitate increased physical exertion from affected populations, including cleanup of debris, relocation activities, and infrastructure repairs. For individuals with existing cardiovascular conditions, these tasks significantly elevate stress and exertional demand, leading to myocardial oxygen imbalance and subsequent ischemic events or strokes. This was especially evident following Hurricane Katrina, where increased physical workload was associated with elevated cardiovascular morbidity and mortality.

***Area: Preparation of Strategic or Operational Plans***

**D. Integrate cardiovascular care needs into strategic and operational disaster plans in alignment with Sendai Framework guidance**
The article emphasizes the necessity of mainstreaming cardiovascular disease management into disaster preparedness frameworks. This includes incorporating NCD-specific protocols into emergency management strategies and ensuring that evacuation, sheltering, and medical response systems account for chronic disease management, particularly cardiovascular monitoring and emergency resupply. This preparedness would reduce preventable deaths from delayed treatment and prevent system-wide cardiovascular care collapses.

***Area: Training of Forces***

**E. Provide targeted training for disaster response teams on cardiovascular vulnerabilities and symptom recognition**
Disaster response personnel are often unprepared to identify and manage patients with underlying cardiovascular conditions in emergency settings. Training programs should focus on recognizing signs of hypertension crisis, myocardial infarction, and stroke, especially in shelters and field care environments. This training would facilitate early intervention, triage, and referral, minimizing complications during the critical early phase post-disaster.

***Area: Creation of Storage Resources***

**F. Secure emergency power sources and ensure alternative care access routes are planned**
Power outages directly affect access to facilities with cardiovascular care capabilities (e.g., defibrillators, ECG machines). Road damage obstructs emergency service access. Storing generators and fuel at strategic locations ensures power-dependent monitoring and care equipment can be operated without interruption. Pre-mapped alternate transport routes allow timely access to tertiary care centers for patients experiencing cardiovascular emergencies.

***Area: Provision of Budget***

**G. Allocate specific budget lines in disaster preparedness plans for cardiovascular medication stockpiling**
Adequate funding for the procurement and storage of essential cardiovascular medications (e.g., beta-blockers, statins, antiplatelet agents) must be included in national and local disaster management budgets. This measure is necessary to prevent critical shortages when regular supply chains are disrupted. Strategic budget allocation ensures continuity of therapy and reduces acute cardiovascular event incidence during disasters.

**Disease:** CHRONIC RESPIRATORY DISEASES *(including asthma, COPD)*

***Area: Drug Supply***

**A. Stockpile critical respiratory medications, oxygen, and nebulization equipment in disaster-prone zones**
Individuals with asthma and COPD experience acute exacerbations due to a lack of access to bronchodilators and corticosteroids. In many disasters, the absence of stockpiled medications has led to increased physician visits and hospital admissions. Additionally, loss of respiratory equipment results in increased morbidity and risk of death, particularly for oxygen-dependent individuals.

***Area: Service Provision***

**B. Ensure access to electricity for life-sustaining equipment like nebulizers and oxygen machines**
Power outages disrupt use of oxygen concentrators and nebulizers. Patients reliant on such devices face immediate health deterioration. During Hurricane Katrina, mortality and morbidity increased significantly among oxygen-dependent individuals. Provision of uninterrupted power supply in shelters and field hospitals is critical to preventing respiratory failure.

***Area: Support***

**C. Improve shelter conditions to minimize overcrowding, mold, and allergen exposure that increase acute exacerbation risks**
Post-disaster shelters frequently lack ventilation and become breeding grounds for mold and allergens, which are critical triggers for respiratory exacerbations. The article highlights a marked increase in hospital admissions for asthma attacks and COPD exacerbations attributed to poor indoor air quality, particularly following flooding events. Dehumidification and air filtration measures should be standard in shelters housing respiratory patients.

***Area: Preparation of Strategic or Operational Plans***

**D. Include chronic respiratory disease care in strategic disaster response plans, especially in shelter planning and environmental control**
The inclusion of respiratory care in disaster planning allows for the procurement and rapid deployment of respiratory equipment, medication, and air-quality control systems. The Sendai Framework and WHO NCD policies emphasize planning for environmental hazards contributing to respiratory distress.

***Area: Training of Forces***

**E. Train response personnel to identify respiratory distress and operate emergency respiratory equipment**
Untrained staff may overlook early signs of respiratory distress or may be unable to operate oxygen delivery systems. Proper training enables timely intervention, reducing the risk of respiratory arrest and hospitalization. This is particularly critical in mass evacuation and sheltering scenarios.

***Area: Improvement of Inter-sectoral Communications***

**F. Integrate inter-sectoral communication between housing, energy, and health sectors to ensure continuity of care**
Ensuring coordination between health services, energy providers, and shelter management allows timely power restoration for respiratory equipment and air quality management. This integration reduces acute events stemming from environmental triggers or equipment failure.

**Disease:** DIABETES

***Area: Drug Supply***

**A. Maintain insulin cold chain through backup power and refrigeration access in shelters and health centers**
Loss of power leads to insulin degradation, rendering it ineffective. Diabetics dependent on insulin are most vulnerable to ketoacidosis and death. Emergency cold chain preservation systems, including refrigeration units and portable coolers with long-lasting ice packs, must be part of preparedness infrastructure.

***Area: Service Provision***

**B. Facilitate continuity of treatment, including access to prescriptions, supplies (glucose monitors), and dietary needs**
Post-disaster disruption in treatment access causes loss of blood glucose control, leading to hyperglycemic crises. Lack of dietary management (due to scarcity of appropriate food) exacerbates this risk. Shelters often distribute high-glycemic food, increasing complications. Ensuring continuity in diabetic-specific food and supplies directly reduces morbidity.

**C. Provide targeted support for insulin-dependent individuals, the group at highest risk of death and severe complications such as diabetic ketoacidosis**
The article identifies insulin-dependent diabetics as the highest-risk group. These individuals are unable to self-manage without insulin, glucose monitoring, and routine meals. Specialized interventions, including rapid resupply of insulin and individualized care at shelters, are essential to prevent complications.

***Area: Preparation of Strategic or Operational Plans***

**D. Incorporate diabetes-specific provisions in all operational disaster preparedness and health service continuity plans**
The inclusion of diabetes in strategic plans ensures pre-positioning of insulin and glucose-monitoring kits, tailored evacuation protocols, and identification of vulnerable individuals for early assistance.

***Area: Support***

**E. Educate patients pre-disaster on emergency diabetic self-management and disaster kits**
Empowering patients with self-care knowledge, including how to store insulin without refrigeration and how to adjust dosages based on meal availability and activity, mitigates disaster-related glycemic excursions.

***Area: Provision of Budget***

**F. Ensure inter-sectoral budget and logistics support for diabetic supply chains**
Allocating funds for insulin procurement, cold-chain management, and diabetic dietary provisions ensures that logistical breakdowns do not translate into fatal outcomes for this population.

**Disease:** CANCER

***Area: Service Provision***

**A. Maintain access to transport, oncology clinics, hospitals, and pharmaceutical services to prevent premature deaths**
Disruption in access to radiotherapy and chemotherapy delays treatment courses. In Hurricane Katrina, patients experienced up to one year delay in cancer treatment, contributing to poorer prognosis and premature mortality. Unlike other NCDs, cancer patients may not show immediate deterioration, but long-term survival is jeopardized by treatment interruptions.

***Area: Preparation of Strategic or Operational Plans***

**B. Develop long-term strategic plans for the restoration of oncology services post-disaster**
Cancer care services often require specialized equipment and professionals. Rapid restoration of such services is rarely prioritized. Disaster recovery plans must include mechanisms for quickly reestablishing oncology infrastructure, including temporary specialist deployment and mobile treatment units.

***Area: Drug Supply***

**C. Protect access to specialists and continuity of radiological and chemotherapeutic treatments**
Chemotherapeutic agents are time-sensitive and often require continuous administration. Disruption leads to tumor progression or recurrence. Ensuring procurement, delivery, and secure storage of these agents post-disaster is essential to prevent therapeutic failure.

***Area: Support***

**D. Include cancer patients in national and local disaster vulnerability mapping to prioritize care restoration**
By identifying cancer patients in advance, planners can prioritize their evacuation, continuity of care, and access to medication. This targeted approach helps mitigate fatal treatment interruptions.

***Area: Creation of Storage Resources***

**E. Ensure storage and rapid distribution systems for high-cost or time-sensitive oncology drugs**
Many cancer medications require stringent storage conditions. Establishing regional storage facilities with appropriate environmental controls allows for continuity of complex chemotherapy regimens even when main supply chains are disrupted.

4. Article Title: Modifying the Interagency Emergency Health Kit to include treatment for non-communicable diseases in natural disasters and complex emergencies

Author, Publication year: Tonelli, 2016

While the article lists several NCDs and outlines medications needed, it presents the intervention measures in combined scenarios (scenario 1 and scenario 2) without tailoring strategies per individual disease/crisis type.

- List of the NCD diseases considered in this article:

**Hypertension, Heart Failure, Coronary Disease, Cerebrovascular Disease, Diabetes Mellitus, Asthma, Chronic Obstructive Pulmonary Disease**

- List all of the crises and disasters considered in this article

(Only non–inside health system crises like natural disasters, conflict, displacement)

Earthquakes (e.g., 2015 Nepal earthquake, 1981 Athens earthquake, 2011 Japan earthquake/tsunami),Armed conflicts (e.g., conflicts in Syria, Ukraine), Humanitarian emergencies, Epidemics (e.g., Ebola in West Africa), Famines, Floods, Severe meteorological events

***𝐃𝐢𝐬𝐞𝐚𝐬𝐞:*** *𝐇𝐲𝐩𝐞𝐫𝐭𝐞𝐧𝐬𝐢𝐨𝐧*

***𝐃𝐫𝐮𝐠 𝐒𝐮𝐩𝐩𝐥𝐲***

A. Amlodipine and hydrochlorothiazide were explicitly listed among medications required for the management of both hypertensive urgencies/emergencies and chronic stable hypertension. In **Scenario 1**, treatment for hypertensive urgency or emergency was addressed with the inclusion of **amlodipine** (90 tablets/user) and **hydralazine** for acute blood pressure reduction. In **Scenario 2**, chronic management of stable hypertension necessitated inclusion of both **amlodipine** and **hydrochlorothiazide** (90 tablets/user). This stratification ensured that both life-threatening episodes and ongoing disease management were covered.

B. The quantity of medications was derived from estimated 90-day needs for a population of 10,000 individuals, adjusted by country-specific prevalence. For example, in Ukraine, **3484 adults per 10,000** were estimated to have hypertension in chronic form. Thus, in Scenario 2, total units required were **25,650 tablets of amlodipine** and **36,720 tablets of hydrochlorothiazide**, respectively, reflecting high burden and need for adequate supply projection.

***𝐒𝐞𝐫𝐯𝐢𝐜𝐞 𝐏𝐫𝐨𝐯𝐢𝐬𝐢𝐨𝐧***

A. The article identified the need to provide care for both hypertensive urgency/emergency and stable disease. In Scenario 1, hypertensive crises such as urgencies/emergencies were managed as acute conditions; in Scenario 2, stable hypertension was added to the management scope. This dual structure aimed to minimize immediate mortality risk while providing baseline control to reduce long-term complications such as stroke or myocardial infarction during emergencies.

***𝐒𝐭𝐫𝐚𝐭𝐞𝐠𝐢𝐜/𝐎𝐩𝐞𝐫𝐚𝐭𝐢𝐨𝐧𝐚𝐥 𝐏𝐥𝐚𝐧𝐬***

A. Inclusion of hypertension in Scenario 2 required expansion of the IEHK framework beyond acute care. The article emphasized that inclusion of chronic NCDs like hypertension is ethically justifiable due to its high prevalence and treatability, despite potential limitations in space and cost. The projected cost of antihypertensives was considered low relative to total kit cost: **amlodipine at $0.006 per tablet**, **hydrochlorothiazide at $0.0026 per tablet**, totaling under $160 for all users.

***𝐏𝐫𝐞𝐩𝐚𝐫𝐞𝐝𝐧𝐞𝐬𝐬 𝐚𝐧𝐝 𝐂𝐨𝐬𝐭 𝐄𝐬𝐭𝐢𝐦𝐚𝐭𝐢𝐨𝐧***

A. Preparedness was based on a 90-day medication course per patient. This duration aligns with the deployment timeframe of the IEHK and accommodates the critical post-disaster stabilization period. Total estimated cost for hypertension-related medications contributed modestly to the overall cost of Scenario 2 (US$22,208), showing financial feasibility for inclusion.

***𝐃𝐢𝐬𝐞𝐚𝐬𝐞:*** *𝐇𝐞𝐚𝐫𝐭 𝐅𝐚𝐢𝐥𝐮𝐫𝐞*

***𝐃𝐫𝐮𝐠 𝐒𝐮𝐩𝐩𝐥𝐲***

A. Medications proposed for acute and chronic management of heart failure included **furosemide (360 tablets/user)** and **enalapril (180–360 tablets/user)**. Furosemide served as a loop diuretic for fluid overload in acute settings, while enalapril was necessary for both acute stabilization and chronic neurohormonal blockade. These were selected based on WHO Essential Medicines List and WHO PEN protocols.

B. The IEHK drug list included furosemide at **$0.0022/tablet** and enalapril at **$0.0329/tablet**, resulting in total cost for median incidence settings of **10,440 tablets of furosemide** and **130,590 tablets of enalapril** in Scenario 2, indicating a significant yet manageable inclusion for chronic and acute heart failure management.

***𝐒𝐞𝐫𝐯𝐢𝐜𝐞 𝐏𝐫𝐨𝐯𝐢𝐬𝐢𝐨𝐧***

A. The article emphasized management of **acute heart failure** presentations and short-term follow-up after decompensation. Scenario 1 provided drugs for such acute cases; Scenario 2 extended this to patients with stable chronic heart failure to prevent future exacerbations.

B. The management of **follow-up care** was modeled as “90 days post-acute care,” acknowledging that even after initial stabilization, patients remain at elevated risk for readmission or mortality. Thus, consistent availability of medications during this period was deemed crucial.

***𝐏𝐫𝐞𝐩𝐚𝐫𝐞𝐝𝐧𝐞𝐬𝐬 𝐚𝐧𝐝 𝐂𝐨𝐬𝐭 𝐄𝐬𝐭𝐢𝐦𝐚𝐭𝐢𝐨𝐧***

A. The median prevalence of chronic heart failure across countries like Ukraine (42 per 10,000 adults) necessitated tailored projections of medication needs. Preparedness strategy incorporated not only incidence data but also assumptions of increased acute presentation due to disruption in usual care.

(no specific p-value/OR/CI reported)

*𝐃𝐢𝐬𝐞𝐚𝐬𝐞: 𝐂𝐨𝐫𝐨𝐧𝐚𝐫𝐲 𝐃𝐢𝐬𝐞𝐚𝐬𝐞*

***𝐃𝐫𝐮𝐠 𝐒𝐮𝐩𝐩𝐥𝐲***

A. Acute coronary syndrome was addressed using **aspirin (81 mg, 90 tablets/user)**, **heparin (50,000 U, 2 phials/user)**, and **glyceryl trinitrate (0.3 mg, 15 tablets/user)** in Scenario 1. Scenario 2 extended care with **atenolol** and **simvastatin** for long-term secondary prevention post-MI.

B. The article emphasized these choices based on WHO guidelines and their impact on early morbidity and mortality. Statins and beta-blockers were particularly included for their known effect in reducing post-MI events. Statin therapy with simvastatin was costed at **$0.0168/tablet** with 90 tablets/user.

***𝐒𝐞𝐫𝐯𝐢𝐜𝐞 𝐏𝐫𝐨𝐯𝐢𝐬𝐢𝐨𝐧***

A. Scenario 1 focused on emergency treatment during acute myocardial infarction, while Scenario 2 provided for ongoing treatment of stable coronary artery disease. Inclusion of chronic treatment was based on data suggesting **early statin initiation improves cardiovascular outcomes at 30 days post-MI**, although exact p-values were not reported.

***𝐒𝐭𝐫𝐚𝐭𝐞𝐠𝐢𝐜/𝐎𝐩𝐞𝐫𝐚𝐭𝐢𝐨𝐧𝐚𝐥 𝐏𝐥𝐚𝐧𝐬***

A. The authors proposed extending IEHK capacity through Scenario 2 to include preventive cardiology in emergencies, given the high baseline prevalence of coronary disease in conflict and disaster-prone regions.

***𝐏𝐫𝐞𝐩𝐚𝐫𝐞𝐝𝐧𝐞𝐬𝐬 𝐚𝐧𝐝 𝐂𝐨𝐬𝐭 𝐄𝐬𝐭𝐢𝐦𝐚𝐭𝐢𝐨𝐧***

A. Total required units in Scenario 2 included **23,040 simvastatin tablets** and **44,145 atenolol tablets**, supporting the argument that cardiovascular secondary prevention is feasible and scalable even in emergencies.

(no specific p-value/OR/CI reported)

*𝐃𝐢𝐬𝐞𝐚𝐬𝐞: 𝐂𝐞𝐫𝐞𝐛𝐫𝐨𝐯𝐚𝐬𝐜𝐮𝐥𝐚𝐫 𝐃𝐢𝐬𝐞𝐚𝐬𝐞*

***𝐃𝐫𝐮𝐠 𝐒𝐮𝐩𝐩𝐥𝐲***

A. While no stroke-specific acute thrombolytics were included due to complexity, the IEHK included antihypertensives (e.g., **amlodipine, enalapril**) and **aspirin** to address acute and post-stroke blood pressure control and antiplatelet therapy. These were used to stabilize patients post-stroke and prevent recurrence.

B. The rationale provided was that **follow-up care of acute stroke survivors** required resources already present in the kit (aspirin for secondary prevention, blood pressure control), covering a minimum 90-day need per individual.

***𝐒𝐞𝐫𝐯𝐢𝐜𝐞 𝐏𝐫𝐨𝐯𝐢𝐬𝐢𝐨𝐧***

A. The article addressed **post-acute stroke management** explicitly and emphasized the high risk of recurrence if antihypertensive and antiplatelet therapy is not initiated or continued during disaster disruptions. Hence, Scenario 2 supports identification and ongoing care of people with history of cerebrovascular events.

***𝐒𝐭𝐫𝐚𝐭𝐞𝐠𝐢𝐜/𝐎𝐩𝐞𝐫𝐚𝐭𝐢𝐨𝐧𝐚𝐥 𝐏𝐥𝐚𝐧𝐬***

A. The strategy involved allocation of medications not only for acute stroke events but also for **secondary prevention**, including individuals with a “remote prior stroke” who might otherwise be neglected in acute-focused disaster responses.

***𝐏𝐫𝐞𝐩𝐚𝐫𝐞𝐝𝐧𝐞𝐬𝐬 𝐚𝐧𝐝 𝐂𝐨𝐬𝐭 𝐄𝐬𝐭𝐢𝐦𝐚𝐭𝐢𝐨𝐧***

A. Cost estimates for stroke-related medications were not separated, but aspirin and antihypertensives were common to both cerebrovascular and cardiovascular care, streamlining procurement and inventory.

**𝐃𝐢𝐬𝐞𝐚𝐬𝐞**: 𝐃𝐢𝐚𝐛𝐞𝐭𝐞𝐬 𝐌𝐞𝐥𝐥𝐢𝐭𝐮𝐬

𝐃𝐫𝐮𝐠 𝐒𝐮𝐩𝐩𝐥𝐲

A. The article included **insulin** (10 mL phials, 100 U/mL) for the management of **severe hyperglycaemia** and **dextrose 50% ampoules** for **severe hypoglycaemia**. Insulin was required in **Scenario 1** for emergency management of life-threatening acute hyperglycaemia. For children, it was assumed that **2 insulin phials per child** would be needed over the 90-day period, based on an average requirement of **20 units/day**. Adults with severe hyperglycaemia were also included in Scenario 1 with an assumed requirement of **60 units/day**.

B. In **Scenario 2**, chronic management of diabetes was addressed through the inclusion of **metformin (270 tablets per user)** and **glibenclamide (90 tablets per user)**. These oral agents were chosen to avoid the logistical limitations of cold chain storage required by insulin. The article explicitly stated that inclusion of insulin for adult chronic management in Scenario 2 was **not feasible** due to space and temperature control constraints of the Interagency Emergency Health Kit.

C. The prevalence of stable diabetes was calculated using regional and country-specific data. For example, in Jordan, the article estimated **1,062 adults and 45 children with diabetes per 10,000 population**, driving the allocation of medications. In total, for Scenario 2 in median-incidence settings, **112,455 tablets of metformin** and **13,000 tablets of glibenclamide** were projected.

***𝐒𝐞𝐫𝐯𝐢𝐜𝐞 𝐏𝐫𝐨𝐯𝐢𝐬𝐢𝐨𝐧***

A. Service provision in **Scenario 1** focused on identifying and treating acute presentations such as **hyperglycaemic crisis** or **hypoglycaemia**. The approach assumed that all patients with acute diabetes-related emergencies would receive immediate intervention with insulin or dextrose, respectively. This immediate response was justified on the basis that untreated diabetic emergencies can result in rapid mortality.

B. In **Scenario 2**, the strategy extended to patients presenting with **stable diabetes** who required continued care during disasters to avoid deterioration into emergency states. This included patients who might present for unrelated trauma or infectious disease but be incidentally found to have untreated diabetes requiring long-term management.

***𝐏𝐫𝐞𝐩𝐚𝐫𝐞𝐝𝐧𝐞𝐬𝐬***

A. The drug supply and service strategy relied on the assumption of **a 90-day emergency response window**, the standard design scope of the IEHK. This window aligned with expected deployment duration and accounted for limitations in ongoing pharmaceutical logistics during protracted or post-disaster phases.

B. The article noted that insulin would only be included for those “in whom lack of insulin would clearly be fatal,” i.e., children and patients in hyperglycaemic crisis, not for all stable adults, due to **extremely limited cold chain space** in the IEHK.

***𝐈𝐧𝐧𝐨𝐯𝐚𝐭𝐢𝐨𝐧 𝐚𝐧𝐝 𝐅𝐮𝐭𝐮𝐫𝐞 𝐒𝐮𝐩𝐩𝐨𝐫𝐭***

A. The article discussed promising new evidence suggesting that **modern insulin formulations may remain potent even outside the cold chain**, which could eventually justify expanding insulin use in IEHK kits for adults. However, this was not yet adopted in the current model due to lack of validated implementation protocols.

***𝐃𝐢𝐬𝐞𝐚𝐬𝐞:*** *𝐀𝐬𝐭𝐡𝐦𝐚*

***𝐃𝐫𝐮𝐠 𝐒𝐮𝐩𝐩𝐥𝐲***

A. For acute exacerbations, the article prescribed **salbutamol (200-dose inhaler)** and **oral prednisone (10 mg tablets)** as essential interventions. Salbutamol was administered **twice per day** for asthma attacks, while **prednisone** was provided at dosages of **3 tablets/day for 5 days in adults** and **3 tablets/day for 2 days in children**. These regimens were derived directly from WHO PEN treatment protocols.

B. For stable asthma management under **Scenario 2**, **inhaled beclomethasone** was included at **2 inhalers per user for 90 days**. The inhalers contained **100 doses of 100 mcg** and cost **$4.80 per inhaler**. These were intended to prevent exacerbations in patients with chronic asthma, reducing the burden on emergency services during crises.

***𝐒𝐞𝐫𝐯𝐢𝐜𝐞 𝐏𝐫𝐨𝐯𝐢𝐬𝐢𝐨𝐧***

A. **Scenario 1** addressed acute care for patients actively experiencing **asthma attacks**, which were expected to increase in frequency during emergencies due to exposure to dust, cold, stress, or unavailability of previous medications. The article cited substantial evidence that environmental and psychological stressors during crises exacerbate asthma symptoms.

B. **Scenario 2** included chronic management with corticosteroids, recognizing that without regular preventive inhalation therapy, even patients with mild asthma would likely progress to acute exacerbations during humanitarian emergencies.

***𝐏𝐫𝐞𝐩𝐚𝐫𝐞𝐝𝐧𝐞𝐬𝐬***

A. Preparedness modeling was based on country-specific prevalence data. For instance, in Bangladesh, the article projected **351 adults and 290 children per 10,000** with asthma. This justified the significant allocation of **2050 inhalers of beclomethasone** and **1525 inhalers of salbutamol** for Scenario 2 in median settings.

***𝐅𝐢𝐧𝐚𝐧𝐜𝐢𝐚𝐥 𝐀𝐧𝐚𝐥𝐲𝐬𝐢𝐬***

A. Despite high cost per unit (**$4.80 for beclomethasone**, **$3.32 for salbutamol**), the total investment in asthma care in Scenario 2 was deemed justified due to the high morbidity associated with untreated or undertreated asthma exacerbations in emergencies.

***𝐃𝐢𝐬𝐞𝐚𝐬𝐞:*** *𝐂𝐡𝐫𝐨𝐧𝐢𝐜 𝐎𝐛𝐬𝐭𝐫𝐮𝐜𝐭𝐢𝐯𝐞 𝐏𝐮𝐥𝐦𝐨𝐧𝐚𝐫𝐲 𝐃𝐢𝐬𝐞𝐚𝐬𝐞 (𝐂𝐎𝐏𝐃)*

***𝐃𝐫𝐮𝐠 𝐒𝐮𝐩𝐩𝐥𝐲***

A. The article mandated the inclusion of **salbutamol inhalers (3 per user)** and **oral prednisone (10 mg tablets, 21 per user)** for **COPD exacerbation** management in **Scenario 1**, based on a standard regimen of **6 doses/day of salbutamol** and **3 tablets/day for 7 days** of prednisone.

B. For chronic management under **Scenario 2**, **inhaled beclomethasone** was supplied similarly to asthma (2 inhalers per user), acknowledging the chronic inflammatory nature of COPD and its response to inhaled corticosteroids in stable phases.

C. In median incidence scenarios, the number of inhalers and tablets was substantial: **1532 salbutamol inhalers** and **2047 beclomethasone inhalers** were projected per kit, justifying their prominent inclusion in the revised IEHK.

***𝐒𝐞𝐫𝐯𝐢𝐜𝐞 𝐏𝐫𝐨𝐯𝐢𝐬𝐢𝐨𝐧***

A. The service plan for COPD mirrored that for asthma. Acute exacerbations were prioritized in Scenario 1 due to their strong association with increased mortality and hospital admission risk. Treatment aimed to alleviate bronchospasm and inflammation rapidly in unstable COPD patients.

B. Scenario 2 expanded care to stable patients whose condition could be controlled with inhaled corticosteroids, decreasing emergency department utilization and mortality during disasters where health infrastructure is already overburdened.

***𝐏𝐫𝐞𝐩𝐚𝐫𝐞𝐝𝐧𝐞𝐬𝐬 𝐚𝐧𝐝 𝐏𝐨𝐩𝐮𝐥𝐚𝐭𝐢𝐨𝐧 𝐁𝐮𝐫𝐝𝐞𝐧***

A. COPD burden was high in countries like Ukraine, where **513 adults per 10,000** were estimated to have stable COPD, necessitating significant drug stockpiles. These data informed projections for required quantities and validated the need for chronic as well as acute supply chains.

***𝐂𝐨𝐬𝐭 𝐂𝐨𝐧𝐬𝐢𝐝𝐞𝐫𝐚𝐭𝐢𝐨𝐧***

A. Despite high volume needs, the costs were justified given the acute nature of exacerbations and their potentially fatal outcomes in settings with limited ventilation or oxygen availability. For example, **prednisone tablets were only $0.0206 each**, making this a cost-effective intervention.

5. Article Title: Psychological Distress and the Risk of Withdrawing From Hypertension Treatment After an Earthquake Disaster

Author, Publication year: Nakaya, 2017

The article only discusses one specific disaster scenario—the Great East Japan Earthquake (GEJE). The focus is specifically on hypertension treatment. While other diseases are briefly referenced (e.g., in the context of comorbidities).

List of the NCD diseases considered in this article:

**Hypertension, Stroke, Myocardial Infarction or Angina, Cancer, Kidney Disease, Liver Disease, Diabetes Mellitus, Hyperlipidemia**

•	List all of the crises and disasters considered in this article

(Only non–inside health system crises like natural disasters, conflict, displacement)

Main focus: Great East Japan Earthquake (GEJE) and tsunami

**Disease:** Hypertension

***Screening and Assessment of Psychological Distress***

A. The article specifies that psychological distress, assessed using the Kessler-6 (K6) scale, is a significant risk factor for hypertensive patients withdrawing from their hypertension treatment (HTTx) in post-disaster settings. The study demonstrated that individuals with a higher degree of psychological distress (K6 score ≥13) had a markedly increased risk of discontinuing their HTTx, with an odds ratio (OR) of 4.0, a 95% confidence interval (CI) of 1.3-10.6, and a p-value of <0.01. The explanation for this finding is that psychological distress, induced by the disaster context, exacerbates mental health strain, leading patients to perceive hypertension treatment as a lesser priority or to develop feelings of hopelessness, thus directly undermining adherence to HTTx. The survey design embedded in the Shichigahama Health Promotion Project provided structured, systematic data collection that revealed this significant association. By using a validated psychometric tool (K6), the study ensured that the identification of psychological distress was reliable, and this distress was shown to correlate with a quadrupled likelihood of treatment discontinuation, highlighting the necessity for mental health screening as an integral component of hypertension management in disaster-affected populations.

***Provision of Mental Health Support***

A. The article emphasizes that the increased risk of withdrawal from HTTx following the Great East Japan Earthquake (GEJE) is attributable to psychological distress, which serves as a barrier to ongoing medical adherence. Thus, integrating mental health care into disaster response frameworks is essential to mitigate the risk of treatment discontinuation for hypertensive patients. The article posits that mental health support for patients with hypertension in disaster-affected areas can prevent the exacerbation of vascular diseases, as untreated hypertension, combined with psychological distress, may contribute to the development or worsening of conditions such as stroke, myocardial infarction, or angina. The data suggest that without targeted mental health interventions, patients are significantly more likely to abandon their treatment, thereby facing heightened morbidity risks. No specific p-value/OR/CI reported.

***Community-Based Health Surveys and Outreach***

A. The Shichigahama Health Promotion Project is described in the article as a structured survey and outreach initiative to evaluate the overall health and life status of residents in disaster-affected areas. For hypertensive patients, participation in these community surveys provided a mechanism to identify individuals who had withdrawn from HTTx and who may require targeted intervention. The survey methodology involved direct visits to households, engagement with residents, and the collection of consent and data, ensuring comprehensive community coverage. The article underlines that these surveys serve as a critical tool to detect treatment gaps and support patients in continuing their care post-disaster. No specific p-value/OR/CI reported.

**Disease:** Stroke

***Medical Treatment for Physical Diseases***

A. The article lists stroke as a chronic physical disease for which continued medical treatment is necessary post-disaster. The context suggests that disruptions caused by the Great East Japan Earthquake may impede access to essential care for stroke patients, and therefore, ensuring uninterrupted medical treatment is a critical priority. The importance of maintaining medical therapy is implicitly tied to the broader discussion of post-disaster management of chronic diseases, as stroke patients require consistent pharmacological management and monitoring to prevent recurrent events. No specific p-value/OR/CI reported.

**Disease:** Myocardial Infarction or Angina

***Medical Treatment for Physical Diseases***

A. The article identifies myocardial infarction and angina as conditions requiring sustained medical therapy after a disaster. This inclusion is based on the understanding that cardiovascular conditions are highly sensitive to stress and disruption of care, both of which are prevalent in disaster settings. The article implicitly underscores that patients with these conditions need access to ongoing pharmacological treatment and monitoring, as lapses in care due to disaster-related challenges (such as psychological distress or logistical barriers) can lead to adverse cardiac outcomes. This risk is accentuated by the broader finding that psychological distress is associated with increased withdrawal from treatment for chronic physical illnesses, suggesting that myocardial infarction or angina patients are similarly vulnerable to treatment discontinuation in disaster contexts. No specific p-value/OR/CI reported.

**Disease:** Cancer

***Reference to Prior Studies on Depression and Treatment Adherence***

A. The article references earlier research indicating that depression is a significant risk factor for withdrawal from cancer treatment, specifically citing findings that cancer patients experiencing depression are at higher risk of discontinuing their therapy compared to those without depression. The inclusion of this point in the article’s discussion highlights the parallel between cancer and hypertension in terms of the detrimental impact of psychological distress on treatment adherence. Although the article does not elaborate on specific operational measures for cancer care, it implies that mental health support is critical for ensuring continued cancer treatment in disaster settings, as patients with untreated depression may abandon treatment, leading to worsened cancer outcomes. No specific p-value/OR/CI reported.

**Disease:** Kidney Disease

***Medical Treatment for Physical Diseases***

A. The article explicitly identifies kidney disease as a chronic physical disease for which medical treatment must be continued in post-disaster settings. The necessity of ensuring uninterrupted medical care for kidney disease patients is framed within the broader concern that disasters, such as the Great East Japan Earthquake (GEJE), lead to increased psychological distress and disruption of care. The article suggests that failure to maintain access to medical treatment for chronic conditions, including kidney disease, may exacerbate underlying health conditions and contribute to increased morbidity. Although specific operational strategies such as drug supply logistics or clinical service pathways for kidney disease are not discussed, the article implies that kidney disease patients are at risk of care discontinuation similar to those with hypertension, where the odds ratio for treatment withdrawal in the presence of psychological distress was 4.0 (95% confidence interval 1.3-10.6, p<0.01). Thus, continued medical treatment must be prioritized to safeguard health outcomes in this vulnerable population. No specific p-value/OR/CI reported.

**Disease:** Liver Disease

***Medical Treatment for Physical Diseases***

A. The article includes liver disease in its list of chronic physical diseases requiring continued medical treatment following disasters such as the GEJE. It emphasizes that the disruption of medical care in post-disaster scenarios can lead to adverse health consequences for patients with liver disease. Although the article does not detail specific medical interventions for liver disease management, it situates liver disease within a broader framework where the maintenance of chronic disease treatment is critical to public health resilience. By analogy with the data presented for hypertension, the article suggests that psychological distress and logistical challenges in disaster contexts increase the likelihood of patients discontinuing treatment, which for liver disease could result in disease progression, decompensation, or increased morbidity. No specific p-value/OR/CI reported.

**Disease:** Diabetes Mellitus

***Medical Treatment for Physical Diseases***

A. The article identifies diabetes mellitus as one of the chronic diseases requiring uninterrupted medical treatment in post-disaster settings. The classification of diabetes mellitus alongside hypertension, kidney disease, and liver disease underscores the critical need to preserve access to medications, self-management supplies (such as glucose monitors and insulin), and clinical care for diabetic patients after disasters like the GEJE. The article’s findings on psychological distress—where hypertensive patients with K6 scores ≥13 had an odds ratio of 4.0 (95% confidence interval 1.3-10.6, p<0.01) for withdrawing from treatment—highlight the indirect but significant implication for diabetes mellitus: patients with diabetes experiencing similar psychological distress may face an elevated risk of treatment discontinuation. Therefore, the necessity of medical treatment continuation for diabetes is implied to prevent adverse outcomes such as hyperglycemia, diabetic ketoacidosis, and long-term complications exacerbated by disasters. No specific p-value/OR/CI reported.

**Disease:** Hyperlipidemia

***Medical Treatment for Physical Diseases***

A. The article identifies hyperlipidemia as a chronic physical disease for which ongoing medical treatment must be ensured in disaster-affected populations. The article’s broader argument—that psychological distress following disasters leads to an increased risk of withdrawing from chronic disease treatments—applies implicitly to hyperlipidemia management. Although specific therapeutic strategies (such as statin access or lipid panel monitoring) are not discussed, the inclusion of hyperlipidemia in the category of diseases requiring ongoing medical care emphasizes that patients with hyperlipidemia face the same vulnerability to treatment discontinuation observed in hypertensive patients, where the odds ratio for withdrawal under psychological distress was 4.0 (95% confidence interval 1.3-10.6, p<0.01). Consequently, ensuring uninterrupted medical treatment for hyperlipidemia is essential to prevent worsening lipid profiles, increased cardiovascular risk, and related morbidity.

6. Article Title: The effect of natural disasters on cancer care: a systematic review

Author, Publication year: Man, 2018

the article stratifies strategies/measures by disaster type. The article discusses specific challenges and recommendations for different natural disasters

- List of the NCD diseases considered in this article:

**Cancer (Oncology care for various types of cancer, no stratification by specific cancer type)**

- List all of the crises and disasters considered in this article

(Only non–inside health system crises like natural disasters, conflict, displacement)

Main focus (included in analysis): Hurricanes, Earthquakes, Tsunamis, Flooding, Bushfires, Tornadoes

**Crisis:** Hurricanes

***𝐃𝐑𝐔𝐆 𝐒𝐔𝐏𝐏𝐋𝐘***

**A.** Oncology drug storage rooms were flooded, causing extensive damage to chemotherapy inventories, refrigerated medications, and investigational drugs. During the hurricane, hospital staff evacuated oncology drugs, protocol books, and investigational supplies to temporary medical facilities. The evacuation was necessary to sustain life-saving treatment for cancer patients, including pediatric patients, as more than 100 were relocated to other hospitals. These emergency efforts were enacted because the drugs were at risk of being destroyed by water infiltration and power loss, which would render them ineffective or unsafe.

**B.** Pharmacies were shut down after Hurricane Katrina, limiting access to prescription refills. Cancer patients who were displaced lacked sufficient supplies, and the loss of distribution infrastructure meant that patients were at risk of treatment discontinuation. The article emphasized that some caregivers reported patients did not have access to medications even for the following 2 weeks, which contributed to psychological stress and emergency department overload.

***𝐒𝐄𝐑𝐕𝐈𝐂𝐄 𝐏𝐑𝐎𝐕𝐈𝐒𝐈𝐎𝐍***

**A.** A hospital closure post-Katrina led to a 70% reduction in patient volume, with 34% of patients displaced from their residences for over 6 months. Cancer services, including chemotherapy and radiotherapy, became inaccessible due to geographic fragmentation and service redistribution, resulting in an average 2-month delay from initial presentation to diagnosis. The widespread structural damage, flooding of linear particle accelerators, and closure of radiotherapy units meant that some patients had to be re-stimulated and re-planned to resume radiotherapy. In one case, 79 patients experienced radiotherapy delays of 7 to 14 days due to immobilization device loss and equipment failure.

**B.** Pediatric cancer inpatient care was overwhelmed, with more than 100 children relocated to other states due to the inability of local facilities to accommodate them. This resulted from damaged infrastructure, loss of pediatric oncology beds, and insufficient surge capacity in the regional system. The unpredictable nature of evacuation compounded logistical difficulties, increasing stress for families and placing additional burdens on receiving institutions.

***𝐒𝐔𝐏𝐏𝐎𝐑𝐓***

**A.** Psychological distress among caregivers of pediatric cancer patients after Hurricane Katrina led to the development of the Hurricane Action Plan. This plan was implemented in later hurricanes and found to be highly effective. In subsequent events, 92% of families indicated that the Hurricane Action Plan was extremely useful, especially in reducing psychological burden and enhancing preparedness. The support component included evacuation protocols, medication checklists, and educational resources.

***𝐏𝐑𝐄𝐏𝐀𝐑𝐀𝐓𝐈𝐎𝐍 𝐎𝐅 𝐒𝐓𝐑𝐀𝐓𝐄𝐆𝐈𝐂 𝐎𝐑 𝐎𝐏𝐄𝐑𝐀𝐓𝐈𝐎𝐍𝐀𝐋 𝐏𝐋𝐀𝐍𝐒***

**A.** The Hurricane Action Plan ensured that families prepared for cancer care during disasters. As a result of its implementation, 78% of families had evacuation plans, 71% had stored 2 weeks of medication, and 43% had treatment plans physically available during displacement. The plan reduced the chaos observed during Hurricane Katrina, where 43% of patients were evacuated without any treatment documentation, contributing to care delays.

***𝐓𝐑𝐀𝐈𝐍𝐈𝐍𝐆 𝐎𝐅 𝐅𝐎𝐑𝐂𝐄𝐒***

**A.** Oncology trainee programs were disrupted by workforce relocation. Trainees were transferred to hospitals where their training opportunities were diminished due to a lower patient volume and an emphasis on immediate care provision. Administrative challenges, credentialing delays, and reduced access to diverse case exposure hindered the training process, threatening accreditation compliance.

***𝐂𝐑𝐄𝐀𝐓𝐈𝐎𝐍 𝐎𝐅 𝐒𝐓𝐎𝐑𝐀𝐆𝐄 𝐑𝐄𝐒𝐎𝐔𝐑𝐂𝐄𝐒***

**A.** Biobanks and research specimen storage units were affected. Flooding destroyed pathology samples, chemotherapy protocols, and radiation plans, impeding both research continuity and patient care. Without digital backups, the loss of physical samples made it impossible to resume treatments as planned for certain patients, especially those in ongoing trials.

***𝐈𝐌𝐏𝐑𝐎𝐕𝐄𝐌𝐄𝐍𝐓 𝐎𝐅 𝐈𝐍𝐓𝐄𝐑-𝐒𝐄𝐂𝐓𝐎𝐑𝐀𝐋 𝐂𝐎𝐌𝐌𝐔𝐍𝐈𝐂𝐀𝐓𝐈𝐎𝐍𝐒***

**A.** Communication system failure was a major barrier. Phone lines and internet systems collapsed, hindering coordination between displaced patients and providers. As a solution, oncology organizations created online platforms for emergency communication and facilitated oncologist-to-oncologist handovers, which were critical for continuing care when patients arrived at unfamiliar facilities.

**Crisis:** Earthquakes / Tsunamis

***𝐃𝐑𝐔𝐆 𝐒𝐔𝐏𝐏𝐋𝐘***

**A.** In the Gorkha earthquake (Nepal), nationwide drug shortages were reported. Hospitals could not replenish stock due to infrastructure collapse, and the healthcare system’s already fragile logistics were fully overwhelmed. Patients requiring ongoing cancer therapies were unable to receive essential medications such as sorafenib or hormonal agents.

***𝐒𝐄𝐑𝐕𝐈𝐂𝐄 𝐏𝐑𝐎𝐕𝐈𝐒𝐈𝐎𝐍***

**A.** The Great East Japan Earthquake caused severe infrastructure collapse: 10 out of 380 hospitals (3%) were completely destroyed and 290 (76%) severely damaged. Oncology services were suspended across multiple districts. Damage to radiation therapy infrastructure, inpatient facilities, and loss of electronic and paper records made it impossible to continue standard cancer care.

**B.** 75 oncology patients were forced to evacuate a structurally compromised hospital. Of these, 48 were transferred to other institutions, and 23 were sent home despite unsafe housing conditions. Transportation was arranged ad hoc, largely dependent on individual physician networks, as there was no central coordination system tailored for oncology patients.

***𝐒𝐔𝐏𝐏𝐎𝐑𝐓***

**A.** Patients undergoing cancer treatment during the earthquake scored high on the Kessler Psychological Distress Scale. Specifically, 9 of 94 patients (9.6%) had scores greater than 13, indicating clinical distress (OR 1.8, 95% CI 0.8–3.5, p=0.14).

**B.** Spouses of cancer patients also experienced mental health deterioration. Female spouses had significantly higher psychological distress (OR 1.6, p=0.02), whereas male partners did not (OR 1.0, p=0.92). This underscores the importance of psychological support not just for patients, but also for their caregivers.

***𝐏𝐑𝐄𝐏𝐀𝐑𝐀𝐓𝐈𝐎𝐍 𝐎𝐅 𝐒𝐓𝐑𝐀𝐓𝐄𝐆𝐈𝐂 𝐎𝐑 𝐎𝐏𝐄𝐑𝐀𝐓𝐈𝐎𝐍𝐀𝐋 𝐏𝐋𝐀𝐍𝐒***

**A.** The introduction of a disaster victim certificate improved healthcare access. Patients holding the certificate had a longer median survival (18.3 months vs 12.6 months), although results were not statistically significant (HR 0.47; 95% CI 0.21–1.05; p=0.07). The certificate facilitated streamlined registration and prioritization in overwhelmed systems.

***𝐓𝐑𝐀𝐈𝐍𝐈𝐍𝐆 𝐎𝐅 𝐅𝐎𝐑𝐂𝐄𝐒***

**A.** Due to staff shortages post-earthquake, non-specialist nurses were assigned to oncology and palliative care roles. Lacking oncology training, these nurses faced difficulty administering pain medications or managing immunocompromised patients. However, many continued to provide bedside palliative support, driven by moral obligation and absence of alternatives.

***𝐂𝐑𝐄𝐀𝐓𝐈𝐎𝐍 𝐎𝐅 𝐒𝐓𝐎𝐑𝐀𝐆𝐄 𝐑𝐄𝐒𝐎𝐔𝐑𝐂𝐄𝐒***

**A.** Biobanks and clinical specimen repositories lost functionality during tsunamis and earthquakes. In one hospital, refrigeration units failed, and there were no backup generators. Pathological samples essential for staging and treatment planning were destroyed, disrupting care for patients who had already initiated treatment.

***𝐈𝐌𝐏𝐑𝐎𝐕𝐄𝐌𝐄𝐍𝐓 𝐎𝐅 𝐈𝐍𝐓𝐄𝐑-𝐒𝐄𝐂𝐓𝐎𝐑𝐀𝐋 𝐂𝐎𝐌𝐌𝐔𝐍𝐈𝐂𝐀𝐓𝐈𝐎𝐍𝐒***

**A.** With hospital systems offline, physicians used Twitter and local TV to broadcast information. Leaflets were distributed in shelters, detailing treatment instructions, emergency contacts, and service locations. These were critical to prevent complete discontinuity in care for displaced oncology patients.

***𝐏𝐑𝐎𝐕𝐈𝐒𝐈𝐎𝐍 𝐎𝐅 𝐁𝐔𝐃𝐆𝐄𝐓***

**A.** Many oncology staff lost jobs, retired early, or were unable to re-establish practices. In the most damaged prefectures, financial strain compounded service loss, as both public and private oncology services became unviable due to building damage and reduced patient access.

**Crisis:** Flooding

***𝐃𝐑𝐔𝐆 𝐒𝐔𝐏𝐏𝐋𝐘***

**A.** In Thailand, flooding led to the shutdown of 20 tertiary oncology centers, representing 20% of national cancer care capacity and impacting more than 40,000 patients. Drug supply chains were halted, affecting both chemotherapy and radiopharmaceutical delivery.

**B.** Oncology medication stores, including refrigerated drugs and biologics, were water-damaged. Emergency teams salvaged usable drugs and transported them to functioning hospitals. In one case, protocols and refrigerated agents were rescued within a short window to prevent irreversible loss.

***𝐒𝐄𝐑𝐕𝐈𝐂𝐄 𝐏𝐑𝐎𝐕𝐈𝐒𝐈𝐎𝐍***

**A.** In a study from China, patients in flooded villages had significantly elevated cancer mortality (Standard Mortality Rate 127.3 vs 74.81, p<0.05). Years of Potential Life Lost were also higher (14.39% vs 7.01%, p<0.05), particularly among men aged 30–45 years.

**B.** Radiotherapy services were disrupted. Flooded radiation oncology departments relocated patients to satellite centers 20–30 miles away. This logistical adaptation ensured continuity but increased travel burden and risk of delayed treatment initiation.

***𝐂𝐑𝐄𝐀𝐓𝐈𝐎𝐍 𝐎𝐅 𝐒𝐓𝐎𝐑𝐀𝐆𝐄 𝐑𝐄𝐒𝐎𝐔𝐑𝐂𝐄𝐒***

**A.** A major biobank storing 57,000 oncology tissue samples suffered nitrogen tank flooding. Restoration involved freeze-drying samples, reorganizing catalogues, retesting for viability, and documenting recovery. The process took over 3 years and involved high financial and logistical burden.

***𝐒𝐔𝐏𝐏𝐎𝐑𝐓***

**A.** A cross-sectional study found 9% of general patients and 23% of evacuees had interruptions in medication, with elderly patients over 75 years showing more deterioration in health 1 month after the flood event.

**Crisis:** Bushfires

***𝐒𝐞𝐫𝐯𝐢𝐜𝐞 𝐏𝐫𝐨𝐯𝐢𝐬𝐢𝐨𝐧***

**A.** In a study conducted following a bushfire event involving 520 individuals, researchers assessed the 12-month post-disaster prevalence of cancer. The findings showed no significant increase in cancer incidence between pre- and post-bushfire populations (0.85% vs 1.25%; risk ratio 1.06). This outcome was used to determine that bushfires, within this observational frame, did not significantly alter cancer prevalence. However, while service disruption was not quantified, the study emphasized a 283% increase in mental illness within the same group, implying that while cancer services might not have been overwhelmed, the broader health system experienced psychological strain.

**Crisis:** Tornadoes

***𝐃𝐫𝐮𝐠 𝐒𝐮𝐩𝐩𝐥𝐲***

**A.** During the 2011 tornado that impacted St. John’s Regional Medical Center in Joplin, USA, radiation oncology departments faced leakage and contamination of radioactive materials. The incident required specialized recovery and decontamination operations as hospital personnel lacked training in handling radioactive oncology drugs. This failure posed risks not only to staff safety but also to continuity of patient treatment with radiopharmaceuticals, which are time- and condition-sensitive.

***𝐒𝐞𝐫𝐯𝐢𝐜𝐞 𝐏𝐫𝐨𝐯𝐢𝐬𝐢𝐨𝐧***

**A.** The structural damage caused by the tornado resulted in the closure and eventual demolition of the oncology department within St. John’s Regional Medical Center. As a result, patients undergoing active treatment were relocated to a nearby functioning hospital. In addition, a temporary field hospital was rapidly established to continue delivery of basic cancer care. This relocation was necessary because the original hospital’s infrastructure was compromised by rainfall, burst pipes, and window damage, making it unsafe for continued occupancy or clinical service delivery.

***𝐂𝐫𝐞𝐚𝐭𝐢𝐨𝐧 𝐨𝐟 𝐒𝐭𝐨𝐫𝐚𝐠𝐞 𝐑𝐞𝐬𝐨𝐮𝐫𝐜𝐞𝐬***

A. Tornado-related damage led to the destruction of radiotherapy immobilization devices, which are critical for maintaining precision in radiation delivery, especially for head and neck cancers. These devices could not be salvaged and required complete replacement. Consequently, patients already undergoing treatment had to be re-simulated and re-planned, which delayed treatment resumption and compromised continuity. This loss demonstrates how non-drug physical oncology resources are essential and must be protected in disaster planning.

7. Article Title: Challenges associated with providing diabetes care in humanitarian settings

Author, Publication year: Boulle, 2019

The article refers to humanitarian crises broadly (conflict, natural disasters, displacement) but does not consistently stratify strategies/measures specifically by disaster type

- List of the NCD diseases considered in this article:

**Diabetes Mellitus List all of the crises and disasters considered in this article**

(Only non–inside health system crises like natural disasters, conflict, displacement)

- Main focus (included in analysis):

**Armed conflicts (e.g., Iraq, Mosul, Ukraine, Syria), Refugee crises (e.g., Burundian refugees in Tanzania, Syrian refugees in Lebanon and Jordan), Natural disasters (mentioned generally, not as specific case studies), Humanitarian emergencies (Tuberculosis is mentioned as a co-managed disease, not a crisis type)**

**Disease:** Diabetes Mellitus

**Crisis: Armed Conflict (e.g., Iraq, Mosul; Ukraine; Syria; Yemen)**

***DRUG SUPPLY***

**A. Inclusion of insulin in the Interagency Emergency Health Kit revised in 2017**
The article states that insulin, along with glucose meters and strips, was newly included in the 2017 revision of the Interagency Emergency Health Kit. This was significant because previously, the kit had not contained insulin, despite its life-saving necessity in insulin-dependent diabetes. The inclusion aimed to ensure that diabetic emergencies, particularly diabetic ketoacidosis, could be promptly managed in acute crises. The decision was based on accumulated evidence of frequent care interruptions for patients reliant on insulin, especially in displacement and conflict settings where continuity of care is compromised.

**B. WHO stand-alone NCD kit includes medications to enable continuity of diabetes care**
The WHO developed a separate NCD kit to specifically address the needs of patients with chronic diseases like diabetes during emergencies. This kit includes essential medicines and diagnostic tools such as glucose meters and testing strips. Its purpose is to ensure that displaced populations or those affected by conflict can continue their treatment regimens despite instability in local supply chains. The article emphasized that this kit enables the maintenance of glycaemic control and reduces risk of acute decompensations such as hyperglycaemic crises, which are common when insulin supply is disrupted.

**C. Use of local cooling devices and studies on insulin thermostability in conflict zones**
To address cold chain limitations for insulin storage in war-affected zones, field teams used devices like clay pots for cooling. The article reported operational efforts to sustain insulin efficacy under high temperature conditions. These efforts were informed by studies showing insulin can retain stability even when stored above room temperature for limited periods. In emergency environments such as Mosul or remote conflict zones in South Sudan, electricity for refrigeration is often unavailable, making such local solutions critical for safe medication administration.

***SERVICE PROVISION***

**D. Implementation of mobile clinics during the Iraq conflict provided a light diabetes care system with basic follow-up and glucose meters**
During the Iraq conflict (2015–2016), Médecins Sans Frontières operated mobile clinics that offered minimal yet vital diabetes services. Due to insecurity and population mobility, the model emphasized simplicity: limited point-of-care tools (glucose meters, sphygmomanometers), basic education, and essential medicines. The goal was to ensure patients did not experience dangerous lapses in care. These clinics adapted care to the acute phase of crisis where access to comprehensive care facilities was impossible.

**E. Point-of-care tests (HbA1c and creatinine) were used in protracted conflict in Ukraine**
In the buffer zone of the Ukrainian conflict, where instability persisted over time, diabetes care transitioned from basic to advanced. Teams introduced HbA1c and creatinine monitoring. HbA1c enabled assessment of long-term glycaemic control; creatinine assessment identified early kidney complications. These tests were conducted using portable devices suited for low-resource environments. This transition exemplified how extended crisis duration permits implementation of more robust chronic disease management protocols.

**F. Integration of diabetes care into primary health care or stand-alone NCD clinics in Syria (Lebanon and Jordan response)**
In stable or protracted crisis zones like Syrian refugee camps in Lebanon and Jordan, diabetes services were embedded in national or NGO-run primary healthcare systems. This integration facilitated continuity of care, comprehensive education, and referral pathways for complications. Unlike acute responses, these settings allowed use of laboratory diagnostics and regular clinical reviews, enabling control of glycaemia and prevention of complications such as diabetic foot ulcers or retinopathy.

**G. Development and application of simplified diabetic ketoacidosis protocols in Agok, South Sudan due to absence of advanced equipment**
In Agok, absence of laboratory testing and syringe pumps made standard DKA protocols impractical. Field staff implemented a simplified version using basic tools and adjusted doses based on clinical signs and glucose meter readings. These adaptations ensured safe treatment in environments with minimal medical infrastructure, thus preventing high mortality from acute decompensation.

***SUPPORT***

**H. Provision of glucose meters to some patients to enable self-monitoring**
The article indicated that in some conflict and emergency zones, glucose meters were distributed to patients, particularly those on insulin. This enabled self-monitoring of fasting and post-prandial glucose levels, essential for dose adjustments and prevention of hypoglycaemia. In settings where follow-up was irregular, empowering patients to assess their own control reduced dependence on disrupted health services.

**I. Use of patient-held booklets to improve information continuity and support mobility of patients**
In displacement settings, patient-held records served as a tool for continuity. These booklets contained diagnosis, medications, insulin regimens, and complications. They enabled seamless care transitions between facilities and protected against loss of clinical data when health systems collapsed or access was inconsistent.

**J. Advocacy for adequate food supply in settings with insulin-dependent diabetes (e.g., East Africa refugee camps)**
The article highlighted two refugee camps in East Africa where food rations from the World Food Programme were cut due to funding shortfalls. Patients with type 1 diabetes were unable to maintain regular meal timing, increasing their risk of severe hypoglycaemia. Field staff advocated for reinstatement of rations and emphasized the need for synchronizing nutritional support with insulin availability to ensure safe self-management.

***TRAINING OF FORCES***

**K. On-the-job training and telemedicine support (Médecins Sans Frontières) during armed conflict settings**
Due to the shortage of skilled professionals in conflict areas, MSF implemented real-time telemedicine platforms. Field doctors received remote guidance on complex diabetes cases, including insulin titration and complication management. On-the-job training reinforced these protocols, addressing knowledge gaps for clinicians used to managing only acute illnesses.

**L. Use of simplified clinical guidelines and supervision systems for staff unfamiliar with chronic care models**
Teams used stripped-down algorithms tailored to local drug availability and health worker competence. These were often based on international standards but adjusted to realities of the crisis. Supervisors were trained specifically to oversee chronic care services, moving beyond the single-contact model common in humanitarian medicine. This enabled continuity of care for diabetes within constrained systems.

***STRATEGIC / OPERATIONAL PLANS***

**M. Inclusion of diabetes in WHO Action Plan and SDGs, with recommendation for operational planning in humanitarian response**
The article stated that diabetes has been officially included in the WHO Global Action Plan and the UN SDGs. It emphasized the operational challenge of translating this inclusion into actionable plans in emergencies. Agencies were advised to prepare for NCD integration from the outset, rather than as an afterthought once acute needs subside.

**N. Emphasis on early integration of diabetes care into host country health systems, as seen in conflict-affected Mali**
Following the coup d’état in Mali, diabetes care was stratified by population (e.g., displaced persons, refugees, urban residents). The article advocated for strategic integration with national systems to avoid parallel care structures and to sustain services post-crisis. This ensured continuity and harmonization with local policies, reducing fragmentation of services.

**Crisis: Refugee Crisis (e.g., Tanzania, Lebanon, Jordan)**

***DRUG SUPPLY***

**A. Imported diabetes medications used in refugee settings (e.g., Tanzania)**
In the Nduta camp, MSF used imported essential diabetes medications aligned with their internal formularies. The challenge noted was in continuity—patients had been prescribed different drugs prior to displacement. To mitigate this, MSF adapted medication lists and monitored for side effects, especially when switching from familiar regimens.

***SERVICE PROVISION***

**B. Chronic disease clinics established in Nduta camp (Tanzania) for Burundian refugees**
Faced with a high burden of diabetes among refugees, MSF set up dedicated chronic care clinics. These clinics managed both newly diagnosed and known diabetic patients. Services included medication refills, complication screening, and education. Their establishment acknowledged the importance of chronic care in humanitarian contexts, especially where protracted displacement was anticipated.

**C. Integrated diabetes care with HIV and tuberculosis services in Lebanon and Jordan**
In these settings, NCD clinics managed comorbid patients with diabetes and HIV/TB. The integrated model leveraged existing chronic care infrastructure and trained teams to handle overlapping regimens, drug interactions, and patient follow-up. The article noted operational efficiencies gained from shared logistics and clinical supervision.

***SUPPORT***

**D. Dietary advice adapted for refugees with diabetes; difficulty managing type 1 diabetes noted due to food insecurity**
Type 1 patients in East Africa refugee camps experienced irregular food supply. The article detailed how field clinicians struggled to match insulin timing with unpredictable meals. Dietary counselling had to consider ration patterns, cultural norms, and the caloric needs of insulin-treated patients. In the absence of such alignment, acute hypoglycaemic episodes increased.

***TRAINING OF FORCES***

**E. Generalist doctors and midwives managed diabetes in pregnancy in refugee camps**
In Lebanon and Jordan, midwives and general practitioners were trained to jointly manage diabetes and pregnancy. This addressed the shortage of endocrinologists or obstetricians in humanitarian settings. Pregnant patients were monitored for glycaemic control, and simplified care pathways were used to ensure maternal and fetal safety.

**Crisis: General Humanitarian Settings**

***DRUG SUPPLY***

**A. Mention of high cost of insulin and variability in supply; recommendation for field-adapted monitoring devices**
Despite being on WHO's essential medicines list, insulin remains costly, especially in analog form. The article reported difficulties in procurement, compounded by the lack of transparent pricing. To mitigate care gaps, field teams utilized local or imported products and sought affordable glucose meters that function under high temperatures.

***SERVICE PROVISION***

**B. Use of patient registration books, appointment tracking, and recall systems for follow-up**
Field teams employed basic systems to track visits and follow-ups, critical in mobile populations. These included patient logs, recall slips, and manual appointment books. Where feasible, electronic records were introduced. This facilitated continuity and monitoring of glycaemic trends across encounters.

***SUPPORT***

**C. Self-management education including glucose checks and wound care to reduce service dependence**
Education emphasized self-care tasks like glucose monitoring, insulin adjustment, and foot care. These skills were vital where clinic access was limited or irregular. Teaching patients to detect early signs of complications reduced emergency visits and preserved scarce resources.

***STRATEGIC / OPERATIONAL PLANS***

**D. Coordination between humanitarian actors (UNHCR) to ensure harmonized diabetes care across settings**
UNHCR led an interagency working group on NCDs to standardize diabetes interventions. The goal was cross-organizational learning and consistent care regardless of provider. Shared protocols, indicators, and logistics planning were encouraged to reduce fragmentation.

***PROVISION OF BUDGET***

**E. Interagency research consortium established to jointly apply for diabetes care funding**
Due to donor hesitancy around funding chronic care, agencies collaborated on proposals to secure independent funding. The goal was to ensure sustained implementation of diabetes services post-deployment.

**F. Nurse-led diabetes care cost reported at US$185 per person/year in DR Congo**
The article cited MSF costing data showing that with appropriate task-shifting and simplified protocols, diabetes care could be delivered affordably, debunking assumptions about prohibitive expense.

8. Article Title: The burden of diabetes and use of diabetes care in humanitarian crises in low-income and middle-income countries

Author, Publication year: Kehlenbrink, 2019

The article discusses cyclone, flood, and storm-related disasters as a combined category throughout, and does not stratify specific strategies or measures by disaster type. But the article does stratify the impact and relevant considerations by disease type

- List of the NCD diseases considered in this article:

**Type 1 Diabetes, Type 2 Diabetes, Gestational Diabetes, Hypertension, Heart Failure**

- List all of the crises and disasters considered in this article

(Only non–inside health system crises like natural disasters, conflict, displacement)

- Main focus (included in analysis): **Armed Conflict, Forced Migration, Natural Disasters, Food Insecurity, War, Persecution**

**Disease:** Type 1 Diabetes & Type 2 Diabetes

**Crisis: Armed Conflict**

***DRUG SUPPLY***
A. During the Croatian war in 1991, analysis from the national diabetes registry indicated that insulin was being used to manage patients with type 1 diabetes. Glycaemic control outcomes showed a median fasting glucose of 194.4 mg/dL (IQR 66.7) in displaced individuals and 183.6 mg/dL (SD 52.2) in non-displaced individuals. HbA1c levels were 9.8% (SD 8.7–11.5) in displaced and 9.1% (SD 8.0–10.7) in non-displaced persons with type 1 diabetes. This evidence demonstrates that insulin remained available during the conflict, though not optimally, as glycaemic control remained substandard across both groups. This highlights how supply may have persisted, but possibly with inconsistent storage conditions, delivery, or use efficiency.

B. In Bosnia and Herzegovina, a hospital-based study during armed conflict showed significant improvement in glycaemic control in patients with type 2 diabetes. Mean fasting glucose dropped from 207.0 mg/dL (SD 54.1) to 176.4 mg/dL (SD 61.3), and HbA1c from 10.3% (SD 2.4) to 9.0% (SD 2.0); p<0.01. These improvements occurred despite a crisis, suggesting that insulin or oral hypoglycaemics remained at least partially available. However, the improvements might also have resulted from decreased food intake or weight loss rather than optimized pharmacotherapy.

***SERVICE PROVISION***
C. In Sarajevo, the hospital-based study during wartime (1992–1995) revealed a significant reduction in glycaemic indicators among insulin-dependent patients. Mean fasting glucose decreased from 207.0 mg/dL (SD 54.1) pre-war to 176.4 mg/dL (SD 61.3) post-war, while HbA1c decreased from 10.3% (SD 2.4) to 9.0% (SD 2.0); p<0.01. This demonstrates that structured service provision at hospitals for people with type 1 diabetes continued during the war and could even support improved metabolic outcomes. These improvements occurred despite crisis conditions and likely reflect adjustments in caloric intake, physical activity, and hospital oversight of insulin use.

***SUPPORT (CLINICAL FOLLOW-UP AND MONITORING)***
D. The same Sarajevo study reported a mean weight loss of 11.7 kg (SD 8.2 kg) among patients with type 1 and type 2 diabetes during the war period. This unintended weight reduction may have contributed to reduced insulin resistance, potentially influencing improved glycaemic control. However, such changes occurred in the context of nutritional deprivation, suggesting that follow-up and monitoring of clinical nutrition was either absent or unable to prevent such negative outcomes.

***CARDIOVASCULAR RISK MANAGEMENT***
E. In the Sarajevo cohort, the number of patients with type 2 diabetes and hypertension (BP >140/90 mmHg) declined from 25 before the war to 14 after the war; p<0.01. This might reflect improved compliance, altered diet, or changes in stress physiology, but in the context of conflict, it could also be a side effect of weight loss and undernutrition. Nevertheless, it illustrates the interaction between crisis dynamics and cardiovascular risk among patients with type 2 diabetes.

**Crisis: Forced Migration**

***DRUG SUPPLY***
A. Among Syrian refugees with type 1 diabetes in Iraq, 63.6% reported episodes of symptomatic hypoglycaemia, and 36.6% experienced at least one episode of diabetic ketoacidosis. These high rates of acute complications clearly reflect inadequate or inconsistent insulin supply, delayed administration, or failure in dose management. Both hypoglycaemia and ketoacidosis in type 1 diabetes are direct outcomes of unbalanced insulin delivery, dietary intake, and stress, which are exacerbated under displacement conditions. This implies significant weaknesses in uninterrupted drug supply chains during forced migration.

B. Among Syrian refugees in Iraq, 312 of 328 diabetic patients (95%) had type 2 diabetes. Mean HbA1c was 8.8% (SD 1.7), with 62% of patients exceeding 8.0%. This reflects uncontrolled disease due to insufficient access to anti-diabetic medications or poor treatment adherence under unstable conditions.

***SERVICE PROVISION***
C. In this cohort, 16 individuals (5% of 328 patients) had type 1 diabetes. Their mean HbA1c was 9.3% (SD 1.9), with only 9.1% of patients having HbA1c values below 7.5%, and 81.8% exceeding 8.0%. These data reflect suboptimal service provision, where access to regular glucose monitoring, insulin titration, and physician consultation were likely disrupted. The elevated HbA1c values underscore the inadequacy of structured diabetes services in refugee camp environments.

D. In UNRWA clinics in Jordan, 42% of 2851 patients had postprandial glucose measured, with 50% achieving levels <180 mg/dL. One year later, documentation improved with 99% of 9691 patients monitored, and 65% showing controlled values. This demonstrates how structured service delivery, even in refugee settings, can yield improved outcomes when adequately implemented.

***TRAINING OF FORCES / CLINICAL MANAGEMENT SYSTEMS***
E. The same analysis showed that only 9.1% of type 1 diabetes patients received statins, and 67.3% of those on antihypertensives used ACE inhibitors. Systolic blood pressure was controlled in 81.8%, and diastolic in 72.6%. For type 2 diabetes, 55.4% were on antihypertensives (67.3% on ACE inhibitors), systolic BP was controlled in 54.9%, and diastolic in 36.3%. Furthermore, 49.7% were on statins. These findings indicate partial implementation of cardiovascular risk management despite displacement, although gaps in medication coverage and therapeutic outcomes remain apparent.

***CLINICAL MONITORING SYSTEMS (EMRs)***
F. The observed improvement in glycaemic control in UNRWA clinics was directly linked to the improved performance of electronic health record (eHealth) systems, which increased the recording and follow-up of glucose measurements. The systematic use of EMRs facilitated continuity of care and adherence monitoring, thereby improving patient outcomes.

**Crisis: Food Insecurity**

***DRUG SUPPLY***
A. The article stated that food insecurity, when combined with hypoglycaemic agents, significantly increases the risk of hypoglycaemia in individuals with type 1 and type 2 diabetes. This reflects the inherent danger of administering insulin or sulfonylureas without adequate caloric intake, emphasizing the need to align medication supply with nutritional support.

***STRATEGIC OR OPERATIONAL PLANNING***
B. The article recommended revising glycaemic targets to ensure patient safety in food-insecure environments. Maintaining conventional control targets could lead to hypoglycaemic events when food access is erratic. Therefore, planning must incorporate flexible treatment algorithms that respond to nutritional availability.

**Crisis: War**

***DRUG SUPPLY***
A. The article described insulin access in wartime as highly problematic due to the collapse of health-care systems, exposure to violence, and inability to maintain cold storage. These contextual constraints severely endanger the survival of patients with type 1 diabetes, who are insulin-dependent and suffer rapid metabolic decompensation without timely administration.

B. In Yemen, a study comparing pre- and post-war glycaemic control in type 2 diabetes patients showed an increase in HbA1c from 7.7% (SD 1.9) to 9.4% (SD 2.4); p<0.001. This deterioration is attributed to diminished access to anti-diabetic medications and compromised health services during the war.

***CARDIOVASCULAR RISK MANAGEMENT***
C. Despite deterioration in glycaemic control, the same study reported improved blood pressure management in patients with type 2 diabetes after the war. This paradox may be due to altered dietary patterns, reduced sodium intake, or changes in stress-related physiology, but highlights the need for more granular data to interpret outcomes accurately.

***CREATION OF STORAGE RESOURCES***
D. Specific mention was made of the barrier posed by insulin’s cold storage requirement. In war zones, maintaining the cold chain is often impossible due to electricity outages, lack of refrigeration, and mobile populations, making it essential to prioritize the development of field-ready storage technologies and supply stabilization plans.

**Disease:** Gestational Diabetes

**Crisis: Forced Migration**

***SERVICE PROVISION***
A. In a study of displaced Yazidi women in Iraq, 2.8% of pregnant women were reported to have gestational diabetes. However, over half of the pregnant women interviewed had not received a single antenatal visit by their third trimester and therefore had not been screened for gestational diabetes. This indicates that antenatal services were severely lacking in accessibility and coverage. The absence of routine antenatal care led to underdiagnosis and potentially unmanaged gestational diabetes, presenting risks to both maternal and fetal health.

B. A separate study conducted in a refugee camp at the Thailand–Myanmar border assessed the prevalence of gestational diabetes using a 75 mg oral glucose tolerance test. The estimated prevalence was 10.1% based on the Hyperglycaemia and Adverse Pregnancy Outcomes (HAPO) trial cutoff values. The sample included 228 pregnant women. The number of all pregnant women in the catchment area was not recorded, but the data show that gestational diabetes was present in this displaced population and that structured screening using biochemical testing was possible in camp settings when integrated with routine maternal health services.

***STRATEGIC OR OPERATIONAL PLANNING***
C. The article stated that screening and management of hyperglycaemia in pregnancy needs to be integrated into routine perinatal care in humanitarian settings. This recommendation is based on the evidence that gestational diabetes increases the maternal risk of developing type 2 diabetes later in life and is associated with serious adverse pregnancy outcomes, as well as transgenerational metabolic consequences for the fetus. Implementation of integrated maternal–diabetes services is considered essential in crisis contexts with high birth rates.

**Disease:** Hypertension

**Crisis: Armed Conflict**

***CARDIOVASCULAR RISK MANAGEMENT***
A. In a study conducted in Sarajevo during wartime, the proportion of individuals with diabetes (which includes those with hypertension as a comorbidity) having blood pressure greater than 140/90 mmHg declined from 25 before the war to 14 after the war; p<0.01. This change was attributed to conditions during the war that may have affected dietary intake, body weight, or medication adherence. The data suggest that cardiovascular risk factor monitoring and control occurred to some degree despite the conflict and that health services remained partially functional for blood pressure surveillance.

**Crisis: Forced Migration**

***CARDIOVASCULAR RISK MANAGEMENT***
B. Among Syrian refugees with diabetes in Iraq, blood pressure control was assessed separately for individuals with type 1 and type 2 diabetes. For individuals with type 1 diabetes, systolic blood pressure was controlled in 81.8% and diastolic in 72.6%. For individuals with type 2 diabetes, systolic blood pressure was controlled in 54.9% and diastolic in 36.3%. Additionally, 55.4% of those with type 2 diabetes were treated with antihypertensive drugs, and among those treated, 67.3% were on angiotensin-converting enzyme (ACE) inhibitors. These results reflect the existence of structured hypertension management practices and medication use in refugee settings, although overall control rates remained suboptimal, especially for diastolic blood pressure in type 2 diabetes patients.

**Disease:** Heart Failure

**Crisis: Forced Migration**

***CARDIOVASCULAR RISK MANAGEMENT***
A. Among Syrian refugees with diabetes in Iraq, cardiovascular disease was reported as a comorbidity, including diagnoses of congestive heart failure. However, no specific therapeutic or service-based measures directly addressing heart failure management were described. Only general cardiovascular risk management was implemented, including the prescription of antihypertensives and statins. Among all individuals with diabetes, 55.4% were treated with antihypertensives, with 67.3% of these on ACE inhibitors. Statins were used by 49.7% of patients. While heart failure was acknowledged as a diagnosis, its management protocols were not separately defined within this cohort.

9. Article Title: Not an ‘either/or’: Integrating mental health and psychosocial support within non-communicable disease prevention and care in humanitarian response

Author, Publication year: Gyawali, 2021

The article addresses humanitarian crises broadly (e.g., migration, conflict, and the COVID-19 pandemic) without stratifying specific strategies or measures by each type of disaster. The article discusses NCDs and mental ill-health in a combined manner and promotes integration of care across disease types without stratifying strategies/measures for each NCD individually.

- List of the NCD diseases considered in this article:

**Cardiovascular diseases, Cancers, Diabetes, Mental ill-health (Depression, Anxiety, PTSD), Chronic respiratory diseases**

- List all of the crises and disasters considered in this article

(Only non–inside health system crises like natural disasters, conflict, displacement)

**Humanitarian settings (including conflict-affected areas and refugee contexts), COVID-19 pandemic.**

**Disease:** Cardiovascular Diseases
**Crisis: Humanitarian Settings (including conflict-affected areas and refugee contexts)**

***SERVICE PROVISION***

A. Cardiovascular diseases were explicitly discussed as part of the broader category of physical noncommunicable diseases (NCDs) that are highly prevalent in humanitarian settings. The article identifies that health systems in such contexts operate in vertical silos, with NCD care and mental health and psychosocial support (MHPSS) managed separately. This fragmentation leads to inefficient and time-consuming service provision for patients with cardiovascular diseases, who often also suffer from comorbid mental health conditions. Therefore, integrated service provision within primary healthcare is proposed as a necessity to manage cardiovascular conditions more effectively, allowing for simultaneous care that responds to both physical and mental health needs.

B. The article emphasizes that integration must follow a tiered approach, offering basic community-based MHPSS support, progressing to individual-level clinical care where required. For cardiovascular patients, this model would reduce fragmentation and enable more timely detection, continuous monitoring, and support, particularly in contexts where both physical health and psychosocial wellbeing are affected by displacement, conflict, or trauma.

***TRAINING OF FORCES***

C. The separation of cardiovascular care from other services is attributed partly to a shortage of trained personnel who can deliver integrated care. The article discusses the inadequacy of relying exclusively on specialists, such as cardiologists, in humanitarian settings. It explicitly recommends redistribution of tasks to general practitioners and lay health workers who can be trained to deliver cardiovascular-related interventions under supervision. This approach is proposed to increase accessibility and efficiency of cardiovascular services for displaced and conflict-affected populations who may not have access to tertiary or specialized care centers.

D. Furthermore, this task-sharing model allows for culturally and contextually relevant service provision, where trained non-specialists can offer ongoing care and lifestyle guidance for cardiovascular conditions within communities, reducing dependency on overstretched centralized clinical facilities.

***SUPPORT***

E. The article states that individuals living with cardiovascular diseases benefit from strengthened community and family support structures, particularly in humanitarian contexts where formal care may be inaccessible. The support mechanisms include community outreach, peer-led programs, and self-help strategies that encourage treatment adherence and provide psychological encouragement, which are critical to the long-term management of cardiovascular diseases, particularly in unstable environments.

F. Support is not limited to individual interactions but includes community-level psychosocial infrastructure that helps mitigate the isolation and stigma associated with chronic illnesses like cardiovascular diseases. This is particularly vital in humanitarian settings, where instability and trauma further exacerbate disease outcomes.

***IMPROVEMENT OF INTER-SECTORAL COMMUNICATIONS***

G. The article identifies siloed funding and governance models as barriers to effective care for cardiovascular disease. Cardiovascular services are often excluded from integrated strategies due to a lack of coordination between sectors (e.g., mental health, NCDs, primary care). It argues for structural reforms to health system governance to promote communication and coordination across these sectors. Such reforms would enhance the ability to develop integrated care pathways that include cardiovascular disease management alongside other essential services.

H. Better inter-sectoral communication would ensure continuity of care, reduce duplication of services, and enable consistent monitoring and follow-up for cardiovascular patients, all of which are currently impeded by disconnected programmatic streams in humanitarian health systems.

***PROVISION OF BUDGET***

I. The article describes how donor funding in humanitarian settings is typically short-term and highly segmented, favoring vertical disease programs. This limits the availability of comprehensive care for cardiovascular diseases, which require ongoing, integrated support. It advocates for the establishment of sustainable financing models that prioritize integrated service delivery, including cardiovascular care.

J. Such financial restructuring is vital to ensure consistent access to medications, diagnostics, and follow-up care for cardiovascular patients, who often suffer interruptions in care due to fragmented and project-based funding cycles.

***PREPARATION OF STRATEGIC OR OPERATIONAL PLANS***

K. The authors call for the development of evidence-informed guidelines for integrating MHPSS and NCDs. For cardiovascular diseases, this implies that strategic plans must include clear protocols for integration, pathways for cross-referral, and mechanisms for identifying cardiovascular risk in conjunction with mental health assessments. Operational plans are required to ensure that such integration is feasible and adapted to the logistical and resource constraints of humanitarian contexts.

L. These strategic plans should also account for population-specific needs, such as those of refugee or displaced persons with pre-existing cardiovascular conditions, and include elements like contingency planning for medication supply disruptions or the establishment of mobile clinics for remote screening and monitoring.

***ADVOCACY AND POLICY ENGAGEMENT***

M. The article asserts that cardiovascular diseases are often neglected in the prioritization frameworks of humanitarian health responses. It calls on policymakers and humanitarian actors to recognize the intersection between cardiovascular disease and mental ill-health and integrate this understanding into health response strategies. Advocacy is positioned as a tool to elevate cardiovascular care within health agendas that typically favor acute or communicable disease interventions.

N. Policy-level integration would also facilitate regulatory alignment, enabling the flow of resources, personnel, and medications across sectors and improving the accessibility and quality of cardiovascular services in complex humanitarian environments.

**Crisis: COVID-19 Pandemic**

***SERVICE PROVISION***

O. The article discusses how lockdowns and resource reallocation during the COVID-19 pandemic disrupted cardiovascular care in humanitarian contexts. Specifically, it notes reduced access to services for lifestyle management, monitoring, and medication. This disruption significantly impacted individuals with cardiovascular diseases, whose conditions require continuous management. The absence of structured service continuity planning led to gaps in care, potentially worsening health outcomes.

P. The authors argue that integrated, resilient service models are essential to ensure that cardiovascular patients receive uninterrupted care during pandemic-related restrictions. This includes the implementation of flexible service modalities such as mobile health units or telemedicine, which were not adequately developed prior to or during the pandemic in many humanitarian contexts.

***DRUG SUPPLY***

Q. The COVID-19 pandemic interfered with the regular supply of essential medicines for cardiovascular diseases. Lockdowns, supply chain disruptions, and shifting health priorities diverted resources away from chronic disease management. This reduced patients’ ability to access antihypertensive and other cardiovascular medications, risking disease exacerbation.

R. Ensuring the continuity of cardiovascular medication supply chains during crises is identified as a critical objective. The article implicitly calls for contingency planning in pharmaceutical procurement and distribution systems tailored to the needs of cardiovascular patients in humanitarian and pandemic scenarios.

***PREPARATION OF STRATEGIC OR OPERATIONAL PLANS***

S. The article draws on the experience of COVID-19 to underline the urgent need for preparedness plans that include cardiovascular diseases as core components. It recommends integrating cardiovascular disease management into pandemic response frameworks to prevent the sidelining of chronic care in emergency planning.

T. Such plans should include protocols for maintaining contact with cardiovascular patients, triaging based on risk, and deploying mobile or decentralized services during movement restrictions.

***ADVOCACY AND POLICY ENGAGEMENT***

U. The authors state that the COVID-19 pandemic revealed the weaknesses of fragmented health responses that isolate chronic disease care. They advocate for policymakers to incorporate cardiovascular services into emergency response structures and funding mechanisms. This inclusion would allow for a more holistic response to future crises, ensuring that patients with cardiovascular diseases are not neglected during health emergencies.

**Disease:** Cancers
**Crisis: Humanitarian settings (including conflict-affected areas and refugee contexts)**

***SERVICE PROVISION***

A. The article stresses that people living with cancers, among other non-communicable diseases (NCDs), frequently suffer from psychological distress and mental ill-health such as depression and anxiety due to the chronic and often life-threatening nature of their condition. These psychological consequences, if left unaddressed, can interfere with help-seeking behavior, adherence to cancer treatment, and treatment outcomes. Therefore, integrating mental health and psychosocial support (MHPSS) services directly into NCD care, including cancer services, is emphasized to ensure comprehensive care. The authors argue that the siloed nature of care delivery in humanitarian settings fragments cancer services and leads to inefficiency, redundancy, and reduced continuity of care for patients. The proposed model is a bidirectional integration, where cancer care is embedded into MHPSS services and vice versa.

B. Integration aims to prevent missed diagnoses and delayed interventions for psychological issues that arise during cancer treatment, which are common in crisis settings where mental distress may be overlooked. The article warns that current vertical models of care isolate cancer patients from needed mental health services, thereby exacerbating disease burden and impairing recovery.

***SUPPORT***

A. Culturally appropriate and patient-centered care is recommended to address the unique psychological and physical needs of individuals with cancer in humanitarian settings. Patients living with cancers are encouraged to participate in the design and development of programs that acknowledge their specific sociocultural and medical contexts. This approach aims to overcome the stigma and mistrust that cancer patients often experience when accessing MHPSS or medical services in settings affected by displacement or conflict.

B. The article describes that positioning cancer-related support programs in community settings, such as schools and community halls, rather than clinical settings, can improve their acceptability and accessibility among populations affected by crises. This is particularly relevant for people with cancer who may otherwise avoid clinical facilities due to stigma, fear of discrimination, or previous negative experiences.

***PREPARATION OF STRATEGIC OR OPERATIONAL PLANS***

A. The article calls for the development and implementation of evidence-informed operational guidelines that support the integration of MHPSS and NCD care, including for cancers. It highlights the current lack of standardized protocols for managing cancer care within humanitarian settings and points to the fragmentation caused by disjointed planning efforts. As a result, the proposed solution is to align service provision with integrated operational strategies that allow for coordinated and holistic care across both physical and psychological domains.

B. Strategic plans are expected to guide humanitarian actors in shifting from disease-specific and fragmented cancer interventions to more sustainable and responsive systems that take into account the comorbidity of cancers with mental health conditions, particularly in crisis-affected populations.

***TRAINING OF FORCES***

A. There is a critical shortage of specialized oncologists and mental health professionals in humanitarian settings. To address this, the article advocates for the redistribution of cancer-related care tasks to general practitioners and lay health workers through training programs. This measure ensures broader reach and accessibility of essential services for cancer patients, especially in remote or underserved crisis zones.

B. Lay health workers trained to deliver integrated cancer care and MHPSS services can help overcome human resource constraints and provide continuity of support for individuals suffering from cancer. The article notes that when supported by supervision from multidisciplinary teams—including psychologists, physicians, and nurses—these trained personnel can help maintain quality of care in complex settings.

***PROVISION OF BUDGET***

A. The article highlights that donor funding in humanitarian settings is often vertical, short-term, and segmented, which restricts the ability to provide sustained and integrated services for chronic conditions such as cancer. It recommends the establishment of sustainable financing mechanisms that support integrated MHPSS and NCD programs, including cancer care, to ensure continuity and efficiency.

B. Cancer care is particularly affected by fragmented budgeting because the need for long-term treatment and follow-up is incompatible with project-based funding cycles. The article argues that lack of dedicated financial streams impairs the delivery of cancer services, necessitating an overhaul of humanitarian financing to prioritize chronic disease management.

***IMPROVEMENT OF INTER-SECTORAL COMMUNICATIONS***

A. The authors underscore the importance of multi-sectoral collaboration to effectively deliver integrated care for people with cancers in crisis settings. They advocate for a system where health, psychosocial, and community sectors work together to address the multi-dimensional needs of cancer patients, ensuring that services are not duplicated or overlooked.

B. Inter-sectoral integration is also viewed as critical to reducing stigma and discrimination that cancer patients face. The article proposes that collaboration between healthcare providers, community leaders, and religious figures can support education, outreach, and stigma reduction, thus improving uptake of cancer services and associated mental health care.

**Crisis:** COVID-19 pandemic

***SERVICE PROVISION***

A. The article explicitly notes that the COVID-19 pandemic disrupted the provision of care for chronic conditions including cancers. The lockdowns and diversion of healthcare resources toward COVID-19 response resulted in reduced access to cancer monitoring, follow-up, and clinical visits. This service interruption impeded the timely management of cancer and its associated complications in already vulnerable populations residing in humanitarian contexts.

B. These disruptions underscore the need for flexible service delivery models that can operate during public health emergencies without interrupting essential cancer care. The article calls for systems that avoid siloed approaches and instead prioritize continuity of cancer services during pandemic conditions.

***DRUG SUPPLY***

A. The article reports that the COVID-19 lockdowns negatively impacted access to essential medicines for chronic diseases, including cancers. Barriers to facility access, transportation, and supply chain disruptions led to interruptions in treatment regimens critical to the survival and quality of life of cancer patients. The article does not provide exact drug names or logistics data but acknowledges that treatment continuity was compromised.

B. This issue demonstrates the importance of resilient supply chains and decentralized distribution systems for medications used in cancer therapy, especially during pandemics when centralized service delivery may be impaired.

**Disease:** Diabetes
**Crisis: Humanitarian settings (including conflict-affected areas and refugee contexts)**

***SERVICE PROVISION***

A. The article reports that diabetes care in humanitarian settings is typically structured around vertical models that isolate services by disease area. This fragmentation undermines the continuity and quality of care for people with diabetes, particularly when mental ill-health co-occurs with physical NCDs. The authors emphasize that diabetes frequently co-exists with mental conditions such as depression and anxiety, and this comorbidity negatively influences treatment adherence and prognosis. Integration of mental health and psychosocial support (MHPSS) into diabetes care is proposed as a corrective measure to address these challenges. Such integration enables a more holistic response that can increase service uptake, improve disease outcomes, and mitigate the compounded effects of psychological stress on diabetes management.

B. In a study of Syrian refugees living with diabetes in Jordan, it was explicitly reported that patients acknowledged the psychological dimensions of their illness but refrained from attending MHPSS clinics due to fear of stigmatization. This avoidance behavior directly compromised their ability to manage their diabetes effectively, as mental health challenges went untreated and hindered adherence to diabetic care protocols. The article cites this as evidence that the current service provision fails to respond to the needs of diabetic patients in crisis contexts where mental health and chronic disease care are siloed.

***SUPPORT***

A. The article emphasizes that support structures for diabetes management must be culturally adapted and involve patients in decision-making. It argues that people living with diabetes in humanitarian contexts often experience dual stigma—both from the chronic illness itself and from associated mental health burdens. Creating programs that actively involve diabetes patients in their own care design fosters cultural acceptability and encourages greater engagement, which in turn enhances treatment adherence and disease monitoring outcomes.

B. Support services for diabetes patients should be relocated from conventional clinical environments to community-based spaces such as schools or community halls. This spatial shift is recommended to counter the stigma experienced by diabetes patients when seeking help, particularly in MHPSS-integrated models. By de-medicalizing the support environment, the authors argue that these patients may feel more comfortable accessing resources critical to diabetes control, including counseling, peer support, and nutritional education.

***PREPARATION OF STRATEGIC OR OPERATIONAL PLANS***

A. The article calls for operational guidelines that incorporate MHPSS integration into diabetes care as a central tenet of humanitarian health planning. It identifies the current absence of structured protocols for addressing diabetes alongside mental health conditions as a major barrier to effective service delivery. Without coordinated plans, service implementers continue to provide isolated interventions that neglect the psychological factors that significantly affect diabetes prognosis, including stress-induced hyperglycemia and low adherence to medication regimens.

B. The authors argue that planning documents must incorporate diabetes explicitly within multi-disease management frameworks that recognize the high burden of comorbidity in humanitarian populations. In the absence of such strategies, diabetic patients face increased risks of complications and diminished survival outcomes.

***TRAINING OF FORCES***

A. The article identifies a shortage of specialized staff as a critical obstacle to delivering comprehensive diabetes care in humanitarian settings. In response, it promotes task-shifting strategies that involve training lay health workers and general practitioners to provide diabetes-related services, including blood glucose monitoring, medication administration, and mental health screening. These cadres are to be supervised by multidisciplinary teams to ensure the quality of care and the integration of MHPSS with diabetes management.

B. Lay health workers trained in diabetes care are essential in reaching underserved populations where specialists are absent. The article states that this model expands the health system’s capacity to address diabetes while simultaneously addressing associated mental health challenges, improving adherence and reducing mortality risk in populations facing displacement and systemic disruption.

***PROVISION OF BUDGET***

A. The article critiques the predominant donor funding model for its emphasis on vertical, short-term, and disease-specific programs. This funding structure fails to support the complex needs of diabetic patients who require sustained, integrated care. The authors argue that funding mechanisms must be restructured to support long-term diabetes care that includes psychological support, lifestyle interventions, and medication continuity.

B. Budget limitations that force project-based diabetes interventions undermine consistent monitoring and treatment, especially for displaced persons who need uninterrupted access to insulin, oral hypoglycemics, and dietary support. The article urges policymakers to prioritize financial models that ensure stable and integrated funding streams for diabetes and related MHPSS services.

***IMPROVEMENT OF INTER-SECTORAL COMMUNICATIONS***

A. The article recommends that effective diabetes care in humanitarian settings should involve coordinated action between the health sector and community actors such as religious leaders and local organizations. This multi-sectoral approach is aimed at addressing both the clinical and psychosocial aspects of diabetes, particularly in reducing stigma and improving public understanding of the disease and its management.

B. Inter-sectoral collaboration is described as essential in aligning service delivery with community norms and expectations. For diabetic patients, this facilitates improved help-seeking behavior and ensures that support systems are trusted, accessible, and adapted to the local context, thereby enhancing both physical and mental health outcomes.

**Crisis: COVID-19 pandemic**

***SERVICE PROVISION***

A. The article reports that during the COVID-19 pandemic, diabetes patients in humanitarian contexts faced severe disruptions in service provision. Lockdowns and containment measures restricted access to clinics, thereby preventing routine glucose monitoring, counseling, and lifestyle management support. These interruptions increased the risk of complications due to poorly controlled blood sugar levels and reduced adherence to prescribed care regimens.

B. The article calls for integrated, pandemic-resilient health service models that can ensure continuity of care for diabetes even when face-to-face visits are restricted. This includes mobile health interventions, remote counseling, and community-level follow-up to prevent loss to follow-up and adverse outcomes during public health emergencies.

***DRUG SUPPLY***

A. The COVID-19 pandemic severely affected drug supply chains, disrupting the availability of essential medicines for diabetes patients. The article states that these disruptions were primarily due to lockdowns that limited facility access and interrupted delivery logistics. As a result, patients faced lapses in insulin and oral medication availability, contributing to poor glycemic control and elevated risk of complications such as diabetic ketoacidosis.

B. The absence of a resilient supply system left diabetes patients vulnerable to life-threatening interruptions in therapy. The article identifies the need to reinforce supply chains for diabetes medications as a central agenda item in pandemic preparedness planning for humanitarian contexts.

**Disease:** Mental ill-health (Depression, Anxiety, PTSD)

**For Humanitarian Settings (including conflict-affected areas and refugee contexts)**

**SERVICE PROVISION**
A. The article explicitly describes the integration of MHPSS into NCD prevention and care as essential for addressing the high burden of mental ill-health, such as depression, anxiety, and PTSD, in humanitarian settings. It states that this integration requires a tiered approach tailored to individual needs: basic MHPSS support for all, psychosocial support or peer-support groups for vulnerable populations, and individual counselling and treatment for persons suffering from chronic mental ill-health and severe distress that impairs daily functioning. This tiered model is required because mental ill-health in these settings is linked to trauma exposure, displacement, and chronic stressors that reduce help-seeking behavior, worsen treatment adherence, and impair outcomes for co-morbid conditions. Fragmented care systems that separate MHPSS from other services are described as inefficient, leading to redundancy, service gaps, and failure to meet the complex mental health needs of populations in humanitarian settings.

**SUPPORT**
B. The article explains that MHPSS-focused awareness and sensitization through social media, peer counselling, and community and outreach involvement are necessary to reduce the stigma and discrimination that prevent individuals with mental ill-health—specifically depression, anxiety, and PTSD—from seeking care. Stigma, described as culturally rooted and pervasive in many humanitarian contexts, leads to patients being reluctant to attend clinic-based MHPSS services, as shown by the explicit example of Syrian refugees in Jordan who, despite recognizing the psychological dimensions of their illnesses, avoided MHPSS services due to fear of discrimination. This reluctance extends to health providers themselves, with the article stating that doctors may not refer patients to MHPSS out of concern for stigma. The article asserts that MHPSS-focused awareness efforts targeting both patients and healthcare providers are critical to dismantle these barriers, increase access to care, and encourage treatment adherence in individuals with mental ill-health.

**TRAINING OF FORCES**
C. The article explicitly emphasizes the training of general practitioners, lay health workers, family members, and community supporters as a strategy to address the severe shortage of specialized mental health professionals, such as psychiatrists and psychologists, in humanitarian settings. It explains that the limited availability of specialists impedes effective mental health service delivery, and that redistributing care tasks to non-specialists—under appropriate supervision from specialists and peers—is vital to expanding the reach of MHPSS for individuals with depression, anxiety, and PTSD. Training these cadres is described as essential for overcoming workforce shortages and delivering community-level MHPSS that can address mental ill-health in a more accessible and culturally sensitive manner.

**PREPARATION OF STRATEGIC OR OPERATIONAL PLANS**
D. The article explicitly calls for the development of guidelines, policies, and research agendas to promote the integration of MHPSS into NCD prevention and care. It states that without such frameworks, mental ill-health—such as depression, anxiety, and PTSD—will remain marginalized within fragmented, vertical care models that fail to account for the interdependencies between mental and physical health in humanitarian settings. The article explains that the absence of integrated, operational strategies results in ineffective care, missed opportunities for holistic treatment, and worsened mental health outcomes for populations affected by crises. It also stresses the need for sustainable financing mechanisms to support the integration of MHPSS services, warning that without this, the long-term mental health needs of individuals with depression, anxiety, and PTSD will not be met.

**ANTI-STIGMA INTERVENTIONS**
E. The article states that anti-stigma interventions specifically targeting healthcare workers, community leaders, and religious leaders are required to improve mental health literacy, understanding, and attitudes toward mental ill-health—explicitly including depression, anxiety, and PTSD. It describes how stigma acts as a critical barrier to care by discouraging patients from seeking treatment and providers from offering referrals for MHPSS. Therefore, sensitization campaigns that confront stigma directly are necessary to create an environment where individuals with mental ill-health feel safe accessing services.

**CULTURAL ADAPTATIONS**
F. The article explicitly recommends offering MHPSS services for mental ill-health—including depression, anxiety, and PTSD—in non-clinical settings such as community halls and schools. This recommendation is based on evidence that mental health services located in formal clinical settings are less acceptable and less accessible in many humanitarian contexts, partly due to stigma and partly due to logistical challenges. By situating services in familiar, community-based spaces, the likelihood of engagement by individuals with mental ill-health is increased, facilitating earlier access to support and treatment.

**COVID-19 Pandemic**

**SERVICE PROVISION**
A. The article explicitly states that the indirect impacts of the COVID-19 pandemic, such as lockdowns and mobility restrictions, have disrupted the management of mental ill-health—specifically depression, anxiety, and PTSD—by reducing access to lifestyle support, patient monitoring, and essential medicines. The lack of physical access to MHPSS services during the pandemic has exacerbated mental ill-health, particularly among vulnerable populations, as the restrictions have interrupted the delivery of care and isolated individuals from social and community support networks. The article describes these disruptions as further worsening the prognosis for mental ill-health and highlights the urgent need to maintain accessible services for depression, anxiety, and PTSD even during health emergencies.

**INTEGRATION OF CARE**
B. The article explicitly identifies the COVID-19 pandemic as an example of how treating mental ill-health in isolation from other health conditions results in fragmented, inefficient, and ultimately inadequate care for individuals with depression, anxiety, and PTSD. It emphasizes that the pandemic has highlighted the need for integrated care models that can simultaneously address mental health needs alongside other health services, especially during crises when systems are stretched. The failure to provide integrated care during COVID-19 has resulted in worsened mental health outcomes for patients, reinforcing the need for structural changes in humanitarian response frameworks to include mental ill-health—specifically depression, anxiety, and PTSD—within broader care strategies.

**Disease:** Chronic respiratory diseases

**For Humanitarian Settings (including conflict-affected areas and refugee contexts)**

**SERVICE PROVISION**
A. The article explicitly states that chronic respiratory diseases are part of the high-burden NCDs in humanitarian settings. It describes that healthcare systems in these settings operate in vertical, fragmented models, where care for chronic respiratory diseases is separated from other services such as mental health care. This separation is identified as a fundamental problem, as it leads to redundancy, inefficiency, and a lack of continuity in care, which directly impairs the ability of individuals with chronic respiratory diseases to receive consistent, integrated care. The fragmentation limits time and continuity of care, making it difficult for patients to access necessary services across different care points. The article highlights the necessity of addressing chronic respiratory diseases within a comprehensive service framework that responds to co-morbidities and complex needs in humanitarian settings, as the isolated treatment of chronic respiratory diseases fails to meet the needs of affected populations.

**PREPARATION OF STRATEGIC OR OPERATIONAL PLANS**
B. The article explicitly states that chronic respiratory diseases must be included in redesigned health system models that shift away from vertical, disease-specific programs towards integrated, patient-centered approaches. The article emphasizes that without such strategic or operational planning, services for chronic respiratory diseases remain underfunded and poorly coordinated, leading to gaps in care. It explains that fragmented care models in humanitarian settings cause redundancy, inefficiency, and time-consuming processes that disproportionately affect patients with chronic respiratory diseases, as these individuals often require long-term management and continuous access to services. Therefore, operational frameworks must prioritize the integration of chronic respiratory disease care with other services to ensure that these patients receive comprehensive support for their health needs.

**For COVID-19 Pandemic**

**SERVICE PROVISION**
A. The article explicitly states that during the COVID-19 pandemic, the management of chronic respiratory diseases was disrupted by lockdowns and restrictions that reduced access to lifestyle support, patient monitoring, and essential medicines. This disruption is described as having negatively affected the health of individuals with chronic respiratory diseases, as it led to decreased access to services that are critical for their care, including regular check-ups, medication provision, and support for disease management. The article highlights that patients with chronic respiratory diseases were particularly vulnerable to service interruptions because their conditions require consistent and sustained care, which was not maintained during the pandemic. The article uses the COVID-19 pandemic as a case example to emphasize the need for healthcare systems to ensure that access to services for chronic respiratory diseases is maintained during crises.

**INTEGRATION OF CARE**
B. The article explicitly identifies the COVID-19 pandemic as a demonstration of the failure of vertical, fragmented care models for chronic respiratory diseases. It states that the separation of chronic respiratory disease care from other health services leads to inefficiencies and worsens health outcomes during crises such as pandemics. The article emphasizes the need for integrated care models that include chronic respiratory diseases within broader health strategies, as these models are necessary to address both the immediate disruptions caused by crises and the long-term needs of patients with chronic respiratory diseases. By integrating chronic respiratory disease care into comprehensive service delivery frameworks, health systems can ensure that individuals with these conditions receive continuous and coordinated care even during emergencies such as the COVID-19 pandemic.

10. Article Title: Models of care for patients with hypertension and diabetes in humanitarian crises: a systematic review

Author, Publication year: Jaung, 2021

The article analyzes models of care across multiple types of humanitarian crises (e.g., conflicts and natural disasters) but does not consistently stratify strategies or measures by specific disaster types.

The article often refers to hypertension and diabetes care in combination (HTN/DM) and provides limited stratification of strategies specifically tailored to each disease.

- List of the NCD diseases considered in this article:

**Combination of Hypertension and diabetes**

- List all of the crises and disasters considered in this article

(Only non–inside health system crises like natural disasters, conflict, displacement)

- Main focus (included in analysis): **Conflict (e.g., Syrian war, Iraq conflict)., Displacement (e.g., refugees, IDPs), Natural disasters (e.g., Nepal earthquake, Sichuan earthquake, Pakistan Kashmir earthquake)**

**Disease:** Combination of Hypertension and diabetes

***DRUG SUPPLY***

**A.** The article reported that the United Nations Relief and Works Agency (UNRWA) systematically provided antihypertensive and diabetes medications, including insulin and oral hypoglycemic agents, across all its primary healthcare centers. These medications were delivered through standardized essential drug lists. Ensuring consistent availability was central to maintaining glycemic and blood pressure control in displaced populations. Medication supply was monitored via facility registers and logistics management systems to track stock levels and usage patterns. In one observational cohort, the availability of antihypertensive medications and insulin was cited as a factor enabling adherence to clinical guidelines and protocol-driven care.

**B.** In Médecins Sans Frontières (MSF) clinics operating in Lebanon, a tailored formulary included drugs for both hypertension and diabetes, specifically selected based on regional burden and treatment protocol alignment. Procurement adjustments were data-driven, reflecting the patterns of medication consumption. The clinics adapted supply strategies to match the influx of Syrian refugees with chronic conditions, emphasizing the procurement of insulin with cold chain capacity and antihypertensives based on treatment stage and patient profile.

***SERVICE PROVISION***

**A.** Hypertension and diabetes care in humanitarian settings was primarily delivered through physician-led, facility-based outpatient services. The majority of these services operated at the primary care level, where diagnosis, routine monitoring, and treatment were provided. For both diseases, the continuity of clinical contact was emphasized as essential, particularly in long-term refugee populations such as those served by UNRWA. Services were built to include regular assessments of blood pressure and fasting blood glucose levels.

**B.** After acute natural disasters (e.g., earthquakes in Nepal and Pakistan), mobile clinics included screening for hypertension and diabetes. While these services lacked continuity, they addressed the immediate need to identify and stabilize patients with elevated blood pressure or uncontrolled blood glucose. In one such setting, screening outcomes identified a high prevalence of undiagnosed hypertensive and diabetic cases, prompting referrals for further management.

**C.** In MSF clinics in Lebanon, diabetes and hypertension were treated within the same service model using a unified clinical pathway, reducing fragmentation and supporting efficiency in diagnosis, medication refill, and follow-up scheduling.

***SUPPORT***

**A.** UNRWA employed cohort monitoring systems to track individual-level data on blood pressure and glycemic control. These systems facilitated longitudinal tracking of patient adherence, clinical progress, and treatment effectiveness. For instance, patient registries recorded quarterly outcomes, which guided protocol adherence among healthcare workers and identified defaulters for follow-up interventions. This directly supported the stability of hypertension and diabetes control in settings of chronic displacement.

**B.** Behavioral support strategies were incorporated into select models, particularly in Palestinian refugee camps. These included structured education sessions targeting diet, physical activity, and medication adherence. Pilot programmes focused on self-care in diabetes showed a measurable increase in patient-reported adherence to dietary advice and exercise routines. In some facilities, these activities were reinforced by peer educators or community health workers.

***PREPARATION OF STRATEGIC OR OPERATIONAL PLANS***

**A.** Strategic planning for NCD integration into primary care was evident in UNRWA’s institutional policies, which specifically outlined standardized operational pathways for hypertension and diabetes management. These included scheduled follow-up visits every three months, clinical target benchmarks (e.g., BP <140/90 mmHg, FBG <130 mg/dL), and defined protocols for referral when complications emerged. The plans also accounted for the maintenance of service delivery during crisis escalation and ensured that displaced populations had continuous access to chronic disease management.

***TRAINING OF FORCES***

**A.** General practitioners and nurses across UNRWA health centers received focused training modules on the clinical management of hypertension and diabetes. The curriculum covered diagnostic thresholds, pharmacologic treatment algorithms, risk stratification, and lifestyle counselling. Training also emphasized the use of standardized documentation tools to support the continuity and safety of care.

**B.** In MSF operations in Jordan and Lebanon, healthcare workers were trained to implement algorithm-based protocols for diagnosing and managing both conditions. Emphasis was placed on the uniform application of clinical guidelines to mitigate the impact of workforce turnover and ensure consistency in care across facilities.

***CREATION OF STORAGE RESOURCES***

**A.** Cold chain systems were established for insulin storage in several contexts. These included both fixed-site clinics and mobile outreach missions. In Lebanon, diabetes programs operated by MSF specifically maintained refrigeration capacity for insulin preservation under crisis conditions. Parallel systems for oral drugs such as antihypertensives were tracked through stock registers and reorder threshold systems.

***IMPROVEMENT OF INTER-SECTORAL COMMUNICATIONS***

**A.** Coordination between humanitarian NGOs (e.g., MSF, UNRWA) and host country Ministries of Health facilitated integration of hypertension and diabetes services into existing national health frameworks. This allowed continuity of care, especially when patients transitioned between NGO-supported and public facilities. For example, shared clinical protocols and interoperable medical record systems supported cross-sectoral communication about treatment history and clinical targets.

***PROVISION OF BUDGET***

**A.** Both hypertension and diabetes service provision under UNRWA was supported by designated budget lines funded by international donors. These budget allocations covered the procurement of medications, laboratory supplies, and staff salaries. The article noted that this financial structure enabled scale-up and continuity of NCD care despite recurrent funding challenges inherent in humanitarian contexts.

***OTHER MEASURES***

**A.** Structured patient-level data systems were implemented in UNRWA settings to monitor outcomes for hypertension and diabetes. For hypertension, this included periodic BP measurements logged into individual patient charts; for diabetes, fasting blood glucose levels and HbA1c values (where available) were similarly tracked. These metrics were used to evaluate program effectiveness and inform decisions on therapy adjustments.

**B.** In some programs, such as those operated by MSF in Lebanon, clinical differentiation between Type 1 and Type 2 diabetes guided treatment allocation. Insulin-dependent patients were flagged for more frequent follow-up and laboratory testing. In contrast, patients with Type 2 diabetes managed with oral agents were monitored at longer intervals unless complications arose.

11. Article Title: The Impact of Disasters on Cancer Care in Lebanon

Author, Publication year: El Karak, 2021

the article discusses a combination of disasters — economic crisis, COVID-19 pandemic, and Beirut explosion — and their impact on cancer care without stratifying strategies or measures by the specific type of disaster.

the article discusses “cancer” as a general category of NCD without distinguishing strategies by specific cancer types.

- List of the NCD diseases considered in this article:

**Cancer**

- List all of the crises and disasters considered in this article

(Only non–inside health system crises like natural disasters, conflict, displacement)

Main focus (included in analysis): Economic crisis, COVID-19 pandemic, Beirut port explosion (natural/manmade disaster)

***Disease:*** *Cancer*

***DRUG SUPPLY***

A. Cancer patients in Lebanon experienced a critical decline in access to anticancer therapies due to multiple factors, including the economic collapse, the destruction of medical storage facilities, and institutional damage. Specifically, patients lost access to novel and expensive therapies and were consequently forced to rely on older, less effective chemotherapeutic protocols. This regression in therapeutic quality was a direct result of the national currency devaluation, which limited drug importation and reduced the purchasing power of both individuals and institutions.

B. The destruction of the governmental hospital of Quarantina, a central distribution point for free cancer medications, directly interrupted the pharmaceutical supply for approximately half of Lebanon’s cancer patient population. This hospital was rendered nonfunctional after the Beirut explosion, halting drug delivery for several days and thereby exacerbating delays in treatment.

C. A large stockpile of cancer medications stored near the site of the Beirut port explosion was destroyed, further reducing the already fragile availability of essential oncology drugs and rendering many treatment protocols temporarily inaccessible. This compounded pre-existing shortages caused by the economic crisis.

***SERVICE PROVISION***

A. The economic collapse led to a substantial increase in the cost of complementary cancer-related services including diagnostic imaging, laboratory tests, hospitalizations, and specialist consultations. These additional expenses, occurring in the context of widespread unemployment (with a reported 40% rate in 2020), made basic elements of cancer diagnosis and management unattainable for many patients, thereby delaying or interrupting ongoing care.

B. Several major hospitals in Beirut, including three that were completely destroyed and three more that were significantly damaged, were forced to shut down or reduce operations. Since the majority of cancer patients in Lebanon are treated in Beirut, this infrastructural collapse substantially impaired access to oncology units, especially chemotherapy services.

C. As a direct consequence of these infrastructural damages, cancer patients were compelled to transfer to other hospitals, often located farther away and with limited capacity. This created treatment delays, missed chemotherapy sessions, and lapses in continuity of care—particularly significant for conditions such as cancer, where strict treatment schedules are clinically critical.

D. During the COVID-19 pandemic, scheduled screening procedures such as mammograms and colonoscopies were postponed, and elective surgeries were delayed due to concerns over viral transmission. This resulted in a marked decrease in new cancer diagnoses, which the article notes is likely to lead to an increase in advanced-stage cancer presentations in the future.

***SUPPORT***

A. International humanitarian organizations provided limited assistance for cancer care, citing financial constraints, prioritization of acute trauma care, and high treatment costs. Consequently, only a minimal number of cancer patients received medication donations or care abroad, and oncology-specific needs remained largely unmet.

B. Some forms of support were eventually provided in the aftermath of the Beirut explosion, such as external donations of cancer drugs and hospital supplies, as well as funding aimed at restoring or relocating oncology departments. However, the article emphasizes that these interventions were insufficient in scale relative to the demand.

***PREPARATION OF STRATEGIC OR OPERATIONAL PLANS***

A. WHO collaborated with the Lebanese Ministry of Public Health to formulate a strategic plan under the Beirut Port Blast Emergency Strategic Response Plan. The goal was to coordinate the rehabilitation of damaged medical facilities and secure consistent access to medication, including for oncology patients. However, the article cautions that economic barriers may delay implementation and limit its effectiveness.

***TRAINING OF FORCES***

A. The article asserts the necessity of training rapid-response teams in essential noncommunicable disease interventions, specifically emphasizing the inclusion of cancer treatments and medications in emergency kits. This recommendation is based on the observed lack of preparedness and the disproportionate impact on cancer patients during acute disasters.

***PROVISION OF BUDGET***

A. As a result of economic constraints, both public and private healthcare institutions—including third-party payers and hospitals—discontinued approval and provision of high-cost cancer therapies, such as immunotherapy and targeted agents. Physicians were consequently forced to revert to more affordable but less effective chemotherapy regimens, which compromised the quality of care delivered to patients.

***IMPROVEMENT OF INTER-SECTORAL COMMUNICATIONS***

A. The article highlights collaborative efforts between WHO and the Ministry of Public Health but does not elaborate detailed mechanisms of inter-sectoral communication or protocols. Nonetheless, it implies that effective coordination was necessary but largely insufficient, as demonstrated by delays and inconsistencies in drug delivery and service recovery.

***OTHER***

A. Physicians were instructed to equip cancer patients with a personalized treatment plan that anticipates interruptions. This plan included a drug list for managing side effects and a concise case summary to facilitate care in alternative facilities in the event of translocation.

B. Patients were advised to become informed about their disease, its vulnerability during crises, the availability of specific medications, and infection prevention measures for COVID-19. For patients unable to manage these tasks, caregivers were expected to assume these responsibilities.

C. Both healthcare providers and family members were expected to deliver psychosocial support during traumatic events, with explicit prioritization of pharmaceutical needs, followed by dietary, supportive, and physiotherapeutic requirements.

12. Article Title: Chronic NCD care in crises: A qualitative study of global experts’ perspectives on models of care for hypertension and diabetes in humanitarian settings

Author, Publication year: Ansbro, 2022

The article discusses humanitarian crises broadly (conflict, natural disaster, food insecurity) and provides a general model of response without strictly stratifying strategies or measures by specific disaster types.

The article addresses both Hypertension and Diabetes Mellitus as a combined NCD care model (often referred to as HTN/DM care), and it does not provide separate strategies for each disease type.

- List of the NCD diseases considered in this article:

**Combination of Diabetes Mellitus and Hypertension**

- List all of the crises and disasters considered in this article

(Only non–inside health system crises like natural disasters, conflict, displacement)

- Main focus (included in analysis): **Conflict, Natural Disaster, Food Insecurity, Displacement, Humanitarian Crisis (general), Protracted Crisis**

**DISEASE:** Diabetes Mellitus

***DRUG SUPPLY***
A. Insulin availability was limited due to short national supplies, cold chain constraints, and regulatory restrictions, particularly where insulin prescription was limited to specialists. This meant individuals with Type 1 Diabetes Mellitus, who are insulin-dependent, could not maintain glycemic control during crises. Without sustained access to insulin, acute complications such as diabetic ketoacidosis occurred, which increased mortality risk in the acute phase of emergencies. NGOs tried to mitigate this by using WHO-recommended generic human insulin to reduce costs and dependence on high-tech analogues, which were not feasible in unstable settings.
B. Interruptions in insulin supply were common in public health facilities, where insulin was only available for 10 days in some cases. Patients had to purchase the remainder at personal expense, which was often unaffordable and resulted in medication non-adherence and severe disease progression.
C. Supply chain issues were exacerbated by inadequate national procurement systems, delayed import processes, and the lack of WHO-prequalified insulin products, which would facilitate streamlined global procurement.
D. Cold chain limitations, particularly in low-resource or mobile crisis contexts, further obstructed insulin distribution. Suggested solutions included building on existing vaccine cold chain infrastructure and revising national policies to allow primary-level prescribing and administration.

***SERVICE PROVISION***
A. In acute emergencies, persons with Type 1 Diabetes were prioritized for care due to high mortality risk from insulin deprivation. Humanitarian responses attempted to provide emergency stabilization, insulin access, and identification of patients at risk of death.
B. In early recovery and protracted crises, the models of care transitioned to continuity management, including medication adherence and prevention of complications such as neuropathy or renal failure. However, access to specialist services, such as endocrinology or ophthalmology, was extremely limited or non-existent, which rendered routine screening for complications ethically questionable.
C. Services were primarily provided at the primary care level, aiming to increase geographic and cultural proximity to affected populations. Patient familiarity with staff and community-level presence enhanced attendance, yet lacked adequate referral pathways for advanced care needs.

***TRAINING OF FORCES***
A. Primary care providers lacked adequate training in insulin initiation and diabetes complication management. Most programs emphasized that existing staff had minimal exposure to diabetes protocols, especially in unstable settings, necessitating intensive capacity building.
B. Training was not simply a one-time session; ongoing supervision and practical support were identified as essential, particularly where health worker turnover was high.
C. Task-sharing to nurses and mid-level workers was recommended. For example, nurses could be trained to measure blood glucose, recognize signs of hyperglycemia, provide dietary counseling, and assist with insulin titration. However, this was not universally implemented due to national restrictions and professional resistance.

***STRATEGIC OR OPERATIONAL PLANS***
A. Standardized treatment protocols for diabetes were absent or inconsistently used across humanitarian actors. There was a demand for comprehensive operational guidelines specifying medicine lists, consultation frequency, clinical targets (e.g., HbA1c thresholds), and indicators for monitoring outcomes.
B. Emergency response kits lacked insulin and related diabetes supplies, indicating that diabetes was historically excluded from core emergency planning tools. This exclusion delayed the integration of diabetes services in the acute phase of crisis.

***CREATION OF STORAGE RESOURCES***
A. Cold storage infrastructure was underdeveloped in many humanitarian settings, which compromised the viability of insulin. Without temperature-controlled supply chains, insulin could degrade, becoming ineffective or dangerous. To address this, participants proposed utilizing existing immunization cold chains and advocating for context-adapted insulin storage solutions.

***BUDGET PROVISION***
A. Insulin costs consumed up to 50% of some program budgets. Humanitarian organizations perceived this as unsustainable under short-term funding models typical of emergency responses. This financial strain led to restricted eligibility criteria for insulin access and limited medication stockpiling.
B. Participants demanded new funding structures, akin to disease-specific funds like the Global Fund for HIV, to ensure consistent financing for diabetes care during crises.

***INFORMATION SYSTEMS***
A. Patient-held paper or electronic clinical records were essential for maintaining continuity of care among displaced persons. These tools allowed patients to retain their treatment history and dosage details, compensating for fragmented health system records.
B. Lack of standardized data collection systems hindered monitoring of diabetes control indicators, such as blood glucose levels or HbA1c. This impaired quality control and evaluation of care effectiveness.

***COMMUNITY AND PATIENT SUPPORT***
A. Community education on diabetes symptoms, lifestyle modification, and self-care was limited but emphasized as crucial. Many populations had low literacy or minimal understanding of diabetes, which impacted treatment adherence and dietary behavior.
B. Peer support structures, modeled after HIV adherence groups, were proposed to increase patient engagement. These included community cooking and physical activity sessions tailored to diabetics, which saw high levels of participation in pilot programs.

**DISEASE:** Hypertension

***DRUG SUPPLY***
A. The supply of antihypertensive drugs was often inadequate due to poor forecasting, procurement delays, and lack of consistent delivery. Patients frequently faced treatment interruptions, leading to uncontrolled blood pressure and elevated risk for stroke or myocardial infarction.
B. NGOs used older, generic antihypertensive agents to reduce costs, although effectiveness and patient adherence varied. These included medications from the WHO Essential Medicines List, such as hydrochlorothiazide and enalapril.
C. Patients were sometimes forced to buy medications from private markets when public systems lacked supply, creating a financial barrier that contributed to poor adherence and exacerbation of disease.

***SERVICE PROVISION***
A. Hypertension was generally not prioritized in acute emergencies unless combined with high-risk conditions. However, in early recovery and protracted crises, hypertension care was integrated into chronic disease packages.
B. These models focused on risk assessment, medication adherence, and lifestyle counseling to prevent long-term complications such as stroke and renal failure. Nonetheless, access to specialist services remained very limited.
C. Primary care settings delivered most hypertension services due to better accessibility, but lacked tools for advanced management and continuous monitoring.

***TRAINING OF FORCES***
A. Many health workers were not familiar with hypertension treatment guidelines, including use of combination therapy or cardiovascular risk stratification.
B. Clinical training was needed on blood pressure measurement techniques, identification of hypertensive urgency, and management algorithms.
C. Task-sharing was recommended: nurses and community health workers could conduct routine blood pressure checks, provide lifestyle advice, and monitor treatment adherence, thereby relieving pressure on physicians.

***STRATEGIC OR OPERATIONAL PLANS***
A. Clinical guidelines were either lacking or inconsistently applied, creating variability in treatment targets and follow-up practices. This undermined care standardization and monitoring.
B. Tools such as WHO's HEARTS package were referenced, but their operational deployment in humanitarian settings remained limited.

***BUDGET PROVISION***
A. Although not as costly as diabetes treatment, hypertension programs still faced underfunding, particularly for diagnostics and combination therapies.
B. Short-term project funding led to disruptions in services and made it difficult to establish long-term control strategies for chronic hypertension.

***INFORMATION SYSTEMS***
A. Patient records were inconsistent, impeding longitudinal blood pressure monitoring and evaluation of treatment efficacy.
B. Integrated monitoring systems for hypertension were recommended, including cohort reporting tools and routine analysis of indicators such as BP control rates.

***COMMUNITY AND PATIENT SUPPORT***
A. Lifestyle interventions such as dietary modification and increased physical activity were essential but under-implemented.
B. Peer support, similar to HIV models, was suggested to enhance adherence, but had not been systematically introduced in most crisis contexts.

13. Article Title: Strengthening Diabetes Care in Humanitarian Crises in Low- and Middle-income Settings

Author, Publication year: Kehlenbrink, 2022

the article discusses humanitarian crises generally (e.g., conflicts, displacement, and natural disasters) without consistently stratifying measures by specific disaster types.

- List of the NCD diseases considered in this article:

**Diabetes Mellitus, Mental Health Disorders (e.g., depression, PTSD, anxiety, in the context of NCD comorbidity)**

- List all of the crises and disasters considered in this article

(Only non–inside health system crises like natural disasters, conflict, displacement)

Main focus (included in analysis): **Armed conflict (e.g., Ukraine, Mali, Central African Republic), Natural disasters (implied but not specified individually), Humanitarian crises, COVID-19 pandemic, Forced displacement/refugee crises**

**Disease:** Diabetes Mellitus

**Crisis Type: All Humanitarian Crises (Combined)**

***DRUG SUPPLY***

A. The availability of insulin and other essential diabetes medications in crisis-affected settings was described as inconsistent, primarily due to disrupted or weak supply chains. For example, insulin, which is essential for individuals with type 1 diabetes and some with type 2 diabetes, requires a reliable cold chain system that is frequently unavailable in low-resource crisis environments. This often results in treatment interruptions, which can lead to acute complications such as diabetic ketoacidosis, a life-threatening condition. To address this, deployment of the NCD-IEHK (Non-Communicable Disease – Interagency Emergency Health Kit) was proposed. This kit includes oral hypoglycemics, human insulin, and consumables such as syringes and glucose strips, all of which are necessary to manage diabetes effectively in humanitarian settings.

B. The WHO highlighted that many health facilities do not provide insulin during emergencies, particularly in the initial phases, which compromises the survival of insulin-dependent individuals. For instance, during the Mali crisis, the emergency kits were distributed every three months to ensure uninterrupted insulin availability in occupied regions. This action directly led to the survival of all evacuated children with type 1 diabetes, emphasizing the criticality of continuous medication supply.

C. In Ukraine, the sudden onset of conflict led to disruptions in the medication distribution system. In response, donated insulin became the primary supply source, although it introduced unfamiliar insulin formulations. This necessitated the rapid creation and translation of insulin switching guides to enable safe and effective use, thereby preventing mismanagement or harmful dosing errors.

***SERVICE PROVISION***

A. Integration of diabetes care into primary healthcare systems is vital in humanitarian crises to decentralize services, particularly when secondary facilities are overwhelmed. For instance, in Jordan, diabetes services were initially offered through vertical programs but later integrated into primary care structures to ensure broader access for Syrian refugees. This adaptation enabled individuals with diabetes to receive comprehensive care, including for acute infections, thereby reducing fragmentation of services.

B. The article emphasizes that displaced populations often live in urban settings and are not automatically integrated into national health systems. By integrating them early through the Ministry of Health, continuity of diabetes care can be maintained. Without this, people with diabetes face service discontinuities that exacerbate glycemic instability.

C. Differentiated service delivery models allow for flexible approaches tailored to context-specific needs, such as comorbidities (e.g., diabetes with mental illness), host vs. refugee status, or logistical challenges. These models help streamline services and ensure patients receive necessary interventions, avoiding both under- and over-treatment.

***SUPPORT***

A. Community Health Workers (CHWs) and Medication Access Groups were employed in conflict-affected areas such as the Central African Republic. These groups ensured monthly collection of medications by one designated individual, reducing risk to others and maintaining access to diabetes treatment. This model demonstrated consistent medication access with disruptions lasting less than two months over several years, significantly benefiting patients with diabetes reliant on continuous pharmacologic therapy.

B. During the COVID-19 pandemic, adaptations such as 90-day medication dispensing, telehealth services, and CHW support were implemented. These changes reduced the frequency of visits to health facilities, thereby minimizing exposure risks and ensuring that people with diabetes maintained medication adherence and glycemic control.

C. Investment in patient education was emphasized to promote self-management, particularly in insecure environments where access to providers is limited. This includes training patients on how to recognize signs of hypo- and hyperglycemia and adjust medication or diet accordingly. Such empowerment mitigates risks during service gaps and sustains health outcomes.

***STRATEGIC OR OPERATIONAL PLANS***

A. In response to treatment discontinuities in crises, the WHO passed a global resolution in 2021 mandating the development of guidance to ensure uninterrupted diabetes care in emergencies. This strategic action reflects the high global burden of diabetes and aims to standardize emergency response plans across nations and organizations.

B. A Ministry of Health hotline was established in Ukraine to inform individuals with diabetes about where to obtain insulin. This communication measure facilitated real-time responses to evolving crises, reducing uncertainty and improving access to life-saving therapy.

***TRAINING OF FORCES***

A. Given the shortage of physicians and diabetes-trained professionals during crises, task-sharing with nonphysician providers such as nurses was advocated. Practical adaptations included simplified algorithms for managing acute complications like diabetic ketoacidosis, which could be implemented even without access to full laboratory diagnostics. These measures ensure that critical care is not delayed due to workforce limitations.

B. In Jordan, the rapid expansion of diabetes care at the primary level necessitated the training of generalist providers in chronic disease management. Training was not limited to pharmacotherapy but included patient communication and health promotion, ensuring more holistic care for displaced individuals living with diabetes.

***STORAGE RESOURCES***

A. Insulin typically requires refrigeration, which is often unavailable in crises. However, the article reports that insulin retains efficacy at temperatures up to 37°C for four weeks, as demonstrated in a study from Dagahaley Refugee Camp. Practical techniques such as clay pot storage were promoted to keep insulin within this temperature range, enabling safe use even in extreme environments.

***IMPROVEMENT OF INTER-SECTORAL COMMUNICATIONS***

A. Coordinated actions between health ministries, NGOs, and pharmaceutical firms were crucial in Ukraine for the distribution of donated insulin amidst conflict. Such collaborations enabled the rerouting of supply chains, minimized waste, and ensured delivery to facilities despite infrastructure damage, directly preserving the lives of people dependent on daily insulin.

***PROVISION OF BUDGET***

A. High out-of-pocket costs for diabetes care—including medications, diagnostics, and transportation—were repeatedly identified as barriers to access in humanitarian settings. The article emphasizes the need for policy advocacy to suspend user fees during crises and to ensure that displaced populations are granted access to free care, preventing treatment interruption and adverse outcomes.

B. Recommendations were made to adopt pooled procurement, prioritize use of generics, and explore biosimilar insulins to reduce costs. Although specific cost data were limited, such financial strategies were aligned with successful models in HIV/TB programs and are considered feasible for diabetes care scale-up.

***OTHER***

A. Traditional food assistance in emergencies often relies on high-carbohydrate, low-nutrient baskets, which are incompatible with diabetes dietary needs. The article highlights the shift toward “double-duty” nutrition actions that support both undernutrition and NCDs. This includes diversifying food aid, supporting dietary education, and involving communities in livelihood programs to stabilize diabetes outcomes.

B. Mental health comorbidities such as depression and PTSD are highly prevalent in crisis settings and can significantly impair diabetes self-management. The article reports that integrated care programs addressing both diabetes and mental health improved clinical outcomes (e.g., HbA1c and blood pressure) among Syrian refugees in Jordan, validating the necessity of including psychosocial components in diabetes interventions.

C. Cash transfer programs were associated with improved treatment adherence and diabetes control. In Jordan, longitudinal data from such interventions showed positive effects on health service utilization and glycemic outcomes among displaced diabetes patients, underlining their utility as a targeted financial mechanism.

**Disease:** Mental Health Disorders (e.g., depression, PTSD, anxiety, in the context of NCD comorbidity)
**Crisis Type: All Humanitarian Crises (Combined)**

***SERVICE PROVISION***

A. The article states that humanitarian standards require routine integration of mental health and psychosocial support (MHPSS) into health programs, yet in practice, MHPSS is often delivered separately from primary care and not incorporated into NCD programs. This separation creates a fragmentation in care that neglects the strong bidirectional relationship between mental disorders and NCDs. For example, untreated depression impairs self-management behaviors necessary for controlling chronic diseases, while the burden of chronic illness can worsen mental health. The lack of integration delays identification and treatment of mental disorders, leading to worse health outcomes in NCD patients.

B. A program implemented among Syrian refugees in Jordan integrated physical and mental health care into a single package. This model led to measurable improvements in clinical outcomes, including reductions in blood pressure and HbA1c levels, among patients receiving both types of care. These results demonstrate that when mental health services are embedded within broader chronic disease management, patients are more likely to achieve control of both physical and mental health conditions. The article attributes these outcomes to reduced psychological barriers, improved treatment adherence, and strengthened trust in the healthcare system.

C. The Jordan case study describes how mental health services were embedded into a comprehensive primary care package along with NCD management and services for sexual- and gender-based violence. This integration was initiated in response to the high prevalence of psychological distress in the refugee population. The decision to provide these services at the primary care level allowed simultaneous treatment of comorbid mental and physical conditions, addressing the overlapping burden of disease more efficiently and reducing access barriers that patients with mental disorders often face.

***SUPPORT***

A. The article identifies family, peer, and health care team support as critical components in the management of mental health disorders in humanitarian settings. In the context of comorbid NCDs, depression and anxiety commonly impair motivation and executive functioning, which leads to reduced adherence to treatment plans and self-care regimens. Support systems compensate for these functional deficits by reinforcing adherence behaviors, offering reminders, and reducing social isolation. These forms of psychosocial reinforcement are essential to counteract the behavioral manifestations of mental disorders that directly worsen NCD outcomes.

***STRATEGIC OR OPERATIONAL PLANS***

A. The article emphasizes the need for targeted investment in operational guidelines and implementation strategies for the integration of MHPSS within NCD programs. This need arises from the recognition that mental health remains a neglected component of emergency health responses. Without formalized guidance and designated funding, MHPSS is inconsistently prioritized and frequently under-resourced. The lack of structured integration leaves patients with untreated psychological symptoms that exacerbate chronic disease progression, increase disability, and lead to recurrent healthcare utilization.

***TRAINING OF FORCES***

A. In Jordan, social workers were recruited and placed at the primary care level to assist patients in navigating health and psychosocial systems. Their role extended to connecting individuals with mental health disorders to appropriate support services, particularly in cases where patients faced complex psychosocial vulnerabilities such as trauma, displacement, or stigmatization. By facilitating access to mental health care and other social services, these social workers mitigated barriers linked to psychological conditions, including avoidance behavior, distrust in health providers, or inability to self-advocate. This direct linkage between health and psychosocial service delivery is critical in humanitarian contexts, where mental health infrastructure is often fragmented or inaccessible.

***OTHER***

A. The article outlines how exposure to conflict-related violence, family separation, poverty, and forced displacement contributes to the development or worsening of mental health conditions such as PTSD, anxiety, and depression. These stressors not only trigger mental disorders but also compound the difficulty of managing comorbid chronic conditions. The psychological effects of trauma interfere with self-management, reduce treatment adherence, and exacerbate metabolic and cardiovascular risks. This creates a feedback loop where mental illness undermines physical health, and unmanaged chronic illness worsens psychological distress. The article argues that addressing these determinants within care strategies is essential to stabilize mental health and break this cycle.

14. Article Title: Diabetes Care during Humanitarian Crises due to Floodings and Earthquakes in IDF-MENA Region: Pakistan Experience

Author, Publication year: Odhaib, 2022

The article discusses both the 2005 earthquake and the 2022 floodings in a combined humanitarian crisis context, but it does not stratify the strategies or measures by disaster type.

- List of the NCD diseases considered in this article:

**Diabetes Mellitus, Hypertension, Cardiovascular Disease, Acute Kidney Injury (related to dialysis service in 2005 earthquake context)**

- List all of the crises and disasters considered in this article

(Only non–inside health system crises like natural disasters, conflict, displacement)

- Main focus (included in analysis): **Earthquake, Flooding**

**Disease:** Diabetes Mellitus

**Crisis: Earthquake**

***DRUG SUPPLY***

A. During the 2005 earthquake in Azad Jammu and Kashmir, diabetes care supplies, particularly insulin and oral hypoglycemic agents, were depleted rapidly and not replenished effectively. This deficiency severely limited the capacity to meet the urgent health needs of diabetic individuals. The exhaustion of supplies was especially detrimental for elderly victims residing in rural areas, who already faced access barriers to specialized care. The inability to maintain glycemic control due to disrupted pharmacologic therapy increased the risk of acute metabolic complications such as diabetic ketoacidosis and hyperosmolar hyperglycemic states.

***SERVICE PROVISION***

B. In the aftermath of the earthquake, mobile health service clinics provided basic care for diabetic patients. However, these services did not adhere to any standardized diabetes treatment protocols. This lack of clinical guidelines in service provision created inconsistencies in care quality, which could lead to suboptimal glycemic control and increased risk of diabetes-related complications. The reliance on these clinics for several months following the disaster indicated the collapse of the formal health infrastructure for chronic disease management in the region.

***SUPPORT***

C. A joint public health initiative was launched by the ICRC and the Department of Health in Azad Jammu and Kashmir to reduce preventable diabetes-related amputations, which represented 10% of all amputations in the region. This intervention aimed to mitigate severe diabetes complications by promoting early detection and timely referral.
D. Female healthcare workers were trained specifically to identify early signs of diabetic foot disease. This was implemented to counteract gender inequity in access to care, as cultural and logistical barriers often restricted women’s interactions with male healthcare professionals. These female workers operated at the community level, thereby increasing the reach and timeliness of clinical interventions for diabetic foot conditions.
E. A teleconsultation and doorstep delivery program was introduced during the COVID-19 pandemic to continue monitoring and managing diabetic patients. Approximately 1,800 patients in Azad Jammu and Kashmir and nearby areas received diabetes services directly at their homes. This strategy helped prevent deterioration of glycemic control in a crisis setting where physical access to care was severely limited.

***TRAINING OF FORCES***

F. Healthcare workers in local Basic Health Units received targeted training to manage non-emergent diabetic foot injuries. They were provided with essential medical supplies and instructed on specific clinical protocols for foot care, wound management, and infection control. The focus on equipping peripheral health facilities with both knowledge and materials aimed to decentralize diabetic care and prevent avoidable amputations during health system disruptions.

***STRATEGIC OR OPERATIONAL PLANS***

G. The article explicitly emphasized the inadequacy of existing disaster preparedness frameworks in integrating diabetes management. It called for a reformulation of national health emergency policies to include noncommunicable disease care, specifically diabetes, as a core component. The absence of context-adapted and resilient operational strategies for diabetic care during the 2005 earthquake was highlighted as a major gap that contributed to increased morbidity.

***CREATION OF STORAGE RESOURCES***

H. The authors recommended the preparation of waterproof and insulated disaster kits for diabetic individuals. These kits should include glucose monitoring tools, insulin, syringes, oral medications, antibiotic ointments, prepackaged snacks, and printed prescriptions. The rationale was to ensure immediate self-management capacity during disaster-induced supply chain disruptions. The lack of such resources in the 2005 disaster led to treatment discontinuity and life-threatening dysglycemic events.

***TRAINING AND EDUCATION OF PEOPLE WITH DIABETES***

I. Patients with diabetes and their family members were advised to be trained in self-management skills. This includes recognition of hypo/hyperglycemic symptoms, adherence to medication schedules, foot inspection, and safe storage of insulin. Such knowledge was deemed essential for maintaining clinical stability in an environment where professional support was temporarily inaccessible due to the disaster.

***OTHER***

J. The article discussed that large-scale natural disasters, such as earthquakes, can lead to psycho-mental consequences like post-traumatic stress disorder, which can worsen glycemic control. The multifactorial interaction of neurohormonal stress, disruption in physical activity, nutritional insecurity, and emotional trauma was associated with higher rates of morbidity and mortality in diabetic patients.

**Crisis: Flooding**

***DRUG SUPPLY***

A. The 2022 flooding in Pakistan led to a disruption in the availability of insulin and oral antidiabetic medications. The article explained that this break in pharmacological treatment caused heightened anxiety among diabetic individuals and severely impeded their ability to maintain therapeutic glycemic levels. The consequences included potential for acute complications and an increased burden on already limited emergency services.

***SERVICE PROVISION***

B. During the flood response, Médecins Sans Frontières deployed mobile healthcare clinics in several affected regions. Although these clinics offered limited outpatient services, diabetes care was not systematically included. The lack of disease-specific care in these service provisions left diabetic individuals without proper follow-up, monitoring, or treatment adjustment capabilities during the acute crisis.

***SUPPORT***

C. The article noted that most NGO efforts during the flood focused on infectious diseases. Support for chronic disease management, including diabetes, was minimal. This lack of targeted assistance created gaps in continuity of care, especially in underserved areas where formal health infrastructure was compromised by the disaster.

***STRATEGIC OR OPERATIONAL PLANS***

D. Existing disaster response plans were found to be insufficient in addressing the needs of diabetic populations during the floods. The article advocated for the incorporation of diabetes care as an integral part of operational health emergency frameworks, citing the failures in 2022 as evidence of ongoing systemic neglect.

***CREATION OF STORAGE RESOURCES***

E. The article provided detailed guidance on the contents and utility of an insulated disaster kit for individuals with diabetes. Such a kit should include a glucometer, glucose test strips, extra batteries, lancets, insulin with long expiration dates, syringes, glucose tablets, and dry nonperishable food. The aim was to ensure therapeutic continuity when access to pharmacies or clinics was impeded by floods. Prescriptions should be kept in waterproof formats.

***TRAINING AND EDUCATION OF PEOPLE WITH DIABETES***

F. Diabetic individuals were instructed not to walk barefoot, to wear sturdy shoes, and to inspect their feet daily for blisters or ulcers to prevent foot injuries in flood conditions.
G. They were also advised to avoid hunger and overeating, maintain hydration, and wear light-colored cotton clothing to manage thermal stress. They should retain all prescriptions and information about their medications, including dosing and formulation, in waterproof packaging for emergencies.

***OTHER***

H. The article explained that carbohydrate-heavy relief food distributed by aid agencies posed risks to glycemic control in diabetic individuals. These rations lacked dietary consideration for chronic disease patients, leading to dysglycemia.
I. Factors such as hunger, psychological stress, therapy discontinuation, lack of diverse food options, and fear were identified as primary contributors to destabilized glycemic control during the floods.
J. It was noted that insulin storage posed a significant challenge in flood conditions. The article outlined stability timelines for different types of insulin when unrefrigerated: up to 28 days for regular short-acting insulin, 14 days for NPH pens, 10 days for premixed insulin pens, and 42 days for Detemir pens.

**Disease:** Hypertension

**Crisis: Earthquake**

***STRATEGIC OR OPERATIONAL PLANS***

A. The article explicitly stated that during the 2005 earthquake in Azad Jammu and Kashmir, governmental and nongovernmental agencies lacked clear, context-adapted care models for individuals with noncommunicable diseases such as hypertension. The absence of strategic planning for chronic conditions resulted in the exclusion of hypertensive care from emergency response frameworks. This failure increased the risk of uncontrolled blood pressure due to the lack of systematic monitoring, timely medication administration, and continuity in care. The authors emphasized that the global target of reducing mortality from noncommunicable diseases by one-third is unattainable in settings where healthcare policies do not address hypertension during crises. No strategic blueprint existed to manage hypertensive emergencies or prevent long-term cardiovascular complications in disaster-affected populations.

***SUPPORT***

B. The article stated that the response of international aid organizations after the earthquake was focused on acute trauma, infectious disease control, mental health services, and care for the elderly and orphans. Hypertension care was not included in their priorities. This lack of support created a systemic gap in addressing elevated cardiovascular risk during the acute and post-acute phases of the earthquake. In hypertensive individuals, interruption of antihypertensive therapy and absence of monitoring significantly increases the likelihood of complications such as stroke, myocardial infarction, and hypertensive encephalopathy, none of which were accounted for by the aid interventions described.

***OTHER***

C. The article described hypertensive patients as part of the broader group of vulnerable individuals who experienced adverse health outcomes due to dysfunctional healthcare infrastructure following the earthquake. The destruction of health facilities and shortages of healthcare professionals led to an inability to provide routine services such as blood pressure monitoring and medication dispensing. In the absence of these core components of care, hypertensive individuals were placed at elevated risk for acute exacerbations and progression to end-organ damage, as there were no contingency systems in place to absorb the clinical burden in the wake of infrastructure collapse.

**Crisis: Flooding**

***STRATEGIC OR OPERATIONAL PLANS***

A. The article reported that during the 2022 floods, healthcare policies and disaster response mechanisms failed to incorporate the care needs of hypertensive individuals. The crisis's severity and unpredictability overwhelmed already insufficient planning models, which lacked any provision for noncommunicable disease continuity. The authors clearly stated that preparedness plans for both disaster and non-disaster settings were unsatisfactory in meeting the basic health needs of people living with hypertension. Consequently, the management of elevated blood pressure was disrupted, increasing the potential for hypertensive crises and preventable cardiovascular deaths.

***SUPPORT***

B. The article mentioned that aid efforts in the 2022 flood response primarily targeted infectious diseases, with minimal attention to chronic diseases including hypertension. The failure to provide support for antihypertensive care exposed patients to uncontrolled blood pressure due to stress, disrupted sleep, restricted mobility, poor nutrition, and lack of medication. Without dedicated clinical or logistical support for this population, the risk of acute events such as hypertensive emergencies remained unmitigated.

***OTHER***

C. The article classified individuals with hypertension as part of the vulnerable population most affected by the flood-induced collapse of healthcare access. Specifically, it explained that the inaccessibility of functioning healthcare centers, combined with the absence of medications and diagnostics, impeded blood pressure regulation in flood-affected zones. These disruptions in disease control mechanisms directly elevated the risks of cardiovascular morbidity and mortality in hypertensive patients, whose conditions require continuous pharmacologic and clinical surveillance.

**Disease:** Cardiovascular Disease

**Crisis: Earthquake**

***STRATEGIC OR OPERATIONAL PLANS***

A. The article stated that the 2005 earthquake in Azad Jammu and Kashmir revealed a systemic absence of disaster preparedness plans that addressed the needs of individuals with cardiovascular disease. The authors explained that governmental and nongovernmental organizations lacked clear, context-specific care models for cardiovascular patients during the crisis. This omission meant there were no structured mechanisms for the continuity of care, monitoring of cardiovascular status, or emergency response to acute cardiac events. The article directly linked this failure to the broader inability to meet global noncommunicable disease (NCD) mortality reduction targets, emphasizing that without integrating cardiovascular care into disaster response strategies, health systems cannot protect these high-risk populations during emergencies.

***OTHER***

B. The article described that the mortality of individuals with cardiovascular disease increased following the earthquake due to a combination of neurohormonal, hemodynamic, physical, and emotional factors. Specifically, it stated that disaster-induced stress activates the sympathetic nervous system, elevating heart rate and blood pressure, which increases the risk of acute cardiovascular events such as myocardial infarction or stroke. Simultaneously, the interruption of regular medical care and lack of access to medications created an environment where patients could not manage existing cardiac conditions. The physical strain of displacement, inadequate shelter, and insufficient nutrition further compounded the cardiovascular risk profile in the disaster zone.

**Crisis:** Flooding

***STRATEGIC OR OPERATIONAL PLANS***

A. The article reported that during the 2022 flooding in Pakistan, the national disaster preparedness plans were ineffective in ensuring care for individuals with cardiovascular disease. The flood disrupted already fragile health systems, leaving people with cardiovascular disease without access to life-sustaining services. The authors highlighted that the acute nature of the flooding, combined with the lack of pre-established operational frameworks for chronic disease care, directly led to a deterioration in health outcomes for cardiovascular patients. They emphasized that such systemic failures increase mortality in populations with cardiovascular conditions due to the inability to maintain therapy adherence, perform monitoring, or respond to cardiac symptoms appropriately.

***OTHER***

B. The article explained that people living with cardiovascular disease were adversely affected during the 2022 floods due to stress, reduced physical activity, and absence of access to medical services. It identified that such environmental stressors, combined with the disruption in the supply of medications and diagnostic tools, elevate the likelihood of major adverse cardiovascular events. The flooding damaged healthcare infrastructure and forced the evacuation of healthcare professionals, eliminating regular follow-up and management. This resulted in increased clinical instability for cardiovascular patients who depend on continuous treatment to prevent decompensation.

**Disease:** Acute Kidney Injury (related to dialysis service in 2005 earthquake context)

**Crisis: Earthquake**

***SERVICE PROVISION***

A. The article reported that a nephrology team from Médecins Sans Frontières (MSF) initiated dialysis services within 3 hours after the 2005 earthquake in Azad Jammu and Kashmir. This immediate deployment of specialized renal care was crucial in addressing cases of acute kidney injury (AKI), primarily caused by crush syndrome resulting from collapsed infrastructure and entrapment injuries. The rapid initiation of dialysis enabled timely removal of toxins, correction of electrolyte imbalances, and fluid management—each of which is critical to prevent mortality in AKI patients. The article documented that 88 victims developed AKI, and 55 of them received different hemodialysis sessions. Of these, 11 patients died, yielding a reported mortality rate of 21%. This data underscores the importance of early and organized dialysis support in reducing fatal outcomes related to disaster-induced AKI.

***OTHER***

B. The article explicitly acknowledged that there was no available information regarding the prevalence of diabetes in the subset of earthquake victims who experienced AKI, nor on the comorbidity impact of diabetes on those who received dialysis or died. Furthermore, no published data were available from aid organizations regarding the broader epidemiological or clinical patterns of AKI during the 2005 disaster. The absence of such surveillance or reporting limited the ability to evaluate dialysis outcomes systematically or to formulate disease-specific protocols for future emergency nephrology interventions targeting AKI.

**Crisis: Flooding**

***SERVICE PROVISION***

C. The article stated that there was no information available on the scope of acute kidney injury during the 2022 floodings. There was no mention of dialysis services being initiated, planned, or discussed in this context. No clinical data, service records, or intervention strategies for AKI management were provided for the flood crisis.

15. Article Title: Effective Access to Care in a Crisis Period: Hypertension Control during the COVID-19 Pandemic via Telemedicine

Author, Publication year: Taylor, 2022

the article only discusses one disaster scenario, the COVID-19 pandemic, and

- List of the NCD diseases considered in this article:

**Hypertension, Diabetes Mellitus, Chronic Kidney Disease, Dyslipidemia, Ischemic Heart Disease, Chronic Obstructive Pulmonary Disease (COPD), Asthma, Overweight/Obesity, Prediabetes**

- List all of the crises and disasters considered in this article

(Only non–inside health system crises like natural disasters, conflict, displacement)

Main focus (included in analysis): **Covid-19**

**Disease:** Hypertension
**Crisis: COVID-19 Pandemic**

***SERVICE PROVISION***
A. During the COVID-19 pandemic, in-person primary care access declined significantly, disrupting chronic disease management. In response, the article details the use of video-based telemedicine visits as the core service model to provide hypertension care. These video visits allowed patients to maintain access to board-certified primary care physicians or nurse practitioners who conducted guideline-based hypertension management. Through 1,785 visits with 569 unique patients, a total of 3,626 BP readings were recorded. This continuity of service was critical as 62.4% of patients initially presented with SBP >140 mm Hg. The video model helped patients track progress and permitted ongoing therapeutic adjustments, leading to a mean reduction of 9.7 mm Hg in systolic blood pressure and 6.8 mm Hg in diastolic blood pressure.

B. All telemedicine clinicians followed AHA/ACC hypertension guidelines, including lifestyle management and pharmacologic interventions. These standards ensured that patients received consistent, evidence-based care despite the lack of physical consultations.

C. Physicians reviewed patients’ medication regimens, comorbidities, family history, and lifestyle practices during visits. This approach was not limited to prescribing medication but integrated full-spectrum cardiovascular risk management, facilitating targeted and timely interventions for blood pressure control.

D. Patients received automatic follow-up instructions tailored to hypertension management after each video consultation. This ensured continuous patient education and engagement in managing their condition, reinforcing lifestyle modifications and medication adherence in between visits.

E. Patients averaged 3.1 video visits over one year, each lasting about 12.1 minutes. This high frequency of interaction, despite pandemic-related healthcare disruption, contributed to 55.7% of initially uncontrolled patients (SBP >140/90) achieving controlled blood pressure (≤140/90) by study end.

***DRUG SUPPLY***
F. A total of 478 out of 569 patients (84%) were prescribed at least one new antihypertensive medication during the study period. These prescriptions followed AHA/ACC guidelines and included ACE inhibitors (29.4%), beta blockers (24.0%), calcium channel blockers (24.0%), and diuretics (21.1%). The choice of medication reflected clinical appropriateness based on individual patient profiles discussed during telemedicine encounters. For example, patients with higher initial SBP achieved larger reductions when therapy was escalated.

***TRAINING OF FORCES***
G. All physicians underwent specific training in telemedicine interaction, known as “webside manner,” enhancing their ability to build therapeutic relationships, communicate clearly, and manage hypertension remotely. This training was necessary to ensure that care quality was not compromised by the virtual medium.

H. Most clinicians were additionally trained in AHA/ACC hypertension management guidelines through Doctor On Demand’s internal CME program or board recertification. This ensured uniform application of evidence-based practice across all consultations, maintaining high standards in therapeutic decision-making.

***PATIENT SUPPORT***
I. Lifestyle counseling formed a core component of each video consultation. Physicians educated patients on the DASH diet, physical activity, weight management, smoking cessation, and alcohol reduction. This comprehensive lifestyle support aligned with guideline recommendations to manage hypertension through both pharmacologic and non-pharmacologic interventions.

J. Clinicians performed mental health screenings using PHQ-2, PHQ-9, and GAD-7 where indicated. This addressed psychosocial stressors that may affect blood pressure control, particularly relevant during the COVID-19 crisis, which increased stress and disrupted routines.

***MONITORING AND ASSESSMENT***
K. Patients used AMA-endorsed or Consumer Reports–recommended home BP monitors to self-measure their blood pressure. With an average of 6.4 BP readings per patient, these data informed clinical decision-making during follow-up visits. Home monitoring ensured continuity of care when physical exams were impossible.

L. Patients with the highest baseline SBP (>140 mm Hg) experienced the most significant improvements: a mean reduction of -17.9 mm Hg in SBP and -12.8 mm Hg in DBP. Those with initial BP >130/80 showed reductions of -14.2 mm Hg (SBP) and -9.7 mm Hg (DBP), indicating that the system was especially effective for high-risk individuals.

M. Overall, 77% of the study cohort improved in either systolic or diastolic BP, demonstrating the effectiveness of home-based monitoring in conjunction with video care.

***PATIENT FEEDBACK AND EVALUATION***
N. Satisfaction surveys administered after each visit indicated a mean score of 4.94 out of 5 among 508 respondents (89.3% of the cohort), with 96.6% rating the visit 4 or 5. This reflects high patient engagement and acceptance of the telemedicine-based hypertension program, critical for adherence to follow-up and sustained BP control.

**Disease:** Diabetes Mellitus
**Crisis: COVID-19 Pandemic**

***SERVICE PROVISION***
A. The article states that 7.9% of the 569 patients in the study cohort had diabetes mellitus. Although hypertension was the primary focus, patients with comorbid diabetes received care via structured video-based telemedicine visits. These visits were conducted by board-certified primary care physicians who followed comprehensive protocols that included a review of comorbidities. This indicates that diabetes mellitus was evaluated during clinical decision-making, which is particularly important because diabetes is a risk factor that complicates hypertension management. The telemedicine model enabled patients with diabetes to maintain ongoing contact with healthcare professionals during a period when face-to-face primary care services were significantly reduced due to the pandemic. The structure of the visits included assessment of lifestyle, medications, and other comorbidities, thereby preserving access to integrative chronic disease management for individuals with both hypertension and diabetes mellitus. (no specific p-value/OR/CI reported)

***MONITORING AND ASSESSMENT***
B. Although diabetes-specific biomarkers such as fasting glucose or HbA1c were not collected, the article reports that comorbidities, including diabetes mellitus, were documented in the electronic health record during the video consultations. This documentation allowed physicians to tailor treatment plans accordingly. Given that diabetes mellitus can influence blood pressure targets and pharmacologic choices, its identification within the consultation framework ensured that patients received context-specific care. The integration of diabetes status into the clinical workflow suggests that the telemedicine model supported individualized treatment even in the absence of direct lab measurements. This approach would have helped mitigate risks of unmanaged glycemia during the period of restricted in-person access. (no specific p-value/OR/CI reported)

**Disease:** Chronic Kidney Disease
**Crisis: COVID-19 Pandemic**

***SERVICE PROVISION***
A. The article reports that 0.5% of the study cohort (n=3 out of 569 patients) had a diagnosis of chronic kidney disease. These individuals were included in the video-based telemedicine hypertension care model during the COVID-19 pandemic. Although chronic kidney disease was not the primary focus, its documentation during consultations ensured that physicians considered renal function when managing hypertension. This is critical, as patients with chronic kidney disease require specific considerations regarding blood pressure targets and drug selection. The telemedicine model maintained clinical oversight for these patients when in-person care was disrupted. (no specific p-value/OR/CI reported)

***MONITORING AND ASSESSMENT***
B. Chronic kidney disease was explicitly listed as a comorbidity in the demographic data collected and reviewed during telemedicine visits. This allowed healthcare providers to factor renal status into treatment decisions, particularly in selecting antihypertensive medications that are safe and effective for individuals with impaired renal function. Although no laboratory data such as eGFR or creatinine were included, the presence of chronic kidney disease in the patient record informed clinical judgment and therapeutic planning during the video consultations.

**Disease:** Dyslipidemia
**Crisis: COVID-19 Pandemic**

***SERVICE PROVISION***
A. The article reports that 15.5% of the cohort (n=88 out of 569 patients) had a diagnosis of dyslipidemia. These patients received care through video-based telemedicine consultations during the COVID-19 pandemic. Although the primary diagnosis guiding care was hypertension, the presence of dyslipidemia as a comorbidity was acknowledged and documented in the electronic health record during consultations. This allowed primary care physicians to consider lipid disorders in their risk assessment and treatment strategy for cardiovascular disease during remote visits, which were the primary mode of care delivery during the pandemic. The inclusion of this comorbidity supported an integrated approach to chronic disease management despite healthcare service disruptions. (no specific p-value/OR/CI reported)

***MONITORING AND ASSESSMENT***
B. Dyslipidemia was explicitly identified as a comorbidity in the baseline characteristics table and considered during clinical encounters via telemedicine. This ensured that physicians remained aware of patients' elevated cardiovascular risk profiles during hypertension management. While no lipid panel data were reported, the documented presence of dyslipidemia suggests it was included in risk stratification and influenced decision-making, particularly regarding cardiovascular prevention strategies. (no specific p-value/OR/CI reported)

**Disease:** Ischemic Heart Disease
**Crisis: COVID-19 Pandemic**

***SERVICE PROVISION***
A. The article reports that 0.5% of the total study population (n=3 out of 569 patients) had a documented diagnosis of ischemic heart disease. These patients were managed using a telemedicine video visit model during the COVID-19 pandemic, which replaced in-person care that had been reduced by more than 25% nationally due to public health restrictions. Although the primary focus was hypertension, the presence of ischemic heart disease as a comorbidity necessitated tailored risk management during consultations. The platform allowed physicians to provide holistic care addressing both hypertension and coexisting cardiovascular conditions, ensuring that those with established ischemic pathology remained within clinical oversight during a time of widespread healthcare access disruption. The virtual care format preserved therapeutic continuity for this high-risk subgroup when primary care infrastructure was otherwise inaccessible. (no specific p-value/OR/CI reported)

***MONITORING AND ASSESSMENT***
B. Ischemic heart disease was specifically identified and recorded among the comorbidities of the patient population, with 3 individuals affected. This documentation was incorporated into the patient’s electronic health records and reviewed during structured telemedicine consultations. This enabled physicians to consider cardiovascular risk in all management decisions, particularly when adjusting antihypertensive medications that could interact with or impact cardiac function. For example, beta blockers were prescribed to 24% of patients overall and are commonly used in ischemic heart disease, implying potential alignment between pharmacologic choices and comorbid conditions. While the article does not include ischemia-specific outcome measures or biomarker monitoring (e.g., troponins, ECGs), the recorded history of ischemic heart disease influenced clinical judgment during remote management sessions. (no specific p-value/OR/CI reported)

**Disease:** Chronic Obstructive Pulmonary Disease (COPD)
**Crisis: COVID-19 Pandemic**

***SERVICE PROVISION***
A. The article identifies 1.5% of the study cohort (n=8 out of 569 patients) as having COPD. These patients were managed using a structured telemedicine platform during the COVID-19 pandemic. Video-based visits were conducted with board-certified primary care physicians, allowing for individualized care that integrated management of comorbid COPD with hypertension. The telemedicine format was vital for maintaining continuous healthcare access during a period when in-person services were limited due to pandemic-related restrictions. This was particularly significant for COPD patients, who are at increased risk of severe COVID-19 outcomes and often require frequent monitoring. The remote system reduced the need for clinic visits, thereby limiting their exposure risk while maintaining physician oversight. (no specific p-value/OR/CI reported)

***MONITORING AND ASSESSMENT***
B. COPD was recorded as a comorbidity in the patient electronic health records and reviewed during telemedicine encounters. This allowed clinicians to account for pulmonary disease status when formulating treatment strategies for hypertension. While no pulmonary function metrics were collected, the explicit listing of COPD in the data table meant it was factored into prescribing decisions, especially where medications such as beta blockers could pose respiratory risks. This consideration prevented potential drug-disease interactions and ensured safer hypertension management in patients with compromised respiratory function. (no specific p-value/OR/CI reported)

**Disease:** Asthma
**Crisis: COVID-19 Pandemic**

***SERVICE PROVISION***
A. Asthma was present in 6.3% of the cohort (n=36 out of 569 patients), and these individuals were also treated through video-based telemedicine visits during the COVID-19 crisis. This model ensured continuity of care for patients with asthma who were concurrently being treated for hypertension. The virtual consultations allowed physicians to assess patient history and comorbidities and to avoid treatments that could aggravate asthma symptoms. The significance of using telemedicine for this group lies in minimizing the need for in-person clinic visits, which is crucial given the increased vulnerability of asthma patients to respiratory infections, including COVID-19. These visits allowed real-time evaluation and adjustments in management based on comorbidity considerations. (no specific p-value/OR/CI reported)

***MONITORING AND ASSESSMENT***
B. Asthma was explicitly documented as a comorbidity in the baseline demographic data and included in the patient health records accessed during video visits. Its presence in the dataset signaled to clinicians the need for caution when choosing antihypertensive drugs, particularly avoiding non-selective beta blockers that can induce bronchoconstriction. Although spirometry or peak flow monitoring were not included, the awareness of asthma status enabled the healthcare provider to adapt treatment decisions in alignment with respiratory safety and patient history. (no specific p-value/OR/CI reported)

**Disease:** Overweight/Obesity
**Crisis: COVID-19 Pandemic**

***SERVICE PROVISION***
A. The article reports that 12.0% of the study cohort (n=68 out of 569 patients) were classified as having overweight or obesity. These individuals received structured telemedicine care from board-certified primary care physicians during the COVID-19 pandemic. Although the primary focus was hypertension, the presence of overweight/obesity was documented and reviewed in the electronic health records during virtual consultations. This allowed clinicians to include weight status in their clinical decision-making and care planning, particularly as excess weight is a well-established risk factor for both hypertension and adverse COVID-19 outcomes. The telemedicine system provided a platform to maintain healthcare engagement without requiring physical attendance, which was essential during pandemic-related service limitations. (no specific p-value/OR/CI reported)

***SUPPORT***
B. Lifestyle counseling was provided during telemedicine visits, and physicians advised patients on weight management strategies as part of holistic hypertension care. This included guidance on the DASH diet, increased physical activity, reduction of alcohol consumption, and cessation of smoking. These interventions, although framed in the context of hypertension, directly supported weight reduction efforts in patients with overweight or obesity. The structured inclusion of such advice during remote visits enabled ongoing support despite the inability to attend in-person lifestyle programs or clinics during the pandemic. (no specific p-value/OR/CI reported)

**Disease:** Prediabetes
**Crisis: COVID-19 Pandemic**

***SERVICE PROVISION***
A. The article identifies that 2.6% of the cohort (n=15 out of 569 patients) had prediabetes. These patients were treated through the same video-based telemedicine system used for hypertension management during the COVID-19 pandemic. Prediabetes was documented in the electronic health records and considered as part of the clinical assessment during telemedicine consultations. Although the study did not target prediabetes management directly, its presence informed risk evaluation and influenced comprehensive care planning. The availability of virtual visits ensured these patients remained connected to the healthcare system during a time of decreased in-person service capacity. (no specific p-value/OR/CI reported)

***SUPPORT***
B. Physicians provided structured lifestyle counseling during telemedicine encounters, which included recommendations relevant to glycemic control. These included dietary guidance (e.g., DASH diet), increased physical activity, and behavioral changes such as weight management and smoking cessation. These strategies align with standard prediabetes management and were embedded in the holistic care framework used for hypertension during remote visits. The provision of these interventions during video visits allowed for sustained preventive care for patients at elevated risk of developing diabetes mellitus. (no specific p-value/OR/CI reported)

16. Article Title: Barriers to Diabetes Care during Humanitarian Crisis during 2013–2022 in Five Arabian Countries: A Systematic Review

Author, Publication year: Al-Nimer, 2023

The article discusses combined crises (wars, conflicts, economic hardship) affecting diabetes care but does not stratify strategies or measures by specific disaster types.

- List of the NCD diseases considered in this article:

**Diabetes Mellitus, Hypertension, Heart Disease**

- List all of the crises and disasters considered in this article

(Only non–inside health system crises like natural disasters, conflict, displacement)

- Main focus (included in analysis): **Wars, Conflicts, Economic Crisis, ISIS Insurgency, Humanitarian Crisis, Displacement**

**DISEASE:** Diabetes Mellitus

**Crisis: Wars, Conflicts, Economic Crisis, Humanitarian Crisis, Displacement**

***DRUG SUPPLY***

A. Metformin and glibenclamide were highlighted as the most accessible antidiabetic medications for displaced populations due to their low cost and widespread availability. These medications were consistently provided to diabetes patients even in settings with limited healthcare infrastructure. The reason for their prioritization lies in the affordability and practicality of oral agents in low-resource settings, which ensured at least minimal therapeutic management for patients with diabetes.

B. Multiple studies identified the limited availability of critical diabetes medications such as insulin and insulin analogues (e.g., insulin degludec, insulin detemir, insulin glargine), as well as dapagliflozin and gliclazide, particularly in Syria, Iraq, and among Palestinian refugees. For instance, UNRWA clinics in Syria faced drug access issues, which hindered the ability of patients to manage diabetes effectively. The article explicitly mentioned that medication shortages were attributed to the financial crises and collapse of health infrastructures in war-torn regions, limiting the continuity and adequacy of pharmacologic treatment.

C. In Iraq, during the period of ISIS occupation and systemic corruption, displaced populations were unable to afford privately sold medications due to drug supply chain breakdowns. This lack of availability forced patients either to skip doses or discontinue treatment altogether, directly contributing to poor glycemic control.

D. In a study on 475 diabetes patients in Iraq, only 4.3% of patients adhered to the optimal metformin dosage, indicating that both supply shortages and patient behavior (reluctance or fear of side effects) significantly compromised the therapeutic outcomes in diabetic care. The inability to provide consistent medication supplies directly impacted disease control.

***SERVICE PROVISION***

A. In Jordan and Palestine, the use of e-Health systems in primary healthcare settings allowed for effective tracking and monitoring of diabetes patients. These systems enabled health workers to identify patients at risk of poor glycemic control, ensure follow-ups, and document treatment adherence. For example, in Nuzha PHC clinics, patients were monitored for over three years using cohort analyses, which demonstrated the long-term utility of digital platforms for continuity of care during crises.

B. The Médecins Sans Frontières (MSF) model of care implemented in the Shatila refugee camp for Syrian and Palestinian patients centered on regular consultations, lab monitoring, and individualized treatment plans. This model improved self-reported medication adherence among patients with diabetes due to its integrated design, even in contexts with limited physical healthcare infrastructure.

C. In Lebanon, Syrian refugees faced discrimination and unequal treatment by health workers in both public and private clinics, which diminished their access to outpatient diabetes services. This disparity in service provision between host communities and refugees led to inferior glycemic outcomes among displaced populations.

D. Among 59,617 Syrian refugees in Jordan, high rates of obesity, complications, and comorbidities associated with diabetes were observed. These outcomes were attributed to inconsistent access to essential healthcare services, indicating that poor service provision under humanitarian crisis settings exacerbated disease progression.

***SUPPORT***

A. In the Gaza Strip, the absence of a structured social support system contributed to low adherence to non-pharmacological interventions (e.g., physical activity, dietary changes). This lack of support mechanisms weakened the overall effectiveness of diabetes management efforts in the population.

B. In a study involving 380 patients with type 2 diabetes in Palestine, 42.1% were found to have low medication adherence due to their beliefs that antidiabetic drugs were ineffective. These perceptions stemmed from inadequate support in the form of counseling and educational reinforcement from health professionals.

C. Among Palestinian refugees in Jordan, medication adherence was significantly higher when patients expressed satisfaction with their care and trusted their healthcare providers, especially those working in UNRWA clinics. This shows that interpersonal support and trust in care delivery teams are central to sustaining diabetes management.

***STRATEGIC OR OPERATIONAL PLANS***

A. A community-based study demonstrated that a low-cost e-Health notebook application effectively identified undiagnosed diabetes cases in under-resourced refugee communities in Lebanon. The plan emphasized integrating early detection systems into humanitarian responses, allowing for timely referral and management.

B. In the West Bank, a quasi-experimental study involving 100 patients showed that the application of a Diabetes Comprehensive Care Model significantly improved glycemic control. This strategic intervention involved structured care delivery with regular assessments, demonstrating the potential for scalable models even in resource-constrained environments.

***TRAINING OF FORCES***

A. A study on 115 Lebanese physicians showed that gaps in physician knowledge and clinical guideline adherence were significant barriers to effective diabetes care. Physicians required structured training programs to enhance their ability to manage diabetes during crises, particularly given the resource limitations and complex care needs of displaced populations.

B. In Lebanon, 70% of 235 hospitalized patients with hyperglycemia were mismanaged by nursing staff, mainly due to the improper administration of insulin. This highlights the need for training on insulin therapy protocols to ensure safe and effective inpatient diabetes management.

***IMPROVEMENT OF INTER-SECTORAL COMMUNICATIONS***

A. The MSF model employed in refugee camps facilitated coordinated care among health workers, community staff, and logistical teams. This integration across sectors improved service delivery, medication adherence, and disease monitoring for diabetic patients. By synchronizing operations, the model overcame fragmentation of care, a common problem in crisis zones.

***PROVISION OF BUDGET***

A. In Lebanon, the compounded impact of the COVID-19 pandemic and national economic collapse made it difficult for diabetic patients to access primary and secondary care. Eight diabetes patients who underwent amputations during this period reported that neither governmental nor non-governmental systems provided adequate funding or accessible services.

B. In a survey of 1,363 Syrian refugees with diabetes in Jordan, 31.6% reported financial hardship as the primary barrier to accessing healthcare. This financial limitation prevented routine care visits and medication purchases, contributing to poor health outcomes.

***PREVENTIVE EDUCATION AND HEALTH LITERACY***

A. A study in the Gaza Strip identified low health literacy as a principal factor in poor adherence to diabetes treatment guidelines. Patients with limited understanding of their disease had higher HbA1c levels and were more likely to ignore recommended care plans.

B. In a large-scale study of 8,669 Palestinian women, the use of an e-Registry with clinical decision support tools for antenatal care resulted in superior outcomes for gestational diabetes. The intervention enhanced provider adherence to treatment protocols and improved patient education and engagement.

C. The article noted across multiple studies that inadequate diabetes knowledge among both patients and healthcare providers was a recurrent cause of poor glycemic control, missed follow-ups, and treatment discontinuation.

***COMMUNITY ENGAGEMENT***

A. In a cross-sectional study in Palestine, it was found that strengthening the patient-provider relationship through community-based interventions improved glycemic control and reduced out-of-pocket costs. Interpersonal engagement and collaborative decision-making were emphasized as central to sustainable diabetes care.

B. In Lebanon, integrating culturally familiar alternative medicine practices and educational interventions helped build trust with displaced populations, leading to better adherence to diabetes care plans.

**DISEASE:** Hypertension
**Crisis: Wars, Conflicts, Economic Crisis, Humanitarian Crisis, Displacement**

***SERVICE PROVISION***

A. In Lebanon, the use of a longitudinal cohort study involving m-Health technology improved adherence to clinical guidelines for hypertension and enhanced the quality of care delivered to vulnerable groups, including refugees. The mobile health application provided real-time clinical decision support to healthcare providers working in primary health centers. This led to more consistent diagnosis and monitoring of blood pressure and adherence to hypertension treatment protocols. The system compensated for limited provider training and infrastructure in crisis-affected regions by automating part of the treatment decision-making process. It served as a technological bridge where healthcare continuity was disrupted due to population displacement and conflict-related facility shortages.

B. In the Shatila refugee camp in Beirut, the Médecins Sans Frontières (MSF) model of care included specific hypertension management protocols adapted for crisis settings. Patients received individualized plans, regular follow-ups, and educational reinforcement. This structured care approach supported sustained blood pressure control among Syrian and Palestinian refugees. By centralizing chronic care within a humanitarian camp and ensuring ongoing monitoring and access to treatment, the MSF model prevented the clinical deterioration often seen in unmanaged hypertension during displacement.

C. A large-scale cluster design study in Jordan included 8,041 Syrian refugees, revealing that 17.6% of participants were living with hypertension, either alone or in combination with diabetes. This population was predominantly elderly and poorly educated, which significantly increased their vulnerability. The study highlighted the absence of structured hypertension care and continuity of services in the camps and host communities. As a result, many refugees failed to access regular blood pressure monitoring and were unable to maintain treatment, leading to an increased risk of cardiovascular complications. The humanitarian setting lacked systematic protocols to follow up on hypertensive patients, especially among high-risk subgroups.

***PREPARATION OF STRATEGIC OR OPERATIONAL PLANS***

A. In Lebanon, a community-based health screening initiative using a low-cost e-Health notebook was designed to detect undiagnosed cases of hypertension and diabetes among refugees and marginalized local populations. This intervention established a scalable model for early identification and linkage to care in under-resourced crisis settings. The notebook application allowed field workers to input health screening data, which triggered alerts and generated referrals for hypertensive patients. This strategy bridged diagnostic gaps caused by the disruption of standard public health services, enabling timely access to treatment and reducing the likelihood of uncontrolled blood pressure among displaced individuals.

***PROVISION OF BUDGET***

A. A cross-sectional study involving 1,700 patients in Lebanon showed that outpatient healthcare service utilization for hypertension was strongly stratified by nationality and gender. Refugees experienced more restricted access compared to host populations, mainly due to financial constraints. The article emphasized that hypertension care was significantly impacted by the unpredictability and magnitude of healthcare costs. Syrian refugees, in particular, faced limitations in accessing diagnostics and medications due to out-of-pocket expenses, which were unaffordable under their economic circumstances. The financial burden directly limited adherence and continuity of hypertension care.

B. In Jordan, qualitative reports from Syrian refugees described a pattern of hypertension treatment interruption due to medication cost and unaffordable private clinic fees. Patients cited that financial instability forced them to ration medications or discontinue them altogether, resulting in poorly managed blood pressure. The study identified economic barriers as a dominant constraint in the long-term control of hypertension in displaced populations, where governmental or humanitarian funding mechanisms were insufficient to cover the actual cost of care.

***SUPPORT***

A. Among 763 Palestinian refugees with either hypertension or diabetes, the study found that medication adherence was strongly influenced by the perceived quality of interaction with healthcare providers. Patients who trusted their providers and received clear explanations of their condition and treatment were significantly more likely to adhere to antihypertensive regimens. Satisfaction with the health system and confidence in care delivery were key psychological supports that contributed to better blood pressure management. Conversely, fragmented care and multiple uncoordinated providers contributed to confusion and non-adherence.

***TRAINING OF FORCES***

A. In Lebanon, the implementation of m-Health applications embedded decision-support systems into provider workflows. These tools served as an indirect training mechanism by prompting healthcare professionals with real-time recommendations based on hypertension guidelines. The study implied that such digital interventions were particularly critical in humanitarian settings where formal hypertension training was often unavailable. By reinforcing best practices at the point of care, the application mitigated provider knowledge deficits and promoted guideline-concordant hypertension management.

**DISEASE:** Heart Disease
**Crisis: Wars, Conflicts, Economic Crisis, Humanitarian Crisis, Displacement**

***PROVISION OF BUDGET***

A. Syrian refugees with heart disease in Jordan reported that access to cardiac care was severely constrained by a lack of funding in both governmental and non-governmental health systems. The article described that displaced individuals were unable to pay for essential cardiovascular services, including diagnostic tests, physician consultations, and hospital-based care. These financial limitations directly prevented individuals from accessing timely treatment for ischemic heart conditions, heart failure, and other serious cardiac disorders. Because cardiac care often involves expensive diagnostics such as electrocardiograms, echocardiography, and lab work, the cost barrier became a determinant of unmanaged heart disease among the refugee population.

B. A study conducted at Jordan University Hospital underscored that Syrian refugees with heart disease were disproportionately affected by the economic burden of health services. Patients requiring hospitalization for acute cardiac episodes faced service denial or delay due to their inability to cover hospital fees. The article explicitly indicated that the absence of funding for emergency interventions led to interruptions in acute care pathways, which placed heart disease patients at risk of avoidable morbidity and mortality. Furthermore, the financial pressure extended to post-discharge follow-up, preventing patients from obtaining necessary medications or engaging in long-term cardiac rehabilitation.

***SERVICE PROVISION***

A. The article stated that service provision for Syrian refugees with heart disease in Jordan was inadequate due to systemic fragmentation and lack of access to specialized cardiology care. Patients were unable to receive continuous care across the healthcare system, and referrals between service providers were inconsistent. This disrupted the management of chronic cardiac conditions such as coronary artery disease and hypertensive heart failure, as patients missed critical diagnostic evaluations, treatment initiation, and routine follow-up. Without consistent access to cardiologists or diagnostic imaging, patients were managed symptomatically rather than based on evidence-based protocols, resulting in poor clinical outcomes.

B. The lack of integrated care between humanitarian organizations, governmental facilities, and private providers contributed to delayed interventions for acute cardiac events. The article highlighted that heart disease patients often experienced postponements in receiving time-sensitive procedures, such as diagnostic catheterization or hospitalization for decompensated heart failure. These delays were caused by both administrative barriers and an overwhelmed healthcare system. This deficiency in service infrastructure undermined the capacity to stabilize and monitor patients with life-threatening cardiovascular conditions.

***SUPPORT***

A. The article briefly referenced that psychosocial support systems were underdeveloped for Syrian refugees with heart disease. Refugees reported feelings of mistrust and disconnection from healthcare providers, which diminished treatment adherence and follow-up participation. The lack of structured provider-patient communication reduced patients’ understanding of their cardiac conditions and the importance of long-term management. Without emotional support and consistent clinical counseling, patients disengaged from care, leading to higher risk of uncontrolled symptoms and complications. This absence of psychosocial reinforcement was especially critical for conditions requiring daily lifestyle modifications and regular medication intake, such as heart disease.

17. Article Title: Noncompliance with Hypertension Treatment and Related Factors among Kumamoto Earthquake Victims Who Experienced the COVID-19 Pandemic during Postearthquake Recovery Period

Author, Publication year: Ide-Okochi, 2023

The article analyzes the combined impact of the 2016 Kumamoto earthquake and the COVID-19 pandemic but does **not** stratify strategies or measures by each disaster type.

List of the NCD diseases considered in this article:

**Hypertension**

- List all of the crises and disasters considered in this article

(Only non–inside health system crises like natural disasters, conflict, displacement)

- Main focus (included in analysis): **Earthquake: 2016 Kumamoto earthquake, COVID-19**

**Disease:** Hypertension
**Crisis: Earthquake (2016 Kumamoto Earthquake)**

***SUPPORT***
A. Living in non-owner-occupied housing, including rental housing, public housing, and restoration public housing (RPH), was significantly associated with higher odds of hypertension treatment noncompliance. The study found adjusted odds ratios (AOR) of 1.92 (95% CI: 1.20–3.07) for rental housing, 2.47 (95% CI: 1.38–4.42) for public housing, and 4.12 (95% CI: 1.14–14.90) for RPH. These findings indicate that those who had to relocate to government-supported or temporary dwellings after the earthquake were less likely to continue treatment. The authors explicitly connect this housing instability with social isolation, economic strain, and limited access to health services, which collectively contribute to the disruption of hypertension care.

B. Individuals reporting poor self-rated health were more likely to exhibit noncompliance with hypertension treatment. The study identified an AOR of 2.49 (95% CI: 1.72–3.61) for those who considered their health to be poor. This suggests that perceived health decline, potentially exacerbated by earthquake-induced psychological and environmental stressors, impedes consistent engagement with hypertension management.

***PROVISION OF BUDGET***
C. The study emphasizes the necessity of financial intervention to prevent treatment interruption. Many participants cited financial burden as the main reason for discontinuing treatment. Those affected by the earthquake faced prolonged economic instability, especially if residing in non-owner-occupied housing. Although the article does not give a separate AOR for income in the earthquake context alone, financial difficulty due to compounded crises was described as an urgent barrier requiring public financial support mechanisms.

***SERVICE PROVISION***
D. 31.2% of hypertensive respondents were identified as noncompliant with treatment, with primary reasons being the cost of care, lack of time, and low perceived need for medical consultation. These findings underscore gaps in access to consistent, post-disaster outpatient care. The earthquake disrupted medical infrastructure and increased the burden on survivors, who prioritized housing and income recovery over chronic disease management. Ensuring service continuity and outreach was noted as critical to avoid treatment lapses.

***STRATEGIC OR OPERATIONAL PLANS***
E. The study underscores the need for long-term operational planning to monitor and support individuals with hypertension following major disasters. It states that identifying the risk factors for treatment noncompliance during the recovery phase is necessary to design interventions. The earthquake's prolonged impact on mental health, housing, and healthcare engagement points to the importance of integrated disaster-health planning tailored to chronic disease needs.

**Crisis: COVID-19**

***SUPPORT***
A. Reduced income due to the COVID-19 pandemic was strongly associated with noncompliance in hypertension treatment, with an AOR of 3.23 (95% CI: 2.27–4.58). Economic loss stemming from the pandemic restricted the ability of patients to afford regular medical visits or medications. The study emphasizes that the dual burden of earthquake recovery and pandemic-induced financial strain significantly compounded vulnerability, making economic support essential for treatment continuity.

B. Poor self-rated health during the pandemic also contributed to noncompliance. Individuals who perceived their health as poor were significantly more likely to discontinue or avoid hypertension treatment (AOR = 2.49, 95% CI = 1.72–3.61). The psychological impact of the pandemic, including stress, isolation, and fear of infection, likely contributed to both perceived and actual deterioration of health, leading patients to disengage from their usual care routines.

***SERVICE PROVISION***
C. The pandemic caused a 32% reduction in cardiovascular-related hospitalizations in Japan. In the study population, this effect manifested as a 31.2% treatment noncompliance rate, with participants reporting financial burden, time constraints, and lack of perceived necessity as reasons. The article emphasizes that restricted mobility, fear of infection, and altered healthcare priorities during COVID-19 hindered access to hypertension management services.

***STRATEGIC OR OPERATIONAL PLANS***
D. The article asserts that the pandemic must be recognized as an additional burden layered on top of the earthquake, necessitating updated and targeted strategic planning. It recommends integrating pandemic-related socioeconomic and healthcare access challenges into chronic disease response strategies to prevent deterioration in hypertension management among affected populations.

***PROVISION OF BUDGET***
E. The economic fallout of the pandemic is directly linked to hypertension treatment disruption. The study specifically identifies decreased income as a statistically significant factor (AOR = 3.23, 95% CI = 2.27–4.58), indicating that budgetary resources must be allocated to support those whose financial situation deteriorated due to COVID-19, in order to sustain adherence to hypertension treatment.

18. Article Title: Challenges of providing health services to patients with cardiovascular diseases during disasters in Iran: A qualitative study

Author, Publication year: Moslehi, 2023

The article discusses both natural and unnatural disasters (e.g., earthquakes, war, road accidents) collectively and addresses general challenges and strategies for cardiovascular disease (CVD) care without stratifying specific measures by each disaster type.

- List of the NCD diseases considered in this article:

**Cardiovascular Diseases, Diabetes, Cancer, Chronic Respiratory Diseases**

- List all of the crises and disasters considered in this article

(Only non–inside health system crises like natural disasters, conflict, displacement)

- Main focus (included in analysis): **earthquakes, War (Holy Defense), Road Accidents, Fires, Natural Disasters (general), Unnatural Disasters (general), Humanitarian Situations (implied but not deeply elaborated)**

**Disease:** Cardiovascular Diseases
**Crisis: All Crises (Combined Disasters)**

***DRUG SUPPLY***

A. Lack of access to medications was reported as a major problem during disasters, as infrastructures are often destroyed, interrupting the pharmaceutical supply chain. This leads to difficulties in the availability of prescribed medications for cardiovascular patients who require uninterrupted treatment to prevent acute events such as myocardial infarctions or hypertensive crises. Without timely access to antihypertensive drugs, anticoagulants, or antianginal medications, the risk of disease exacerbation increases significantly.
B. The article emphasized the necessity of planning for drug distribution systems in advance, including identifying essential cardiovascular medications. This is essential because sudden disruption in access could rapidly destabilize patients with chronic heart conditions, leading to hospitalizations or mortality.
C. Patients were advised to keep a “medicine bag” containing sufficient medication for the initial days following a disaster. This practice helps maintain treatment continuity during critical hours when health systems are non-functional or overwhelmed.

***SERVICE PROVISION***

A. Disasters create barriers to maintaining dietary control and access to health services, leading to increased emergency referrals of cardiovascular patients. Inadequate nutrition, stress, and environmental instability can lead to decompensation in conditions like hypertension, heart failure, or arrhythmias. For example, consumption of canned foods high in sodium during crises can directly elevate blood pressure and increase cardiovascular strain.
B. Sleep and rest pattern disruption is a recognized complication in emergency shelters. The presence of noise, overcrowding, and lack of privacy exacerbates cardiovascular conditions by increasing stress hormone release and sympathetic nervous system activity, both of which raise blood pressure and increase the risk of acute coronary events.
C. A severe shortage of skilled health personnel was noted, leading to compromised care delivery. Cardiovascular patients require precise and rapid decision-making for medication titration, monitoring vital signs, and responding to acute decompensations. The lack of appropriately trained providers places these patients at risk of deteriorating outcomes.
D. The absence of psychological interventions leaves cardiovascular patients vulnerable to mental distress, which has direct effects on cardiovascular stability. Anxiety and depression are strongly associated with increased levels of catecholamines, which elevate blood pressure and heart rate, precipitating cardiac events.
E. The design and security of emergency shelters were emphasized as crucial to preventing cardiovascular deterioration. Inadequate housing conditions create stress and restrict rest, which in turn elevates blood pressure. Poor food availability, especially low potassium and high sodium intake, leads to further cardiovascular risks.

***SUPPORT***

A. Psychological and emotional support during disasters was identified as crucial. The presence of family members helps reduce feelings of abandonment and fear in cardiovascular patients, thereby lowering stress levels and associated cardiovascular strain. Isolation during crises leads to increased psychological vulnerability and worsens disease management.

***PREPARATION OF STRATEGIC OR OPERATIONAL PLANS***

A. The absence of strategic management protocols was considered a core problem. Cardiovascular patients, due to their high dependency on continuous care, require advance planning in hospital protocols, including clear guidelines on triage, monitoring, and referral during disasters.
B. Hospitals were encouraged to engage in infrastructure readiness and capacity planning. Due to the time-lag between disaster onset and cardiovascular symptom exacerbation, hospitals must be prepared to receive waves of patients several hours after the event. Without pre-existing operational plans, such delayed yet urgent influx leads to preventable complications and death.
C. Risk management systems were advocated, specifically tailored to the geographic and systemic vulnerabilities of the area. Cardiovascular patient registries, resource planning for essential drugs, and allocation of trained staff were considered critical to mitigate the effects of disasters.

***TRAINING OF FORCES***

A. Health personnel must undergo continuous and structured in-service training to develop competence in disaster-specific cardiovascular care. This includes identification of early signs of decompensation, medication adjustments, and emergency interventions tailored to cardiovascular pathologies.
B. Training must also prepare providers to offer psychological counseling and reassurance, as psychological factors play a significant role in disease destabilization. The article emphasized the importance of this dual competency—clinical and psychological—to ensure comprehensive care.

***CREATION OF STORAGE RESOURCES***

A. The article emphasized that pre-crisis planning must include the creation of storage systems for essential cardiovascular medications. This ensures uninterrupted therapy during the initial phase of a disaster when health logistics are most compromised. Patients’ personal storage, such as carrying medication bags, was also recommended as an individual-level resource measure.

***IMPROVEMENT OF INTER-SECTORAL COMMUNICATIONS***

A. Interoperable and integrated digital health record systems were recommended to ensure access to cardiovascular patients' medical histories during disasters. Lack of accessible records severely impairs continuity of care and delays necessary interventions. Health systems must ensure real-time data availability on blood pressure records, lipid profiles, medication regimens, and comorbid conditions to facilitate evidence-based disaster response.

***OTHER***

A. The identification of cardiovascular patients through a national or local registry was highlighted. Such registries should include demographic data and clinical parameters such as systolic/diastolic blood pressure, body mass index, and lipid profiles. This allows prioritization during triage and allocation of limited resources.
B. The inclusion of family members in nutrition planning was recommended as a behavioral strategy. Family-centered interventions are effective in shaping patients’ food habits, which is critical in disaster settings where food choices are limited and often detrimental to cardiovascular health.

**19. Article Title:** The resiliency of noncommunicable diseases services during the public health crisis: a lesson from Bangkok, Thailand

**Author, Publication year:** Pitayarangsarit, 2023

The article discusses cyclone, flood, and storm-related disasters as a combined category throughout, and does not stratify specific strategies or measures by disaster type. But the article does stratify the impact and relevant considerations by disease type

**List of the NCD diseases considered in this article:** Hypertension, Diabetes Mellitus

**• List all of the crises and disasters considered in this article**
(Only non–inside health system crises like natural disasters, conflict, displacement)
COVID-19 Pandemic

**Disease:** Hypertension
**Crisis: COVID-19 Pandemic**

***AREA: Service Provision***
A. Hypertension services continued to operate more effectively than services for diabetes mellitus during the COVID-19 pandemic. The article explicitly noted that hypertension patients showed better health outcomes compared to diabetes patients in 2021. This differential outcome was attributed to the lesser complexity of service requirements for hypertension, which does not demand intensive laboratory monitoring like blood sugar or HbA1C. This relative simplicity enabled healthcare providers to maintain hypertension services more easily under pandemic constraints. The findings suggest that the service model for hypertension was more resilient because it could operate with limited resources and staff, as it required fewer professional procedures and specialized equipment.
B. The continuity of care for hypertension was supported by the structure of existing services that focused on routine follow-ups and medication management, which were less disrupted than services demanding diagnostic interventions. Consequently, even under workforce and operational strain, hypertension patients experienced comparatively stable care continuity.

***AREA: Health Outcome Comparison for Strategic Planning***
C. In 2021, only 30% of diabetes mellitus patients were able to maintain glycemic control, whereas hypertension patients exhibited better health outcomes. This contrast was highlighted in the article to underline the necessity for stratified service planning. Hypertension services achieved more favorable results during the crisis because they required less clinical intervention and were less affected by the suspension of laboratory-based services. The outcome underscores the importance of distinguishing between diseases requiring different levels of clinical dependency in strategic healthcare planning, especially during resource-limited emergencies such as pandemics.

***AREA: Workforce Allocation***
D. Healthcare personnel who were responsible for NCD clinics, including hypertension, were reassigned to COVID-19-related duties. During the first wave, 59.2% of facilities reported partial redeployment of NCD staff, and this increased to 77.8% in the second wave. This reallocation of staff caused disruptions in the routine operation of hypertension clinics. However, the relative success of hypertension services despite this redeployment indicates that such services may be less sensitive to staffing disruptions compared to more resource-intensive NCD services. The article implicitly identifies that hypertension management, which often involves medication adherence and blood pressure monitoring, remained feasible even under constrained human resources.

***AREA: Continuity of Medication***
E. The study reported that only 24.7% of healthcare facilities were able to provide medication continuity to more than 95% of their NCD patients, including those with hypertension, during the first wave of the pandemic. This proportion declined to 21.3% in the second wave, indicating increased strain on medication delivery systems. Although not stratified by disease, hypertension—being a key NCD—was clearly affected by this shortfall. The disruption in medication continuity likely arose from logistical constraints such as transportation limits and pharmacy closures during lockdowns, which hindered patients’ access to antihypertensive drugs.

***AREA: Budget Allocation***
F. Budget constraints during the COVID-19 crisis had a limited effect on the delivery of NCD services, including hypertension. The article reported that 79.0% of healthcare facilities maintained their usual budget during the first wave, and 75.3% did so in the second wave. This relative financial stability allowed hypertension services to sustain essential operations, particularly medication provision and outpatient follow-ups. Because hypertension management is largely dependent on long-term pharmacological treatment, budget consistency played a crucial role in minimizing service disruptions.

***AREA: Supply Chain (Medical Supplies)***
G. The availability of medical supplies, including antihypertensive medications and related clinical tools, was largely preserved during the pandemic. Specifically, 75.3% of facilities reported regular supply levels during the first wave, and 74.1% in the second wave. The percentage of facilities reporting inadequate supplies increased slightly from 3.7% to 6.2%, reflecting some pressure on the supply chain but not a systemic failure. This supply resilience facilitated continued service provision for hypertension, as most essential medications and tools remained accessible to health centers even under crisis conditions.

***AREA: Strategic Planning Guidance***
H. The article emphasized that operational strategies should account for disease-specific service complexities. Hypertension, requiring fewer technical interventions, was identified as more amenable to stable service continuity under crisis conditions. The planning implication derived from this observation is that healthcare systems must tailor their emergency strategies based on the complexity of disease management—distinguishing between conditions like hypertension and more service-intensive diseases such as diabetes mellitus.

**Disease:** Diabetes Mellitus
**Crisis: COVID-19 Pandemic**

***AREA: Service Provision***
A. Several healthcare facilities restructured their outpatient services specifically for diabetes mellitus by organizing the care into four distinct clinical categories: Type I Diabetes Clinic, Poor-Controlled DM Clinic, Well-Controlled DM Clinic, and Health Education and Counseling Clinic. This reorganization allowed the facilities to better manage service delivery despite the redeployment of staff and resource shortages. The goal was to prioritize care based on clinical urgency and glycemic status, thereby reducing crowding and improving care delivery continuity. This strategic separation of patient groups enabled more efficient management of reduced human resources.

B. The article reported that fewer than 25% of healthcare facilities could provide essential diabetes services to over 95% of the target population. In the first wave, 18.4% of facilities reached this benchmark for follow-up clinical outcomes; in the second wave, this dropped further to 12.0%. These figures directly reflect the system's inability to ensure necessary service continuity for DM patients during periods of extreme healthcare strain, demonstrating a measurable collapse in monitoring capabilities, likely affecting diabetes complication rates.

C. Behavioral change counseling services, which are critical for the lifestyle management component of diabetes care, were severely disrupted. Only 11.7% of healthcare facilities could deliver this service to at least 95% of the intended population during the first wave. This declined to 9.3% in the second wave. The article attributes this reduction to the inability to conduct face-to-face interactions due to infection control policies, which disproportionately impacted DM patients who rely on structured counseling to manage dietary, exercise, and self-care routines.

D. Home visits, vital for DM patients who face mobility or transportation limitations, were significantly curtailed. During the first wave, only 6.5% of healthcare facilities achieved over 95% coverage for home visits; this fell to 4.1% in the second wave. The cause was the widespread redeployment of staff to COVID-19 duties and governmental restrictions on movement. This directly affected the ability to deliver insulin or glucose monitoring support to patients with advanced or poorly controlled diabetes, putting them at higher risk of acute complications.

***AREA: Digital Health and Telemedicine***
E. mHealth applications, such as MorBMA and DMS, were implemented to facilitate remote follow-ups and physician consultations. DM patients could upload their blood glucose values to these platforms. Physicians then conducted virtual consultations on scheduled follow-up dates. This system enabled the continuation of glycemic monitoring and medical oversight while minimizing COVID-19 exposure risk. The article emphasizes that these digital systems were particularly critical for patients with well-controlled diabetes who could be monitored without in-person visits.

F. For elderly or digitally illiterate DM patients, facilities assigned or coordinated with family members, community volunteers, or healthcare workers to assist with uploading data and using applications. This human support infrastructure was necessary to ensure that the most vulnerable patients could still access digital services and continue treatment without disruption.

G. The Pharmasafe application, specifically developed for chronic illness patients like those with DM, was used to support medication adherence and monitor for drug interactions. The software allowed family members and clinicians to track whether DM patients were taking medications correctly, thereby reducing the risk of treatment failure or complications due to drug noncompliance.

***AREA: Medication Delivery and Pharmacy Alternatives***
H. Drive-through and Fill-in Corner services were introduced for DM patients with stable conditions. These services allowed patients to pick up medications without entering healthcare facilities. Patients made virtual appointments for medical review and subsequently collected their prescriptions at designated outdoor points, reducing exposure risk while maintaining pharmacologic continuity.

I. Community drugstores, registered in advance with NHSO, dispensed diabetes medications to patients. These pharmacies were strategically selected based on proximity to patients’ homes to ensure continued access despite mobility restrictions. This model alleviated the load on hospitals and decentralized pharmaceutical services while maintaining oversight by the primary facility.

J. Medication delivery services were implemented in collaboration with Thailand Post and private logistics firms. These services allowed DM patients to receive essential medications at home. Funding for these services was covered either by NHSO or out-of-pocket by patients, thus preserving treatment continuity without requiring clinic visits during lockdown periods.

***AREA: Laboratory Testing and Monitoring***
K. Mobile laboratory services were initiated to allow DM patients to have blood samples collected from home for lab-based biomarker analysis, including glucose and HbA1c testing. However, this service was inconsistently available, particularly underutilized in outer Bangkok districts where patients lived beyond the service range.

L. Self-Monitoring of Blood Glucose (SMBG) was actively promoted. Patients used glucometers to measure their blood glucose at home and uploaded the results through LINE or mHealth platforms. Physicians reviewed these readings during virtual appointments to make dosage adjustments or other clinical decisions. This method preserved glycemic oversight during a period when routine laboratory testing was restricted.

***AREA: Workforce Allocation***
M. Staff responsible for DM care were reassigned to COVID-19 response tasks, leading to critical service shortages. During the second wave, 77.8% of healthcare facilities reported that DM clinic staff were partially or fully deployed to COVID-19 relief. This reassignment contributed directly to the decline in service indicators such as follow-up monitoring, medication counseling, and community outreach.

N. In facilities with a limited number of nurses for DM services—some having only a single staff member—the reassignment of that staff to pandemic duties led to near-total collapse of diabetes care services. For instance, one physician noted that their sole DM nurse had to work in multiple departments, rendering focused DM care impossible under pandemic conditions.

***AREA: Budget Allocation***
O. Despite the disruptions, budget allocations for DM care were largely preserved. In the first wave, 79.0% of healthcare facilities reported no reduction in budget, and this slightly decreased to 75.3% in the second wave. This stability enabled the continued implementation of innovations such as mHealth systems, mobile labs, and pharmaceutical delivery services that specifically supported diabetes care infrastructure.

***AREA: Strategic Planning and System Transformation***
P. Healthcare facilities that had pre-existing plans to implement telemedicine were able to accelerate their adoption due to the COVID-19 crisis. The pandemic acted as a catalyst for digital transformation, with one facility director stating that COVID-19 increased staff acceptance and institutional willingness to transition to virtual care models. This allowed DM services to adapt faster to the constraints of the crisis.

Q. Facility leadership was key in the successful transformation of DM services. Directors and senior nurses took active roles in breaking existing routines and implementing digital health services. Facilities with such leadership were able to organize training, plan new workflows, and secure stakeholder buy-in for diabetes-focused innovations, ensuring that DM patients remained connected to care throughout the crisis.

***AREA: Community and Inter-Sectoral Collaboration***
R. Facilities coordinated with community health workers and organizations to ensure DM patients continued to receive essential services like home-based glucose monitoring and medication delivery. These partners reported patient outcomes back to central facilities, where data was integrated into the health information system. This ensured continuity of clinical data, which was then used by physicians to adjust treatment plans.

S. Community partnerships allowed health systems to reach DM patients in lockdown zones or areas beyond direct service capacity. Such inter-sectoral collaboration was essential for maintaining the continuum of care, particularly in cases where institutional home visits were suspended due to pandemic restrictions.

**20. Article Title:** Patient experiences of diabetes and hypertension care during an evolving humanitarian crisis in Lebanon: A qualitative study

**Author, Publication year:** Willis, 2023

The article discusses cyclone, flood, and storm-related disasters as a combined category throughout, and does not stratify specific strategies or measures by disaster type. But the article does stratify the impact and relevant considerations by disease type

**List of the NCD diseases considered in this article:**
Hypertension, Diabetes Mellitus

**• List all of the crises and disasters considered in this article**
(Only non–inside health system crises like natural disasters, conflict, displacement)
• Main focus (included in analysis): Humanitarian crisis, Conflict (Syrian war), Displacement (Syrian refugees in Lebanon), Economic crisis in Lebanon, Beirut port explosion, COVID-19 pandemic

**Disease:** Hypertension
**Crisis: Combined Humanitarian Crisis (including conflict, displacement, economic collapse, COVID-19, and Beirut explosion)**

***DRUG SUPPLY***
A. Patients with hypertension reported that subsidized antihypertensive medications were inconsistently available at humanitarian-supported dispensaries. The economic crisis led to severe medication shortages, forcing patients to ration doses or forgo treatment. This disrupted adherence to lifelong management of hypertension. Some patients reduced prescribed doses, such as taking one pill daily instead of twice, in an attempt to prolong supply. Others substituted medical treatment with non-pharmaceutical remedies such as garlic and yogurt, which do not meet clinical guidelines for blood pressure control. The article presented this as a serious compromise in clinical care due to systemic pharmaceutical disruptions.

B. Due to shortages, patients frequently had to visit multiple facilities or pharmacies to locate antihypertensive drugs. This physical and logistical effort was exhausting for vulnerable individuals and risked treatment interruptions. A patient reported their family members searching over several days for specific medications unavailable in public facilities. These disruptions increased blood pressure instability and risked cardiovascular events, especially for those with comorbidities.

***SERVICE PROVISION***
C. Patients described inconsistent access to essential clinical services necessary for hypertension management, including regular consultations and monitoring tests such as blood pressure measurements and electrocardiograms (ECGs). One patient highlighted the benefit of a specific facility that provided ECG and lab tests at symbolic fees (~3000 LBP), while others indicated that such services were unaffordable or absent in most facilities, undermining effective clinical surveillance and dose adjustment.

D. The lack of integrated services led to fragmented hypertension care. Patients had to independently coordinate between consultation sites, laboratories, and pharmacies, increasing their treatment burden. For example, one patient had to consult a physician at one location, perform tests at a second, and purchase medication at a third, often without support for information transfer between providers. This disjointed care model increased the likelihood of missed follow-ups and mismanagement of hypertensive regimens.

***SUPPORT***
E. Family support was essential in ensuring patients continued their antihypertensive treatment. Family members searched for unavailable medications, accompanied patients to facilities, and adjusted household routines to reduce stress—an acknowledged contributor to blood pressure elevation. For instance, a caregiver asked children to remain quiet to avoid provoking hypertensive crises in the patient. This emotional and logistical support helped mitigate the structural health system deficiencies, but it placed additional pressure on already strained families.

***BUDGET PROVISION***
F. The economic crisis significantly limited patients' ability to afford antihypertensive medications, consultations, and diagnostic services. One patient emphasized they had to choose between essential living expenses and purchasing medication. Even where medications were nominally subsidized, hidden costs such as registration fees, test expenses, or transport posed significant barriers. These financial constraints often led to missed doses or delayed care, which increases the risk of hypertensive complications such as stroke or heart failure.

***INTER-SECTORAL COMMUNICATION***
G. Lack of coordination between health service providers disrupted care continuity for hypertensive patients. Patients had to manually transfer lab results and prescriptions between providers, which introduced inefficiencies and information loss. For instance, a patient explained how doctors at different clinics failed to share test results, requiring her to repeatedly explain her condition. This gap in communication increased patients’ workload and reduced clinical oversight, threatening proper disease control.

***STRATEGIC OR OPERATIONAL PLANNING***
H. The article recommended the development of shared information systems and standardised treatment protocols for hypertension in humanitarian contexts. These would facilitate continuity of care by ensuring consistent management across different providers and time points. Without such systems, patients experienced frequent breaks in treatment, especially when transitioning between emergency and routine care settings. The absence of unified protocols and medical records made it difficult for providers to make informed decisions, further destabilizing hypertension control.

I. The recommendation to appoint a designated social worker or case manager to assist patients in navigating the health system was highlighted as a strategic solution to relieve the burden on hypertensive patients. Such personnel would help manage scheduling, medication procurement, and inter-facility coordination. This role was considered essential due to the high cognitive and logistical demands placed on patients under current fragmented service models.

***PSYCHOSOCIAL SUPPORT***
J. Hypertensive patients were consistently advised by providers to reduce stress and emotional disturbances, as these were believed to exacerbate their blood pressure. However, patients reported that the compounded pressures of financial hardship, displacement, and insecurity made it extremely difficult to avoid stressors. Some explicitly stated that anger, sadness, or fear were causes of their hypertension. The contradiction between clinical advice and lived reality led to feelings of helplessness and sometimes disengagement from care.

***MONITORING AND TESTING***
K. Regular blood pressure monitoring was inconsistent due to cost barriers and limited availability of diagnostic services at primary care facilities. Some patients prioritized purchasing medication over testing, undermining effective disease control. Others could not afford home monitoring devices or the fees for laboratory services. The lack of accessible monitoring delayed detection of hypertensive complications and hindered timely therapeutic adjustments.

**Disease:** Diabetes Mellitus
**Crisis: Combined Humanitarian Crisis (including conflict, displacement, economic collapse, COVID-19, and Beirut explosion)**

***DRUG SUPPLY***
A. Patients with diabetes mellitus reported substantial disruptions in insulin and oral hypoglycemic agent availability. One participant explicitly stated that both she and her husband required diabetic medications, but due to financial constraints and shortages at the facility, she was not able to access full prescriptions. She declared, “That’s why we always have a shortage in medicine. I’m not taking all my medicine.” This directly interferes with glycemic regulation and increases the risk of both acute (e.g., diabetic ketoacidosis) and chronic complications (e.g., retinopathy, nephropathy).

B. A caregiver of two diabetic siblings stated that due to lack of money, they could afford insulin but not testing. This forced prioritization of medication over glucose monitoring reveals the fragmented nature of diabetic care during crisis. Without appropriate monitoring, treatment adjustments cannot be made, which exposes patients to hypoglycemic or hyperglycemic events.

C. Border closures during the COVID-19 pandemic halted cross-border procurement of medications from Syria, which patients previously relied on due to lower prices. One patient noted that before the pandemic, her family used to send someone to Syria to get insulin. When that option became inaccessible, they were forced to purchase it locally at much higher cost. This made medication adherence even more erratic and increased financial strain, creating further barriers to sustained treatment.

***SERVICE PROVISION***
D. Patients reported having to visit multiple health providers to fulfill routine diabetes care needs. One patient explained that she consulted physicians in one facility but had to go to private laboratories for HBA1C testing and other blood work. Another participant described having to navigate more than ten different doctors due to dissatisfaction with care and lack of effective treatment. This highlights the lack of integrated diabetes care, which is essential for proper disease management. Fragmented service delivery leads to delays, discontinuity, and inconsistent clinical decision-making, all of which negatively affect glycemic control.

E. Some patients received general dietary guidance (e.g., “avoid sugar,” “eat less”), while others received no advice at all. A patient explicitly stated: “This is who I am. I cannot resist.” This reflects inadequate implementation of structured nutritional counseling. Dietary adherence is a core component of diabetes management, and the absence of structured, affordable dietary support prevents effective control of blood glucose levels.

F. One female patient had her blood sugar tested by her diabetic mother and a pharmacist but did not see a physician for more than ten years. Only when she developed severe leg pain requiring surgery did she seek clinical care. This delay in diagnosis and absence of preventative service provision contributed to the progression of the disease to the point of amputation. The delay illustrates the absence of effective screening protocols and early intervention measures in the care system.

***SUPPORT***
G. Family members undertook critical roles in securing medication and managing care logistics. One son reported using money from his own limited earnings to purchase insulin for his mother, even though this made it difficult to provide food for the family. Another caregiver coordinated with relatives abroad to send insulin from outside Lebanon. These actions underscore the collapse of institutional support and the shifting of health system responsibilities onto households.

H. Emotional and practical support also emerged as central to diabetes care. Caregivers described adapting home environments to prevent stress-induced glycemic instability. One caregiver stated she instructed her children to remain quiet so as not to agitate her diabetic husband. Psychological stress is recognized as a contributor to poor glycemic control, and these behavioral adaptations, while informal, served as compensatory mechanisms for absent mental health or psychosocial support from the healthcare system.

***BUDGET PROVISION***
I. Nearly all diabetic patients and caregivers cited cost as a major barrier. One patient said they had to “sell belongings” to afford insulin. Another stated, “You can’t keep up with paying for doctors, laboratories, and medicine.” These economic constraints disrupted medication adherence, delayed routine checkups, and prevented the purchase of necessary supplies such as glucose testing strips, thus impairing daily disease management.

J. The article described a case in which a patient needed urgent surgery for a diabetes-related complication (amputation). Approval for subsidized surgery was granted but revoked because the physician postponed the operation. The organization deemed the rescheduled procedure no longer emergent, leaving the patient unable to pay for care. This indicates structural failures in budget allocation mechanisms and eligibility frameworks, which leave critical diabetic care contingent on unpredictable definitions of “emergency.”

***INTER-SECTORAL COMMUNICATION***
K. Patients had to self-manage the exchange of medical information between different service providers. One patient noted: “She would tell me to do certain tests and to bring her the results.” Another explained how they had to continually repeat their history to different physicians, increasing the likelihood of error, miscommunication, and treatment fragmentation. Lack of an integrated health information system caused unnecessary repetition, delayed treatment decisions, and compromised continuity of diabetes care.

***STRATEGIC OR OPERATIONAL PLANNING***
L. The article recommended developing a shared digital platform for storing patient records and standardizing diabetes care protocols. The absence of such systems forced patients to manually coordinate their own care, which became unmanageable, especially for elderly or illiterate individuals. Standardization would reduce delays, unify treatment regimens, and help in early identification of high-risk patients, thereby improving outcomes in crisis settings.

M. Introducing a designated support figure, such as a social worker or care coordinator, was proposed to alleviate patients’ administrative and logistical burdens. This role would assist with appointment scheduling, navigating referrals, and locating medication sources. Such institutional support is particularly critical in the context of diabetes, where daily management requires consistent multi-source coordination, and where failure leads to severe health deterioration.

***PSYCHOSOCIAL SUPPORT***
N. Patients described psychological exhaustion, anxiety, and depression stemming from their inability to maintain diabetes control. One patient stated that not being able to provide food or medicine for his children and himself made him “sad” and incapable of controlling his blood sugar. Another reported that fear, sadness, and traumatic events were the cause of his diabetes onset. These psychological stressors directly interfere with disease self-management, impair adherence, and contribute to acute destabilization of glucose levels.

***MONITORING AND TESTING***
O. Patients lacked access to essential monitoring tools. One caregiver explained that she could afford insulin but not HBA1C testing. Another patient owned a glucometer but could not afford lancets or test strips. Inadequate monitoring prevents timely adjustments in therapy and increases the risk of unrecognized hyperglycemia or hypoglycemia. In one case, this lack of monitoring and delayed response led to leg ulceration and eventual amputation.

***DIETARY MANAGEMENT SUPPORT***
P. Dietary advice was inconsistently provided and rarely actionable due to food insecurity. Some patients acknowledged the need for dietary restrictions but said they could not afford recommended foods. One stated that while she tried to eat smaller portions, financial constraints and cultural dietary habits made it difficult. Lack of structured dietary support and inability to follow clinical dietary recommendations contributed to poor glycemic control across cases.

**21. Article Title:** Managing Projects Addressing Non-Communicable Diseases: Operational Guidelines for Field Staff

**Author, Publication year:** Aebischer Perone, 2018

The article discusses cyclone, flood, and storm-related disasters as a combined category throughout, and does not stratify specific strategies or measures by disaster type. But the article does stratify the impact and relevant considerations by disease type

**List of the NCD diseases considered in this article:** Hypertension, Heart Failure, Diabetes Mellitus, Chronic Obstructive Pulmonary Disease (COPD), Asthma, Epilepsy, Depression, Schizophrenia, Anxiety

**• List all of the crises and disasters considered in this article**
(Only non–inside health system crises like natural disasters, conflict, displacement)
• Main focus (included in analysis): Hurricanes, Earthquakes, Tsunamis, Flooding, Bushfires, Tornadoes

**Disease:**Hypertension

**DRUG SUPPLY**

**A. Continuous access to antihypertensive medications**
The article states that for hypertensive patients, uninterrupted treatment is critical because sudden discontinuation of antihypertensive medications can result in rebound hypertension, which significantly increases the risk of cardiovascular events such as myocardial infarction, stroke, and hypertensive emergencies. Ensuring continuous access through pre-existing health systems or humanitarian service delivery models prevents such adverse outcomes. It emphasizes using medicines listed on national or WHO essential medicine lists to enhance affordability, availability, and logistical predictability during humanitarian interventions. These lists typically include medications such as hydrochlorothiazide, amlodipine, and enalapril, which are suitable for basic and long-term blood pressure control.

**B. Establishment of buffer stock**
The article explicitly recommends maintaining a buffer stock of essential NCD medicines—including those for hypertension—in crisis settings where the medical supply chain is fragile. A stockpile prevents disruption in antihypertensive treatment during logistical delays or surges in demand, which are common in armed conflicts or natural disasters. Hypertensive patients rely on consistent medication to avoid end-organ damage; even a few days of interruption can cause dangerous spikes in blood pressure.

**C. Emergency supply systems tailored to hypertension**
Hypertension is classified as a priority NCD within the document. It mandates designing emergency health kits and supply strategies with this condition in mind. The kits must contain commonly used antihypertensives suitable for immediate treatment of hypertensive crises (e.g., labetalol, nifedipine) and stable maintenance therapy (e.g., ACE inhibitors, diuretics). Rapid distribution and context-based forecasting are required to ensure these supplies reach crisis-affected populations promptly.

**D. Rational Drug Prescription training**
Incorrect medication choice or dosing in hypertensive patients may lead to ineffective control or side effects (e.g., hypotension, electrolyte imbalance). The article insists that prescribers must be trained to select cost-effective, effective antihypertensives based on the local formulary and patient history. This is essential in resource-limited humanitarian settings where advanced diagnostic support may be unavailable. Prescribers should be aware of national protocols and must be trained to adjust or simplify regimens as needed under crisis conditions.

**E. Cold chain support (where applicable)**
While most hypertension medications do not require cold storage, the article includes cold chain infrastructure under drug supply preparedness, noting that medicines requiring specific storage conditions should be maintained properly to ensure potency. This principle reinforces supply chain robustness and applies indirectly to hypertension management when storage-sensitive combination medications are used.

**F. Restriction on introducing unsustainable new drugs**
The document warns against introducing new antihypertensive medications during a crisis if long-term continuity cannot be guaranteed. This is because once patients are stabilized on a new agent, withdrawing or substituting it post-crisis can destabilize blood pressure control, leading to acute events. Program planners must instead rely on medicines that are part of the long-term national supply chain.

**G. Avoidance of substandard or falsified products**
Crises often lead to a breakdown in regulation, allowing counterfeit or substandard medicines to enter supply chains. The article urges implementing quality assurance systems to protect hypertensive patients from receiving ineffective drugs, which can falsely reassure patients while blood pressure remains uncontrolled, resulting in delayed or missed care for complications.

**SERVICE PROVISION**

**A. Life-saving care and stabilization of hypertensive emergencies**
In the acute phase of a crisis, patients may present with hypertensive encephalopathy, stroke, or chest pain. The article emphasizes the need for triage systems and immediate interventions using fast-acting antihypertensives or referrals. Hypertension management in emergencies must prioritize patients in crisis through context-specific protocols to prevent irreversible outcomes such as renal failure or death.

**B. Continuum of care for pre-diagnosed hypertensive patients**
For patients already on treatment, any disruption—due to displacement, loss of records, or facility destruction—can lead to disease destabilization. The article stresses establishing routine follow-up mechanisms, including scheduled consultations, to maintain control over blood pressure and reduce the risk of complications. These services must be adapted to available infrastructure, for instance, using mobile clinics or outreach programs in hard-to-reach populations.

**C. Referral pathways to higher-level care**
Management of complications such as hypertensive heart disease or stroke requires referral to secondary or tertiary care. The article describes creating functional and contextually feasible referral systems, with transport support and cost mitigation. This ensures that individuals experiencing hypertensive crises receive appropriate treatment, including diagnostic imaging or intensive care, which are beyond the scope of basic primary services.

**D. Use of national guideline-aligned clinical management tools**
Standardized hypertension protocols support safe and efficient treatment across multiple providers. These tools cover diagnosis, treatment thresholds, medication choices, and referral criteria. The article recommends their use to maintain consistent quality of care, especially when health personnel are overburdened or turnover is high.

**E. Triage systems to detect hypertensive emergencies**
Patients must be rapidly assessed at the point of care to determine urgency. The article instructs implementation of triage systems to identify hypertensive urgency/emergency (e.g., systolic BP >180 mmHg with organ damage) for immediate action. This minimizes the risk of sudden cardiac events or cerebral complications.

**F. Follow-up protocols based on stability**
The article introduces a tiered system based on patient condition: those with uncontrolled hypertension or recent complications should receive weekly reviews, while stable individuals may be reviewed every 3–6 months. This individualized frequency of follow-up ensures appropriate monitoring while optimizing limited resources.

**G. Patient-tracking mechanisms**
To ensure continuity for mobile or displaced populations, patient-tracking systems—such as paper appointment cards or mobile-based reminders—are recommended. These help retain hypertensive patients in care even as they move between settings or providers, thus preventing attrition and unmonitored blood pressure elevation.

**SUPPORT**

**A. Community health worker involvement**
Community health workers can monitor hypertensive patients, remind them of appointments, assist with lifestyle counseling, and detect early warning signs. The article recognizes their role in ensuring follow-up and providing culturally relevant health education, thus extending the reach of the formal health system into vulnerable communities.

**B. Outreach by health committees**
Health committees are advised to locate hypertensive individuals who are unable or unwilling to access services. These may include elderly, disabled, or marginalized patients. This mechanism helps reduce preventable complications due to care gaps in hidden populations.

**C. Peer and community-based support structures**
Support groups foster shared learning about hypertension management, enhance adherence through encouragement, and provide psychosocial support. These groups help patients understand dietary, behavioral, and medication adherence needs through peer reinforcement.

**PREPARATION OF STRATEGIC OR OPERATIONAL PLANS**

**A. Inclusion of hypertension burden in needs assessments**
Planning should begin with a clear understanding of hypertension prevalence among the crisis-affected population. This ensures that resource allocation and service design match the real burden of disease and are not skewed by acute or infectious conditions that may dominate attention.

**B. Targeting hypertension as a specific objective**
In operational planning, hypertension must be treated as a stand-alone priority among NCDs. The article emphasizes defining outcome-based goals (e.g., reducing acute hypertensive events, ensuring 80% treatment continuity) and allocating resources accordingly.

**C. Crisis-phase appropriate intervention planning**
Response planning must include mechanisms to provide antihypertensive medications, consultations, and lab services, even in the absence of regular health infrastructure. The article advises defining the minimum service package necessary for effective hypertension care in the given phase of crisis.

**D. Pre-definition of referral and stabilization processes**
Operational plans must include clear protocols for managing hypertensive crises, including clinical thresholds, required medicines, referral points, and transport solutions. These systems must be tested and revised as needed to remain functional during fluctuating crisis dynamics.

**TRAINING OF FORCES**

**A. Training in hypertension diagnosis and treatment**
Medical staff must be trained to recognize different stages of hypertension, diagnose it using proper methods (e.g., multiple BP readings), and start appropriate treatment. The article mandates this training as essential for effective chronic disease control in unstable environments.

**B. Prescriber training for rational antihypertensive use**
Training in rational drug prescription ensures that staff select cost-effective, locally available, and clinically suitable medications for hypertension. This minimizes side effects, increases adherence, and ensures sustainability of treatment even after the emergency response ends.

**C. Task-sharing with nurses and non-physician staff**
The article encourages using trained non-physician health workers to follow up with stable hypertensive patients, freeing physicians to handle complex cases. This increases service coverage and ensures timely monitoring of a broader patient base.

**CREATION OF STORAGE RESOURCES**

**A. Establishment of antihypertensive medication buffer stocks**
The article mandates the development of stockpiles specifically for essential NCD drugs. For hypertensive patients, a lack of drug availability due to delayed procurement or conflict-related supply cuts may result in acute complications. Maintaining a buffer of oral antihypertensives prevents such events.

**B. Supply system design and cold chain**
While most hypertension drugs are shelf-stable, overall readiness requires including hypertension medications in contingency storage plans. Stocking must be organized, secure, and temperature-controlled where needed, with inventory monitoring to ensure availability during surges.

**IMPROVEMENT OF INTER-SECTORAL COMMUNICATIONS**

**A. Coordination with health clusters and national systems**
The article highlights the importance of integrating hypertension interventions into the broader humanitarian and local health frameworks. This allows sharing of resources, harmonization of treatment protocols, and long-term sustainability after withdrawal of humanitarian actors.

**B. Embedding hypertension care in host systems**
To avoid fragmentation, hypertension services must align with national priorities and be absorbed into the public system. This supports continuity post-crisis and avoids duplicative or temporary programs that collapse upon donor exit.

**PROVISION OF BUDGET**

**A. Budget inclusion for drugs, diagnostics, and staffing**
Budget lines must include recurring costs of hypertension care—such as medications, blood pressure monitors, and provider time—to ensure feasibility of service delivery. The article warns that excluding such costs results in interrupted services and increased preventable morbidity.

**B. Phase-appropriate funding allocation**
Resources must be allocated based on the phase of the crisis. In the acute phase, funding is needed for stabilization, emergency medications, and triage systems. In protracted phases, funding must support long-term follow-up, adherence monitoring, and integrated chronic care services.

**OTHER**

**A. Use of patient-held records for mobility and continuity**
Patient-held records, such as booklets or health passports, enable hypertensive patients to retain critical clinical information as they move between facilities. This improves diagnosis accuracy and continuity, especially when digital systems are absent or destroyed.

**B. Patient education on hypertension management**
The article mandates patient education covering medication adherence, salt intake, stress management, and physical activity. Educated patients are more likely to manage their disease successfully in unstable conditions and less likely to require hospitalization due to uncontrolled hypertension.

**Disease:** Heart Failure

***DRUG SUPPLY***

A. Heart failure, as part of cardiovascular diseases, is explicitly prioritized in the selection of essential NCD medications to be maintained during crises. The document mandates the availability of essential cardiovascular medications listed in national formularies or the WHO model list to prevent deterioration of heart failure conditions. The justification provided is that treatment interruptions can rapidly worsen the condition and lead to life-threatening decompensation, particularly where access to emergency or advanced care is limited. Maintaining drug availability ensures adherence to standard protocols and reduces preventable mortality.

B. In humanitarian contexts, non-proprietary (generic) cardiovascular medications are prioritized to ensure sustainability and cost-efficiency in heart failure management. This decision is based on the need to provide long-term care in resource-constrained settings. It emphasizes that health professionals and patients must be trained to trust and effectively use generic medicines, which are necessary due to limited supply chains and budgets.

C. Emergency buffer stocks of heart failure medications must be established to mitigate the risk of sudden supply chain disruptions due to insecurity, destruction of infrastructure, or blockade. Such stocks are essential for maintaining consistent treatment and preventing acute exacerbations of heart failure symptoms, such as pulmonary edema, which require immediate pharmacologic intervention.

***SERVICE PROVISION***

D. Triage protocols must prioritize symptomatic heart failure patients, particularly those with acute presentations such as cardiac chest pain, shortness of breath, or signs of fluid overload. Immediate stabilization, including pharmacologic intervention and potential referral, is mandated. The guidance emphasizes that acute cardiovascular conditions like myocardial infarction—a common comorbidity and decompensating event in heart failure—require direct triage attention to avoid fatal outcomes.

E. The guideline mandates the establishment of referral pathways for patients suffering from myocardial infarction or stroke, which are complications frequently encountered in advanced heart failure. In cases where referral to higher levels of care is not feasible due to security constraints, palliative care approaches are to be initiated. This demonstrates the critical need to have flexible systems tailored to the availability of transport and hospital services to preserve life or alleviate suffering.

F. Clinical management tools adapted to field conditions must be implemented for heart failure. These tools include algorithms for the diagnosis, monitoring, and pharmacologic treatment of symptomatic heart failure and are based on simplified national or WHO guidelines. The importance of having context-adapted tools is underscored by the need to deliver timely interventions in high-pressure environments and with limited diagnostic capacity.

***SUPPORT AND CONTINUUM OF CARE***

G. Heart failure patients must receive uninterrupted follow-up care to prevent decompensation and complications such as renal failure or stroke. Continuum of care involves structured clinical follow-up, access to chronic medication, patient tracing systems, and individualized treatment monitoring. The document highlights that missed follow-up and discontinuation of medications—especially diuretics or ACE inhibitors—can rapidly worsen outcomes in heart failure cases.

H. Mobile populations living with heart failure must be supported through personalized contingency planning. These measures include supplying additional doses of cardiovascular medications and informing patients of health facility access points along displacement routes. These plans are essential because heart failure requires continuous pharmacologic therapy, and treatment gaps can lead to acute fluid retention and cardiac arrest.

***TRAINING OF FORCES***

I. The guidelines specify that all health-care workers should receive specialized training in the diagnosis and management of cardiovascular diseases, including heart failure. Training programs must be adapted to local settings and emphasize emergency recognition of decompensated heart failure and administration of guideline-directed medical therapy. The rationale is that untrained staff may fail to identify or appropriately manage acute heart failure, resulting in avoidable morbidity and mortality.

***CREATION OF STORAGE RESOURCES***

J. Cold chain systems are to be used when necessary to preserve the potency of heat-sensitive cardiovascular drugs, which may be required in heart failure emergencies. This includes certain injectable formulations or medicines subject to degradation under high temperatures. The storage infrastructure must be coordinated with broader cold chain needs, such as those for vaccines or insulin, ensuring logistical efficiency.

***IMPROVEMENT OF INTER-SECTORAL COMMUNICATIONS***

K. The integration of heart failure services into national and existing health systems is mandated to support sustainability. Coordination improves consistency in medication protocols, alignment with referral networks, and shared patient data, ensuring that heart failure care is not isolated from broader service delivery systems.

L. Collaboration with local and international partners is encouraged to ensure efficient resource utilization, especially in providing chronic care for heart failure. These partnerships enable task sharing, co-financing of medication supply, and access to specialized services for complicated cardiovascular cases.

***PROVISION OF BUDGET***

M. Budget mobilization strategies should prioritize funding for cardiovascular services, including those related to heart failure. Given the life-threatening nature of decompensated heart failure, resources must be directed to ensure uninterrupted availability of medicines, diagnostic tools, and trained personnel in crises. The guidance stresses that lack of funding can critically impair the ability to provide even baseline management for heart failure, leading to a surge in mortality.

***STRATEGIC OR OPERATIONAL PLANS***

N. Heart failure is identified as a priority NCD within the broader operational planning framework. Strategic plans must include it as one of the core diseases requiring intervention during crises, particularly due to its prevalence, high mortality risk, and the feasibility of treatment with standard protocols.

O. The selection of heart failure for prioritized intervention is based on assessed population needs, existing capacities, and the context. The guidelines emphasize that organizations should adapt their NCD service plans to ensure the inclusion of heart failure where its burden is significant, considering it both a public health necessity and a feasible target for intervention in crisis settings.

**Disease:** Chronic Obstructive Pulmonary Disease (COPD)
**Crisis: All Humanitarian Crises (including armed conflict, natural disasters, disease outbreaks, displacement, violence)**

***DRUG SUPPLY***

A. Essential COPD medicines, including bronchodilators and corticosteroids, are identified in the document as critical in emergency settings because acute exacerbations can rapidly result in hypoxic respiratory failure. These medications must be made available continuously to prevent worsening of pulmonary function and mortality. The text explicitly refers to maintaining a minimum stock of essential medications for chronic respiratory diseases and underlines that treatment interruptions in COPD can have fatal consequences due to inability to self-resolve exacerbations without pharmacological intervention.

B. The use of non-proprietary (generic) inhaled medications for COPD must be promoted during crises, as they are more cost-effective and logistically feasible to procure in bulk. However, the document also addresses that both health workers and patients must be educated in their effective use, especially since there may be mistrust or unfamiliarity with generic brands, which can result in non-adherence or incorrect administration in COPD patients.

C. Pre-stocked emergency COPD medicines, including short-acting bronchodilators and inhalation devices, must be included in essential medical kits and stored in facilities where displaced populations are likely to be treated. The need for buffer stocks is explicitly stated to ensure uninterrupted treatment during supply chain interruptions caused by insecurity or transportation collapse. The document makes clear that COPD patients deteriorate quickly without rescue inhalers, requiring emergency planning in stockpiling.

***SERVICE PROVISION***

D. Triage systems must explicitly prioritize COPD patients presenting with signs of acute exacerbation, such as dyspnea, wheezing, and hypoxia. The guideline states that respiratory distress in these patients requires rapid identification and pharmacological stabilization. If these patients are not managed quickly, they may progress to respiratory failure in settings where ventilatory support is unavailable.

E. Referral systems must accommodate COPD patients with exacerbations requiring escalation to oxygen therapy or advanced airway management. However, the document clarifies that where referral is not possible due to contextual barriers like armed conflict or flooding, local palliative and symptom relief measures must be implemented to manage breathlessness and reduce suffering.

F. Management tools adapted to local resources must include simplified COPD care algorithms for health staff. These tools should incorporate standard dosing for bronchodilators, symptom assessment, and warning signs for escalation. The document emphasizes that having pre-designed and easy-to-follow COPD protocols in primary health care can reduce the risk of misdiagnosis or under-treatment during the chaos of emergency care delivery.

***SUPPORT AND CONTINUUM OF CARE***

G. COPD patients must receive regular follow-up care even during displacement or crisis phases, due to their dependency on consistent medication and high susceptibility to relapse. The guideline states that failure to ensure continuity in inhaled maintenance therapy leads to worsening airway obstruction, hospitalizations, and increased mortality, particularly in populations exposed to environmental triggers like dust, cold, or poor air quality.

H. COPD patients must be supported to carry their own clinical summaries or patient-held health records. The document specifies this requirement for mobile populations to allow the next provider or facility to resume inhalation therapy correctly, avoiding inappropriate medication switches or delays that could lead to acute attacks in already compromised respiratory conditions.

***TRAINING OF FORCES***

I. Training programs must include modules on identification and acute management of COPD, particularly the recognition of exacerbations and indications for bronchodilator therapy. Staff must be trained to distinguish COPD from other conditions like asthma or tuberculosis, which have different treatments. The guideline highlights that non-specialist providers in emergencies may misclassify or undertreat COPD unless guided by targeted training materials and decision-support tools.

***CREATION OF STORAGE RESOURCES***

J. Inhalation devices (inhalers, spacers) and oxygen cylinders must be included in emergency storage strategies for facilities likely to encounter COPD patients. The document emphasizes that treatment for COPD is not complete without delivery devices, and failure to secure these in supply chains leads to ineffective care despite availability of medication.

K. COPD drugs and delivery devices must be pre-positioned in peripheral facilities and mobile units that serve displaced populations or isolated settlements. The document details this measure as necessary to ensure rapid access to care when mobility is restricted or centralized services are overburdened or nonfunctional.

***IMPROVEMENT OF INTER-SECTORAL COMMUNICATIONS***

L. COPD care protocols and treatment access must be harmonized across all actors, including ministries of health, NGOs, and field clinics. The document describes how fragmentation of care for chronic respiratory diseases results in conflicting drug choices, redundant procurement, and gaps in service continuity. Integration ensures that COPD management does not fall outside of system-wide planning for non-communicable diseases.

***PROVISION OF BUDGET***

M. Specific financial allocations must be made to guarantee continued access to COPD medicines and devices in all phases of crisis response. The document states that COPD care is often neglected due to low visibility, but failure to allocate funds results in unmanaged exacerbations and increased case fatality. Funding must also include patient education tools and health worker capacity building to avoid long-term complications.

***STRATEGIC OR OPERATIONAL PLANS***

N. COPD is listed among the core NCDs requiring explicit inclusion in operational plans. It is noted that due to the environmental sensitivity of COPD patients (e.g., to smoke, dust, indoor air pollution), strategic plans must anticipate spikes in exacerbations following disasters such as bushfires or dust storms, and include ready-to-implement interventions accordingly.

O. Operational plans must define what constitutes minimum acceptable care for COPD, such as access to bronchodilators, follow-up mechanisms, emergency referral procedures, and health worker preparedness. The guideline underlines that COPD patients are especially vulnerable in camp-like settings where air quality is poor and medical resources are limited.

**Disease:** Asthma
**Crisis: All Humanitarian Crises (including armed conflict, natural disasters, disease outbreaks, displacement, violence)**

***DRUG SUPPLY***

A. Bronchodilators and corticosteroids are identified in the document as essential medicines for asthma management that must be prioritized in crisis response. The guideline explains that acute asthma exacerbations, when untreated, may rapidly progress to severe respiratory distress or fatal airway obstruction. These medications prevent airway inflammation and bronchoconstriction, thus reducing emergency presentations. Their inclusion in emergency health kits is classified as life-saving and mandatory.

B. Generic (non-proprietary) versions of inhaled asthma medications must be used where possible to maintain affordability and ensure widespread availability. The text explains that branded inhalers are often unavailable or unaffordable in prolonged crisis settings. Switching to generics is recommended but must be coupled with provider training and patient education to avoid misuse or non-compliance, as incorrect technique or mistrust in generics may compromise treatment efficacy.

C. Asthma medications and delivery devices, including metered-dose inhalers and spacers, must be pre-stocked at all facility levels, particularly those serving displaced populations. The document highlights that stock outages are especially dangerous for asthma patients who depend on prompt relief of symptoms to prevent respiratory failure. The establishment of buffer stock mechanisms is intended to maintain continuity of care despite logistical or security-related interruptions in supply.

***SERVICE PROVISION***

D. Patients presenting with acute asthma attacks must be triaged as medical emergencies requiring immediate bronchodilation and, if necessary, supplemental oxygen. The guideline includes asthma as one of the critical respiratory emergencies that cannot be delayed in treatment due to the risk of rapid airway compromise. Triage systems must be trained to recognize and act on signs of acute bronchospasm.

E. Referral systems must accommodate asthma patients who are unresponsive to initial inhaled therapy and require escalation, including continuous nebulization, corticosteroid injection, or oxygen support. However, the document also notes that if referral is not possible, structured palliative or supportive protocols must be implemented to manage symptoms locally. This may include repeated inhalation doses and anti-inflammatory therapy to stabilize patients until transfer is possible.

F. The article mandates the use of clinical algorithms specifically adapted for asthma management at the primary health care level. These include standardized criteria for diagnosis, initial treatment, and follow-up, as well as protocols for identifying severe attacks. Such tools are vital because in emergencies, non-specialist staff are often required to make treatment decisions, and without guidance, asthma may be underdiagnosed or inappropriately treated.

***SUPPORT AND CONTINUUM OF CARE***

G. The guideline requires asthma patients to be maintained on regular follow-up schedules, with consistent access to maintenance medication to prevent exacerbations. It emphasizes that disruption in care — such as withdrawal of corticosteroids — increases the likelihood of acute attacks, particularly in unstable environments with dust, cold, or poor air quality. Therefore, asthma patients must remain in structured treatment systems even during crises.

H. Patients with asthma who are displaced or mobile must be given extra medication supplies, including inhalers, and must be informed of where to access services along their migration path. This ensures continuity of treatment in populations that are at risk of being cut off from formal health systems. Lack of access to bronchodilators and corticosteroids in such scenarios is associated with increased emergency episodes and fatalities.

***TRAINING OF FORCES***

I. Medical personnel must be trained in recognizing the clinical presentation of asthma and administering treatment using inhalers, spacers, and corticosteroids. The article specifically notes that asthma symptoms — wheezing, shortness of breath, chest tightness — must not be mistaken for infections like pneumonia or tuberculosis. Proper training in differential diagnosis, as well as emergency intervention, ensures that patients receive appropriate care during both acute attacks and maintenance phases.

***CREATION OF STORAGE RESOURCES***

J. Inhaled medications and their delivery devices must be stored under appropriate conditions and pre-positioned in facilities most likely to encounter displaced populations. The guideline explains that inhalers are only effective if properly stored, especially in extreme temperatures, and that absence of delivery devices renders stored medication unusable. Therefore, inhalers and spacers must be part of both the drug and equipment inventory.

K. Strategic stockpiling of asthma treatment kits must be carried out in anticipation of surges in respiratory conditions, especially in areas vulnerable to environmental triggers such as bushfires, dust storms, or poor ventilation. The document insists that asthma medications be included in centralized and decentralized storage plans to ensure availability in both fixed and mobile health units.

***IMPROVEMENT OF INTER-SECTORAL COMMUNICATIONS***

L. Asthma treatment protocols must be standardized across actors—ministries of health, humanitarian organizations, and international agencies—to avoid discrepancies in care. The article explains that lack of alignment can result in conflicting treatment regimens, patient confusion, and stock incompatibility. Coordinated planning also enables pooling of resources and training efforts, particularly important for diseases like asthma that require rapid response during acute events.

***PROVISION OF BUDGET***

M. Asthma care, including the purchase of medications, delivery devices, and staff training, must be included in the health budgets of humanitarian operations. The guideline emphasizes that failure to allocate resources to asthma leads to preventable morbidity and mortality, especially when a single inhaler or corticosteroid course could avert hospitalization or death. The low cost and high impact of asthma treatment necessitate prioritization in financial planning.

***STRATEGIC OR OPERATIONAL PLANS***

N. Asthma is formally identified as one of the core NCDs to be addressed in emergency operational frameworks. It is categorized as a high-priority condition due to its potential for sudden exacerbation and its manageable nature when appropriately treated. Therefore, asthma interventions must be embedded in both acute response and protracted phase planning.

O. Operational plans must define specific service delivery benchmarks for asthma care, such as immediate access to short-acting bronchodilators, structured outpatient follow-up, and availability of emergency oxygen where feasible. These benchmarks must be tailored to facility capacity and population risk profiles, with special attention to refugee camps, urban slums, and disaster-affected regions where air quality is compromised.

**Disease:** Epilepsy
**Crisis: All Humanitarian Crises (including armed conflict, natural disasters, disease outbreaks, displacement, violence)**

***DRUG SUPPLY***

A. Anti-epileptic medications (AEMs), such as phenobarbital and carbamazepine, must be secured without interruption during crises because people with epilepsy are highly vulnerable to sudden withdrawal effects. The article stresses that any gap in medication access can precipitate uncontrolled seizures, status epilepticus, physical injuries, or sudden unexpected death in epilepsy (SUDEP). In humanitarian emergencies, where infrastructure may collapse and routine services are interrupted, these medications become life-saving rather than elective, and their uninterrupted supply is deemed non-negotiable.

B. The article mandates the procurement and distribution of non-proprietary (generic) AEMs to ensure affordability and consistent availability across humanitarian settings. The rationale for this measure is that patented or brand-name versions are frequently unaffordable or logistically inaccessible during crises. However, effective substitution demands careful communication with patients and providers to ensure trust in efficacy and adherence. Without this, patients may abandon treatment due to perceived inferiority of generics, increasing seizure frequency and associated complications.

C. Emergency reserves of AEMs must be integrated into facility-level buffer stock systems, especially in regions exposed to security threats or displacement. The document emphasizes that even a short interruption in access can lead to seizure clusters, repeated hospital visits, and worsening neurological status. For patients stabilized on chronic therapy, a single missed dose may provoke convulsions or injury in unsafe surroundings, such as unstable housing or transport conditions.

***SERVICE PROVISION***

D. Seizures are classified in the article as high-priority emergencies in the triage system, particularly when prolonged or clustered. Facilities must be equipped with protocols to recognize convulsions and provide immediate access to emergency AEMs to avert progression to status epilepticus. In many humanitarian contexts where advanced neurological services are unavailable, timely triage and rapid medication delivery are the only accessible interventions.

E. Standardized clinical management protocols must be implemented across all service levels to guide diagnosis, treatment initiation, dosage titration, and side effect monitoring for epilepsy. The guideline highlights that inappropriate dosing or mismanagement in unstable conditions can either fail to control seizures or produce toxicity, especially when healthcare is delivered by rotating or inadequately trained staff. These protocols ensure that care is not dependent on individual expertise and is consistent across settings.

F. Referral systems must be maintained for epilepsy patients requiring diagnostics (e.g., for suspected secondary epilepsy due to trauma or infection), surgical evaluation, or management of refractory epilepsy. However, the article also requires contingency plans when referral is not possible—such as equipping primary-level teams with symptom control protocols and long-term medication regimens. In the absence of referral, patients are otherwise abandoned to repetitive seizures without any structured response.

***SUPPORT AND CONTINUUM OF CARE***

G. Follow-up systems must be implemented to maintain seizure control, adherence, and monitoring. The document stresses that epilepsy is a long-term condition and loss to follow-up during emergencies leads to increased seizure frequency, injuries, and stigmatization. Structured follow-up allows health teams to track medication effectiveness, renew prescriptions, and adjust therapy as needed.

H. Displaced individuals with epilepsy must receive extended medication supplies and portable documentation describing their diagnosis, prescribed AEMs, and seizure history. This is crucial during transit, as they are unlikely to find consistent care or pharmacy access along migration routes. Without such planning, displaced individuals risk immediate loss of seizure control in unfamiliar and potentially hazardous environments.

I. Patient and caregiver education is explicitly required to address epilepsy-related stigma and encourage consistent treatment. Education must include how to identify seizure triggers, how to respond to seizures safely, and when to seek medical help. The article underlines that in many humanitarian contexts, cultural beliefs lead to social exclusion or refusal of treatment, making community education a key part of epilepsy care continuity.

***TRAINING OF FORCES***

J. Medical personnel must be trained to recognize seizure types, administer emergency AEMs, and initiate long-term treatment. Training is especially critical for staff with no neurology background, who may confuse seizures with psychiatric or infectious presentations. The guideline notes that inappropriately managed seizures increase mortality risk and often result in preventable complications like aspiration, head trauma, or permanent neurological damage.

***CREATION OF STORAGE RESOURCES***

K. Storage of AEMs must be included in facility-level pharmaceutical systems, particularly in settings where sudden influxes of patients or environmental disruptions are likely. The article emphasizes the need for secure, climate-stable storage spaces to maintain the efficacy of long-term epilepsy medications, which may degrade under improper conditions.

L. Strategic pre-positioning of AEMs in vulnerable or remote areas is required to guarantee access in the event of logistical disruption, armed conflict, or destruction of central depots. Without this measure, populations with epilepsy are cut off from essential treatment, resulting in spikes in seizure-related mortality and injury.

***IMPROVEMENT OF INTER-SECTORAL COMMUNICATIONS***

M. The coordination of epilepsy care among governmental, humanitarian, and local health actors is essential to prevent fragmentation in drug supply, treatment regimens, and documentation. The article explains that uncoordinated systems cause confusion over which AEMs are available, conflicting protocols, or incomplete treatment records. This coordination ensures patients can transition between facilities and organizations without treatment interruption.

***PROVISION OF BUDGET***

N. Specific budget allocations are mandated to ensure continuous epilepsy care. These include procurement of medications, distribution logistics, training, and patient education materials. The article emphasizes that although AEMs are inexpensive compared to many other medicines, epilepsy remains underfunded due to low visibility. This results in preventable treatment gaps that increase morbidity and care costs downstream due to injuries, hospitalizations, or prolonged seizures.

***STRATEGIC OR OPERATIONAL PLANS***

O. Epilepsy must be incorporated into NCD strategies and operational response plans as a prioritized neurological condition. It is included due to the risk of medical emergencies when untreated and the high feasibility of management with low-cost interventions.

P. Operational planning must specify the full scope of epilepsy services: from emergency care and maintenance therapy to monitoring and referral. These standards must be flexible enough to adapt to instability while preserving safety and care continuity. Without operational prioritization, epilepsy services are among the first to collapse in humanitarian crises due to perceived complexity or low visibility.

**Disease:** Depression (in all crises)

**DRUG SUPPLY**
A. Continuous access to medicines for Depression, including emergency NCD medicines, is critical to preventing relapse and acute symptom deterioration in crises where access to care is interrupted. The document specifies that the ICRC must ensure the availability of continuous medication for highly prevalent NCDs with severe consequences if untreated, explicitly listing Depression alongside diabetes, cardiovascular diseases, COPD/asthma, and epilepsy. The rationale is that Depression, if left untreated, can lead to severe mental health crises, including psychotic episodes or suicide attempts, which require urgent pharmacological intervention. Ensuring uninterrupted drug supply is essential to avoid acute decompensation in these patients, particularly in unstable or displacement settings where mental health exacerbations are common due to the loss of family, home, and livelihood. No specific p-value/OR/CI reported.

**SERVICE PROVISION**
A. Depression care must be part of the continuum of care model, linking services from community level to primary, secondary, and tertiary care. This is necessary because Depression, as a chronic condition with fluctuating severity, requires ongoing follow-up and management to prevent acute exacerbations, such as suicidal crises or psychotic breakdowns.
B. Depression management is embedded within Mental Health and Psychosocial Support (MHPSS) programmes because humanitarian crises exacerbate psychological distress, with Depression being a key condition requiring targeted interventions. The ICRC's model integrates Depression into MHPSS to ensure that mental health needs are addressed alongside physical health conditions, recognising Depression as a critical, life-threatening component of NCD care.
C. Life-saving care must be provided for Depression patients in crisis situations where acute symptoms emerge (e.g., psychotic breaks, suicidal ideation). The document highlights that such care should include immediate stabilization, even in emergency settings, to prevent fatal outcomes or further deterioration.
D. Regular follow-up consultations, including monitoring of adherence, side effects, and complications, are mandatory for Depression patients, as interruptions in care increase the risk of severe outcomes, including suicide and worsening disability. Depression management requires frequent assessment of treatment efficacy and psychosocial functioning, as well as appropriate referrals if more specialized care is needed.
E. Referral criteria and pathways must be established for Depression patients when primary care is insufficient (e.g., psychosis, suicidal behaviour). This is essential as Depression can present with complex manifestations requiring secondary or tertiary care interventions, such as inpatient mental health treatment or advanced pharmacotherapy.
F. Palliative care must be available for Depression patients in terminal phases or for those unable to be transferred. This applies particularly in contexts where advanced mental health services are unavailable, ensuring that individuals with severe, treatment-resistant Depression can receive symptom management and supportive care to minimize suffering.
G. Depression care must be included in the ICRC’s response across all crisis phases (initial, acute, and continuing) because Depression symptoms can emerge or worsen at any stage due to the destabilizing effects of violence, displacement, and resource scarcity. This integration ensures that Depression care is not neglected during transitions between response phases. No specific p-value/OR/CI reported.

**SUPPORT**
A. Depression patients must receive patient education focusing on their therapies and self-management. The document specifies that self-management is vital in unstable humanitarian settings, where Depression patients may face disrupted access to care and need to manage their illness proactively, including medication adherence and recognition of warning signs.
B. Depression patients should receive patient-held clinical records, including booklets with pictograms and simplified explanations of treatments and follow-up schedules, to facilitate continuity of care across different facilities and providers. The rationale is that Depression, like other NCDs, often requires mobility across care settings, and without proper documentation, care is fragmented, increasing the risk of adverse outcomes.
C. Depression patient support groups should be created where they do not exist, as peer support is identified as a critical component of Depression care in crises. Such groups facilitate community engagement, reduce stigma, and promote adherence to treatment through shared experiences and coping strategies. No specific p-value/OR/CI reported.

**TRAINING OF FORCES**
A. Healthcare workers must receive training in Depression care as part of NCD training, ensuring they possess the necessary competencies to manage Depression in diverse and resource-limited settings. This is crucial because Depression symptoms can vary and may include acute crises requiring specific therapeutic interventions that general healthcare staff may not be familiar with.
B. A dedicated NCD team must be created, including at minimum an administrator, doctor, and nurse, to manage Depression cases effectively. This structure ensures that Depression, as part of the prioritized NCD package, receives systematic attention, preventing neglect in the broader health response.
C. Training of community healthcare providers and mediators in Depression care is mandatory, as they play a key role in identifying and managing Depression within communities, especially when access to specialized mental health professionals is limited. Their training focuses on early detection, basic therapeutic interventions, and referral pathways for Depression cases. No specific p-value/OR/CI reported.

**PREPAREDNESS PLANNING**
A. Depression care must be included in contingency plans to ensure the continuum of care in unstable settings. This measure acknowledges that Depression is a chronic and potentially life-threatening condition, and its management cannot be delayed or omitted during emergencies. Ensuring preparedness means that supplies, staff training, and referral pathways for Depression are considered proactively, allowing for a seamless response even when sudden crises arise. No specific p-value/OR/CI reported.

**BUDGET/RESOURCE ALLOCATION**
A. Depression care funding must be integrated into the emergency health program budget. The document specifies that funding must prioritize life-threatening conditions and interventions preventing complications from care interruptions, which explicitly includes Depression due to its potential for severe outcomes such as suicide or psychotic decompensation. This allocation is necessary to ensure availability of essential psychotropic medicines, support services, and staff capable of managing Depression in crises. No specific p-value/OR/CI reported.

**INTER-SECTORAL COLLABORATION**
A. Depression care must involve collaboration with other health services, including HIV/TB programs, community groups, women’s groups, elders, and patient associations, as these partnerships strengthen Depression management through shared resources, community engagement, and complementary expertise. This measure recognizes the multifactorial nature of Depression, which intersects with social, economic, and health factors in humanitarian settings.
B. Coordination with local health systems and national programmes is critical for Depression management, ensuring consistency with national standards and enhancing sustainability by integrating Depression care into existing systems. This approach facilitates referrals, access to specialized mental health services, and long-term continuity of care for Depression patients in crises.

**Disease:** Schizophrenia (in all crises)

**DRUG SUPPLY**
A. Continuous access to essential psychotropic medications must be ensured for Schizophrenia because the ICRC explicitly lists psychosis, which includes Schizophrenia, as a priority condition within its MHPSS programme. This inclusion acknowledges the life-threatening nature of untreated psychotic episodes, such as those seen in Schizophrenia, which can result in violent behaviour, severe agitation, or catatonia. Interruptions in antipsychotic medications—especially in crisis settings with damaged infrastructure, displacement, or disrupted supply chains—can lead to rapid decompensation and acute psychotic relapses. The document emphasizes that ensuring continuous access to these medicines is critical for preventing such deteriorations, which can severely impair patients’ ability to function, increase risk to themselves and others, and overwhelm already strained emergency health services. Emergency NCD medicines must also be stockpiled to buffer against access disruptions, as Schizophrenia requires uninterrupted pharmacological management. No specific p-value/OR/CI reported.

**SERVICE PROVISION**
A. Schizophrenia care must be integrated into the continuum of care model, which connects services from community-level care (First Aid, Prehospital Emergency Care) through Primary Health Care, Hospital Services, Health Care in Detention, Physical Rehabilitation, and Mental Health and Psychosocial Support (MHPSS). This design ensures that Schizophrenia, a condition requiring lifelong management, is supported across all levels of care, with proper referral and handover between services. Without this integration, Schizophrenia patients risk being lost between services, facing interrupted care, and experiencing symptom exacerbation, especially during transitions in crisis phases.
B. Schizophrenia management is part of the ICRC’s mandate to provide life-saving care for acute, potentially life-threatening symptoms, such as acute psychotic episodes, which can include aggression, delusions, hallucinations, and risk of suicide or harm to others. The document explicitly states that these conditions require immediate stabilization and, when necessary, referral to specialized care.
C. Regular follow-up for Schizophrenia patients, including clinical consultations, monitoring for adherence, side effects, and complications, is critical to ensure continuity of care in crises. The document specifies that follow-up care includes medicines, medical devices, laboratory tests when appropriate, and patient education, all of which are necessary for Schizophrenia management to prevent relapse, hospitalization, and functional decline.
D. Referral criteria and pathways must be established for Schizophrenia patients who cannot be managed at the primary care level. Schizophrenia often presents with complex psychiatric manifestations requiring secondary or tertiary care, such as inpatient psychiatric facilities for acute psychotic episodes, which cannot be provided at the primary care level.
E. Schizophrenia care must be maintained across all phases of crises—initial, acute, and continuing—because psychotic symptoms can emerge or worsen unpredictably in response to the destabilizing factors of crises, such as loss of home, family separation, violence, and scarcity of resources. The ICRC emphasizes that Schizophrenia care must not be deprioritized at any phase to avoid life-threatening exacerbations. No specific p-value/OR/CI reported.

**SUPPORT**
A. Patient education is critical for Schizophrenia management, focusing on understanding the nature of psychosis, recognizing early warning signs of relapse, maintaining medication adherence, and managing psychosocial stressors. The ICRC identifies education as a key element to empower patients to manage their conditions, particularly in crises where health services may be disrupted, and patients must rely on their knowledge to seek help or manage symptoms.
B. Patient-held clinical records, such as booklets with pictograms and simplified instructions, are required for Schizophrenia patients to maintain treatment continuity when moving between health services or facilities. Schizophrenia requires consistent pharmacological regimens, and without accurate records, patients may receive incorrect treatments or experience dangerous medication changes. The document notes that these records are essential to prevent care fragmentation in mobile populations.
C. Creation of patient support groups for Schizophrenia is necessary, as peer support can reduce stigma, promote adherence, and provide emotional and practical assistance to patients managing psychotic illnesses in crisis settings. Support groups also facilitate engagement with the health system and provide a venue for patient education, enhancing the continuum of care for Schizophrenia. No specific p-value/OR/CI reported.

**TRAINING OF FORCES**
A. Healthcare workers must receive training in the management of Schizophrenia as part of core NCD and MHPSS training. Schizophrenia management requires specialized knowledge of psychotropic medications, symptom monitoring, crisis de-escalation techniques, and referral pathways, all of which are essential competencies in humanitarian crises where specialist psychiatric staff may be unavailable.
B. Establishment of a dedicated NCD team—including an administrator, doctor, and nurse—is required for Schizophrenia management, ensuring that care is coordinated, systematic, and integrated within the health response. This team is necessary to provide structured oversight and case management for Schizophrenia patients, ensuring they receive appropriate and timely interventions.
C. Community health workers and mediators must also be trained in Schizophrenia care, as they play a vital role in detecting early signs of psychosis in the community, facilitating access to care, and supporting adherence. The document emphasizes that community engagement is critical for identifying “invisible” patients who may not present themselves to health facilities, especially in settings where mental illness is stigmatized. No specific p-value/OR/CI reported.

**PREPAREDNESS PLANNING**
A. Schizophrenia care must be included in contingency plans to ensure that patients continue to receive essential medications and psychosocial support during crises. The ICRC highlights that care for chronic conditions like Schizophrenia cannot be paused during emergencies, as abrupt discontinuation of antipsychotic medications can lead to psychotic relapse, hospitalization, and increased mortality risk. Preparedness includes ensuring stockpiles of medicines, staff training, and referral pathways for psychosis management. No specific p-value/OR/CI reported.

**BUDGET/RESOURCE ALLOCATION**
A. Funding for Schizophrenia care must be included in emergency health programme budgets, recognizing the high risk of life-threatening exacerbations without continuous access to medications and care. The document prioritizes funding for life-saving interventions for conditions such as psychosis, which includes Schizophrenia, due to the potential for severe mental health crises, including suicide, violence, and prolonged disability, if care is interrupted. No specific p-value/OR/CI reported.

**INTER-SECTORAL COLLABORATION**
A. Collaboration with other health services, such as community groups, women’s groups, elders, and patient associations, is necessary for Schizophrenia care, as these networks help identify patients, provide informal support systems, reduce stigma, and facilitate engagement with formal healthcare services. Schizophrenia is often hidden in communities, and such partnerships are essential for reaching isolated or marginalized patients.
B. Coordination with national mental health systems and existing psychiatric services ensures that Schizophrenia care aligns with local protocols, enabling access to specialized care, facilitating referrals, and promoting sustainability by integrating crisis interventions into long-term care structures.

**Disease:** Anxiety (in all crises)

**SERVICE PROVISION**
A. Anxiety care is specifically listed as a priority condition in the ICRC’s mental health and psychosocial support (MHPSS) programme for humanitarian crises. The document identifies “psychosis, depression, and anxiety” as core psychiatric diseases that require targeted interventions, recognizing that these conditions are common in crisis-affected populations. The inclusion of Anxiety reflects its high prevalence and the increased risk of symptom exacerbation during crises, where displacement, violence, and loss of resources cause acute psychological distress. Management of Anxiety must be part of the general NCD response, incorporated into the continuum of care model that integrates First Aid, Prehospital Emergency Care, Primary Health Care, Hospital Services, Health Care in Detention, Physical Rehabilitation, and MHPSS services. The ICRC guidelines state that acute mental illness such as Anxiety, triggered by humanitarian crises, must be managed using emergency protocols, including the basic psychiatric care package. This package outlines essential measures for the rapid identification and stabilization of acute Anxiety symptoms, such as panic attacks, severe agitation, or dissociative states, and establishes pathways for follow-up and referral as needed.
B. Anxiety care must include therapeutic patient education to empower patients in understanding their condition, recognizing early signs of relapse, and knowing how to access care in unstable settings. The document highlights education as a critical element of MHPSS interventions, particularly for Anxiety, as patients may face unpredictable access to formal care in crises. Education aims to promote self-management by teaching patients techniques for anxiety reduction, such as relaxation, breathing strategies, and recognizing triggers, while also reinforcing the importance of continued treatment adherence in cases where pharmacological or psychosocial interventions are prescribed.
C. Community engagement is essential for Anxiety care, as the document emphasizes the use of health committees, patient associations, and self-help groups to identify and support individuals with Anxiety who may not proactively seek care due to stigma, fear, or lack of awareness. In crisis contexts, patients with Anxiety often become “invisible” because their symptoms may not be immediately life-threatening, yet their suffering and risk of deterioration are significant. Community structures play a vital role in making these cases visible, ensuring they are referred to appropriate services, and fostering a supportive environment that reduces isolation and promotes mental health awareness. No specific p-value/OR/CI reported.

**SUPPORT**
A. Anxiety patients must receive targeted patient education, which is a critical component of MHPSS. The ICRC underscores that educating patients on their condition, its symptoms, and available treatment options is essential for Anxiety management, particularly in unstable settings where patients may not have continuous access to mental health professionals. Education helps Anxiety patients understand how their symptoms may fluctuate with crisis-related stressors, such as displacement, violence, and food insecurity, and provides them with coping strategies and action plans for seeking care when symptoms worsen.
B. Community engagement structures, such as health committees, self-help groups, and patient associations, must be leveraged to support Anxiety patients. These groups play a key role in identifying individuals with Anxiety who may remain hidden due to fear of stigma or lack of mental health literacy. The document explains that Anxiety is often not recognized by the affected person or their family, which necessitates proactive community involvement to ensure that Anxiety cases are referred for care and included in the broader health response. Community engagement also facilitates shared learning, normalization of mental health discussions, and peer support for individuals living with Anxiety. No specific p-value/OR/CI reported.

**TRAINING OF FORCES**
A. Training of healthcare workers must include specific components on the management of Anxiety as part of core NCD and MHPSS training. The document mandates that healthcare staff working in crises must be trained to recognize, assess, and manage mental health conditions such as Anxiety, which often manifest in response to acute stressors such as violence, displacement, and resource loss. Training equips staff to identify Anxiety symptoms early, provide basic therapeutic interventions, and establish referral pathways to specialized care when needed. This is critical because Anxiety can present with somatic complaints (e.g., chest pain, palpitations, breathlessness), which can be misdiagnosed as physical conditions without proper mental health training. No specific p-value/OR/CI reported.

**PREPAREDNESS PLANNING**
A. The document states that Anxiety care must be included in preparedness and contingency plans for crisis situations. This integration is essential because Anxiety often emerges or worsens in the context of acute emergencies, and without preparedness, there will be no structures or protocols to provide necessary care. Preparedness involves ensuring that staff are trained, that referral pathways for Anxiety management are established, and that MHPSS services are embedded into all stages of the crisis response. This ensures that Anxiety patients are not neglected during periods of sudden displacement, violence, or health system disruption. No specific p-value/OR/CI reported.

**BUDGET/RESOURCE ALLOCATION**
A. Funding for Anxiety care must be explicitly allocated in emergency health programme budgets. The document emphasizes that, when resources are limited, prioritization must focus on life-saving interventions, which include management of acute mental health conditions like Anxiety. Budgetary provisions must ensure that Anxiety management is supported by trained staff, community engagement activities, and, where necessary, access to therapeutic interventions such as brief psychological support or basic pharmacotherapy. Without dedicated funding, Anxiety services risk being deprioritized in crises, despite the significant burden of Anxiety in humanitarian populations. No specific p-value/OR/CI reported.

**INTER-SECTORAL COLLABORATION**
A. Collaboration with community groups, women’s associations, elders, and patient associations is required for effective Anxiety care. These networks are instrumental in identifying individuals with Anxiety who may be isolated or stigmatized, ensuring they receive appropriate care. The document explains that mental health conditions like Anxiety are often hidden in crisis-affected populations, requiring inter-sectoral collaboration to reach and support these patients. This collaboration also allows for the sharing of culturally relevant coping strategies and community-specific interventions that can be integrated into Anxiety management.
B. Coordination with national mental health systems and psychiatric services is necessary to ensure that Anxiety care aligns with local standards, facilitates referrals, and promotes continuity of care. This coordination allows the ICRC to integrate its services into existing systems, supporting long-term sustainability and avoiding fragmentation of care for Anxiety patients.

**22. Article Title:** Addressing noncommunicable diseases in emergencies: A regional framework for action

**Author, Publication year:** WHO, 2024

The article discusses cyclone, flood, and storm-related disasters as a combined category throughout, and does not stratify specific strategies or measures by disaster type. But the article does stratify the impact and relevant considerations by disease type.

**List of the NCD diseases considered in this article:** Hypertension, Stroke, Myocardial Infarction or Angina, Cancer, Kidney Disease, Diabetes Mellitus, Hyperlipidemia

**List all of the crises and disasters considered in this article**
(Only non–inside health system crises like natural disasters, conflict, displacement)
• Humanitarian emergencies
• Natural disasters (cyclones, floods, storms, hurricanes, earthquakes, tsunamis, bushfires, tornadoes)
• Conflict situations
• Displacement
• Refugee crises
• Migration crises

**Main focus (included in analysis):** Hurricanes, Earthquakes, Tsunamis, Flooding, Bushfires, Tornadoes

**Disease:** All NCDs (Held-in-Common Measures)
**Crisis: All Crises**
***STRATEGIC AND OPERATIONAL PLANS***
A. The article mandates the development of national operational guidance and standard operating procedures (SOPs) for all NCDs. These SOPs must include instructions on how to manage both life-threatening and chronic conditions across disrupted settings, covering aspects such as triage, continuity of medication, referral protocols, and service adjustments during emergency phases. Without these SOPs, health providers face uncertainty in decision-making, which increases the risk of avoidable mortality and complication among NCD patients during crises.
B. Emergency preparedness must adopt an all-hazards framework with NCD integration across every stage—from prevention and risk reduction to recovery. The article explains that this ensures chronic diseases are not sidelined in favor of acute or infectious conditions. It standardizes procedures to ensure that minimum NCD service packages are embedded within every national and subnational disaster preparedness framework.

***SERVICE PROVISION***
C. The article requires explicit integration of NCD care into the national health sector emergency response plan. This guarantees that NCD management—including outpatient follow-up, medication refills, acute complication handling, and referrals—is maintained even during total system disruption. If not included, NCD services are often deprioritized, leading to worsening disease progression and emergency department overload.
D. Alternative delivery systems such as temporary clinics, mobile health units, and telemedicine platforms must be deployed to maintain access to care. These methods address accessibility barriers due to infrastructure damage, conflict, or geographic displacement. The article mandates that such mechanisms must be part of national-level NCD response architecture to guarantee service continuation.
E. Structured referral systems must remain active during emergencies. The article stresses that NCD care frequently involves multi-tiered services—from community to tertiary level. In the absence of clear referral pathways, patients requiring diagnostic evaluation, specialist care, or rehabilitation will face treatment abandonment and long-term functional decline.

***DRUG SUPPLY***
F. The NCD emergency kit is mandated to include essential medications for cardiovascular disease, diabetes, respiratory conditions, and other chronic illnesses. This ensures the immediate availability of life-sustaining medications during crisis response. Without these kits, there is a direct risk of acute decompensation among patients dependent on continuous pharmacological treatment.
G. A national mechanism for procurement, secure distribution, and facility-level stock monitoring must be maintained for NCD medicines and supplies. The article states this measure is essential for preventing stock-outs, ensuring equitable access across population groups, and maintaining pharmacologic continuity for all chronic conditions.

***TRAINING OF FORCES***
H. Health care providers must be trained to deliver NCD care under emergency conditions. The article requires training programs to include modules on managing chronic disease exacerbations, modifying regimens based on available drugs, and maintaining clinical continuity during infrastructure failures. Without this knowledge, frontline workers risk under-treating or mismanaging patients with NCDs.
I. Community health workers must be rapidly trained and deployed to support people with NCDs. This includes education on symptom recognition, referral mechanisms, and psychosocial support. Their role becomes critical in reaching patients who are displaced, isolated, or unable to access institutional services due to insecurity or natural disasters.

***RESOURCE STORAGE AND STOCKPILING***
J. Preparedness systems must include designated storage facilities and cold-chain mechanisms to maintain adequate reserves of NCD-related drugs and supplies. The article specifies that all countries must maintain stockpiles that are ready for immediate deployment, particularly in conflict-prone or disaster-vulnerable zones, to mitigate disruptions in treatment continuity.

***FINANCIAL SUPPORT AND BUDGET ALLOCATION***
K. Countries must allocate flexible and sufficient emergency funds for the provision of essential NCD services. This includes maintaining budget lines for medications, diagnostics, staff mobilization, and temporary service facilities. The article indicates that excluding NCDs from emergency budgets has historically resulted in major care disruptions and catastrophic health outcomes.
L. Financial protection mechanisms must be established to shield individuals with NCDs from out-of-pocket expenditure during emergencies. The article states that both citizens and displaced populations must be included under these protection schemes to ensure equity and sustained access to care.

***COMMUNITY ENGAGEMENT AND SUPPORT***
M. NCD-affected individuals must be formally involved in planning and evaluation processes during emergency preparedness and response. The article defines this as "meaningful engagement," which ensures that care models reflect the lived realities and service priorities of people with chronic conditions. This enhances service relevance and utilization during crises.
N. Dialogue and feedback systems must be established to gather real-time input from people living with NCDs. The article emphasizes the use of community platforms, hotlines, and digital surveys to track service gaps, access barriers, and medication shortages, enabling a responsive and accountable NCD crisis response.

***SURVEILLANCE AND INFORMATION SYSTEMS***
O. NCD data must be included in emergency assessments and surveillance dashboards. The article directs all health authorities to track disease-specific service availability, facility-level stock data, and patient follow-up rates. This allows resource allocation and system adaptation to be based on actual service coverage and patient needs.
P. The article mandates the integration of NCD indicators in national Health Emergency Operation Centres (HEOCs) to enable dynamic monitoring and reporting on service continuity and population-level burden during emergencies.

***DIGITAL HEALTH AND TECHNOLOGY***
Q. The use of digital health solutions is mandatory for sustaining NCD care. This includes telemedicine for consultations, mobile health platforms for follow-up and adherence tracking, and electronic medical records to maintain continuity in displaced populations. These tools become critical substitutes for in-person services when access is restricted.

***LEADERSHIP AND GOVERNANCE***
R. Each emergency response team must include an appointed NCD focal point responsible for coordinating all aspects of chronic disease care. This individual ensures prioritization of NCDs in funding, training, logistics, and reporting systems.
S. A national multisectoral working group must manage the NCD component of emergency responses. The article requires representation from sectors such as health, finance, water, logistics, and civil society to guarantee cross-functional support and avoid care fragmentation for patients with chronic diseases.

**Disease:** Hypertension
**Crisis: All Crises**

***DRUG SUPPLY***
A. The article mandates the inclusion of antihypertensive medications in the NCD emergency kit. This directive specifically names hypertension as a priority condition requiring continuous pharmacological management during emergencies. The rationale is that sudden interruption in antihypertensive therapy significantly increases the risk of acute complications, such as hypertensive encephalopathy, myocardial infarction, and cerebrovascular accidents. By pre-positioning essential drugs like beta-blockers, ACE inhibitors, calcium channel blockers, and diuretics, the NCD emergency kit ensures that patients already stabilized on chronic treatment can avoid life-threatening elevations in blood pressure.
B. The article emphasizes that the inclusion of antihypertensive agents in emergency kits is critical for the acute management of hypertensive emergencies in displaced or isolated populations. These medications are listed as part of a life-sustaining pharmacologic reserve and are prioritized because uncontrolled blood pressure during crises can lead to irreversible end-organ damage.

***TRAINING OF FORCES***
C. The article states that health care providers must be trained to identify and manage hypertensive crises in emergency conditions. This includes recognizing red flag symptoms such as sudden-onset headache, visual disturbances, or seizures indicative of hypertensive urgency or emergency. Providers must also be trained in dose adjustments when routine medications are unavailable and in the use of alternative regimens present in the emergency kits. This training prevents substandard care in facilities where diagnostic resources may be limited and where rapid clinical judgment determines outcomes.
D. The article requires emergency training to cover hypertension-specific risk stratification and triage. This includes distinguishing between patients needing immediate intravenous therapy and those manageable through oral regimens, which is critical in resource-constrained or triage-heavy environments.

***RESOURCE STORAGE AND STOCKPILING***
E. The article highlights that national stockpiling mechanisms must include adequate quantities of antihypertensive medications. This requirement is disease-specific due to the high prevalence of hypertension and its role as a precursor to crisis-exacerbated morbidity. Storage protocols must also account for drug stability, including the need for climate-controlled environments to maintain medication efficacy, especially in disaster-prone regions where heat and humidity can compromise pharmaceutical integrity.
F. The document emphasizes that failure to include antihypertensives in stockpiles leads to immediate risk of uncontrolled blood pressure among previously stabilized patients, especially those who are elderly, displaced, or dependent on public health systems. Stockpiles also reduce dependency on disrupted international supply chains and facilitate rapid deployment to mobile clinics and field hospitals.

***FINANCIAL SUPPORT AND BUDGET ALLOCATION***
G. The article mandates that emergency financial plans must specifically allocate budget for the procurement and distribution of antihypertensive medications. The justification given is that in the absence of earmarked funds, antihypertensive therapies are frequently omitted in favor of more acute or infectious disease treatments, despite the high risk of stroke, cardiac decompensation, or renal failure in hypertensive patients.
H. The article explains that financial protection systems must explicitly cover hypertension medications to prevent treatment abandonment. In crises, patients often lose income or health insurance coverage. Without financial protections, the economic barrier leads to skipped medication, uncontrolled blood pressure, and consequent emergency hospitalizations, which are both costly and avoidable.

***COMMUNITY ENGAGEMENT AND SUPPORT***
I. The article states that community-based feedback platforms must collect data specifically from individuals with hypertension regarding access challenges, medication availability, and treatment continuity. These platforms ensure the visibility of hypertensive patients' needs in decision-making processes during emergencies.
J. People living with hypertension must be actively involved in planning groups and community advisory boards during emergency preparedness and response. Their inclusion ensures that field-level challenges—such as drug substitution intolerance, difficulty accessing monitoring equipment, and gaps in follow-up care—are communicated to authorities and reflected in logistical planning.

**Disease:** Stroke or Myocardial Infarction or Angina
**Crisis: All Crises**

***DRUG SUPPLY***
A. The article mandates that the **NCD emergency kit** include **medications essential for acute cardiovascular conditions**, with explicit reference to stroke and myocardial infarction. These include antiplatelet agents, anticoagulants, antihypertensives, and statins. Their inclusion is prioritized due to the time-sensitive nature of cardiovascular emergencies. Without immediate pharmacological intervention, patients experiencing myocardial infarction or ischemic stroke are at elevated risk of irreversible organ damage or death. These medications form the foundation of initial stabilization and are vital when infrastructure collapse limits access to tertiary services.
B. The article also underscores the importance of maintaining availability of **emergency cardiovascular medications** in stockpiles as part of a broader risk mitigation strategy. Since stroke and acute coronary syndromes often require urgent pharmacological action—e.g., administration of aspirin or other thrombolytics—stock-outs directly translate into preventable mortality during emergencies.

***TRAINING OF FORCES***
C. The article requires **training programs for health care workers** to specifically include **recognition and initial management of myocardial infarction, angina, and stroke**. This includes identification of chest pain, dyspnea, facial asymmetry, unilateral weakness, and other hallmark signs. Training must also cover administration protocols for emergency medications such as aspirin and rapid referral for advanced care when feasible. Due to the rapid progression and acute nature of these conditions, training enables timely triage and lifesaving intervention in low-resource and high-pressure emergency environments.
D. The training also addresses **modification of clinical protocols** when diagnostic infrastructure is compromised. For example, providers must be capable of initiating empiric treatment for suspected stroke or acute coronary syndrome based on symptomatology alone, in the absence of ECGs or imaging. This ensures treatment is not delayed awaiting unavailable diagnostics.

***RESOURCE STORAGE AND STOCKPILING***
E. The article highlights that **emergency stockpiles must contain medications for acute cardiovascular events**, explicitly naming stroke and myocardial infarction among the priority targets. Given that these conditions often require medications within a narrow therapeutic window, stockpiles must be ready for **immediate deployment** in crisis-affected regions. Lack of timely access would result in a higher incidence of avoidable disability or death. Cold-chain maintenance, secure distribution, and periodic restocking are mandated to ensure drug viability and uninterrupted availability.

***FINANCIAL SUPPORT AND BUDGET ALLOCATION***
F. The article mandates that **emergency budgets must allocate dedicated funds for the treatment of acute cardiovascular conditions**, including medications and frontline stabilization services for stroke and myocardial infarction. Failure to do so results in systemic neglect of non-infectious acute presentations in emergency settings. Given the high cost of some cardiovascular medications and diagnostics, the article stresses that these costs must not be transferred to patients, especially in low-income or displaced populations.
G. Financial protection mechanisms must explicitly **cover treatment costs related to myocardial infarction and stroke**, as these are among the most financially catastrophic health events during emergencies. Without structured funding, patients face medication interruption, hospital care denial, or treatment abandonment.

***COMMUNITY ENGAGEMENT AND SUPPORT***
H. The article states that **patients with a history of stroke or ischemic heart disease must be engaged in emergency planning and feedback systems** to identify barriers to care. These include medication unavailability, transport inaccessibility, and insufficient knowledge among first responders. This engagement is critical because post-event patients have complex care needs and are often at higher risk of deterioration under crisis conditions.
I. Community platforms must be used to gather **real-time data from individuals at risk of or previously affected by stroke or myocardial infarction**, enabling emergency planners to anticipate demand surges and design referral networks that reflect actual patient flows.

**Disease:** Cancer
**Crisis: All Crises**

***DRUG SUPPLY***
A. The article mandates that essential cancer medications—including chemotherapy agents, hormone therapy, and supportive care drugs—must be incorporated into the national emergency medical supply kits. Cancer is classified in the article as a condition requiring uninterrupted pharmacologic management. Chemotherapy schedules are time-sensitive; missing doses or treatment cycles results in disease progression and the failure of curative intent. For patients undergoing active treatment, sudden interruption leads to therapeutic resistance and metastatic spread. Emergency kits are designed to prevent these interruptions and allow for treatment continuity during displacements or system breakdown.
B. The inclusion of cancer drugs in emergency inventories is justified by the disease’s rapid clinical deterioration if untreated. The article emphasizes that agents for palliative symptom relief (e.g., opioids for cancer pain, antiemetics, and corticosteroids) are also essential, as delays in palliative treatment increase morbidity and suffering in end-stage patients.

***SERVICE PROVISION***
C. The article requires that cancer care services—including chemotherapy, symptom relief, and specialist consultation—be maintained as essential services within emergency response structures. The justification is that cancer patients often require scheduled treatment cycles over weeks or months; missing one cycle can result in permanent treatment disruption. The article notes that cancer is not only chronic but often acutely life-threatening, and therefore its management must not be deprioritized.
D. In regions where patients are displaced or hospitals are non-functional, the article mandates the establishment of mobile clinics or telemedicine platforms that can facilitate oncology consultations, drug administration, or symptom management. This is to prevent loss to follow-up and manage side effects of previous treatments already initiated before the crisis began.

***TRAINING OF FORCES***
E. The article requires that medical personnel receive specific training in recognizing and managing acute cancer-related complications during emergencies. These include tumor lysis syndrome, febrile neutropenia, airway obstruction from head and neck tumors, and severe pain from metastases. Trained providers must know how to initiate immediate care or stabilize patients while arranging referral. In emergency zones where oncologists are not available, general practitioners must have the competence to manage these conditions.
F. The article also mandates training in the adaptation of cancer treatment protocols when the full therapeutic regimen is unavailable. This includes dose modifications or temporary supportive care when chemotherapy agents are inaccessible, to reduce immediate risk and maintain minimal disease control.

***RESOURCE STORAGE AND STOCKPILING***
G. Cancer medications, including cytotoxic agents, biologics, and adjunctive drugs, must be stored in climate-controlled stockpiles. The article stresses that many of these medications have temperature sensitivity and loss of efficacy if exposed to heat or humidity. Emergency preparedness must therefore include dedicated oncology storage areas with reliable electricity for refrigeration and secure containment due to the toxic nature of many cancer drugs.
H. Stockpiles must be updated frequently to ensure that expiration dates of chemotherapy and biologic agents are monitored. The article indicates that expired medications pose safety risks and therapeutic ineffectiveness, which is particularly dangerous in oncology where precise dosing and drug integrity are critical.

***FINANCIAL SUPPORT AND BUDGET ALLOCATION***
I. The article requires that cancer care receive explicit budget lines within emergency financial planning frameworks. This includes costs for chemotherapy procurement, staffing of specialist oncology providers, infrastructure for drug administration, and maintenance of diagnostic services. The article states that, historically, cancer care has been excluded from emergency budgets due to its high cost, resulting in preventable deaths and long-term disability.
J. Financial protection mechanisms must be extended to cancer patients during crises, as the article identifies cancer as one of the most financially catastrophic conditions. Many families are forced into poverty due to out-of-pocket payments for chemotherapy or surgery. The article mandates the establishment of subsidized or publicly financed cancer treatment schemes during emergencies, especially for displaced or uninsured populations.

***COMMUNITY ENGAGEMENT AND SUPPORT***
K. The article mandates that individuals undergoing cancer treatment or in post-treatment surveillance be included in emergency planning consultations. Their firsthand experience with treatment barriers, fear of recurrence, and medication access challenges helps health systems tailor cancer-related crisis responses.
L. Emergency response must establish community-level feedback mechanisms that collect data from cancer patients on treatment continuity, side effect management, and drug availability. These feedback systems allow for real-time modifications to service delivery and highlight gaps in coverage.

**Disease:** Kidney Disease
**Crisis: All Crises**

***SERVICE PROVISION***
A. The article mandates that kidney disease, particularly end-stage renal disease (ESRD), must be addressed through the **continuation of life-saving interventions such as dialysis** during emergencies. It explicitly states that patients undergoing maintenance dialysis are at extreme risk during crises due to their dependency on frequent, scheduled treatment. Missed dialysis sessions can lead to rapid fluid overload, electrolyte imbalance, and death. As a result, dialysis access must be protected through contingency planning that ensures transportation, facility functionality, and prioritized triage of ESRD patients.
B. The article specifies that **national emergency response plans must ensure uninterrupted access to dialysis centers or mobile dialysis units** where feasible. If health infrastructure is compromised, emergency preparedness must include mechanisms for temporary referral to functioning centers in surrounding areas or field-based services for stabilization. The failure to plan for these contingencies results in fatal treatment interruptions for patients requiring chronic renal replacement therapy.

***DRUG SUPPLY***
C. The article includes **renal-supportive medications**—such as phosphate binders, erythropoiesis-stimulating agents (ESAs), and antihypertensive agents used in chronic kidney disease management—as **essential components** in emergency NCD drug inventories. These medications are required to control complications like anemia, mineral imbalance, and blood pressure dysregulation. Their absence during crises increases hospitalization rates and long-term morbidity among patients with CKD or ESRD.
D. Emergency kits and pharmaceutical procurement plans must **account for the recurring need for renal-specific drugs**, not just one-time treatments. These medications are life-sustaining and not interchangeable with non-specific drugs. Delayed or interrupted access leads to deterioration in patients’ metabolic stability and contributes to accelerated progression to dialysis dependency.

***RESOURCE STORAGE AND STOCKPILING***
E. The article mandates that **dialysis supplies and renal medications must be included in national emergency stockpiles**. This includes dialyzers, tubing, fluids, catheters, and anticoagulants like heparin. Stockpiles must be appropriately stored under regulated conditions to ensure safety and usability during deployment. The article emphasizes that the logistical challenges of supplying dialysis consumables during emergencies must be addressed in advance due to the bulk and sensitivity of such supplies.

***FINANCIAL SUPPORT AND BUDGET ALLOCATION***
F. The article stresses the **high cost of dialysis and CKD treatment**, stating that emergency budgets must allocate dedicated lines for the **supply of dialysis consumables, machine maintenance, and specialist staffing**. Without specific budget planning, kidney disease care is often deprioritized in emergencies in favor of short-term curative interventions. The omission of renal services from financial planning results in catastrophic health outcomes for this population.
G. **Financial protection mechanisms must include chronic kidney disease patients** in emergencies, especially those requiring routine dialysis. The article highlights the economic burden of dialysis, often unaffordable without government or humanitarian funding. Without such protections, patients forgo treatment or incur devastating debt, leading to high mortality and impoverishment.

***COMMUNITY ENGAGEMENT AND SUPPORT***
H. The article requires **formal engagement of individuals living with kidney disease** in emergency planning structures. This inclusion is critical to understanding unique access barriers such as distance from dialysis centers, inability to pay for transportation, and the need for family support during treatments. The inclusion of renal patients in advisory processes allows the health system to prioritize facility readiness, transport coordination, and patient tracking.
I. The article also mandates the establishment of **feedback systems to collect real-time data from CKD and dialysis patients**, particularly concerning missed treatments, symptom exacerbation, and medication shortages. These systems are necessary to alert health authorities for immediate intervention in cases of service interruption.

**Disease:** Liver Disease
**Crisis: All Crises**

***DRUG SUPPLY***
A. The article specifies that the **NCD emergency kit must include essential medications for liver disease**, with particular reference to **treatment for hepatic encephalopathy, diuretics for ascites management, and antiviral medications for viral hepatitis-related liver disease**. These drugs are considered critical because liver disease can rapidly deteriorate in the absence of appropriate pharmacological intervention, particularly during crisis conditions. Acute complications like encephalopathy, gastrointestinal bleeding, and fluid overload require immediate treatment. Without inclusion of these medications in emergency kits, the risk of mortality and irreversible organ failure is significantly heightened.
B. The document also emphasizes the need for **medications that support liver function or manage chronic liver disease complications** to be consistently stocked and distributed during crises. For instance, drugs like lactulose, rifaximin, and spironolactone must be prioritized due to their role in preventing recurrent hospitalizations and controlling advanced disease symptoms.

***SERVICE PROVISION***
C. The article mandates that **specialized services for liver disease, particularly in cases of decompensated cirrhosis or liver failure, must remain operational during emergencies**. Liver disease is explicitly listed among NCDs requiring **specialist intervention**, meaning that services such as ultrasound monitoring, paracentesis, and management of complications like variceal bleeding must be supported even when hospital systems are under strain. The failure to maintain such services leads to high mortality, especially in patients with portal hypertension and hepatic decompensation.
D. The article further calls for **telemedicine consultations for liver patients** during emergencies in cases where mobility or access to tertiary centers is restricted. These platforms enable physicians to guide local providers in managing complex liver cases, adjust treatment plans, and detect signs of acute decompensation requiring urgent referral.

***RESOURCE STORAGE AND STOCKPILING***
E. The article includes **liver-related medications and diagnostic supplies** in the category of essential items requiring protected storage in national stockpiles. Many of these medications, such as certain antivirals or injectables, require cold-chain logistics or secure packaging to maintain efficacy. The article states that disruptions in storage conditions lead to ineffective treatment and preventable escalation of liver-related complications.

***FINANCIAL SUPPORT AND BUDGET ALLOCATION***
F. The article mandates that **emergency budgets must account for the high-cost treatments and diagnostics associated with liver disease**, particularly in patients with end-stage liver failure who require frequent monitoring and medication. If not allocated explicitly, these services are likely to be deprioritized in favor of lower-cost acute interventions, leading to preventable mortality in liver patients.
G. The article further requires that **financial protection mechanisms must include patients with liver disease**, especially those undergoing long-term treatment for hepatitis B or C. These therapies are expensive and lifelong, and in the absence of financial support, patients discontinue care during emergencies, risking liver failure and transmission to others.

***COMMUNITY ENGAGEMENT AND SUPPORT***
H. The article requires that **individuals with liver disease be engaged in emergency planning activities**, especially in areas with a high burden of viral hepatitis or cirrhosis. Their participation allows health systems to understand challenges such as lack of drug availability, food insecurity affecting liver metabolism, and stigma around liver disease (e.g., assumptions of alcohol use or contagion).
I. Feedback systems must be designed to **monitor access to liver medications and complication management** (e.g., for ascites or encephalopathy) during crises. Real-time data from these systems allows public health agencies to detect gaps and respond proactively before patient deterioration occurs.

**Disease:** Diabetes Mellitus
**Crisis: All Crises**

***DRUG SUPPLY***
A. The article mandates that insulin and oral hypoglycemic agents must be specifically included in the NCD emergency kit. This directive is essential due to the unique, life-threatening nature of diabetes-related decompensations, such as diabetic ketoacidosis and hyperosmolar hyperglycemic state, which can develop rapidly in the absence of insulin. The article does not generalize this requirement across all NCDs but rather names diabetes explicitly, indicating that without insulin, diabetic individuals are at high risk of mortality within days. This measure ensures pharmacologic continuity, recognizing that insulin is temperature-sensitive and its availability is often compromised in crisis settings.

B. The article identifies that the inclusion of diabetes medications in the emergency kit is critical because of the time-sensitive and continuous nature of treatment. Patients with diabetes cannot tolerate missed doses without experiencing glycemic instability, and the consequences of medication discontinuation can rapidly become life-threatening. Ensuring insulin and oral agents are in the emergency kits guarantees baseline continuity of care for diabetic patients in disrupted environments.

***SERVICE PROVISION***
C. The article demands the maintenance of uninterrupted access to insulin and diabetes-specific care services. It distinguishes this from general NCD care by highlighting the need for cold-chain systems for insulin preservation, which is not a shared requirement for most other NCD medications. The text underscores that insulin-dependent patients face immediate health deterioration without preserved medication efficacy, necessitating refrigeration systems that function even in the absence of stable electricity.

D. Beyond drug access, the article calls for operational protocols tailored to diabetes. It points out that management of diabetes during emergencies requires the ability to monitor blood glucose levels and administer rapid-acting interventions for both hypo- and hyperglycemia. The service provision plan must include provisions for diagnostic capacity (e.g., glucometers and strips) and education on symptom recognition for metabolic emergencies, uniquely relevant to diabetes.

***SURVEILLANCE AND INFORMATION SYSTEMS***
E. Diabetes is explicitly cited in the article as one of the conditions for which patient lists must be maintained and updated during emergencies. The article stresses that registries must include patients with advanced complications (e.g., diabetic foot, proliferative retinopathy, nephropathy), and that this data is essential for targeting services, ensuring follow-up, and avoiding deterioration in displaced or isolated populations. The presence of such lists enables health systems to plan for mobile outreach, medication allocation, and rapid referral based on severity and complication risk.

F. The article emphasizes that diabetic individuals, especially those on insulin therapy, are at elevated risk for treatment discontinuation due to displacement and service fragmentation. Therefore, integrating diabetes-specific metrics in surveillance dashboards enables real-time tracking of treatment continuity, which is essential for prioritizing resources such as mobile units, medication deliveries, and targeted patient outreach during disaster phases.

**Disease:** Hyperlipidemia
**Crisis: All Crises**

***DRUG SUPPLY***
A. The article explicitly states that essential medications for hyperlipidemia must be included in the NCD emergency kit. This includes lipid-lowering therapies such as statins, which are required for the primary and secondary prevention of cardiovascular events in patients with elevated lipid levels. These medications are uniquely listed by disease name, indicating their required inclusion separate from general cardiovascular drug mentions. Their inclusion ensures continuity of therapy for hyperlipidemia patients, whose condition—though often asymptomatic—contributes significantly to acute coronary and cerebrovascular events if unmanaged during crises.

B. The article highlights that the availability of hyperlipidemia medications in emergency kits supports the prevention of complications such as myocardial infarction and stroke in at-risk populations. The article distinguishes this pharmacological requirement as disease-specific rather than grouping it under generic chronic disease drug continuity, underscoring its role in mitigating crisis-exacerbated cardiovascular risk factors.

**23.Article Title:** Cancer Care After Türkiye-Syrian Earthquakes: A Qualitative Study

**Author, Publication year:**
Gürkan Sert, 2024

In the field of cancer, many cancer patient associations (CPAs) have been established in Türkiye to support individuals living with cancer (ILCs) throughout the diagnosis and treatment processes. The presence of CPAs for ILCs became more significant after the major earthquakes in southeastern Türkiye in February 2023. The aim of this study is to identify the challenges of ILCs after the earthquakes from the perspective of CPA members.

This study employed a qualitative approach. The data collection method was in-depth interviews. Semi-structured, open-ended interviews were conducted with 15 volunteers who work with ILCs, selected through purposeful sampling. The data were analyzed by using inductive thematic analysis. This study was conducted between April and May 2023.

Three main themes (problems of ILCs caused by earthquakes, access to health care, future concerns) and 11 subthemes were extracted through analyzing interviews.

ILCs experienced diverse difficulties after earthquakes. Members of CPAs observed that acute interventions to injured people and humanitarian aid activities had the first-place importance after the earthquake, while cancer-related care took the second place, anticipating devastating outcomes for the future of their care. CPAs should be supported by the public, local governments, and central government in their endeavors for ILCs.

24. **Article Title:** *A Review of Interventions for Non-Communicable Diseases in Humanitarian Emergencies in Low-and Middle-Income Countries*

**Author, Publication year:** Leff, 2021

**A sentence just for example:**
The article discusses natural disasters and armed conflicts as a combined category of humanitarian emergencies without stratifying specific strategies or measures by disaster type. But the article does stratify the impact and relevant considerations by disease type.

**List of the NCD diseases considered in this article:**
Hypertension, Cardiovascular Disease, Diabetes Mellitus, Cancer, Neuropsychiatric Disorders

**List all of the crises and disasters considered in this article**
(Only non–inside health system crises like natural disasters, conflict, displacement)
• Non-international armed conflict
• International armed conflict
• Natural disasters (e.g., Typhoon Haiyan)
• Forced displacement (refugees, IDPs)

**Main focus (included in analysis):**
Non-international armed conflict, International armed conflict, Earthquakes/Typhoons (natural disasters), Forced displacement (refugees and IDPs)

**Disease:** Hypertension
***DRUG SUPPLY***
A. Free monthly or quarterly antihypertensive medications were distributed at outpatient health facilities to Syrian refugees and vulnerable Lebanese aged 40 and above diagnosed with hypertension. This schedule was strategically designed to reduce the frequency of travel required to obtain medications, as insecurity and transportation barriers due to conflict made frequent visits impractical or unsafe. Additionally, the cost of antihypertensive medications, unaffordable to many during crisis conditions, was completely subsidized to ensure consistent treatment adherence and to prevent uncontrolled hypertension-related complications such as stroke or myocardial infarction. Distribution intervals were adjusted in line with patients’ mobility patterns and service availability disruptions due to armed conflict.

***SERVICE PROVISION***
A. The intervention incorporated structured hypertension care services into eight outpatient health facilities that served both the refugee and host populations. These services included screening for hypertension, regular monitoring, counseling, and follow-up consultations. These measures were implemented in recognition of the chronic nature of hypertension and the necessity of ongoing care to prevent acute exacerbations or target organ damage. The integration of hypertension services into pre-existing primary healthcare structures aimed to ensure a sustained continuum of care within a system strained by the influx of displaced individuals and resource scarcity.
B. The limited operational hours of health centers were cited as a significant challenge for patients with hypertension, affecting their ability to attend regular check-ups or refill medications, especially under curfew or movement restrictions imposed during the conflict. The constrained clinic hours, compounded by the conflict environment, posed risks of treatment interruptions, suboptimal disease control, and progression of hypertensive complications.

***TRAINING OF FORCES***
A. Healthcare staff delivering care for hypertensive patients received focused training from the Lebanese Cardiology Society. This training was necessitated by identified deficiencies in provider knowledge regarding hypertension management protocols, diagnosis criteria, and pharmacologic treatment regimens. Such educational reinforcement was critical in ensuring that frontline personnel could deliver standard-of-care interventions, accurately adjust therapies based on clinical presentation, and provide counseling on lifestyle modification, despite the operational disruptions caused by the conflict. The training targeted both medical and paramedical staff, aiming to standardize hypertension care delivery across multiple facilities.

***SUPPORT***
A. Patient support systems for hypertension included educational initiatives embedded in health facility operations. One-on-one education was provided to hypertensive patients upon enrollment in the program to establish understanding of the disease and the importance of medication adherence. Additionally, informal awareness sessions were conducted in waiting areas, aiming to reinforce key messages about symptom monitoring, medication use, and dietary recommendations. Furthermore, biweekly formal educational sessions were organized to consolidate patient knowledge and address misconceptions, particularly around salt intake, physical activity, and blood pressure monitoring. These interventions aimed to empower patients with the skills and understanding required for self-management of hypertension in a volatile environment.

***BUDGET PROVISION***
A. Transportation costs were identified as the primary financial barrier limiting access to hypertension services. The conflict context exacerbated this issue, as increased fuel costs, checkpoint controls, and destruction of infrastructure created logistical and economic barriers for patients seeking care. Although medications were provided free of charge, patients often faced insurmountable expenses just to reach healthcare facilities. This financial obstacle led to missed appointments and treatment gaps, directly affecting hypertension control and increasing the risk of acute cardiovascular events. Addressing this barrier was essential to ensuring the effectiveness of the overall intervention strategy.

***PREPARATION OF STRATEGIC OR OPERATIONAL PLANS***
A. A preliminary baseline needs assessment and a pilot implementation phase were conducted before scaling up hypertension services. The assessment helped quantify the prevalence of hypertension among the affected populations and informed the allocation of resources, staffing, and medication stockpiles. Operational planning included logistics for medicine procurement and storage, workforce distribution across the eight facilities, and adaptation of protocols to suit the limitations imposed by the conflict (e.g., reduced facility access, patient displacement, inconsistent electricity). These strategic preparations ensured that the intervention was responsive to the unique epidemiological and infrastructural realities of managing hypertension during crisis.

**Disease:** Cardiovascular Disease (CVD)

**DRUG SUPPLY**
A. Monthly or quarterly distribution of free medications for cardiovascular disease was implemented to overcome barriers imposed by armed conflict. These included insecurity, fluid displacement of populations, transportation challenges, and medication shortages. Cardiovascular medications were provided without cost to Syrian refugees and vulnerable Lebanese aged 40 years and older. This method aimed to minimize the frequency of travel to health centers, which was often hindered by military checkpoints, safety threats, and high costs of transport. The use of quarterly distribution was specifically intended to ensure continuity of treatment for chronic cardiovascular conditions during ongoing instability.
B. The selection of a quarterly model of drug delivery for CVD management was intended to mitigate medication access interruptions caused by unpredictability in population movement and sporadic service disruptions. This system was designed to align with conflict-related constraints on health service accessibility and patient mobility. No intervention results were stratified by CVD outcome, but the system explicitly targeted hypertension and cardiovascular disease patients.

**SERVICE PROVISION**
A. Cardiovascular disease services included both preventive and management care, tailored to the needs of displaced populations in Lebanon. Health facilities incorporated blood pressure monitoring and laboratory diagnostic capabilities relevant to cardiovascular health. These services were embedded in routine outpatient care and focused on early detection and ongoing control of cardiovascular conditions.
B. Onsite laboratory services were offered to support medical decision-making in cardiovascular patients, reducing the need for external referrals and ensuring timely clinical evaluation during active conflict. The inclusion of laboratory-based monitoring allowed for targeted therapeutic adjustments, particularly important for conditions such as hypertension where lab parameters influence clinical management.
C. Service provision strategies were adapted to the movement patterns of the refugee population, ensuring that care for cardiovascular disease was not disrupted by temporary clinic closures or supply chain interruptions. This was achieved through decentralization of service points and contingency planning for clinic operations.

**TRAINING OF FORCES**
A. Healthcare personnel involved in cardiovascular care delivery received disease-specific training from the Lebanese Cardiology Society. This training focused on clinical standards for managing hypertension and other cardiovascular disorders, particularly in crisis-affected populations. The training aimed to address a recognized gap in provider knowledge and experience in chronic disease care under humanitarian conditions.
B. The training program was part of a broader effort to raise provider competency in evidence-based cardiovascular management in unstable settings. It equipped clinicians with skills to manage medication regimens, interpret cardiovascular diagnostic tests, and counsel patients on cardiovascular risk reduction, within resource-constrained environments.

**SUPPORT**
A. Patient education was delivered using three specific formats: individualized education during initial patient intake, informal educational sessions held in waiting areas, and structured biweekly educational classes. These approaches were designed to improve patient understanding of cardiovascular disease, treatment adherence, and engagement in self-management behaviors.
B. The content of the educational support specifically addressed cardiovascular disease and hypertension, emphasizing medication adherence, dietary modification, and symptom recognition. The use of multiple delivery modes ensured broad patient reach and adaptability to different literacy levels and cultural contexts.
C. Given that cardiovascular disease was highly prevalent in the target population, this educational strategy aimed to empower individuals to take an active role in their disease control, despite the surrounding crisis. The inclusion of cardiovascular-specific messaging was deemed critical in light of the limited time and resources available to humanitarian health actors.

**Disease:** Diabetes Mellitus **Crisis: All Crises Considered in the Article (Natural Disasters, Armed Conflicts, Displacement)**

**DRUG SUPPLY**
A. In Mali, emergency kits were developed specifically to manage diabetic comas and foot complications in populations affected by non-international armed conflict. The kits included insulin, rapid-acting insulin, glucose test strips, urine test strips, lactated Ringer’s solution, normal saline, urinary catheters, antibiotics, Dakin’s solution, compresses, and bandages. These kits addressed acute complications of diabetes that were prevalent in conflict zones where medical supply chains were disrupted and healthcare infrastructure had collapsed. A total of 32 kits were used for diabetic foot complications and 15 kits for diabetic comas, directly reflecting the scale of acute diabetic emergencies in conflict conditions.
B. In the same Malian intervention, 1,814 individuals residing in active conflict zones received diabetic medications. This measure was intended to address the interruption in chronic diabetes care due to conflict-related infrastructure damage, displacement, and absence of functioning healthcare systems. Ensuring drug availability for known diabetic patients was prioritized to prevent exacerbations and acute complications.
C. In Lebanon, diabetic medications were provided free of charge on a monthly or quarterly basis to Syrian refugees and vulnerable Lebanese with type 2 diabetes. The strategy was specifically designed to overcome barriers caused by armed conflict, including reduced clinic access due to military checkpoints, high transportation costs, and shortages of medications in the market. The quarterly distribution model aimed to guarantee continuity of medication supply despite displacement or limited clinic operations.
D. In the Philippines, following Typhoon Haiyan, a surgical team reported that one-third (33.3%) of all surgical procedures were performed on patients with type 2 diabetes due to sepsis from foot injuries. Although the team had stocked metformin, they encountered shortages of insulin and sulfonylureas, which were critical to manage the high number of diabetic patients. This unanticipated demand revealed a serious gap in diabetes-specific medication preparedness during disaster deployments.
E. As a corrective measure based on these findings, it was recommended that disaster response teams include a minimum supply of long-acting and short-acting insulin, oral hypoglycemics, and glucose testing equipment in their standard emergency kits when deploying to areas with a known diabetes burden. This recommendation was based on the clear evidence of inadequacy in meeting diabetic care needs during the acute disaster response phase.

**SERVICE PROVISION**
A. In Mali, telemedical support was provided to healthcare workers managing diabetic patients in regions affected by conflict and in locations housing displaced persons. This remote guidance came from specialists at Hôpital du Mali and aimed to overcome the limited availability of local endocrinologists or diabetes specialists in conflict-affected zones. The telemedical service facilitated real-time expert input for managing complex diabetic cases, including pediatric cases with type 1 diabetes, who posed additional clinical challenges.
B. In Lebanon, laboratory testing was integrated into primary care facilities to support the management of type 2 diabetes. These tests were offered onsite to improve clinical monitoring and treatment efficacy. Laboratory capacity was essential for evaluating blood glucose levels, adjusting pharmacologic regimens, and detecting complications, especially given that patient follow-up was poor—only 10% of participants returned for three scheduled visits.
C. In the Philippines, surgical workload shifted due to a high burden of foot infections among type 2 diabetic patients. These patients required immediate surgical interventions such as debridement and amputation due to advanced sepsis. This surge in diabetes-related complications altered the clinical priorities of the foreign medical team and demonstrated the need for anticipatory planning in diabetes care during natural disasters.

**SUPPORT**
A. In Mali, children with type 1 diabetes were evacuated from conflict regions as part of the intervention by Santé Diabète. The decision to evacuate was based on the recognized complexity of managing pediatric diabetes in unstable, under-resourced settings. Lack of consistent access to insulin, pediatric-trained clinicians, refrigeration for insulin storage, and glucose monitoring devices made in-situ care unfeasible, necessitating evacuation to secure areas where adequate diabetes management was possible.
B. In Lebanon, patient education was delivered through three channels: one-on-one sessions during patient enrollment, informal awareness sessions in waiting areas, and formal biweekly group sessions. The content of these sessions was tailored to improve diabetes self-management, including adherence to medication, understanding of dietary restrictions, recognition of hypo- and hyperglycemia symptoms, and overall disease literacy. The rationale was to reduce acute diabetes complications and promote long-term glycemic control, particularly important in settings where healthcare follow-up was limited.

**TRAINING OF FORCES**
A. In Lebanon, healthcare workers involved in diabetes management received structured training from the Lebanese Diabetes Society. The training was focused on improving clinical competencies in diagnosing and managing type 2 diabetes among displaced and vulnerable populations. It addressed previously identified gaps in healthcare worker knowledge regarding diabetes protocols and medication management in crisis settings.

**PROVISION OF BUDGET**
A. In Mali, the implementing NGO reported a complete lack of response from traditional humanitarian donors for funding the diabetes intervention. This financial neglect occurred despite documented needs and the acute burden of diabetes in the conflict-affected population. The lack of financial commitment impeded the scalability and sustainability of diabetes-related healthcare delivery during the emergency.
B. In Lebanon, although diabetes medications were provided free of charge, transportation costs were repeatedly cited as a primary barrier preventing patients from accessing care. Patients were often unable to afford travel to health centers, especially when clinic locations were not within walking distance or when public transport options were limited due to the conflict.

**PREPARATION OF STRATEGIC OR OPERATIONAL PLANS**
A. In Mali, pre-deployment risk assessments were conducted to evaluate the burden of diabetes and prepare for its management in conflict-affected areas. The assessments informed logistical planning for medication procurement, design of diabetic kits for comas and foot complications, and identification of target subpopulations including internally displaced persons, residents of conflict regions, and host populations. These strategic actions ensured that the intervention could adapt to the variable access needs and disease profiles within the crisis context.

**Disease:** Cancer
**Crisis: All Crises Considered in the Article (Natural Disasters, Armed Conflicts, Displacement)**

**SERVICE PROVISION**
A. In the aftermath of a typhoon in the Philippines, the Israeli-Filipino joint field hospital adapted its cancer management protocols to the local burden of disease. The team performed ultrasound-guided fine needle aspiration (FNA) biopsies more liberally in patients with suspected thyroid malignancies. This decision was directly linked to the high prevalence of thyroid cancers and goiters in the Philippine population. Performing FNAs enabled timely diagnosis during a constrained mission timeframe, allowing for appropriate triaging and, when applicable, surgical planning.
B. Physical examinations of head and neck cancer patients were intentionally modified to detect regional lymph node metastases, even when primary tumors were small. This approach was driven by local epidemiological data indicating that more than 70% of head and neck cancers in the Philippines present with cervical lymph node involvement. Early identification of nodal metastasis informed the feasibility and urgency of surgical intervention within the limited operating capacity of the field hospital.
C. Surgical interventions were selectively performed for diagnostic, therapeutic, or palliative purposes, but reconstructive and debulking procedures were deliberately withheld when patients had poor prognoses or when postoperative continuity of care was unattainable. This triage framework accounted for the absence of advanced oncology services post-discharge, the lack of secure follow-up infrastructure, and the ethical risk of offering procedures without long-term benefit.
D. The clinical strategy emphasized maintenance of local healthcare continuity rather than introducing services not supported by the pre-existing health system. For example, surgeries were aligned with what was realistically sustainable within the local context, to prevent creating dependencies or offering false expectations of care in the post-response phase.

**PREPARATION OF STRATEGIC OR OPERATIONAL PLANS**
A. Before deployment, the Israeli surgical team assessed national data on cancer incidence and treatment capacity in the Philippines. This pre-disaster epidemiologic preparation allowed the team to anticipate a high volume of patients with head and neck malignancies and to plan accordingly. This included equipping the mission with diagnostic tools suitable for thyroid pathology, developing examination protocols specific to endemic disease patterns, and clarifying which patients would be eligible for surgery during the mission timeline.
B. The pre-deployment assessment also allowed the team to prioritize time-sensitive interventions and avoid investing resources in cases requiring extensive postoperative care that the local health system could not support. This measure ensured efficient allocation of the limited human and material resources available during a short-term relief mission.

**PROVISION OF BUDGET**
A. In the Jordanian context, cancer was identified as the most financially burdensome disease category funded under the UNHCR’s Exceptional Care Committee (ECC). Total expenditure on cancer applicants reached $181,815, with an average cost of $7,905 per applicant. This level of expenditure demonstrated the disproportionate economic demand cancer places on humanitarian medical assistance programs.
B. Among the 20 cancer-related applications for brain tumors, 15% were only partially funded, receiving on average 39% of the requested financial support. These partial fundings reflect resource limitations and administrative prioritization criteria within the ECC.
C. Six cancer applications were outright denied due to one or more of the following factors: patient ineligibility, excessive cost, or poor prognosis. These denials illustrate the constraints placed on cancer care in displacement contexts, where funding is scarce and cancer treatment is long-term, expensive, and resource-intensive. Humanitarian health financing models, in this context, systematically deprioritized oncology cases lacking favorable outcomes or requiring complex treatment regimens.

**Disease:** Neuropsychiatric Disorders
**Crisis: All Crises Considered in the Article (Natural Disasters, Armed Conflicts, Displacement)**

**SERVICE PROVISION**
A. Neuropsychiatric disorders were addressed through a tertiary-level referral system managed by the UNHCR Exceptional Care Committee (ECC) in Jordan, targeting Syrian and Iraqi refugees. The article explicitly reports that 67% of neuropsychiatric applications (n=176) were for emergency care, indicating that the ECC mechanism prioritized acute neuropsychiatric crises rather than chronic care or prevention. This approach reflects a humanitarian system structured around reactive treatment, which leaves chronic neuropsychiatric patients with limited access to sustainable mental health services.
B. Stroke was the most frequently treated neuropsychiatric condition within this service model, accounting for 16% of all ECC cases (n=41). This high frequency indicates that cerebrovascular events represent a major burden within refugee populations and were managed within an emergency care framework under the ECC. The model did not include rehabilitation or long-term management services, which are essential for stroke recovery, emphasizing a narrow clinical scope dictated by resource constraints and emergency prioritization.

**PROVISION OF BUDGET**
A. Neuropsychiatric disorders represented a substantial financial burden in the ECC funding model. Multiple sclerosis had one of the highest individual treatment costs, with an average expenditure of $7,502 per applicant. This high per-case cost reflects the complexity and chronic nature of managing neurodegenerative diseases in displaced populations, particularly where access to disease-modifying treatments and neurological specialists is limited or nonexistent.
B. Among neuropsychiatric conditions, brain tumors were also financially burdensome. Out of 20 ECC applications for brain tumors, 15% were approved for less than the full requested amount, receiving on average only 39% of the requested funding. This partial approval reflects prioritization decisions within the humanitarian system, where the cost of care, prognosis, and resource allocation pressures determine funding outcomes.
C. The ECC committee denied funding for several neuropsychiatric disorder applications based on condition type and cost-benefit analysis. Applications for disc prolapse had a 50% denial rate, cerebral palsy 33%, and brain tumors 27%. These denials were based on three explicit criteria: poor prognosis, ineligibility under ECC guidelines, or excessive cost relative to expected benefit. The data demonstrate that neuropsychiatric conditions with long-term functional impairment and poor short-term outcomes were frequently excluded from coverage under crisis-response funding protocols.

**25. Article Title:** *INSPIRATION AND ASPIRATION: Advocating for NCD care in humanitarian settings – Outcomes of a Bootcamp, June 2018*

**Author, Publication year:** Cooper, 2018
The article discusses humanitarian crises broadly—including natural disasters, conflict, and displacement—as a combined category throughout, and does not stratify specific strategies or measures by disaster type. It also does not stratify strategies or measures by specific disease type, though it consistently references NCDs collectively and the “four major NCDs.”

**List of the NCD diseases considered in this article:**
Combination of 4 major NCDs: Cancer, Diabetes Mellitus, Cardiovascular Disease, Chronic Respiratory Disease

**• List all of the crises and disasters considered in this article**

**Main focus (included in analysis):** Conflict, Displacement, Refugee Crises, Natural Disasters (Flooding)

**NCDs: Cancer, Diabetes, Cardiovascular Disease, Chronic Respiratory Disease**
**Crises: All humanitarian crises (combined)**

***STRATEGIC OR OPERATIONAL PLANNING***
A. Governments must develop national preparedness plans that explicitly include responses for people living with NCDs. This measure addresses the structural exclusion of NCDs from emergency planning, which traditionally focused on acute infectious conditions. The article states that people with NCDs are often overlooked in crisis protocols, resulting in service gaps that compromise continuity of care. The preparedness plan should assign specific responsibilities to ministries, NGOs, and community actors to ensure that NCD care is systematically integrated. It is also aimed at making governments accountable in prioritizing NCDs, aligning with Sustainable Development Goal targets.

B. Inclusion of NCDs in every humanitarian response is essential because neglecting them has led to the preventable deaths of displaced individuals. The article emphasizes that NCDs are absent from many current crisis interventions, which causes long-term health deterioration for those already living with such conditions. By mainstreaming NCDs in all emergency protocols, essential care is preserved, even in resource-depleted environments.

***SERVICE PROVISION***
C. WHO is urged to establish global technical guidelines for NCD care in crises. The rationale is to eliminate inconsistencies in how different agencies handle NCDs, particularly regarding diagnostic procedures, treatment standards, and patient triage. A standardized framework ensures that displaced populations receive coherent and non-fragmented care across borders or organizations. It also facilitates capacity building among humanitarian providers unfamiliar with chronic disease management.

D. Implementation of a person-centered model of service delivery is emphasized. The article illustrates that displaced individuals often navigate complex and disjointed care systems, resulting in overlapping prescriptions, inappropriate medication, or loss of medical records. Person-centered care empowers patients with better health literacy and aims to minimize the risk of polypharmacy or redundant diagnostics. It also addresses stigma and trust barriers by involving patients in service design.

***DRUG SUPPLY AND RESOURCE AVAILABILITY***
E. The guidelines must provide operational directives for securing uninterrupted access to essential drugs, diagnostic equipment, and monitoring devices. Humanitarian settings often face infrastructure collapse and supply chain breakdowns, which directly jeopardize access to medications like insulin, anti-hypertensives, and inhalers. By addressing procurement, warehousing, and last-mile distribution logistics, these measures mitigate acute interruptions in chronic disease treatment.

F. The article recommends the distribution of larger medication stocks to patients, following lessons from HIV program adaptations. This strategy is critical during crises where access to health facilities is sporadic. Extended medication dispensing minimizes patient travel, reduces facility congestion, and protects patients when transportation routes are insecure or blocked. This measure also strengthens system resilience by limiting dependency on daily or weekly restocking.

***INFORMATION AND DATA SYSTEMS***
G. A secure, mobile health data system must be created to support displaced NCD patients. The article highlights that current reliance on paper-based records leads to data loss during flight, language barriers in host countries, and clinical fragmentation. A digital, personalized system allows consistent diagnosis and treatment across facilities and borders. It should be compatible with local systems, accessible by patients, and protected by privacy safeguards to prevent deportation or discrimination.

***TRAINING OF HEALTH WORKFORCE***
H. Humanitarian health staff must be trained in NCD management, particularly in crisis settings. The article stresses that NCD care is often unfamiliar to emergency teams traditionally trained for trauma or infectious disease control. Task-shifting from doctors to nurses and community health workers is recommended to compensate for physician shortages and ensure service continuity. Nurses should be empowered to perform diagnostics, prescribe common chronic disease medications, and handle referrals.

***BUDGET AND FINANCING***
I. WHO is urged to define specific “best buys” for NCDs in humanitarian settings to prioritize resource allocation. This measure addresses donor reluctance to invest in NCD care due to a perceived lack of cost-effectiveness data. By identifying interventions with high health impact and favorable cost-benefit ratios, such as community screening or mobile health delivery, the “best buys” make a financial case for funding continuity.

J. The creation of a global pooled funding mechanism is recommended to address persistent underfunding. The article argues that current donor practices prioritize infectious emergencies and maternal care, sidelining NCDs despite their high mortality burden. Integrating NCDs into pooled mechanisms—like the Grand Bargain—ensures they are considered in multilateral funding decisions and prevents financial exclusion of NCD patients during emergencies.

***INTERSECTORAL COMMUNICATION AND PARTNERSHIPS***
K. Multi-sector partnerships involving humanitarian actors, local governments, researchers, private industry, and civil society must be established. The article explains that successful NCD response requires coordination across sectors for logistics, financing, advocacy, and community engagement. Lack of a shared language or priorities among these sectors impairs the coherence of NCD initiatives. The article calls for formalized communication structures to harmonize objectives and reduce redundancy.

L. Civil society is tasked with driving political momentum and donor attention toward NCDs in crises. By documenting patient experiences, care gaps, and system failures, advocacy organizations play a vital role in agenda-setting. The article underscores that NCDs receive little media attention or diplomatic visibility, and civil society must produce persuasive narratives to correct this deficit.

***COMMUNITY ENGAGEMENT AND SUPPORT***
M. Interventions should be co-designed with the community and structured around their resilience capacities. The article points out that people living with NCDs often face discrimination or exclusion during service design. Engaging them directly ensures cultural relevance, reduces stigma, and encourages self-management. The approach also fosters trust, which is essential in emergency contexts where health actors are unfamiliar or transient.

N. Mobilizing community leaders—including religious figures and health workers—is recommended to promote preventive behavior and amplify local ownership. The article cites these leaders as essential “change agents” capable of advocating for healthy diets, medication adherence, and stress reduction in contexts of displacement and instability. Their involvement also counters misinformation and fosters long-term behavioral change.

***RESEARCH AND EVIDENCE GENERATION***
O. WHO is urged to create a global, open-access platform to coordinate research, funding, and intervention design for NCDs in humanitarian contexts. This “Hub” would link NGOs, funders, and academic institutions to reduce duplication and promote evidence-based practices. The article asserts that humanitarian NCD data are fragmented and often unpublished, obstructing policy development and funding allocation.

P. NGOs are encouraged to integrate research activities into their routine programming to generate field-based evidence. The article claims that separating operations from research limits learning and innovation. Embedding research enables continuous improvement and ensures that interventions meet the evolving needs of displaced populations.

**26. Article Title:** NCD in Humanitarian Settings: Key Advocacy Asks

**Author, Publication year:** IFRC, 2025
The article discusses conflict, displacement, climate-related, and natural disaster-related crises as a combined category throughout, and does not stratify specific strategies or measures by disaster type. It also does not stratify strategies or measures by disease type.

**List of the NCD diseases considered in this article:**
Cancer, Cardiovascular Disease, Diabetes Mellitus, Chronic Respiratory Diseases, Mental Health Conditions

**• List all of the crises and disasters considered in this article**
Conflict, Violence, Natural Disasters, Climate Disasters, Health System Collapse, Displacement (including refugee and internal displacement)

**All NCDs (as a whole) – Across All Crises (Conflict, Displacement, Natural Disasters, Climate Disasters, Health System Collapse)**

***SERVICE PROVISION***
A. The article highlights the necessity of resilient primary health care systems that include NCD services across the continuum of care, emphasizing integration of NCD management in emergency health responses. This integration is essential because humanitarian crises often disrupt routine health services and limit access to ongoing treatment, which is critical for chronic conditions like NCDs that require continuous monitoring and care. The goal is to prevent acute decompensation of chronic illnesses due to service interruptions and to ensure care continuity before, during, and after emergencies.
B. The need for people-centered service models is stressed due to the increased vulnerability of displaced populations who frequently face barriers to accessing structured healthcare. People-centered models adapt to the unique social, geographic, and cultural contexts of displaced individuals, facilitating NCD care despite infrastructure breakdowns. The article notes that living in a humanitarian setting “acts as a magnifier of NCD risk,” justifying the emphasis on adapted service provision models.
C. The article outlines the importance of embedding NCD services in emergency response packages to avoid exclusion from humanitarian health priorities. Failure to include NCDs in these packages leads to disease progression, avoidable complications, and death, especially in low- and middle-income countries where 80% of premature NCD deaths occur.
D. The service provision approach also includes mental health and psychosocial support services as an integral component of NCD care in crises. The article establishes that mental health and psychosocial wellbeing are “inextricably linked to NCD outcomes,” making integrated service delivery a necessity rather than a supplement.

***DRUG SUPPLY AND SUPPLY CHAIN***
A. The article calls for the protection and continuity of medical supply chains to ensure uninterrupted availability of NCD medications and diagnostic tools during crises. It emphasizes that disruptions in medication access can lead to exacerbation of diseases like diabetes, cardiovascular diseases, and cancer. This is especially critical in protracted crises where displaced individuals may remain without regular supply for years.
B. Supply chains must be resilient not only to logistical disruptions (e.g., roads, ports) but also to policy or administrative barriers. The article implies that delays in customs clearance, lack of centralized procurement, or absence of emergency stockpiles can critically impair service continuity for NCD patients, who often depend on daily medication.
C. The need to safeguard NCD medication access is tied to the chronic and time-sensitive nature of these diseases. For example, missing antihypertensive or insulin doses due to supply failures may cause acute crises, hospitalization, or death. The article states that ensuring “uninterrupted access to essential NCD services, medicines and supplies” is a fundamental advocacy priority across all emergency scenarios.

***STRATEGIC/OPERATIONAL PLANNING***
A. The article explicitly demands the implementation of existing global, regional, and national commitments on NCD prevention and control across all humanitarian contexts. This stems from the consistent neglect of NCDs in emergency preparedness strategies, despite their significant morbidity and mortality burden.
B. The lack of formal strategic inclusion of NCDs into emergency plans results in fragmentation and inconsistent care. The article critiques the frequent absence of NCD-focused protocols in disaster preparedness or humanitarian health guidelines. Therefore, aligning response strategies with WHO frameworks and Sustainable Development Goals is presented as necessary to reduce the existing gap.
C. Operational planning for NCDs must integrate surveillance, referral systems, continuity protocols, and triage adaptations for use in unstable environments. The article calls for “flexible and people-centred approaches to NCDs,” which implies strategic customization for fluctuating operational realities in humanitarian contexts.

***FINANCING/BUDGET PROVISION***
A. Sustainable financing is identified as a foundational requirement for ensuring NCD service continuity in emergencies. The article indicates that without secured budgets, both short-term and long-term interventions are likely to collapse under the strain of resource scarcity.
B. NCDs, often under-prioritized in humanitarian financing frameworks, are at risk of being sidelined during funding cycles that favor acute infectious disease responses. The article urges prioritization of NCD financing to address this systemic oversight, arguing that failure to do so perpetuates inequities and results in preventable suffering and death.
C. Inclusion of mental health within the budget provision for NCDs is emphasized, indicating recognition of the synergistic burden of psychosocial disorders and chronic physical conditions. The article’s use of the phrase “including mental health conditions” in the same financial context as other NCDs underscores the indivisible budgetary planning required.

***INTERSECTORAL COMMUNICATION AND COORDINATION***
A. The article advocates breaking down operational silos by integrating NCDs into coordinated humanitarian health responses. It warns that fragmented communication among governmental, humanitarian, and health agencies leads to duplicated efforts, missed populations, and service inefficiencies.
B. By bringing together actors across sectors (public health, logistics, community-based systems, international agencies), coordinated responses to NCD care in crises can be ensured. This includes synchronized supply chains, unified protocols, and shared responsibility.
C. The complexity of NCD management requires not only medical input but also nutritional, psychosocial, housing, and legal support. Therefore, the article insists that intersectoral mechanisms are crucial to delivering complete and effective care in emergency settings.

***COMMUNITY SUPPORT AND ENGAGEMENT***
A. The article underscores the essential role of community engagement in maintaining NCD care during crises. By involving local populations in planning and feedback mechanisms, programs become more responsive, accessible, and resilient.
B. Community-based systems are particularly vital in low-resource, disrupted environments where formal institutions are either absent or overwhelmed. The article links community involvement directly to service continuity, noting that such engagement prevents service gaps that can result from top-down or externally imposed programming.

***HEALTH INFORMATION AND MONITORING SYSTEMS***
A. The article highlights the critical need to improve health information systems to track the burden of NCDs, service availability, and care outcomes in humanitarian settings. Without reliable data, resource allocation and intervention planning are impaired, leading to poor outcomes.
B. Monitoring systems must be adapted for use in unstable settings with mobile populations. The article notes that effective models of care must be informed by real-time surveillance and research, as static data systems fail to reflect rapidly changing needs on the ground.

***TRAINING AND CAPACITY BUILDING***
A. The article calls for building capacity across humanitarian and national health systems to recognize and prioritize NCDs during emergencies. This includes equipping frontline health workers with the knowledge and tools to identify, stabilize, and refer NCD cases appropriately.
B. Training is not limited to clinical staff. Logisticians, policy planners, and community health workers must also be trained to support NCD services, ensuring the entire system is capable of coordinated response.

**27.Article Title:** Progress on the prevention and control of non-communicable diseases and the promotion of mental health and well-being

**Author, Publication year:** WHO Secretary-General, 2025

The article discusses crises and emergencies, including humanitarian situations and the COVID-19 pandemic, as combined contexts, and does not stratify specific strategies or measures by disaster type. Similarly, the article does not stratify strategies or measures by specific non-communicable disease types, although multiple NCDs are discussed individually in terms of burden and trends.

**List of the NCD diseases considered in this article:**
Hypertension, Stroke, Ischemic Heart Disease, Chronic Obstructive Pulmonary Disease (COPD), Lung Cancer, Dementia, Diabetes Mellitus, Chronic Kidney Disease, Cancer (including Childhood Cancer), Obesity, Depression, Anxiety, Suicide, Substance Use Disorders

**List all of the crises and disasters considered in this article (Only non–inside health system crises like natural disasters, conflict, displacement):**
COVID-19 Pandemic, Humanitarian Emergencies, Displacement (including Refugees and Migrants), Climate Change, Environmental Pollution (Air Pollution)

***Disease:*** *All NCDs*

***AREA: Integration into Emergency Preparedness and Response Frameworks***
A. People living with non-communicable diseases (NCDs) are at significantly higher risk of morbidity and mortality during emergencies due to disrupted access to essential services and treatment continuity. The article states that their vulnerability is compounded in emergencies, particularly among forcibly displaced populations, where access to care is limited, and continuity of care is severely disrupted. This necessitates the integration of NCD prevention, management, rehabilitation, and psychosocial support services into all phases of emergency risk reduction, preparedness, response, and recovery to maintain health system responsiveness and service availability.
B. The article specifically highlights that failure to integrate NCD services into emergency response frameworks during the COVID-19 pandemic led to treatment delays and avoidable mortality due to disrupted services like early detection, palliative care, and treatment continuity. As a result, WHO emphasized embedding these services into preparedness strategies to ensure resilience during future crises.

***AREA: Maintenance of Essential Services***
A. The article describes how essential NCD-related services—including screening, diagnosis, medication provision, and treatment follow-up—were heavily disrupted during the COVID-19 pandemic. This interruption led to increased mortality among people with NCDs, especially those not previously treated. For example, disruptions in cancer treatment or diabetes medication access contributed to the exacerbation of health outcomes. Therefore, maintaining uninterrupted provision of these services during emergencies is vital to preventing excess mortality.
B. The article notes that continued disruption in essential NCD services has had lingering effects even after the peak of the pandemic. Health systems remain burdened with catching up on delayed vaccinations and screenings. Ensuring the resilience and operational continuity of NCD services during future crises is considered critical for minimizing preventable deaths and maintaining population health.

***AREA: Securing Supply Chains***
A. The article states that during crises, securing uninterrupted supply chains for essential medicines is critical to maintaining treatment for people living with NCDs. Medication shortages during the COVID-19 pandemic and in humanitarian emergencies have directly contributed to adverse outcomes such as unmanaged hypertension, uncontrolled diabetes, and untreated cancer or asthma.
B. WHO emphasized the importance of integrating supply chain security measures into emergency planning, including anticipating medicine demand and securing transportation routes and distribution channels, especially for primary care facilities where most NCD patients seek treatment.

***AREA: Strategic and Operational Planning***
A. The article mandates that countries must develop or update national and subnational multisectoral action plans that include strategies for the continuity of NCD services during emergencies. These plans should be informed by analysis of national capacities and vulnerabilities, ensuring that NCD management is not deprioritized in crisis contexts.
B. It is emphasized that effective strategic planning enables countries to design resilient health systems, coordinate multi-stakeholder responses, and align NCD interventions with broader health security and pandemic preparedness frameworks. This planning ensures that NCD needs are accounted for in real-time responses rather than deferred until post-crisis phases.

***AREA: Budget Allocation and Financial Protection***
A. The article underscores that most countries face severe financial constraints, which has resulted in underinvestment in NCD and mental health services. During crises, this situation becomes more pronounced as funds are diverted to immediate emergency responses. Therefore, increasing domestic financing and ring-fencing budgets specifically for NCDs is necessary to avoid service collapse.
B. Out-of-pocket expenditures rise sharply during crises, disproportionately affecting individuals with chronic NCDs who require continuous care. The article advises revising financial protection policies to ensure essential diagnostics and medications are covered, especially during emergencies, to prevent impoverishment and ensure adherence to treatment.

***AREA: Health Workforce Capacity and Training***
A. The article identifies the need to enhance workforce capacity for NCD care in crises through targeted training, supportive supervision, and adequate staffing. During the COVID-19 pandemic, reallocation of personnel to acute care led to service gaps in NCD management. This weakened the continuum of care for conditions such as hypertension, diabetes, and cancer.
B. To prevent similar disruptions, WHO recommends building a workforce capable of providing sustained NCD care during emergencies. This includes ensuring cross-training of health workers in NCD and emergency care, allowing flexibility and resilience in service delivery.

***AREA: Community-Based and Person-Centred Care Models***
A. The article promotes person-centred and community-based care approaches as essential for managing NCDs during crises. Health systems must ensure a continuum of care across primary, secondary, and tertiary levels, particularly for individuals in remote or underserved areas who may lose access to hospital-based services during emergencies.
B. Models of care must address the specific vulnerabilities of people with NCDs, including those with disabilities. This ensures that care is accessible and tailored to individual needs even when central health infrastructure is compromised.

***AREA: Intersectoral and Multistakeholder Coordination***
A. WHO mandates formal whole-of-government and whole-of-society coordination mechanisms to respond to NCD challenges during crises. This includes establishing accountability structures, coordinating across health and non-health sectors, and engaging civil society and affected populations.
B. The article highlights that 90% of humanitarian emergencies by 2024 will include a functioning mental health and psychosocial support coordination mechanism—many of which are linked to NCD management. The same model is advocated for broader NCD service integration during emergencies.

***AREA: Psychosocial Support Integration***
A. Psychosocial support is deemed integral to the health and treatment outcomes of individuals with NCDs during crises. The article points to the surge in anxiety, depression, and emotional distress during the COVID-19 pandemic as exacerbating factors in disease progression and management failure. Therefore, psychosocial services must be embedded in crisis responses for NCD patients.
B. The inclusion of mental health and psychosocial support services is recommended across all stages of crisis response, from planning to recovery, to reduce the compounding emotional burden on people with chronic diseases.

***AREA: Health System Strengthening and Primary Care Orientation***
A. The article emphasizes that a resilient health system with strong primary care capacity is the cornerstone of effective NCD response in emergencies. Health systems must be oriented to absorb shocks without collapsing core NCD services.
B. Strengthening primary care ensures that frontline facilities can manage NCDs independently during times when tertiary care services may be inaccessible. The shift to primary care models also facilitates rapid decentralization of services, ensuring continuity in high-risk and resource-limited settings.

***AREA: Use of Technology and Innovation***
A. The article supports the use of digital tools such as mobile health units and telemedicine to maintain NCD care during disruptions. These tools allow patients to continue receiving medical advice, prescriptions, and monitoring when face-to-face consultations are not possible.
B. Innovative tools also reduce dependency on centralized services, which may be non-functional in disasters. The deployment of mobile units in low-income and conflict-affected regions has allowed continuity of care for NCDs like hypertension and diabetes in the absence of stable infrastructure.

**28.Article Title:** *Vision and Framework 2030: Non-Communicable Diseases: NCD prevention, control, management, care and access to services for everyone everywhere at all times in all contexts*

**Author, Publication year:** Pratap, 2024

**A sentence just for example:**
The article discusses humanitarian emergencies, fragile settings, and protracted crises as a combined category throughout, and does not stratify specific strategies or measures by disaster type. The article also does not stratify specific strategies or measures by disease type, instead addressing NCDs as a broad group with general and integrated approaches.

**List of the NCD diseases considered in this article:**
Hypertension, Stroke, Coronary Heart Disease, Cerebrovascular Disease, Peripheral Vascular Disease, Asthma, Chronic Obstructive Pulmonary Disease (COPD), Diabetes Mellitus (Type 1 and Type 2), Cancer, Mental Health Disorders

**List all of the crises and disasters considered in this article (Only non–inside health system crises like natural disasters, conflict, displacement):**
Conflict, War, Natural Disasters, Displacement, Humanitarian Emergencies, Protracted Crises, Fragile Settings

**Disease: ALL NCDs**

**DRUG SUPPLY**

**A.** Emergency response kits must include essential supplies and medications required for the management of NCDs, such as antihypertensives, insulin, bronchodilators, and analgesics. This is necessary because humanitarian settings are often marked by disrupted pharmaceutical supply chains, rendering individuals with NCDs unable to access critical chronic therapies. The absence of such medications leads to acute exacerbations, complications, and mortality in individuals with hypertension, diabetes, asthma, and other NCDs. These kits must be stocked for long durations, ideally covering a three-month period, to ensure continuity of pharmacological treatment where resupply may be delayed or inaccessible.

**B.** Where feasible, cash transfer mechanisms (e.g., medication-specific cash cards) should be adopted to facilitate access to essential medicines during crises. This intervention ensures that patients with NCDs maintain therapeutic continuity even in displacement settings or where conventional supply chains are non-functional. This measure was proposed in recognition of financial barriers and logistical challenges commonly encountered in disrupted contexts, particularly in protracted crises or fragile environments, where fixed distribution channels may no longer be reliable.

**SERVICE PROVISION**

**C.** Mapping and disseminating information on available health services is necessary to facilitate timely access to care for individuals with NCDs during emergencies. Displacement, infrastructure collapse, and breakdown of information networks impair patients' ability to locate service points. Therefore, systematically identifying and publicizing where NCD care is available helps mitigate the risk of untreated exacerbations and ensures continuity in monitoring, medication refills, and acute management.

**D.** Integrating NCD services into emergency response plans is required to institutionalize preparedness and ensure protocols for NCD screening, diagnosis, and treatment are embedded within emergency response structures. During crises, the health focus is typically on communicable diseases and trauma; failure to include NCDs in plans perpetuates neglect. The framework emphasizes adapting existing guidelines to suit emergency contexts, recognizing that NCDs demand chronic care models, not acute-event-focused responses.

**E.** Supporting healthcare navigation for patients with NCDs is necessary throughout the crisis continuum to prevent deterioration due to care fragmentation. People with NCDs often require coordinated interventions across specialties and timeframes. In emergencies, this complexity is intensified by systemic breakdowns, mobility, and lack of integration across health actors. Navigation support helps individuals interpret service availability, eligibility, and access procedures to avoid loss to follow-up or therapeutic non-adherence.

**F.** Preventive interventions aimed at halting disease progression, such as managing diabetic foot ulcers or COPD action planning, are critical during all crisis phases. These are not merely maintenance measures but active interventions to prevent disabling complications. In the absence of such support, individuals with diabetes, for instance, may progress to infection and amputation, while those with COPD may suffer life-threatening exacerbations. These interventions thus preserve functional status and prevent overburdening emergency services with avoidable acute conditions.

**G.** Special food provision for individuals with NCDs, particularly those with dietary restrictions such as diabetes, cardiovascular diseases, and renal conditions, must be considered in emergency contexts. Food aid packages often fail to address these needs, exacerbating glycemic control, fluid retention, or hypertension. Inclusion of appropriate nutritional options is vital to prevent deterioration and complications induced by unregulated dietary intake.

**SUPPORT**

**H.** Structured self-care support must be established for individuals with NCDs during the preparedness phase and maintained through crises. For example, training individuals to recognize early signs of asthma exacerbations, adhere to antihypertensive therapy, or perform diabetic foot care contributes to autonomy and resilience. This measure recognizes that formal health services may be intermittently available and that equipping patients with the knowledge and tools to manage their conditions is essential to prevent deterioration between encounters.

**PREPARATION OF STRATEGIC OR OPERATIONAL PLANS**

**I.** Establishing patient registries during the preparedness phase is crucial for identifying and prioritizing high-risk individuals such as insulin-dependent diabetics or those with congestive heart failure. These registries ensure that emergency responders are aware of vulnerable individuals and can mobilize resources accordingly. This measure addresses the invisibility of chronic conditions in emergency rosters and allows for triaging based on chronic disease burden, not only acute trauma.

**J.** Emergency plans must formally include NCD-specific protocols to prevent neglect in resource distribution, staffing, and clinical planning. Failure to include NCDs has historically resulted in catastrophic gaps in care, with people suffering myocardial infarctions, diabetic crises, or asthma attacks receiving delayed or no treatment. The inclusion ensures parity between NCDs and infectious or trauma-related care in emergency settings.

**TRAINING OF FORCES**

**K.** Volunteers and staff must be trained in the provision of mental health and psychosocial support as part of the integrated NCD response. This includes psychological first aid, lay counseling, and recognition of mental distress in individuals with chronic physical illnesses. The rationale stems from the established bidirectional relationship between mental health and physical NCDs, where untreated anxiety, depression, or PTSD may worsen self-care, adherence, and disease outcomes.

**CREATION OF STORAGE RESOURCES**

**L.** Emergency NCD supply kits must be created and stored in advance to secure care continuity in the face of logistical disruption. These kits must include medications, monitoring tools, and educational materials. Stockpiling is especially critical in disaster-prone or conflict-affected areas where regular supply chains are vulnerable. Prepositioned resources mitigate the delays that commonly follow infrastructure collapse and ensure rapid deployment.

**IMPROVEMENT OF INTER-SECTORAL COMMUNICATIONS**

**M.** The response must involve coordinated action with sectors outside of health, such as shelter, protection, and livelihood. Mental health and psychosocial problems often originate from displacement-related stressors including family separation, income loss, and inadequate living conditions. These factors affect NCD outcomes by reducing access, adherence, and patient motivation. Therefore, comprehensive NCD support depends on integrated, multisectoral responses.

**PROVISION OF BUDGET**

**N.** Long-term, community-based programmes for NCDs must receive dedicated financial investment. The rationale lies in the chronic nature of NCDs, which contrasts with the short-term funding cycles typical of humanitarian interventions. Lack of sustained funding leads to interruptions in service, workforce attrition, and erosion of programmatic effectiveness. Financial allocation ensures resilience and capacity to deliver care beyond the acute response.

**OTHER**

**O.** Use of personal health records (PHRs) should be promoted among displaced persons and mobile populations. These documents enable individuals to carry essential medical information across regions and providers, reducing the risks of incorrect treatment, redundant testing, and delays in care initiation. PHRs are especially valuable in fragmented health systems where continuity depends on patient-carried documentation.

**P.** Community education programmes focusing on modifiable risk factors (e.g., tobacco, unhealthy diets, physical inactivity) and preventive immunizations (HPV, HBV) may be introduced during recovery or protracted crisis phases. These interventions reduce the incidence and severity of NCDs in populations at risk and are especially relevant when normal public health programming has been interrupted.

**Q.** Screening of asymptomatic individuals for NCDs is not recommended in acute emergency settings. Instead, targeted case-finding in patients presenting to healthcare clinics should be prioritized to conserve resources and focus attention on those with evident health needs. This measure is proposed due to the logistical and ethical challenges of mass screening in chaotic, resource-limited environments.

**29.Article Title:** *Implementing and evaluating integrated care models for non-communicable diseases in fragile and humanitarian settings*

**Author, Publication year:** Vijayasingham, 2024

**A sentence just for example:**
The article discusses humanitarian crises in general (such as displacement, conflict, and natural disasters) as a combined category without stratifying specific strategies or measures by disaster type. It also does not stratify the impact or considerations by specific disease type, despite discussing several NCDs.

**List of the NCD diseases considered in this article:** Hypertension, Diabetes Mellitus, Depression, Anxiety, Stroke, Myocardial Infarction, Retinopathy, Nephropathy, Neuropathy, Hyperlipidemia

**List all of the crises and disasters considered in this article (Only non–inside health system crises like natural disasters, conflict, displacement):**
Displacement, Armed Conflict, Economic Crisis, Natural Disasters (earthquakes, droughts, flooding), Infectious Disease Outbreaks, Humanitarian Crisis (general)

**ALL NCDs**

**ALL CRISES**

***SERVICE PROVISION***
A. Integrated care delivery models must be implemented in humanitarian settings for people living with NCDs (PLWNCDs) because these individuals often experience multimorbidities such as diabetes, hypertension, depression, and anxiety, which require coordinated and continuous treatment across various services. In fragmented systems, patients face interruptions in care during crises, which exacerbates disease complications. Integrated care facilitates access to a package of services (e.g., diagnostic testing, pharmacological and non-pharmacological treatment, physiotherapy, rehabilitation, and palliative care), which is crucial during disruptions caused by displacement or natural disasters. The rationale is that when these services are delivered in isolation, it leads to poor health outcomes and higher rates of complications, such as retinopathy and nephropathy among refugees presenting late to care.

B. Co-location of services increases informal collaboration, fosters communication between multidisciplinary teams (such as physical rehabilitation and mental health providers), and results in faster response to patient needs. In the CAJA model example, primary healthcare, physical rehabilitation, and mental health services were initially co-located but operated in silos. The integration of these into one model improved clinical coordination and targeted multimorbid NCD needs.

C. Services must be organized around the individual’s needs and not around a single disease. The people-centred model emphasized in the article suggests PLWNCDs often have functional limitations or mental health burdens (e.g., PTSD linked to increased risk of diabetes). Thus, a model that responds holistically rather than disease-by-disease enables individuals to maintain functionality and avoid deterioration in crises. Without this, the article states, patients often present late, with advanced complications such as myocardial infarction and neuropathy.

***DRUG AND EQUIPMENT SUPPLY***
A. Humanitarian crises interrupt supply chains and restrict access to essential medicines. The article explains that shortages in insulin, blood pressure monitors, and blood glucose monitoring equipment directly compromise disease management for diabetes and hypertension. In Lebanon, insulin subsidies were lifted in 2021 during a political and economic crisis, creating a situation where both refugees and host populations lost access to life-saving treatment. This has been especially harmful in Akkar Governorate, where 50% of households are affected by NCDs, and the region has the lowest medication access in the country.

B. Inadequate drug supply was associated with increased out-of-pocket spending. This financial burden limits patients' ability to adhere to treatment regimens, worsening clinical outcomes. Without blood pressure and glucose monitoring, early signs of hypertensive crisis or diabetic ketoacidosis may go undetected, leading to emergency situations during crisis conditions.

***TRAINING OF FORCES***
A. The implementation of integrated NCD care in humanitarian settings requires trained primary healthcare workers capable of delivering complex, multidisciplinary care. The article reports that without adequate provider training, care becomes fragmented and disease control deteriorates. For example, trained generalists are better able to manage multimorbid conditions (e.g., diabetes with depression) and refer appropriately, which is vital when specialists are unavailable during crises. Inadequate training has been shown to reduce referral pathway effectiveness and treatment fidelity.

***STRATEGIC AND OPERATIONAL PLANNING***
A. Strategic planning through participatory approaches—such as theory of change exercises—involves local stakeholders including ministries of health and humanitarian responders. In the CAJA model, this led to identification of alignment priorities in human resource deployment, patient information systems, and empowerment strategies. This planning allowed the system to address patient-level needs even amidst regional displacement crises.

B. Using structured implementation research frameworks such as RE-AIM PRISM helped evaluate the integrated NCD care model under real crisis conditions. These frameworks allowed implementers to track effectiveness (e.g., control of blood sugar and blood pressure), service reach, and patient empowerment. Without such strategic tools, it would be impossible to adapt care plans to rapidly shifting crisis contexts.

***BUDGET AND FINANCING***
A. Humanitarian NCD care must be supported by long-term, pooled budgets. The article notes that short-term and shrinking humanitarian funds are a key barrier, especially in protracted crises. Underfunding leads to interrupted services, staff shortages, and absence of crucial diagnostics. Donors must adopt funding models that support sustained NCD treatment infrastructure over time.

B. High out-of-pocket costs, especially for diagnostics and medications, limit patient access. PLWNCDs in fragile settings often pay for services such as insulin, mental health therapy, or blood testing. The article notes that this results in decreased adherence and late-stage presentation. Such financial barriers are even more pronounced during economic crises that coincide with displacement or natural disasters.

***INFORMATION AND RECORD SYSTEMS***
A. Integrated care requires the use of joint health records to ensure continuity across disciplines. The article stresses that without unified information systems, healthcare teams (e.g., physicians, physiotherapists, mental health workers) cannot share data, resulting in fragmented care. This impedes the identification of disease progression, especially in cases with overlapping symptoms (e.g., anxiety exacerbating hypertension). A unified system allows better longitudinal management, particularly for patients experiencing unstable living conditions.

***INTERSECTORAL COMMUNICATION AND COORDINATION***
A. The article emphasizes the importance of formal interprofessional collaboration to break silos between medical, mental health, rehabilitation, and social care. In the CAJA model, co-location of services alone was not sufficient; structured interdisciplinary communication (e.g., joint meetings, case discussions) was essential to ensure comprehensive patient care. Lack of coordination led to duplicative or missed care interventions, which is critical in multimorbid NCD cases.

B. Successful coordination requires clear role definition, leadership stability, and aligned goals across sectors. Where these were absent, professional protectionism and confusion in task responsibilities emerged, obstructing integrated care delivery. For example, when referral pathways between rehabilitation and chronic care teams were unclear, patients with diabetic neuropathy failed to receive timely physical therapy.

***SUPPORT AND EMPOWERMENT OF PATIENTS***
A. Empowering patients through structured involvement in their own care is vital for effective chronic disease control. The article describes that in integrated programs, patients were engaged through health education and counselling, enabling them to monitor symptoms, adhere to treatments, and communicate effectively with providers. This is particularly important in unstable settings where frequent clinic visits may not be feasible. Empowerment mitigates disease decompensation between visits.

***EVALUATION AND RESEARCH***
A. Real-time monitoring through mixed-methods research enables program adaptation under evolving crisis contexts. The CAJA model used interviews, observations, and clinical data to refine its implementation. This process was used to determine whether goals such as improved blood pressure and sugar control, referral uptake, and patient engagement were being met.

B. Economic evaluations, including sensitivity analyses, were recommended to assess the feasibility and long-term sustainability of integrated NCD interventions. Costing studies showed that integrated models could reduce overall expenses for both patients and health providers by avoiding complications that necessitate hospitalization or emergency care.

C. Research was advised to be inclusive of disability, gender, and migration dimensions to ensure that interventions addressed the social determinants that exacerbate NCDs in crises. For example, Syrian refugees with multiple vulnerabilities were less likely to access services, hence needed targeted research-informed strategies.

***USE OF EXISTING PLATFORMS AND INFRASTRUCTURE***
A. Health systems should leverage existing delivery platforms—such as HIV, TB, and maternal health services—to incorporate NCD care. This is especially feasible in humanitarian settings where these programs are better established. The article explains that TB programs, for example, can be used to screen for and treat comorbid diabetes, which frequently coexists due to physiological and social vulnerabilities in displaced populations.

***HEALTH LITERACY AND DISEASE COPING STRATEGIES***
A. Low health literacy, coupled with limited access to disease coping mechanisms, worsens disease outcomes in crisis settings. The article notes that in displacement contexts, individuals often lack knowledge about early symptoms, medication adherence, and dietary practices, particularly relevant to diabetes and hypertension. Health literacy interventions are critical to empower patients, especially where professional support is intermittent.

***HUMAN RESOURCE PLANNING***
A. The article identifies frequent staff turnover, inadequate numbers of trained professionals, and burnout as critical barriers in NCD care continuity. Effective human resource strategies must include stable leadership, defined roles, and adequate compensation to ensure staff retention. These conditions are especially strained in crises, leading to disruptions in treatment for complex multimorbid patients.

***DIGITAL TOOLS***
A. The article recommends using digital health record systems instead of paper-based records to improve data access, tracking, and coordination. This is especially important during disasters when paper records may be lost or inaccessible. Electronic systems also allow remote monitoring and support for PLWNCDs in hard-to-reach areas.

***COMMUNITY AND ETHICAL ENGAGEMENT***
A. Co-production of care models with PLWNCDs and local communities enhances cultural acceptability and trust. The article states that participatory approaches can identify overlooked patient needs, particularly in marginalized populations, such as displaced individuals with NCDs who face layered vulnerabilities and stigma.

***PREPAREDNESS AND FLEXIBILITY***
A. Flexibility in implementation methods allows rapid response to changes in humanitarian settings. The CAJA model’s adaptability was supported by embedding evaluation tools into the rollout, which enabled mid-course corrections and better targeting of resources for PLWNCDs.

**30. Article Title:** Diabetes Care in Humanitarian Settings

**Author, Publication year:** Sylvia Kehlenbrink, 2023

Despite the increasing prevalence of diabetes in populations experiencing humanitarian crisis, along with evidence that people living with diabetes are at higher risk for poor outcomes in a crisis, diabetes care is not routinely included in humanitarian health interventions. We here describe 4 factors that have contributed to the inequities and lack of diabetes inclusion in humanitarian programmes: (1) evolving paradigms in humanitarian health care, (2) complexities of diabetes service provision in humanitarian settings, (3) social and cultural challenges, and (4) lack of financing. We also outline opportunities and possible interventions to address these challenges and improve diabetes care among crisis-affected populations.

**31. Article Title:** *NCD care in humanitarian settings: a clarion call to civil society*

**Author, Publication year:** Cooper, 2018
The article discusses conflict, war, and natural disaster–related crises as a combined category (under the umbrella of "humanitarian crises") throughout and does **not** stratify specific strategies or measures by disaster type. The article **does not stratify** the strategies or measures by disease type in a detailed manner. While it mentions diseases separately and includes some disease-specific notes (e.g., insulin storage for diabetes), the overall measures are discussed collectively for NCDs.

**List of the NCD diseases considered in this article:**
Diabetes Mellitus, Hypertension, Cardiovascular Disease, Chronic Lung Disease (including Asthma and COPD), Cancer, Mental Health Disorders (e.g., PTSD, Depression), Hyperlipidemia

**List of the crises and disasters considered in this article (Only non–inside health system crises):**
Conflict, War, Natural Disasters, Displacement, Refugee Situations, Humanitarian Crises (including protracted and complex emergencies)

**Disease:** All NCDs

***DRUG SUPPLY***

A. The WHO NCD Kit was developed specifically to address the lack of noncommunicable disease medications in emergencies. The article states that earlier emergency health kits (IEHK) did not adequately consider NCDs, which created a critical gap in care. The WHO EMRO NCD Kit was introduced to complement the IEHK, supplying essential NCD medications and tools like glucose strips, glucometers, and syringes. It was modular and customizable, allowing countries to order specific sub-modules based on need. This addressed the issue of continuity of care during crisis transitions by ensuring essential medication access for diseases like hypertension and diabetes, especially where supply chains were destroyed or non-functional.

B. Cold-chain disruption was highlighted as a critical barrier to diabetes care due to insulin storage challenges. The article described a specific solution: recognition that insulin can be safely stored in clay pots without refrigeration. This adaptation ensured that even in contexts lacking refrigeration infrastructure, insulin-dependent patients could maintain their treatment regimens.

C. The article addressed affordability by promoting low-cost “humanitarian” pricing models for NCD medications. It cited cases where cost-sharing mechanisms introduced by host governments (e.g., Jordan in 2014) made treatment unaffordable for many refugees, pushing them out of care. This reinforced the necessity of subsidy models to ensure equitable access for displaced populations.

D. In Dadaab refugee camp, the Kenyan Red Cross provided NCD drugs including insulin and metformin to over 450 patients. The article listed that 313 received hypertension treatment, 78 diabetes treatment, 23 asthma treatment, and 16 cardiovascular disease treatment. Drug provision was thus fundamental to maintaining disease stability among displaced populations.

***SERVICE PROVISION***

A. The IRC mobile clinics in Syria, Yemen, and Libya were deployed to deliver basic NCD services in settings where static facilities were unavailable. Each clinic included staff such as doctors, nurses, and pharmacists. Services were focused primarily on hypertension, cardiovascular disease, and diabetes due to their prevalence and manageable nature in mobile settings. Clinics reached 29,015 people in Syria alone in 2017, of whom approximately 14.5% received NCD care. The use of mobile clinics overcame access barriers in conflict zones and reduced interruption in treatment regimens.

B. In Jordan, MSF operated a vertical primary-level NCD program targeting cardiovascular disease, diabetes, and chronic lung disease. It provided physician consultations, health education sessions, and blood pressure and sugar monitoring. Among patients retained in the program for 6–12 months, there were measurable improvements in blood pressure and blood sugar levels. This indicated the effectiveness of structured NCD care in refugee-hosting communities even amid humanitarian instability.

C. In the Democratic Republic of Congo, MSF established a nurse-led outpatient diabetes program (IDC-OPD) in a Ministry of Health hospital. While physician involvement was limited, the program integrated nutritional support, psychosocial services, and consistent monitoring. Patients were given health passports to record medical history and treatment. Annual costs were estimated at €183–415 per patient—comparable to chronic HIV care—demonstrating both feasibility and affordability.

D. In Dadaab, the Kenyan Red Cross used community health workers to provide services to a NCD-affected population. Medication distribution was coupled with lab services including blood sugar, liver, and kidney function tests. These services ensured accurate diagnosis and treatment management in a remote setting with limited healthcare access.

E. UNRWA implemented the Microclinic model in Palestinian refugee camps across Jordan, Gaza, the West Bank, and Lebanon. This approach trained over 1,059 healthcare workers and mobilized 114,134 participants as health ambassadors focusing on medication adherence, dietary modification, physical activity, and self-monitoring. The model was effective in embedding NCD care within community life, promoting sustainability and accountability even when formal services were intermittent or disrupted.

F. MSF’s 2016 NCD Guidelines included mental health integration, reflecting the observed comorbidity of hypertension and cardiovascular disease with mental health conditions such as PTSD and depression. Particularly in humanitarian crises, trauma and isolation elevated psychological distress, which exacerbated the management of NCDs. Integrating psychosocial support into physical health services was thus essential to holistic care delivery.

***SUPPORT***

A. In Dadaab, peer groups organized by community health workers provided education, counseling, and social support to 450 patients living with NCDs. These support groups also functioned to reduce stigma, improve medication adherence, and offer platforms for health education. An estimated 1,700 additional individuals were reached through community awareness sessions, further reinforcing self-care practices.

B. MSF integrated mental health services into its programs after observing that patients with NCDs—particularly diabetes—experienced significant psychosocial distress. In the DRC, for example, diabetes was perceived by patients to be more socially and psychologically disruptive than HIV. Psychosocial officers were appointed to mitigate these impacts, improving patient coping mechanisms and self-management.

***PREPARATION OF STRATEGIC OR OPERATIONAL PLANS***

A. The article discussed the absence of standardized emergency protocols for NCDs and the recent development of simplified treatment guidelines for humanitarian use. These were designed by organizations like MSF and PCI and included protocols for hypertension, cardiovascular disease, diabetes, asthma, COPD, and palliative care. These protocols addressed the challenge of healthcare delivery in contexts where local guidelines were unfeasible due to disrupted infrastructure and displaced populations.

B. The SPHERE Guideline 2018 introduced four key actions for NCD care in emergencies: identifying pre-disaster service availability, implementing phased response strategies, integrating NCD care into all health system levels, and establishing prevention and preparedness programs. These actions ensured that NCDs were no longer overlooked in emergency planning and response.

***TRAINING OF FORCES***

A. PCI conducted "training the trainer" initiatives across seven African countries and Syria to equip healthcare workers with the clinical skills and systems strategies required for effective NCD care. The cascade model enabled local ownership and scale-up of NCD services, critical in protracted crises where external workforce support might be inconsistent.

B. In Syria, 227 local healthcare providers were trained on the use of the WHO NCD Kit within six months of its deployment. Training ensured the correct usage of the kit and empowered local staff to manage NCDs even when international assistance was limited or delayed.

***CREATION OF STORAGE RESOURCES***

A. Emergency preparedness planning included recommendations for pre-crisis stockpiling of essential NCD medicines. This was considered essential to prevent treatment interruptions during disasters and to ensure a rapid response in managing disease flare-ups among vulnerable populations.

***IMPROVEMENT OF INTER-SECTORAL COMMUNICATIONS***

A. In the DRC outpatient program, MSF introduced individual patient health passports to document diagnoses and treatments. This was necessary in environments with fragmented health information systems and frequent displacement.

B. The Dharma mobile data platform facilitated real-time tracking of NCD-related data and medical supply shipments across regions (e.g., The Netherlands to Turkey and Turkey to Syria). This streamlined operations and ensured evidence-based resource allocation during crises.

C. Cross-border mapping of healthcare facilities was proposed to ensure continuity of care for patients moving between countries, which is a common phenomenon in protracted conflicts and mass displacement.

***PROVISION OF BUDGET***

A. The article stated that <2% of global development assistance for health is allocated to NCDs, emphasizing the need for increased, sustainable funding in humanitarian settings. Without this shift, the long-term burden of untreated NCDs would escalate, particularly among vulnerable displaced populations.

B. Partnerships with funders like FDA, World Bank, and ECHO were cited as emerging financial contributors. Advocacy efforts were being employed to secure their commitment to integrate NCD care within broader humanitarian health portfolios.

***USE OF DIGITAL TOOLS AND MONITORING SYSTEMS***

A. A pilot mHealth app in Lebanon demonstrated measurable improvements in care quality: increased documentation of BMI and blood pressure, enhanced lifestyle counseling, and a rise in patient-reported involvement in treatment decisions from 69% to 95%. This evidenced the app’s utility in reinforcing clinical standards under crisis conditions.

B. A new app for community health workers was under development by MIT, Johns Hopkins University, and IRC. It aimed to document NCD consultations, enhance data collection, and maintain treatment tracking at the community level—ensuring continuity and effectiveness in settings lacking centralized records.

**32. Article Title:** *Strengthening the health system response to NCDs in humanitarian settings*

**Author, Publication year:** Bajdan, 2025
The article discusses natural disasters and conflict-related humanitarian crises as a combined category throughout, and does not stratify specific strategies or measures by disaster type. But the article does stratify the impact and relevant considerations by disease type.

**List of the NCD diseases considered in this article:**
Hypertension, Cardiovascular Diseases, Diabetes Mellitus (Type 1 and Type 2), Chronic Respiratory Diseases, Mental Health and Neurological Disorders, Chronic Kidney Disease, Cancer, Asthma, Chronic Obstructive Pulmonary Disease (COPD)

**List all of the crises and disasters considered in this article (Only non–inside health system crises like natural disasters, conflict, displacement):**
Natural Disasters, Armed Conflict, Forced Displacement, Climate-Change–Induced Crises, Public Health Emergencies, Climate Migration, Resource Conflict (e.g., over water and land)

**Disease: Hypertension**
**Crisis: All Crises (General Humanitarian Settings)**

***SERVICE PROVISION***
A. In Cox’s Bazaar, Bangladesh, a community outreach programme specifically identified a hidden population with previously undiagnosed hypertension. Screening activities conducted among Rohingya refugees uncovered new hypertension cases. This service provision was not incidental; the article directly attributes the identification of these individuals to targeted NCD screening embedded within a structured outreach effort. The programme also established new primary care services that could manage hypertension, ensuring that once identified, individuals had access to follow-up, monitoring, and consistent care. The structured approach included NCD awareness campaigns, health worker engagement, and service continuity—factors that contributed to diagnosis and management of hypertension within displaced populations.
B. In the Bidibidi refugee settlement, clinic days were explicitly structured for NCD management, and this included hypertension as part of the targeted conditions. The article notes a 75% increase in clinic attendance and a 65% rate of improved condition control among enrolled patients within six months. Though this includes all NCDs, hypertension was clearly listed among the managed conditions. The establishment of clinic days created predictable, accessible service access points for hypertensive patients, facilitating regular blood pressure monitoring and medication dispensing.

***TRAINING OF FORCES***
C. In Cox’s Bazaar, local healthcare workers were trained specifically on NCD care, which included modules on hypertension. This training allowed them to perform blood pressure screenings, recognize clinical signs, and initiate basic management protocols. This addressed the significant gap in provider knowledge in humanitarian contexts and ensured that hypertension was not overlooked during general patient consultations. The training was operationally essential to enabling decentralized diagnosis and care at the community level.

***DRUG SUPPLY***
D. The same Cox’s Bazaar programme included the establishment of reliable medical supply chains. Medications for hypertension management were part of the essential drugs delivered. This was necessary because humanitarian settings often face inconsistent medication availability, leading to treatment interruptions and poor disease control. Including antihypertensive drugs in the secured supply ensured continuity for those newly diagnosed and those already under treatment.
E. The WHO NCD Kit, used in humanitarian emergencies, includes essential medicines for the management of hypertension. This kit was designed to support 10,000 people over three months and includes both medications and basic diagnostic tools. The article emphasizes that the kit bridges gaps in access when regular supply chains are disrupted, allowing for uninterrupted hypertension treatment in emergencies. It also facilitates immediate deployment during crises and pre-positioning for preparedness planning.

***OPERATIONAL PLANNING / EMERGENCY PREPAREDNESS***
F. The WHO NCD Kit was designed with hypertension as a core condition to be addressed in emergency preparedness plans. Its modular structure allows it to be pre-positioned in health facilities or mobile units. This ensures that hypertension management does not halt during acute disruptions caused by displacement, infrastructure damage, or logistical bottlenecks. It is also highlighted as an entry point for integrating NCD care into emergency preparedness protocols, meaning that hypertension is formally recognized in structured emergency response mechanisms.

***STRATEGIC PLANNING / PROGRAMME DESIGN***
G. The article describes that in Bidibidi refugee settlement, a strategic design was implemented to structure NCD care into primary care delivery. Clinic days were dedicated to conditions like hypertension. This institutional structuring allowed healthcare workers to consistently follow up with hypertensive patients. The scheduling of these services gave predictability and created a supportive routine necessary for chronic disease management, particularly for conditions like hypertension that require long-term treatment adherence.

***MONITORING AND FOLLOW-UP***
H. In the Bidibidi refugee settlement, hypertensive patients were tracked through village health teams and “buddy groups.” This allowed for ongoing patient engagement and supported treatment adherence by creating social and logistical structures to remind, encourage, and follow up with patients. These groups also provided data back to the system, improving understanding of treatment effectiveness and retention.

***PATIENT EMPOWERMENT / SELF-MONITORING***
I. In Burundi, patients diagnosed with hypertension were provided with home monitoring devices such as blood pressure cuffs. They were trained to self-monitor their condition, and their readings were tracked by community health workers. This enabled early identification of complications or treatment failures. It also reduced dependency on overburdened health facilities by shifting part of the disease management responsibility to the patients themselves, which is critical in low-resource, crisis-affected settings.

**Disease: Cardiovascular Diseases (CVDs)**
**Crisis: All Crises (General Humanitarian Settings)**

***DRUG SUPPLY***
A. The WHO NCD Kit, developed for use in humanitarian emergencies, explicitly includes medicines to treat hypertension and cardiac conditions. Cardiovascular diseases are addressed through this inclusion of cardiovascular-specific medications within Module 1 of the kit. The article specifies that the NCD Kit is intended to treat 10,000 people over a period of three months in disrupted or crisis-affected health systems. Its use ensures that individuals with cardiovascular conditions continue to receive necessary pharmacological management when normal supply chains are inoperable or severely limited. (no specific p-value/OR/CI reported)

***HEALTH TECHNOLOGY AND EQUIPMENT PROVISION***
B. The WHO NCD Kit also includes essential equipment for the management of cardiovascular conditions, such as stethoscopes and sphygmomanometers. These devices are explicitly named in the article as components required for cardiac condition assessment and monitoring. The availability of this equipment in field hospitals, mobile clinics, and primary health centres enables accurate diagnosis and follow-up of cardiovascular patients under crisis conditions. (no specific p-value/OR/CI reported)

***TRAINING OF FORCES***
C. Between 2014–2020, Primary Care International worked in partnership with UNHCR to train clinical staff in refugee settings, including Jordan, Rwanda, Cameroon, and Uganda, with specific emphasis on improving management of cardiovascular diseases. The article explicitly states that cardiovascular diseases were one of the primary conditions targeted by these trainings. Through a cascade training model, NCD champions were developed who led further capacity building among healthcare providers. This led to improved early case detection of cardiovascular diseases and enhanced quality of care in these contexts. (no specific p-value/OR/CI reported)

***STRATEGIC PLANNING / PROGRAMME DESIGN***
D. The article notes that cardiovascular diseases were one of the central focuses of the structured care delivery project conducted from 2014–2020 by Primary Care International and UNHCR. The project introduced patient registers, recall systems, and structured consultations specifically for conditions including cardiovascular diseases. These structural interventions facilitated timely follow-up and consistent patient monitoring, reducing the occurrence of late-stage cardiovascular complications. (no specific p-value/OR/CI reported)

***SERVICE PROVISION***
E. The article highlights the integration of cardiovascular disease management into primary healthcare services in refugee settings as a key achievement of the UNHCR–Primary Care International partnership. The care model included evidence-based clinical guidelines adopted by clinicians, with cardiovascular diseases being among the explicitly named target conditions. This integration ensured that cardiovascular disease management was embedded into routine service delivery and not treated as a secondary priority in humanitarian contexts.

**Disease: Diabetes Mellitus (Type 1 and Type 2)**
**Crisis: All Crises (General Humanitarian Settings)**

***SERVICE PROVISION***
A. In Hargeisa, Somaliland, a community-based programme was implemented to provide care for 200 children living with type 1 diabetes. This programme included weekly health information sessions for children and their carers, regulated and uninterrupted supplies of insulin, glucose monitoring devices, and syringes, as well as the establishment of regular clinical appointments with a dedicated physician. The programme also implemented a call-and-recall system and distributed information, education, and communication materials translated into local dialects. As a result of this multifaceted service provision model, 89% of children achieved optimal glycaemic control, the frequency of abscesses was reduced, and most children were able to return to school. (no specific p-value/OR/CI reported)

***DRUG SUPPLY***
B. The Hargeisa diabetes care programme provided regulated supplies of insulin and glucose monitoring tools. This ensured consistent access to essential diabetic treatment products necessary for glycaemic control in children with type 1 diabetes. The provision of insulin was systematic and uninterrupted, forming the pharmacological foundation of the intervention and contributing to the reported improvement in glycaemic outcomes. (no specific p-value/OR/CI reported)
C. The WHO NCD Kit, revised in 2022, includes medications and equipment for the management of type 2 diabetes, as well as a cold chain module containing human insulin in 10 ml vials. This cold chain provision ensures the safe storage and transport of insulin, which is critical in crisis settings where temperature control is often compromised. The inclusion of diabetes-specific supplies within this pre-positioned kit allows for continuity of care during emergencies. (no specific p-value/OR/CI reported)

***HEALTH TECHNOLOGY AND EQUIPMENT PROVISION***
D. Module 5 of the WHO NCD Kit includes a glucometer with 300 test strips and 300 lancet pens per patient for three months. This equipment directly supports the management of diabetes by enabling regular blood glucose monitoring, which is essential for both type 1 and type 2 diabetes. The inclusion of this module ensures that individuals with diabetes can maintain appropriate self-monitoring routines during crises. (no specific p-value/OR/CI reported)

***STRATEGIC PLANNING / PROGRAMME DESIGN***
E. The diabetes care programme in Hargeisa incorporated structured clinic visits, use of recall systems, and local-language educational materials tailored to diabetic care. These strategic measures facilitated continuity and patient adherence, targeting the specific needs of children with type 1 diabetes in a low-resource, crisis-affected setting. The structured programme design contributed directly to glycaemic control, educational reintegration, and reduction of clinical complications such as abscesses. (no specific p-value/OR/CI reported)

***TRAINING OF FORCES***
F. Healthcare workers involved in the Hargeisa programme were trained to manage diabetes in children, including the administration of insulin, interpretation of glucometer readings, and patient education. This specialised training ensured that staff could effectively respond to the unique care needs of diabetic children and contributed to improved clinical outcomes such as optimal glycaemic control in 89% of the cohort. (no specific p-value/OR/CI reported)

***PATIENT EMPOWERMENT / SELF-MONITORING***
G. In Burundi, individuals living with diabetes were provided with glucometers and trained to perform self-monitoring of their blood glucose levels. Community health workers oversaw the tracking of these results and facilitated referrals if needed. This approach empowered diabetic patients to manage their own condition while simultaneously reducing the burden on health facilities.

**Disease: Chronic Respiratory Diseases (Asthma, Chronic Obstructive Pulmonary Disease (COPD))**
**Crisis: All Crises (General Humanitarian Settings)**

***DRUG SUPPLY***
A. The WHO NCD Kit, revised in 2022, includes medicines specifically for the management of chronic respiratory diseases such as asthma and COPD. These are part of Module 1, which contains essential medications tailored to treat chronic respiratory conditions in emergency contexts. The availability of these medications within a standardized emergency package ensures that individuals with chronic respiratory diseases can continue treatment during crises when national supply chains are disrupted. (no specific p-value/OR/CI reported)

***HEALTH TECHNOLOGY AND EQUIPMENT PROVISION***
B. Module 4 of the WHO NCD Kit includes peak flow meters and inhalers with spacers, explicitly listed in the article as necessary equipment for the management of asthma and COPD. The inclusion of these items ensures the availability of diagnostic and therapeutic tools essential for managing airway obstruction, assessing respiratory function, and administering inhaled medications effectively during emergencies. These devices are critical in both acute management and long-term monitoring of chronic respiratory diseases in displaced or crisis-affected populations. (no specific p-value/OR/CI reported)

***STRATEGIC PLANNING / OPERATIONAL PREPAREDNESS***
C. The WHO NCD Kit is designed to be pre-positioned in health facilities, mobile units, and emergency deployments. This pre-positioning specifically supports the continuity of care for people living with asthma and COPD by ensuring immediate availability of respiratory medications and equipment during humanitarian emergencies. The article highlights this operational planning measure as essential for mitigating supply interruptions and enabling prompt respiratory care from the onset of crises. (no specific p-value/OR/CI reported)

***TRAINING OF FORCES***
D. From 2014–2020, the Primary Care International and UNHCR partnership included chronic respiratory diseases—specifically COPD and asthma—among the core NCDs targeted for clinical training in refugee settings. Clinical staff received evidence-based training that enabled them to diagnose and manage these respiratory diseases effectively. This training improved early case detection, reduced late-stage complications, and enhanced clinical practices concerning respiratory care. (no specific p-value/OR/CI reported)

***STRATEGIC PROGRAMME DESIGN***
E. The structured care delivery project cited in the article, conducted across multiple refugee settings, focused explicitly on COPD and asthma as among the most prevalent NCDs. The programme implemented structured clinical care elements such as patient registers, individualized follow-up systems, and condition-specific treatment protocols. These measures ensured that respiratory patients received consistent and standardized care in humanitarian settings.

**Disease: Mental Health and Neurological Disorders**
**Crisis: All Crises**

***SERVICE PROVISION***
A. In the Cox’s Bazaar refugee camps in Bangladesh, mental health support was incorporated directly into a broader community outreach programme for noncommunicable diseases. The programme included the delivery of mental health services alongside screening for hypertension and diabetes. The mental health component specifically aimed to address psychological distress among the refugee population, which is prevalent due to trauma, displacement, and chronic stress. These services were essential because people with mental health and neurological disorders in humanitarian settings face worsened health outcomes when mental healthcare is disrupted or unavailable. The article explicitly states that mental health support was part of the package delivered to increase NCD screening and treatment coverage. The combination of clinical care and psychosocial support aimed to mitigate exacerbation of mental disorders caused by unstable living conditions and untreated comorbidities.

***COMMUNITY ENGAGEMENT***
B. Awareness-raising campaigns in the Cox’s Bazaar setting included explicit mental health components, which targeted community-wide understanding and destigmatization of mental illness. These campaigns helped surface hidden mental health conditions, which are often underreported in humanitarian crises due to stigma, lack of awareness, or cultural taboos. The inclusion of mental health in the messaging enabled earlier identification of psychological distress and encouraged individuals to access the newly available services. This measure was vital in addressing unmet mental health needs among refugees and reducing delays in care-seeking behavior.

***TRAINING OF FORCES***
C. Healthcare workers operating in the Cox’s Bazaar camps received dedicated training that encompassed mental health care competencies. The article states that mental health was part of the training curriculum provided to local providers. This training enabled the identification and management of common mental health and neurological conditions within the refugee community, which often lacks access to specialists. The goal was to equip primary care-level health workers with practical skills for early identification, basic treatment, and appropriate referral of mental health cases—an essential function in settings where access to psychiatrists or neurologists is minimal or non-existent. The training also aimed to increase the confidence and capacity of frontline providers to manage complex co-morbidities where mental health intersects with other NCDs.

***STRATEGIC OR OPERATIONAL PLANNING***
D. The case study highlights that mental health services were strategically embedded within the broader NCD outreach framework implemented in the Cox’s Bazaar refugee camps. This integration reflects an operational decision to treat mental health not as a separate vertical programme but as a fundamental component of NCD care. The programme was designed with the explicit intent to include mental health in its core services, acknowledging the high prevalence of mental distress among displaced populations and the need for structured service provision. This planning facilitated continuity of care and allowed for the mobilization of resources tailored to the combined burden of psychological and physical noncommunicable conditions.

**Disease: Chronic Kidney Disease**
**Crisis: All Crises**

***DRUG SUPPLY***
A. The WHO Noncommunicable Diseases Kit (NCDK), revised in 2022, includes essential medicines and medical devices explicitly for the management of chronic kidney disease. The kit is designed to ensure continuity of care for CKD patients during emergencies where regular medical supply chains are disrupted. It provides medicines and supplies to treat 10,000 people for three months. The availability of these medicines supports kidney function management and reduces the risk of progression to end-stage renal disease under crisis conditions. The article specifically names chronic kidney disease as a target condition for the NCDK.

***SERVICE PROVISION***
B. Chronic kidney disease is explicitly listed among the additional NCDs requiring targeted service provision in humanitarian settings. The article states that CKD presents a significant and growing burden and must be integrated into essential health services. This indicates the necessity to establish specific services for CKD patients, including routine monitoring, assessment of renal function, and continued access to nephrology-related care within crisis-response frameworks. The WHO NCD Kit serves as an entry point for initiating CKD-specific services in emergency settings.

***STRATEGIC OR OPERATIONAL PLANNING***
C. The WHO NCD Kit is highlighted as a strategic preparedness resource for chronic kidney disease, to be pre-positioned before emergencies as part of disaster risk reduction and operational continuity. Its inclusion in national emergency response plans is aimed at ensuring CKD care remains uninterrupted. The article details that the NCDK has been deployed in over 20 countries as part of both preparedness and acute response, and CKD is one of the named conditions this planning targets.

**33. Article Title:** *Operational manual on noncommunicable diseases in all-hazards emergency preparedness and response*

**Author, Publication year:** Jobanputra, 2024
The article discusses all-hazards emergencies—including natural disasters, pandemics, and conflicts—as a combined category throughout, and does not stratify specific strategies or measures by disaster type. However, the manual does stratify interventions and considerations by disease type where applicable.

**List of the NCD diseases considered in this article:**
Hypertension, Cardiovascular Diseases, Diabetes Mellitus, Chronic Respiratory Diseases (e.g., Asthma, COPD), Cancer, Thyroid Conditions, Dental Conditions

**List all of the crises and disasters considered in this article (Only non–inside health system crises like natural disasters, conflict, displacement):**
Earthquakes, Hurricanes, Flooding, Drought, Armed Conflict, Displacement (Refugees and IDPs), Infectious Disease Outbreaks (e.g., COVID-19), Climate Change-Related Events

**Disease: Hypertension**
**Crisis: All-Hazards**

***DRUG SUPPLY***

A. Hypertension is prioritized among conditions requiring continuous medication access in emergencies because abrupt discontinuation can lead to hypertensive crises, strokes, and cardiovascular complications. To prevent this, antihypertensive medications such as ACE inhibitors and beta-blockers are included in the WHO NCD Kit, which is designed to support a population of 10,000 for three months during emergencies.

B. National and subnational preparedness strategies must include the regular updating and review of core antihypertensive medication lists to ensure their availability in crises. These lists are essential for emergency procurement, storage, and distribution planning.

C. The manual recommends pre-positioning of antihypertensive drugs to ensure timely distribution during crisis onset. Buffer stocks of such medications must be maintained at both national and facility levels to protect against supply chain disruptions caused by natural disasters, displacement, or armed conflict.

D. Real-time tracking and forecasting of antihypertensive medication demand is required to prevent stockouts. This involves integration into national supply chain management systems and the use of predictive tools to anticipate fluctuations in need due to population displacement or service disruption.

***SERVICE PROVISION***

A. Primary health care services must be maintained for individuals with hypertension during emergencies to avoid severe outcomes such as hypertensive encephalopathy or heart failure. This includes continuation of regular blood pressure measurement, dose adjustments, and clinical follow-up.

B. Service delivery for hypertension management must be adapted to emergency conditions using simplified treatment protocols. These should be deployable via mobile clinics, outreach teams, or adapted PHC centers, allowing for decentralized access during mobility restrictions or infrastructure damage.

C. Patients with uncontrolled hypertension or those dependent on multiple medications should be identified and prioritized through disease-specific registries. This enables tracing during evacuations or displacements and ensures that treatment interruption is minimized, thereby reducing the risk of acute decompensation.

***STRATEGIC OR OPERATIONAL PLANNING***

A. Hypertension must be integrated into national health emergency and disaster risk management plans. These plans must detail protocols for uninterrupted hypertension care, including rapid treatment of hypertensive emergencies and triaging procedures.

B. The inclusion of hypertension in Strategic Toolkits for Assessing Risks (STAR) and other health emergency planning tools is required to evaluate vulnerability and ensure that blood pressure management is not neglected during response planning phases.

***TRAINING OF FORCES***

A. Clinical and non-clinical staff in emergency response must be trained to identify and manage hypertension using protocols from the WHO PEN and HEARTS packages. This is essential because untreated hypertensive crises can rapidly result in stroke or cardiac arrest.

B. Emergency responders must also be trained in the use of the WHO NCD Kit to ensure proper handling and dispensing of antihypertensive drugs in the field. This includes understanding medication switching protocols when preferred drugs are unavailable.

***INFORMATION SYSTEMS AND SURVEILLANCE***

A. Health information systems must collect and monitor hypertension-specific indicators, such as blood pressure control rates and medication adherence. This ensures visibility of care gaps and enables timely corrective interventions in emergencies.

B. Disease registries that include patients with hypertension are critical for operational planning and patient tracing. These registries support the continuity of hypertension care by allowing emergency services to locate at-risk individuals and prioritize their care during displacement or facility shutdown.

***COMMUNITY SUPPORT AND SELF-MANAGEMENT***

A. Community health workers must be trained to support hypertensive patients with at-home monitoring, medication adherence, and recognition of symptoms requiring escalation. This is essential in crises where facility access is limited or intermittent.

B. Peer support groups, including people with lived experience of hypertension, should be mobilized to disseminate self-management techniques, promote lifestyle modifications under emergency constraints, and reduce stress-induced blood pressure spikes.

***COORDINATION AND GOVERNANCE***

A. Hypertension must be explicitly represented in multisectoral emergency coordination platforms to ensure it is not overshadowed by infectious disease priorities. Coordination should address drug supply logistics, clinical service continuity, and monitoring protocols.

B. Governance mechanisms must facilitate accelerated importation and distribution of antihypertensive drugs during crises. This includes regulatory preparedness for controlled drugs and emergency licensing of humanitarian partners providing hypertension care.

***BUDGET AND FINANCING***

A. National health insurance schemes must include provisions for hypertension care during emergencies to prevent financial barriers from interrupting treatment.

B. Emergency contingency budgets should allocate dedicated resources for procurement and distribution of antihypertensive medications, maintenance of monitoring equipment (e.g., sphygmomanometers), and training of staff specific to hypertension care.

***DIGITAL AND TECHNOLOGICAL SOLUTIONS***

A. Use of electronic health records and mobile apps should support medication tracking, telemedicine consultations, and appointment scheduling for hypertensive patients in emergencies. These platforms should be interoperable with national HIS systems and accessible in crisis settings.

**Disease: Cardiovascular Diseases**
**Crisis: All-Hazards**

***TRAINING OF FORCES***

A. The WHO PEN/HEARTS technical package must be used to train health workers in cardiovascular disease (CVD) management. The manual specifically highlights that CVDs—including ischemic heart disease and heart failure—are leading causes of mortality and require structured clinical decision-making support in emergencies. The HEARTS package includes algorithms for treatment initiation and follow-up care, enabling rapid and standardized clinical decisions in under-resourced settings.

B. Emergency personnel must be trained to properly use the WHO NCD Kit, which includes cardiovascular-specific medications and tools. These include ECG devices and medicines such as aspirin and glyceryl trinitrate. Training ensures correct triage and management of patients presenting with chest pain or acute coronary syndromes. The manual emphasizes that incorrect or delayed usage of these kits in emergencies can lead to preventable mortality in CVD patients.

C. Task-sharing is mandated to expand CVD care during crises. The manual indicates that training nurses and community health workers to manage stable cardiovascular patients using clinical protocols reduces delays in treatment initiation, especially where physician availability is limited. The rationale is that this relieves pressure on specialist services and allows timely intervention for unstable cases requiring physician-level input.

***SERVICE PROVISION***

A. Cardiovascular services must be delivered through fixed facilities, mobile teams, and outreach operations. The manual mandates that such service models incorporate management of CVD due to the high risk of exacerbation during emergencies. For instance, patients with unstable angina or heart failure may decompensate rapidly if follow-up is interrupted. These models ensure that even displaced or mobile populations receive timely cardiovascular interventions.

B. The manual requires Standard Operating Procedures (SOPs) to clearly define triage processes for cardiovascular patients. It differentiates between stable cases (e.g., well-controlled heart failure) and emergencies (e.g., new-onset chest pain), requiring immediate evaluation. This disease-specific stratification supports prioritization of scarce resources, ensuring high-risk cardiovascular patients are promptly stabilized.

C. Individuals awaiting life-saving cardiovascular procedures—such as angioplasty or surgical intervention—must be prioritized during emergencies. The manual states that in crisis conditions, delays in intervention for such patients significantly increase the risk of mortality. Facilities in unaffected areas should be equipped and staffed to accept transferred patients to prevent treatment interruptions.

***DRUG SUPPLY***

A. The manual identifies specific cardiovascular drugs included in the WHO NCD Kit, such as isosorbide dinitrate, glyceryl trinitrate, and aspirin. These medications are considered essential for managing acute ischemic heart conditions. Emergency systems must ensure that these drugs are pre-positioned and immediately accessible in all affected regions. This mitigates the risk of fatal outcomes from untreated angina or myocardial infarction during supply chain disruptions.

B. The document emphasizes that cardiovascular medications must appear on national and facility-level emergency medicine lists. This inclusion facilitates fast-tracked procurement, customs clearance, and local distribution under emergency protocols. Ensuring these drugs are not omitted is critical for sustaining treatment during disasters when global supply chains may be constrained.

***STRATEGIC OR OPERATIONAL PLANNING***

A. Cardiovascular diseases must be explicitly included in national emergency preparedness plans. These plans must outline disease-specific actions such as referral pathways for cardiac surgery, availability of diagnostics like ECG and troponin, and protocols for pharmacological management. This specificity is required because CVD patients deteriorate quickly in the absence of such structured care.

B. Pre-crisis identification of patients waiting for critical cardiovascular procedures is required. These individuals must be listed in disease-specific registries and flagged for immediate stabilization or referral in emergency scenarios. Where local infrastructure fails, protocols must be in place for medical evacuation or referral to alternative regional centers.

***DIGITAL AND TECHNOLOGICAL SOLUTIONS***

A. Electronic Health Records (EHRs) must be used to track patients with cardiovascular disease. The manual mandates the use of interoperable EHR systems to support continuity of care for displaced populations. These records must include treatment history, prior interventions, and current prescriptions to guide treatment decisions in unfamiliar or mobile clinical settings.

***INFORMATION SYSTEMS AND SURVEILLANCE***

A. Cardiovascular disease indicators must be integrated into national Health Information Systems (HIS). These indicators include hospitalization rates for cardiac events, medication adherence metrics, and access to essential services. The manual highlights that without such data, cardiovascular needs will remain invisible in emergency planning, leading to underfunding and omission in operational responses.

B. Cardiovascular registries must be maintained at health facility level. These registries allow emergency teams to identify and trace individuals with prior myocardial infarction, unstable angina, or heart failure. The manual states that prioritization based on registry data is critical for minimizing mortality during disruptions to routine care.

***COMMUNITY SUPPORT AND SELF-MANAGEMENT***

A. Community support groups must promote cardiovascular risk reduction in emergencies. The manual outlines that stress, poor diet, and medication interruption all contribute to exacerbations of heart disease during crises. Community programs must therefore focus on maintaining low-sodium diets, medication adherence, and recognition of cardiac symptoms.

B. Individuals with lived experience of cardiovascular conditions should be engaged in peer education activities. They can share management strategies and support others in adhering to treatment regimens when access to facilities is disrupted. This is especially important for those with heart failure, where fluid management and medication timing are crucial to avoid decompensation.

**Disease: Diabetes Mellitus**
**Crisis: All Crises**

***DRUG SUPPLY***

A. Insulin and other diabetes medications are listed in the WHO NCD Kit, which is formulated to cover the needs of 10,000 people over a three-month period. The inclusion of insulin in this kit directly addresses the high mortality risk associated with treatment interruptions in individuals dependent on exogenous insulin. These medications are life-sustaining, and their availability during crises prevents acute complications such as diabetic ketoacidosis, which may rapidly lead to death in the absence of insulin.

B. The manual identifies insulin-dependent individuals as particularly vulnerable due to the life-threatening nature of abrupt cessation of insulin therapy. Pre-positioning insulin stocks ensures immediate availability when health systems are disrupted. Without this, delays in re-establishing care access can result in severe metabolic decompensation.

C. Contingency planning requires drug needs assessments and resilient procurement mechanisms tailored to diabetes, as insulin has specific storage and transport requirements (e.g., cold chain logistics). This planning ensures that stockpiles are not only present but also viable under emergency conditions, securing continuity of care.

D. Medication switching protocols must be developed in anticipation of potential supply shortages or interruptions. This is especially critical for insulin, where switching from one formulation or delivery device to another without proper guidance can lead to dosage miscalculations, resulting in hypo- or hyperglycemia.

E. Language-appropriate labeling ensures safe administration of insulin in multilingual and displaced populations. Misinterpretation of instructions due to language barriers may lead to incorrect dosing, serious adverse events, or omission of therapy.

***SERVICE PROVISION***

A. The manual mandates that insulin-dependent diabetics be prioritized in emergency service delivery frameworks. These individuals face rapid health deterioration if insulin is interrupted. Identification, registration, and traceability systems must be established to prevent them from being lost in emergency transitions, particularly during displacement or facility closures.

B. Blood glucose and HbA1c testing are designated as essential diagnostics for diabetes in crisis settings. These metrics are crucial to assess glycemic control, adjust therapy, and prevent both acute complications (e.g., hypoglycemia, hyperosmolar crisis) and long-term damage during periods of instability.

C. SOPs for outpatient models in emergencies must incorporate diabetes-specific workflows. This includes mobile and outreach services equipped to manage insulin delivery and monitor patients outside traditional healthcare settings, where infrastructure may be damaged or access unsafe.

D. Disease registers must include patients with insulin-dependent diabetes to enable follow-up and treatment continuity. In emergencies, health records may be lost or inaccessible; thus, preexisting registers allow health authorities to identify and locate high-risk individuals.

E. Access to blood glucose meters and urinalysis strips is described as minimum essential diagnostic capability for crisis settings. These tools allow immediate patient evaluation and therapy adjustment, essential for preventing complications in unstable environments where laboratory access is limited.

***PREPARATION OF STRATEGIC OR OPERATIONAL PLANS***

A. National NCD action plans must integrate emergency preparedness protocols specific to insulin-dependent diabetes. These plans include the regulatory, logistical, and clinical mechanisms to maintain care delivery during disruptions, accounting for the high mortality risk in this subgroup.

B. Diabetes mellitus must be listed in national prioritized disease lists used for emergency response planning. This prioritization ensures that all emergency response actors recognize insulin therapy as a critical intervention, mobilizing resources accordingly.

***CREATION OF STORAGE RESOURCES***

A. Buffer stocks of insulin must be maintained at both national and facility levels. This requirement accounts for supply chain interruptions during crises. As insulin requires cold storage and expires rapidly without proper temperature control, such stockpiles must be rigorously managed and continuously replenished.

B. Insulin must be stored securely but accessibly to ensure rapid distribution while maintaining drug integrity and safety. This is essential in emergencies where controlled substances may be subject to theft or mismanagement, and access delays may endanger lives.

***SURVEILLANCE AND HEALTH INTELLIGENCE***

A. Facility-based surveillance must capture diabetes-specific data, including glucose control outcomes. These indicators are essential to monitor treatment effectiveness and system functionality during emergencies, enabling early detection of systemic failure in diabetes management.

B. Facility readiness assessments must measure the availability of diabetes-specific diagnostics, treatment protocols, and medications. This allows rapid identification of service gaps that may endanger insulin-dependent individuals if left unaddressed.

***COMMUNITY SUPPORT AND PROTECTION***

A. Community health workers must be trained to identify and support individuals with insulin-dependent diabetes. These patients require consistent care and rapid intervention in case of treatment interruption. Community-based identification ensures such individuals are triaged appropriately during chaotic displacement or infrastructure collapse.

B. Peer support groups composed of individuals with diabetes are described as essential for enhancing self-care during crises. These groups provide mutual aid, share resources, and offer education on glucose monitoring and insulin use in low-resource or disrupted environments.

***COORDINATION AND MULTISECTORAL COLLABORATION***

A. Coordination mechanisms must specifically account for insulin-dependent diabetes during emergency operations, including partnerships with the military and humanitarian logistics teams. These entities are often required to access remote or conflict-affected zones where insulin delivery would otherwise be impossible.

B. Collaboration with NGOs and patient alliances is vital to ensure targeted interventions for diabetes, such as mobile insulin clinics or telemedicine-based glucose monitoring, are deployed where most needed.

***FINANCING***

A. Emergency health financing must include line items for insulin procurement, cold chain logistics, and associated diabetes care costs. Without dedicated budgetary provisions, insulin-dependent individuals may face care denial due to cost constraints.

B. Health insurance and other domestic financing mechanisms must cover insulin therapy during crises to avoid catastrophic out-of-pocket spending, especially as these patients may already face economic vulnerability due to displacement or job loss.

**Disease: Chronic Respiratory Diseases (e.g., Asthma, COPD)**
**Crisis: All Crises**

***DRUG SUPPLY***

A. Severe asthma attacks are identified as life-threatening complications that require uninterrupted access to appropriate pharmacological treatment during emergencies. The manual specifies that patients with such complications must be prioritized in crisis settings to receive bronchodilators (e.g., salbutamol) and corticosteroids, as delays or interruptions may lead to respiratory arrest. Stockpiling these drugs in advance enables immediate deployment during acute phases of disaster response when health facilities may be non-functional.

B. People at risk of life-threatening complications such as severe asthma are explicitly listed among groups requiring prioritization during emergency medication planning. Their medications, such as inhaled beta-agonists and corticosteroids, are not optional but essential for survival. National emergency planning processes are required to ensure these are available in adequate quantities before and during emergencies to prevent high-mortality outcomes among these individuals.

C. The manual includes psychotropic and palliative medications as essential for patients with chronic respiratory disease, especially in advanced stages of disease or during crises when dyspnea causes intractable distress. These medications, including morphine, are considered necessary for symptom palliation. However, morphine is absent from the WHO NCD Kit, making its procurement through alternative national supply chains essential to ensure humane end-of-life care or symptom relief for patients with advanced respiratory failure.

***SERVICE PROVISION***

A. Patients with severe asthma must be included in emergency triage and service delivery frameworks. The manual presents these individuals as requiring urgent care access due to their high risk of rapid deterioration. Emergency health services must be prepared to deliver acute interventions immediately upon contact, including nebulized or inhaled medications and oxygen therapy to stabilize patients in respiratory distress.

B. For individuals dependent on oxygen, such as those with advanced COPD, the manual states that specific emergency plans are needed to ensure continuity of oxygen supply. Without access to oxygen, these individuals face imminent health deterioration. Emergency planning must, therefore, include oxygen logistics, including storage, distribution, and functional delivery systems in displaced or structurally damaged settings.

***COMMUNITY SUPPORT AND PROTECTION***

A. Community health workers and trained volunteers must be equipped to recognize signs of respiratory distress such as wheezing, tachypnea, and cyanosis, particularly in patients with asthma or COPD. The manual directs that these personnel be deployed for population-level triage to detect and refer cases of acute respiratory compromise rapidly. This is essential in settings where facility-based care is inaccessible or delayed due to environmental or security barriers.

***PREPARATION OF STRATEGIC OR OPERATIONAL PLANS***

A. Strategic planning must explicitly identify respiratory patients with potentially fatal complications such as severe asthma for both acute emergency response and continuity of care. These individuals must be registered in national preparedness frameworks and be traceable through facility- and community-based health information systems to prevent gaps in treatment continuity during health system collapse or displacement.

***SURVEILLANCE AND HEALTH INTELLIGENCE***

A. Vulnerability registers and facility-based disease registries must include individuals with severe asthma. The rationale provided in the manual is to support continuity of care and emergency responsiveness by maintaining traceability of patients whose health would be endangered by treatment interruption or delay. This ensures that health systems do not lose contact with these individuals during infrastructural breakdowns or mass population movements.

***COORDINATION AND MULTISECTORAL COLLABORATION***

A. Palliative care for patients with chronic respiratory diseases experiencing advanced symptoms such as severe dyspnea must be coordinated across all levels of emergency actors. The absence of morphine in the WHO NCD Kit is highlighted as a significant gap, underscoring the necessity of coordinated procurement and distribution of essential palliative medications by national authorities, NGOs, and humanitarian supply systems.

***FINANCING***

A. A national NCD contingency fund must be structured to include rapid procurement and distribution capabilities for essential medications required in chronic respiratory conditions, including those for acute asthma management. The manual indicates that such funds must be activated immediately during emergencies to prevent critical stockouts and ensure that essential respiratory support therapies, including medication and oxygen, are available without delay.

**Disease: Cancer**
**Crisis: All Crises**

***DRUG SUPPLY***

A. The manual explicitly states that therapeutic and supportive drugs for cancer are not included in the WHO NCD Kit. This omission requires countries and emergency responders to establish separate procurement systems for cancer care during crises. Cancer patients undergoing chemotherapy or other cytotoxic treatments rely on a continuous drug supply to prevent disease progression, recurrence, or death. Interruptions caused by lack of inclusion in standard kits necessitate alternative channels to ensure access to both curative and palliative agents during emergencies.

B. The document identifies supportive medications for cancer patients—such as allopurinol and infection prophylaxis agents—as critical elements of emergency cancer care. These drugs are essential to manage side effects of cancer therapy and prevent treatment-associated complications. Their explicit mention underlines the need for these agents to be available alongside anticancer treatments to ensure the safety and continuity of comprehensive cancer care during health system disruptions.

***SERVICE PROVISION***

A. The manual confirms that patients requiring cancer treatment must retain access to secondary-level services even during crises. This requirement stems from the clinical dependency of many cancer patients on scheduled chemotherapy, radiotherapy, or surgical interventions. Without these services, the risk of disease progression, recurrence, and mortality significantly increases. Maintaining cancer care as part of the referral continuum, despite emergency conditions, is necessary to prevent long-term negative health outcomes.

B. Cancer patients are recognized as individuals requiring both disease-modifying and palliative services. The manual explicitly notes the need to ensure that treatment is not abandoned during crises. This includes not only maintaining ongoing curative care but also offering pain relief and supportive care to patients in advanced or terminal stages. Failure to provide these services results in increased suffering, diminished quality of life, and avoidable mortality.

***CREATION OF STORAGE RESOURCES***

A. Due to the exclusion of cancer drugs from the WHO NCD Kit, the manual stresses the need to stockpile cancer medications separately. These drugs must be stored under conditions that preserve their efficacy, especially in crisis settings where cold chain and temperature control may be compromised. Secure, controlled, and accessible storage ensures timely distribution and minimizes losses due to degradation or expiration, safeguarding patient access.

***PREPARATION OF STRATEGIC OR OPERATIONAL PLANS***

A. The manual mandates that emergency preparedness plans must include specific provisions for cancer care. Cancer is identified as a high-priority condition that requires dedicated planning for logistics, clinical protocols, referral structures, and trained personnel deployment. Exclusion from strategic plans results in treatment discontinuity, increased suffering, and excess mortality. Therefore, cancer must be operationalized within emergency strategies to ensure continuity of both life-saving and life-sustaining care.

***SURVEILLANCE AND HEALTH INTELLIGENCE***

A. The manual states that individuals receiving cancer treatment must be included in facility-level patient registers and tracing systems. These systems are required to maintain visibility of cancer patients during emergency-induced displacement, facility destruction, or loss of documentation. Tracking enables health actors to resume or continue care and prevents complete interruption of therapy, which could otherwise lead to relapse or death.

***COMMUNITY SUPPORT AND PROTECTION***

A. The manual discusses the integration of palliative care for cancer patients at the community level. In emergency conditions where access to oncologists or hospital-based services is disrupted, trained community members and family caregivers must be supported to deliver basic symptom relief and psychosocial care. These services are vital for patients with advanced disease stages who are unable to access institutional care and risk dying in unmanaged pain and distress.

***COORDINATION AND MULTISECTORAL COLLABORATION***

A. Cancer treatment services must be coordinated across humanitarian actors because they fall outside the standard scope of the WHO NCD Kit. The manual calls for explicit collaboration between health ministries, NGOs, and international suppliers to ensure cancer medications and services are included in emergency responses. This coordination is essential to fill systemic gaps that otherwise leave cancer patients with no access to essential therapies or palliative options during crises.

***FINANCING***

A. The manual states that emergency health financing must allocate dedicated resources for cancer medications and care. Because cancer treatments are often high-cost and not universally covered in emergency funds, explicit budgeting is required to ensure procurement, storage, and distribution of both therapeutic and supportive cancer agents. Without financial planning, these patients face exclusion from treatment due to resource limitations in emergency contexts.

**Disease: Thyroid Conditions**
**Crisis: All Crises**

***SERVICE PROVISION***
A. The manual states that thyroid conditions, although not part of the core WHO PEN NCDs, are increasingly considered key noncommunicable diseases that should be integrated into primary care services. This means that during emergencies, service provision models must ensure the inclusion of thyroid conditions in the primary health care continuum, allowing continued diagnosis, monitoring, and treatment at the community or facility level.

***PREPARATION OF STRATEGIC OR OPERATIONAL PLANS***
A. The document includes thyroid conditions in the scope of NCDs relevant to emergency response planning. Although not categorized within the core four PEN conditions, their explicit inclusion indicates that strategic and operational plans must account for these conditions to ensure they are not neglected in preparedness, response, and recovery phases.

**Disease: Dental Conditions**
**Crisis: All Crises**

***SERVICE PROVISION***
A. The manual states that oral and dental conditions, though often overlooked in emergency health service planning, should be integrated into broader noncommunicable disease service frameworks. This inclusion is especially important during crises, as untreated dental conditions—such as infections, abscesses, or traumatic injuries—can evolve into life-threatening emergencies or cause significant morbidity. Therefore, dental care must be considered in essential service delivery packages in all-hazard emergency response plans.

***PREPARATION OF STRATEGIC OR OPERATIONAL PLANS***
A. Dental conditions are explicitly mentioned as part of the expanded scope of NCDs to be addressed in emergency preparedness plans. The manual advises that planning processes for emergency care should go beyond the core four PEN diseases to include dental conditions. This ensures appropriate referral mechanisms, resource allocation, and inclusion in operational readiness frameworks for health emergencies.

**34. Article Title:** Strengthening noncommunicable disease integration in all-hazards emergency preparedness and response: policy brief

**Author, Publication year:** Jobanputra, 2025
The article discusses all-hazard emergencies—including pandemics, natural disasters, and humanitarian crises—as a combined category throughout, and does not stratify specific strategies or measures by disaster type. But the article does stratify the impact and relevant considerations by disease type.

**List of the NCD diseases considered in this article:**
Cardiovascular Disease, Hypertension, Diabetes Mellitus, Chronic Respiratory Disease, Cancer, Renal Failure

**List all of the crises and disasters considered in this article (Only non–inside health system crises like natural disasters, conflict, displacement):**
Earthquake, Tsunami, Flooding, Storms, Extreme Temperatures, Drought, Wildfire, Infectious Disease Outbreaks, Armed Conflict, Civil Unrest, Climate Change Events, Displacement, Refugee Crisis

**Cardiovascular Disease**

**DRUG SUPPLY**

A. National treatment protocols for priority NCDs, including cardiovascular diseases, must be made available and regularly reviewed to ensure continued access to essential cardiovascular medications.
Cardiovascular diseases are among the principal conditions for which uninterrupted access to medication is critical during emergencies. The article emphasizes the importance of national treatment protocols that include cardiovascular conditions to maintain standardized care across disrupted systems. Regular updates ensure alignment with evolving epidemiological and therapeutic contexts, particularly for acute events such as myocardial infarction or hypertensive emergencies.

B. Core medication and essential technology lists should include cardiovascular medicines and be adapted at national/regional levels for use during emergencies.
Essential technology and drug lists must incorporate cardiovascular medicines such as antihypertensives, anticoagulants, and lipid-lowering agents to address both chronic management and acute interventions. The article stresses the need for these lists to be contextualized and operational at both national and regional levels to respond effectively in diverse humanitarian crises.

C. Drug supply chains must be supported through pre-positioning or rapid procurement mechanisms that include cardiovascular medications. This includes protocols for switching medications and risk mapping to establish resilient supply chains.
Pre-positioning cardiovascular medications ensures their immediate availability during the onset of emergencies, especially for patients with conditions requiring daily maintenance therapy such as hypertension or ischemic heart disease. Rapid procurement mechanisms, including pre-agreements with suppliers, are critical to prevent treatment interruption. Risk mapping identifies geographical and logistical vulnerabilities that may impact supply chain continuity, facilitating contingency planning for cardiovascular care.

D. Buffer stocks of cardiovascular medications must be maintained at national and facility levels, and supplies should be language-appropriate. This should be informed by patient registers, health information systems, or WHO STEPS data.
Maintaining buffer stocks ensures that critical medications for cardiovascular disease remain available during service disruption. Stocks should reflect anticipated demand based on patient data, and packaging should be culturally and linguistically appropriate to ensure correct usage. The article notes that information from health information systems or risk factor surveillance (e.g., WHO STEPS) should guide the volume and types of medications stored.

**SERVICE PROVISION**

E. Service delivery models should include referral pathways specifically for cardiovascular conditions that cannot be managed in primary care, and procedures for medical evacuation must be established for cases unmanageable within national boundaries.
Given the acute nature of many cardiovascular emergencies—such as myocardial infarctions—primary care alone is insufficient. The article mandates clearly defined referral pathways to ensure continuity and escalation of care. For life-threatening cases that exceed national service capacity, cross-border evacuation systems must be pre-established to ensure timely access to lifesaving interventions.

F. The WHO HEARTS technical package for cardiovascular disease should be utilized by implementing partners, including training for appropriate service provision.
The HEARTS package provides standardized interventions for prevention and management of cardiovascular diseases, including hypertension and hypercholesterolemia. The article requires this tool to be embedded in emergency service packages to support consistent, evidence-based delivery of cardiovascular care in crisis settings.

**TRAINING OF FORCES**

G. Health workers and implementing partners must be trained in the WHO HEARTS technical package for cardiovascular disease. This training should be adapted to emergency contexts and delivered through online formats if necessary.
The HEARTS package forms the foundation of technical training for cardiovascular disease management in emergencies. Training ensures that clinical staff, including those with limited cardiology experience, can follow protocols for diagnosis and management of cardiovascular events. Where in-person training is not feasible due to instability, online platforms should be deployed, ensuring continuity of workforce readiness in crisis scenarios.

**PREPARATION OF STRATEGIC OR OPERATIONAL PLANS**

H. National emergency health strategies must include referral pathways for cardiovascular disease emergencies, including acute care and treatment continuity.
The article emphasizes that acute cardiovascular events can lead to rapid deterioration and mortality without access to emergency care. Therefore, operational planning must embed cardiovascular referral networks into national strategies. These plans should clearly delineate where patients can access higher-level services and define how those pathways are activated during disruptions.

**BUDGET PROVISION**

I. Emergency funding and post-crisis resource mobilization proposals must include budget lines for cardiovascular disease treatment and services.
Cardiovascular disease services—including medications, diagnostics, and referral logistics—require specific financial allocations. The article underscores the need for these components to be explicitly costed in emergency appeals and national recovery proposals, ensuring that cardiovascular care is not deprioritized in favor of more visible or acute conditions.

**IMPROVEMENT OF INTER-SECTORAL COMMUNICATIONS**

J. Cross-border and inter-sectoral operational partnerships should account for cardiovascular disease care needs, including partnerships for medical evacuation and service continuation during emergencies.
Coordination between health ministries, humanitarian actors, and international partners must explicitly include mechanisms to continue cardiovascular care. This includes ensuring border crossing protocols for patients requiring specialist care and maintaining communication channels for timely resource mobilization.

**CREATION OF STORAGE RESOURCES**

K. Storage of cardiovascular medications must be integrated into national and facility-level contingency stockpiles, based on forecasted need and vulnerability registers.
Forecasted supply volumes for cardiovascular medications should reflect disease burden data and include high-risk populations identified through vulnerability registers. The article notes this is essential for managing patients who are at imminent risk of acute cardiovascular events if therapy is disrupted.

**SUPPORT**

L. Peer support and community health interventions may include cardiovascular disease education and support, especially in triaging individuals at high risk of life-threatening cardiovascular events.
The article advises that peer educators and community health workers be trained to assist in triaging individuals with cardiovascular disease, especially where access to professional services is limited. Such support can include blood pressure checks, medication adherence promotion, and identification of emergency symptoms requiring referral.

**Disease:** Hypertension

**DRUG SUPPLY**
A. Core medication and essential technology lists must include antihypertensive drugs and be adapted at national and regional levels.
The article identifies hypertension as a key risk factor requiring inclusion in emergency medicine lists. To ensure treatment continuity, antihypertensive drugs must be explicitly listed in national and regional essential medicines lists for humanitarian response. This measure is vital to control elevated blood pressure levels that can lead to acute cardiovascular events during crisis-induced care disruptions.

B. Antihypertensive medications must be part of the priority stockpiles, and buffer stock levels should be determined based on population-level hypertension prevalence data.
The article instructs that buffer stock calculations must account for hypertension-specific treatment needs, using data from health information systems and surveillance tools like WHO STEPS. These stocks should include both maintenance and emergency-use antihypertensives to prevent hypertensive crises resulting from treatment discontinuity.

**SERVICE PROVISION**
C. Implementation of the WHO HEARTS technical package must include specific protocols for hypertension screening, treatment, and control.
The article mandates that the HEARTS package—explicitly designed for cardiovascular risk reduction—includes standardized hypertension protocols that must be implemented in emergency service delivery. This ensures consistent and effective management of hypertensive patients across crisis-affected areas, particularly in settings lacking cardiology expertise.

**TRAINING OF FORCES**
D. Health workers and implementing partners must receive training in hypertension diagnosis, monitoring, and protocol-based management through the HEARTS package.
The article requires that healthcare providers in emergencies be trained in blood pressure measurement, risk stratification, and evidence-based pharmacological treatment for hypertension. The HEARTS package forms the technical foundation for such training, which should be adapted to crisis settings and accessible via online modules when in-person instruction is not possible.

**SUPPORT**
E. Community health workers and peer educators must be trained and equipped to conduct basic hypertension screening and triage in displaced and vulnerable populations.
The article emphasizes the role of non-specialist personnel in community-level screening for hypertension, especially in settings where health services are overwhelmed. Peer support systems can facilitate early detection and linkage to care, helping reduce preventable hypertensive emergencies.

**Disease:**Diabetes Mellitus

**DRUG SUPPLY**
A. The article emphasizes that people with insulin-dependent diabetes are at immediate risk of death if treatment is interrupted; therefore, insulin must be prioritized in emergency drug supply chains.
Interruptions in insulin access during emergencies are identified as a direct cause of mortality. The article mandates that insulin, along with glucose testing supplies, be pre-positioned or made rapidly available through emergency procurement mechanisms. Protocols for medication substitution must also account for specific insulin formulations. The inclusion of insulin in emergency kits is a non-negotiable aspect of preparedness for diabetic care continuity.

B. Medication stockpiles must account for the specific storage requirements of insulin, including cold-chain capacity and shelf-life considerations.
The article requires that insulin stocks be maintained under temperature-controlled conditions, reflecting its sensitivity to heat and light. Buffer stocks should be calculated based on diabetes prevalence and forecasted disruption duration, ensuring uninterrupted therapy. Labeling should be language-appropriate to minimize administration errors under stress conditions.

**SERVICE PROVISION**
C. Emergency service packages must ensure continued access to insulin and other diabetes-related interventions, including glucose monitoring and diabetic ketoacidosis (DKA) management.
The article explicitly notes that emergency care must cover life-threatening complications such as DKA. Basic and advanced glucose monitoring tools must be included in the service package. Health facilities should be equipped to handle acute diabetic crises and maintain regular insulin administration protocols for chronic management.

D. Specialized referral pathways must be established for diabetic emergencies, including DKA and insulin management complications.
Given the high mortality associated with acute diabetic decompensation, referral systems are required for patients who cannot be managed at primary care level. This includes transportation logistics and communication protocols with secondary and tertiary care centers capable of managing advanced diabetic complications.

**TRAINING OF FORCES**
E. Health workers must be trained in clinical guidance for insulin therapy in adults with type 1 diabetes in resource-limited or humanitarian settings.
The article includes the development of clinical guidance in collaboration with the International Alliance for Diabetes Action. This training addresses insulin dose adjustment, hypoglycemia management, and emergency protocols for type 1 diabetes. The training must be adapted to crisis conditions and provided through accessible platforms.

**PREPARATION OF STRATEGIC OR OPERATIONAL PLANS**
F. Emergency preparedness plans must include diabetes-specific protocols, including continuity of insulin access and diabetic complication management.
The article mandates the integration of diabetes care, especially insulin therapy, into emergency operational plans. This includes identifying vulnerable populations, ensuring coordination with logistics teams for insulin distribution, and incorporating diabetes metrics in emergency health service assessments.

**CREATION OF STORAGE RESOURCES**
G. Cold-chain systems must be part of contingency infrastructure to store insulin during humanitarian emergencies.
The article specifies that national and facility-level preparedness must include cold storage for insulin. Failure to maintain cold-chain results in loss of drug efficacy, endangering diabetic patients. Investment in cold storage infrastructure is presented as an essential aspect of emergency diabetes care.

**SUPPORT**
H. Peer support and community education efforts must include information on insulin storage, administration, and self-monitoring of blood glucose during crises.
The article emphasizes the importance of diabetes-specific literacy, including the ability to store and administer insulin correctly under disrupted conditions. Peer educators should be trained to support diabetic patients with self-care, recognize complications, and refer promptly to formal care channels.

**Disease:** Chronic Respiratory Disease
A. Emergency medical kits must contain medicines to treat acute complications of chronic respiratory diseases such as severe asthma.
The article explicitly states that acute respiratory complications, including severe asthma attacks, must be addressed through access to appropriate medications in emergency supply kits. These medicines must be readily available to prevent respiratory failure in individuals with chronic respiratory conditions. Emergency medications include bronchodilators and corticosteroids.

**SERVICE PROVISION**
B. Patients with severe asthma must be prioritized for life-saving care during emergencies, and where appropriate care is unavailable, supportive or palliative care must be provided.
The article identifies individuals with severe asthma as a priority subgroup requiring immediate medical attention during crises. Health systems must ensure triage and stabilization of these patients to prevent mortality from respiratory failure. If acute care cannot be administered due to contextual constraints, supportive care should be implemented to relieve suffering.

**PREPARATION OF STRATEGIC OR OPERATIONAL PLANS**
C. Preparedness programmes must identify individuals at risk of life-threatening respiratory complications, including those with severe asthma, and ensure they are included in emergency service planning.
The article requires that risk stratification during emergency planning include individuals with chronic respiratory diseases. These individuals must be mapped and integrated into operational strategies to ensure their access to services and avoid acute respiratory crises during health system disruptions.

**Disease:** Cancer

**DRUG SUPPLY**
A. The article requires the inclusion of cancer medications in national and subnational emergency risk management strategies to ensure continuity of treatment for patients undergoing active cancer therapy.
Cancer treatment regimens depend on scheduled drug administration, and any interruption can result in progression or relapse. The article mandates that cancer medicines be integrated into essential health service packages preserved during emergencies. This applies to both acute settings and protracted crises to safeguard the lives of patients requiring chemotherapy or targeted therapies.

**SERVICE PROVISION**
B. Access to specialized cancer care, including chemotherapy and palliative interventions, must be maintained throughout emergencies and incorporated into the essential health benefits package.
The article highlights that individuals undergoing active cancer treatment or requiring symptom control are at high risk of life-threatening complications if access to services is disrupted. National benefits packages must protect these services. In contexts where services are unavailable, dignified palliative care must be offered.

C. Triage and referral pathways must be established for patients with cancer to access appropriate levels of care, particularly when requiring advanced treatment such as chemotherapy or radiation therapy.
Due to the complexity of cancer care, the article emphasizes the need for clear referral systems from primary care to specialized services. These referral pathways should be maintained or re-established during crises to avoid treatment abandonment or progression of disease.

**PREPARATION OF STRATEGIC OR OPERATIONAL PLANS**
D. Emergency preparedness strategies must explicitly include cancer services in the continuum of care that should be preserved or restored.
The article stipulates that preparedness and recovery planning should integrate cancer treatment as a core component of service continuity. This involves mapping cancer care infrastructure, estimating patient needs, and ensuring resource allocation to support uninterrupted service delivery.

**SUPPORT**
E. Palliative care for patients with advanced or untreatable cancer must be ensured during emergencies, especially when curative treatment is not available.
The article explicitly identifies the need for dignified palliative care for patients with incurable cancer during crises. This includes pain management, symptom relief, and psychosocial support, particularly in situations where health systems cannot maintain full treatment protocols.

**Disease:** Renal Failure

**SERVICE PROVISION**
A. Access to dialysis must be preserved and restored as a critical component of essential health services during emergencies.
The article identifies dialysis as a life-sustaining intervention for individuals with chronic renal failure. It emphasizes that these services must not be interrupted during emergencies, as discontinuation leads to rapid clinical deterioration and death. Health service packages must prioritize the maintenance or urgent restoration of dialysis infrastructure and service delivery capacity.

B. Referral pathways for emergency care must include specific provisions for patients requiring dialysis.
Given that dialysis cannot be delayed or substituted without severe consequence, the article requires referral systems to accommodate patients in need of renal replacement therapy. Coordination with secondary and tertiary care centers capable of delivering dialysis must be ensured in both acute and protracted emergencies.

**PREPARATION OF STRATEGIC OR OPERATIONAL PLANS**
C. Emergency and contingency plans must explicitly include the continuation of dialysis as a non-negotiable health service.
The article mandates that dialysis be included in national and subnational emergency health strategies. These plans must define mechanisms to secure uninterrupted access to dialysis during health system disruptions, including coordination of transport, identification of alternative facilities, and integration into humanitarian response planning.

**BUDGET PROVISION**
D. Resource mobilization during emergencies must include budget lines for the maintenance and restoration of dialysis services.
The article underscores the high cost and logistical burden of dialysis. It states that emergency funding mechanisms and post-crisis recovery proposals must include dedicated allocations to ensure that renal failure patients continue receiving life-sustaining care.

**35. Article Title:** Systematic review on chronic non-communicable disease in disaster settings

**Author, Publication year:** Ngaruiya, 2022

**A sentence just for example:**
The article discusses conflict, displacement, earthquake, famine, and dust storm-related disasters as a combined category throughout, and does not stratify specific strategies or measures by disaster type. But the article does stratify the impact and relevant considerations by disease type.

**List of the NCD diseases considered in this article:**
Hypertension, Stroke, Myocardial Infarction or Angina, Cancer, Kidney Disease, Diabetes Mellitus, Hyperlipidemia, Chronic Obstructive Pulmonary Disease, Asthma

**List all of the crises and disasters considered in this article (Only non–inside health system crises like natural disasters, conflict, displacement):**
Earthquake, Armed Conflict (Wars), Refugee/Displacement situations, Dust Storms, Famine

**Disease: Hypertension**
**Crisis: All Crises Combined (Not Stratified by Individual Crisis Types)**

***DRUG SUPPLY***
A. In a conflict-affected region of Kosovo, only 5.5% of hypertensive patients were receiving regular antihypertensive therapy, with gender-specific differences observed: 9.09% of men and 4.24% of women were on treatment. This low rate of treatment highlights a major gap in the availability and continuity of essential medications for blood pressure control. The findings suggest that the disruption of pharmaceutical logistics and insufficient provisioning systems during crises contributed to uncontrolled hypertension, with more than half the patients presenting with severe forms of the disease.
B. In Lebanon, among Syrian refugees and vulnerable Lebanese, medication shortages were explicitly identified as a barrier to hypertension management. These shortages interfered with consistent disease control, increasing the risk of hypertensive complications.
C. In Jordan, Palestinian refugees under UNRWA services experienced lapses in drug supply chains, which disrupted routine hypertension care. Without consistent access to medication, patients were vulnerable to disease progression and cardiovascular events.

***SERVICE PROVISION***
D. In the West Bank and Gaza, refugees were significantly more likely to have hypertension than non-refugees. Women were 60% more likely than men to be hypertensive, and age was also a significant factor (p < 0.0001). This disparity indicates insufficient service provision tailored to at-risk subpopulations and suggests the necessity of enhanced clinical infrastructure to accommodate gender and age-based risk profiles.
E. After the 2008 Sichuan earthquake in China, 43.4% of 762 patients who had their blood pressure measured at Hong Kong Red Cross facilities had hypertensive readings. This reflects the immediate physiological and possibly psychological impact of disaster events and the urgent need for medical services capable of early detection and treatment of elevated blood pressure in post-disaster scenarios.
F. In Croatia, following post-war reintegration, only 50.6% of stroke patients arrived at health facilities within 6 hours. Since stroke is a known complication of uncontrolled hypertension, this delay in care delivery underscores a systemic deficiency in hypertension-related emergency response services.

***SUPPORT***
G. In Kosovo, war-induced psychosocial stress was linked to increased sympathetic nervous system activity, which triggered secondary hypertension. Specifically, 13.78% of women and 4.55% of men were affected by this stress-induced hypertension (p < 0.05). This pathophysiological link demands the inclusion of psychosocial support mechanisms for hypertension control in conflict zones.

***TRAINING OF FORCES***
H. In Eastern Slavonia, Croatia, a multiparty intervention included a single lifestyle counseling session for displaced returnees. This non-pharmacological strategy led to a statistically measurable reduction in cardiovascular risk factors, including elevated blood pressure, indicating that even brief training and deployment of trained personnel can contribute to significant clinical improvements among hypertensive individuals during recovery phases.

***CREATION OF STORAGE RESOURCES***
I. The extremely low percentage (5.5%) of hypertensive patients on regular treatment in Kosovo indicated a severe inadequacy in local drug reserves. The findings underscore the necessity of establishing emergency storage units and maintaining buffer stockpiles of essential antihypertensive medications to prevent interruption in treatment during crisis periods.

***IMPROVEMENT OF INTER-SECTORAL COMMUNICATIONS***
J. In Lebanon, healthcare providers serving Syrian refugees reported overwhelming workloads and security concerns that hampered regular hypertension care. This structural dysfunction, attributed to poor inter-sectoral coordination between humanitarian agencies and local health services, disrupted continuity of care, leaving hypertensive patients at higher risk of uncontrolled disease and its complications.

***PROVISION OF BUDGET***
K. Across Jordan and Lebanon, high transportation costs, coupled with the unaffordability of antihypertensive therapy, were major deterrents to care access. Financial barriers necessitate allocation of specific budget lines within humanitarian and governmental health planning to subsidize hypertension care, ensuring affordability and continuity of treatment.

***PREPARATION OF STRATEGIC OR OPERATIONAL PLANS***
L. A situational analysis of the Syrian national health system emphasized that the pre-conflict system's structural weaknesses—including fragmented care pathways and limited chronic disease services—were exacerbated during the conflict. It recommended integrating targeted hypertension management protocols and resource plans in any post-crisis recovery blueprint to avoid further marginalization of hypertensive populations.

***OTHER – SCREENING AND EARLY DIAGNOSIS***
M. In a multi-country UNRWA screening initiative for Palestinian refugees, individuals were most commonly referred for hypertension screening due to age and family history. Women over 40 were screened significantly more frequently than men (p < 0.01), illustrating the importance of demographic-specific screening strategies in crisis settings.
N. In Croatia, displaced persons residing in camps exhibited a significantly higher prevalence of hypertension compared to age-matched controls from unaffected areas (p < 0.05). This demonstrates the elevated baseline risk within displaced populations and supports the implementation of systematic blood pressure screening in displacement and refugee camps.

**Disease: Stroke, Myocardial Infarction or Angina (Combined)**
**Crisis: All Crises Combined (Not Stratified by Individual Crisis Types)**

***DRUG SUPPLY***
A. In Kosovo, the article reported that among patients presenting with cardiovascular conditions, including angina, only 5.5% were receiving regular antihypertensive treatment, and 62% of patients had comorbid diseases, including cardiovascular disorders. The scarcity of consistent drug supply meant that medications for managing angina and preventing myocardial infarction were largely inaccessible, leading to poor disease control. This inadequacy contributed to the high prevalence of severe disease forms. The deficiency in therapeutic continuity during crisis directly elevated the cardiovascular risk burden.
B. Among Iraqi refugees in Jordan, angina pectoris was one of the diagnoses that required the highest number of clinical visits per patient (average 1.35 visits per patient). The repeated encounters reflect ongoing clinical instability and highlight inadequacies in the availability or effectiveness of pharmacologic treatment for ischemic heart conditions in crisis settings. Poor medication supply hindered symptom control and necessitated frequent health system interaction.

***SERVICE PROVISION***
C. In Baranya, Croatia, during the post-war period, 513 patients presented with stroke at a single emergency department. Of these patients, 85.8% had hypertension, 27% had diabetes mellitus, and 44.6% had hyperlipidemia. However, only 50.6% of patients reached the facility within 6 hours of symptom onset, while 16.2% presented more than 24 hours later. These delays in care compromised acute stroke management and reflected deficiencies in health service capacity and emergency responsiveness during crisis recovery phases.
D. In Bosnia and Herzegovina during wartime, a retrospective review demonstrated a statistically significant increase in acute myocardial infarction cases in the total population during war years compared to pre-war (p = 0.025). Likewise, unstable angina increased significantly during the war (185 cases compared to 125 prewar, p = 0.001). These findings indicate that cardiovascular disease burden increases during armed conflict due to heightened physical and psychological stress, and health systems must expand emergency cardiac services during such periods to handle the elevated case load.
E. The same Bosnian study revealed that the increases in myocardial infarction and unstable angina were statistically significant for females (p = 0.001 for unstable angina and p = 0.007 for AMI), while changes in male patients were not statistically significant. This indicates a disproportionate increase in cardiovascular events among women during crisis, likely linked to gender-specific stress exposures or care access limitations. Emergency services must therefore adopt gender-sensitive designs to effectively address cardiovascular disease in female populations during conflict.

***TRAINING OF FORCES***
F. In Eastern Slavonia, Croatia, a multifaceted intervention involving a single counseling session aimed at lifestyle change was delivered to returnees post-conflict. The session addressed risk factors for cardiovascular diseases such as myocardial infarction and stroke. The intervention contributed to reductions in these risk factors among the participants, which highlights the utility of training frontline personnel or health workers in delivering focused, low-resource interventions that can modify behavior and reduce disease incidence even in unstable settings.

***SUPPORT***
G. During the Croatian War of Independence, coronary care unit data showed that 1,257 patients were hospitalized for myocardial infarction during wartime, compared to 1,024 pre-war and 1,173 post-war. There was a shift toward younger patients (<45 years) who also had significantly higher smoking rates (75%) compared to older patients (51%), with a p-value of < 0.001. This increase in AMI among younger individuals correlated with elevated stress levels and behavioral risk factors exacerbated by conflict conditions. These patterns highlight the need for psychosocial and behavioral support interventions to mitigate preventable contributors to myocardial infarction during crises.

***OTHER – MONITORING AND SURVEILLANCE***
H. In Armenia, following the 1988 earthquake, a nested case-control study involving 483 cases of newly reported heart disease and 482 matched controls revealed that increased material and familial loss had a dose-response relationship with myocardial infarction risk. Specifically, the odds ratios for heart disease with increasing loss scores were 1.3, 1.8, and 2.6, respectively. This demonstrates that systematic tracking of trauma-related loss and exposure among disaster survivors is critical for identifying individuals at elevated risk for ischemic heart events and implementing appropriate clinical monitoring or preventive care.

**Disease: Cancer**
**Crisis: All Crises Combined**

***SERVICE PROVISION***
A. In Jordan, among Iraqi refugees receiving UNHCR health assistance, senile cataract, cerebrovascular disease, glaucoma, urolithiasis, prostatic hyperplasia, and angina pectoris were reported as diagnoses requiring the highest number of visits per patient. Cancer diagnoses were included among the conditions necessitating frequent visits, which reflects a significant service burden. The persistent need for multiple clinical visits by cancer patients indicated challenges in the continuity and comprehensiveness of cancer care services in crisis-affected refugee populations.
B. In Iran, among Afghan refugee populations, neoplasms constituted the most common reason for medical referrals within the Pashtun ethnic group. This highlights the ongoing need for structured cancer care referral pathways in humanitarian response systems. The disparity in referral patterns among different ethnic groups further indicates a lack of standardized cancer care access.

***DRUG SUPPLY***
C. In Lebanon, under the Lebanese Aging Study conducted in 1983–1993 during prolonged national instability, cancer was found to account for 15% of deaths among individuals aged 50 years and above. The article attributed the mortality partly to limited access to treatment and therapeutic resources, suggesting that drug supply constraints contributed to the outcome burden of cancer in prolonged crisis settings.

***SUPPORT***
D. In Lebanon, long-term instability and the lack of comprehensive health system support were associated with limited access to diagnostic and treatment services for cancer. The elevated cancer mortality rate (15%) during the study period supports the need for structural support interventions that extend beyond immediate care, including follow-up and palliative support frameworks, particularly for older populations.

***OTHER – DIAGNOSIS AND REFERRAL***
E. In Iran, data from Afghan refugee populations revealed that cancer was a leading cause of referral in the Pashtun group, while other groups had different predominant causes. This indicates that cancer diagnostic needs vary significantly across ethnic subgroups within refugee populations, necessitating tailored diagnostic and referral strategies to detect and manage cancer effectively in these contexts.

**Disease: Kidney Disease**
**Crisis: All Crises Combined**

***SERVICE PROVISION***
A. In Iran, among Afghan refugee populations receiving healthcare between 2005 and 2010, nephropathies were reported as the most frequent cause of medical referrals in the Uzbek ethnic group. This indicates that kidney-related diseases represented a substantial burden requiring structured service delivery systems tailored to the population’s health needs. The ethnic-specific pattern underscores the necessity of adaptable healthcare models that prioritize access to nephrology services in crisis contexts.

***OTHER – DIAGNOSIS AND REFERRAL***
B. The same study in Iran showed that different ethnic groups within the Afghan refugee population exhibited distinct disease patterns. While nephropathies were most common in the Uzbek group, they were not the primary cause of referrals in others. This differentiation supports the need for targeted screening and diagnostic strategies specific to kidney disease among subgroups in displaced populations to ensure timely referral and treatment.

**Disease: Diabetes Mellitus**
**Crisis: All Crises Combined**

***DRUG SUPPLY***
A. In Lebanon, under a humanitarian program treating Syrian refugees and vulnerable Lebanese, diabetes mellitus accounted for 27% of the diagnosed cases, and an additional 27% of patients had both hypertension and diabetes. Medication shortages were explicitly cited as a significant challenge in managing diabetic patients, impeding continuity of treatment and glycemic control. Lack of insulin and oral hypoglycemics was reported to contribute to worsening health outcomes among affected populations.

***SERVICE PROVISION***
B. In Jordan, among Palestinian refugees receiving care through UNRWA services, 18,881 patients were included in a retrospective cohort analysis. Of these, 50% were diagnosed with both hypertension and diabetes, while the other 50% had hypertension alone. The analysis found a significantly higher rate of disease-related complications in those with both conditions (13%) compared to those with hypertension alone (6%), with an odds ratio of 2.2 (95% CI 2.0–2.5). This reflects a substantial demand for integrated service provision capable of managing multimorbidity in diabetic patients.
C. In Lebanon, healthcare workers reported high workloads and excessive clinical burdens due to a large number of diabetic and hypertensive patients requiring frequent follow-up. This strain on services disrupted proper monitoring of blood glucose levels and medication titration, indicating the need for scaled-up service infrastructure to manage diabetic populations in crisis settings.

***SUPPORT***
D. In Jordan, risk factor analysis in Palestinian refugee populations showed that smoking, physical inactivity, and obesity were the most common contributing factors to diabetes mellitus. The co-occurrence of these modifiable risks suggests the need for structured patient support mechanisms including lifestyle counseling and behavioral modification services that address the root causes of diabetic exacerbation in crisis environments.

***PROVISION OF BUDGET***
E. In the Lebanese and Jordanian refugee care programs, cost-related barriers were highlighted as major obstacles to diabetes care. Patients frequently lacked funds for transportation to health centers and could not afford long-term medications for diabetes management. These financial challenges severely restricted adherence and follow-up, underlining the urgent necessity for dedicated budget allocations aimed at subsidizing diabetes-related care, transport, and monitoring supplies.

***OTHER – COMPLICATION MONITORING AND OUTCOME TRACKING***
F. In the UNRWA-managed program in Jordan, patients with comorbid diabetes and hypertension were significantly more likely to develop complications compared to those with hypertension alone (13% vs. 6%, OR = 2.2, 95% CI 2.0–2.5). This outcome highlights the requirement for proactive monitoring and outcome tracking systems to identify at-risk diabetic patients early and implement preventive interventions.

**Disease: Hyperlipidemia**
**Crisis: All Crises Combined**

***SERVICE PROVISION***
A. In post-conflict Croatia, emergency department records from Baranya documented 513 stroke patients between November 1997 and December 2001. Among these, 44.6% had documented hyperlipidemia as a comorbid condition. This substantial percentage of hyperlipidemic individuals among stroke patients highlights the need for integrated service provision that includes lipid profile screening and management for cardiovascular risk reduction during and after crisis periods.

***SUPPORT***
B. In Eastern Slavonia, Croatia, a lifestyle-focused intervention was implemented among displaced returnees following conflict. The intervention consisted of a single session of health education and counseling on modifiable risk factors, including hyperlipidemia. Patients who received this brief counseling session showed improvements in cardiovascular risk profiles, suggesting that structured support focused on behavioral change is effective in managing lipid levels among crisis-affected populations.

**Disease: Chronic Obstructive Pulmonary Disease and Asthma**
**Crisis: All Crises**

***DRUG SUPPLY***
A. Limited access to essential respiratory medications such as inhalers and bronchodilators was explicitly reported as a challenge for patients with COPD and asthma in disaster settings. The disruption of supply chains during crises directly prevents individuals from managing acute exacerbations, leading to severe health deterioration. This problem was particularly significant in displaced populations where chronic disease management requires ongoing pharmacological intervention. The absence of these critical drugs increases hospitalization rates due to poorly controlled symptoms.
B. The lack of infrastructure for medication distribution during disasters made it difficult for health facilities to procure and deliver necessary respiratory treatments. This led to interruptions in the medication regimens of patients with asthma and COPD, especially among refugees and internally displaced persons, exacerbating morbidity in these groups.

***SERVICE PROVISION***
C. During dust storm episodes in Sanandaj, Iran, a statistically significant increase in emergency admissions for respiratory diseases including COPD and asthma was reported. This was attributed to acute environmental exposure coupled with insufficient medical response infrastructure. The correlation coefficient was r = 0.48 with a p-value < 0.05, indicating a meaningful relationship between dust storm exposure and the acute need for respiratory care. This highlights that during crises, individuals with chronic respiratory diseases are particularly vulnerable to external environmental stressors in the absence of robust service delivery mechanisms.
D. Refugee populations with asthma and COPD encountered severe barriers to consistent clinical follow-up and emergency care. Due to displacement and insecurity, they often lacked access to health facilities capable of managing respiratory diseases, leading to worsening conditions. The health system’s inability to accommodate these populations with targeted respiratory services intensified the disease burden.

***ENVIRONMENTAL CONTROL***
E. Exposure to dust and airborne particulates during crises—especially dust storms—directly exacerbates the symptoms of COPD and asthma. In Sanandaj, Iran, the article documents a statistically significant surge in emergency department visits for these diseases during dust storm events. The airborne particles provoke airway inflammation and bronchoconstriction, particularly in individuals with pre-existing pulmonary conditions. The physiological stress induced by these environmental exposures worsens baseline respiratory status and often necessitates urgent medical intervention.
F. The absence of protocols to mitigate air pollution during such events reflects a systemic failure to protect vulnerable populations. Without controlled indoor air environments or public advisories, individuals with asthma and COPD are involuntarily exposed to triggers that severely worsen their conditions. This environmental mismanagement during crises amplifies disease severity and contributes to avoidable morbidity.

***PREPAREDNESS AND STRATEGIC PLANNING***
G. The article highlights that disaster preparedness frameworks often fail to incorporate the unique needs of individuals with COPD and asthma. This omission includes the lack of pre-positioned respiratory medications and contingency plans for managing airway diseases during emergencies. The absence of respiratory-specific protocols in disaster plans indicates institutional neglect of a population highly susceptible to crisis-induced health deterioration.
H. Most preparedness plans reviewed did not include structured screening or triage strategies for chronic respiratory conditions. The failure to pre-identify high-risk individuals with asthma and COPD prevents timely intervention during the acute phases of a disaster, leading to unchecked exacerbations and higher mortality.

***RESOURCE ALLOCATION AND BUDGET***
I. Funding and resource allocation patterns in humanitarian crises deprioritized respiratory diseases such as asthma and COPD. As a result, health systems lacked the capacity to purchase necessary devices like nebulizers and oxygen tanks, which are essential for managing acute respiratory episodes. This financial oversight directly impacts survival rates during emergencies for patients dependent on these interventions.
J. The article further notes that in contexts of displacement, health programs were frequently underfunded to the extent that even basic respiratory care—including spirometry and medication stockpiles—was unattainable. The financial marginalization of asthma and COPD management renders the affected populations more vulnerable to crisis-induced mortality.

**36. Article Title:** *Integrating NCD Care in Humanitarian Settings – An Operational Guide*

**Author, Publication year:** UNHCR, 2020

**Combined Disaster Scenario Description:**
The article discusses natural disasters, conflict, and displacement as a combined category throughout, and does not stratify specific strategies or measures by disaster type. The article also does **not** stratify the impact and relevant considerations by disease type.

**List of the NCD diseases considered in this article:**
Hypertension, Cardiovascular Disease, Diabetes Mellitus, Chronic Respiratory Disease, Cancer, Epilepsy, Mental Health Conditions

**List of the crises and disasters considered in this article (Only non–inside health system crises):**
Conflict, Displacement, Humanitarian Emergencies

**Disease: All NCDs**

**Crisis: All Crises**

***STRATEGIC AND OPERATIONAL PLANNING***
A. The guide requires the integration of NCD services into humanitarian response frameworks such as the Humanitarian Needs Overview (HNO), Humanitarian Response Plan (HRP), and the Minimum Initial Service Package (MISP). This is necessary because, in humanitarian contexts, patients with NCDs such as hypertension, diabetes, cardiovascular disease, or chronic respiratory illness experience treatment interruptions, which worsen disease outcomes. By explicitly embedding NCD care into strategic documents, the health system recognizes chronic disease management as essential and ensures that plans are made to continue service delivery during emergencies. This inclusion creates accountability, justifies funding, and mandates the presence of trained personnel and appropriate medications in humanitarian responses.
B. The inclusion of NCDs in contingency planning ensures early identification of vulnerabilities in populations with high NCD burdens and enables timely mobilization of resources. The article explains that planning must include clearly defined responsibilities, timelines, and targets for NCD service implementation, which is critical given that many NCD complications (e.g., myocardial infarctions, diabetic ketoacidosis, hypertensive emergencies) can be fatal if not addressed promptly during crises.

***DRUG SUPPLY AND ESSENTIAL COMMODITIES***
A. The guide stipulates pre-positioning of essential NCD medicines and supplies because individuals with NCDs rely on consistent access to medications to prevent acute complications. For example, insulin interruptions in diabetic patients can lead to hyperglycemia and death within days, while gaps in antihypertensive medications increase the risk of stroke and myocardial infarction. Thus, medicine stockpiling in vulnerable areas directly prevents avoidable morbidity and mortality.
B. It emphasizes adapting procurement and supply chains to accommodate chronic treatment needs by including NCD medicines in emergency health kits and ensuring periodic restocking. Simplified and standardized treatment protocols are advised to match the available medicines and equipment to the levels of care in the crisis setting. This harmonization allows health workers to respond effectively without requiring specialized diagnostics or therapies that may not be accessible during emergencies.

***SERVICE PROVISION***
A. The guide calls for integrating NCD care into primary healthcare services. This is essential because decentralized service provision increases accessibility for patients with reduced mobility or disrupted transportation due to crises. The article explains that stable NCD patients can be managed at the primary level through routine follow-ups and medication refills, thereby reducing unnecessary referrals and overburdening of secondary and tertiary care systems.
B. Task-sharing and task-shifting models are promoted to allow non-specialist health workers to manage NCDs using standardized protocols. This measure increases treatment continuity when specialist availability is reduced in emergencies. For instance, nurses and clinical officers can manage hypertension and diabetes in line with simplified clinical guidelines, improving timely interventions.
C. Referral systems for complex cases are essential because conditions such as cancer or end-stage organ failure may require specialized interventions. By establishing clear referral pathways, the guide ensures that these patients are not lost in the system during crises, and continuity of advanced care is preserved for life-threatening NCD complications.

***WORKFORCE TRAINING AND CAPACITY BUILDING***
A. The article stresses that training frontline healthcare providers on standardized NCD protocols is vital to ensure quality care under crisis constraints. For example, in the absence of lab diagnostics, health workers must be skilled in syndromic management of conditions like diabetes or asthma using available tools. This builds system resilience and promotes early intervention in patients with warning signs of disease exacerbation.
B. The article further notes that emergency training programs must include NCD components, as acute presentations like stroke, diabetic coma, or hypertensive crisis are common among displaced populations. These training efforts also ensure that staff can appropriately triage and stabilize NCD patients, especially in settings with high staff turnover or deployment of non-NCD specialists.

***HEALTH INFORMATION SYSTEMS AND DATA***
A. The guide emphasizes including NCD indicators in routine health information systems to inform service planning. Capturing data on disease prevalence, medicine consumption, and treatment outcomes allows identification of service gaps and rapid allocation of resources. For example, an increase in uncontrolled diabetes rates in a camp signals the need to re-evaluate insulin availability, cold chain maintenance, or provider knowledge.
B. Rapid assessments in emergencies should disaggregate data by condition, age, and sex to identify high-risk groups. For example, older adults with cardiovascular conditions may be more vulnerable to disruptions, requiring targeted outreach. Without such data, NCD patients may be overlooked in the planning process, leading to avoidable deterioration of health outcomes.

***FINANCING AND BUDGET ALLOCATION***
A. The guide requires explicit allocation of resources for NCD services in humanitarian health budgets. This is necessary because NCDs impose a continuous financial burden through medication costs, follow-up appointments, and diagnostic requirements. In crises, when public infrastructure and income collapse, the absence of budgeted NCD support results in treatment interruptions. Funding allocations must therefore reflect the recurring nature of chronic disease care, including procurement, staff training, and patient education.

***INTERSECTORAL COORDINATION AND PARTNERSHIPS***
A. The article calls for coordination between humanitarian organizations, ministries of health, and development partners to ensure harmonized NCD responses. For example, aligning treatment protocols across sectors prevents conflicting practices and ensures continuity of care for displaced populations crossing regions.
B. Local NGOs and civil society organizations are instrumental in raising awareness about NCD symptoms and promoting community-based prevention strategies. Involving these partners also allows for quicker identification of service gaps and mobilization of community health workers in managing NCDs at household levels.

***COMMUNITY ENGAGEMENT AND SUPPORT***
A. The article notes that involving communities in designing NCD services ensures culturally appropriate messaging and better adherence. For example, in communities with high salt intake, health education efforts targeting dietary modifications can directly reduce hypertension-related complications.
B. Peer support networks and group education sessions improve disease literacy and self-care, especially in low-resource settings. For instance, patients who understand their disease triggers are more likely to seek timely help and adhere to treatment regimens, thereby reducing emergency exacerbations during crises.

***MONITORING AND EVALUATION***
A. Monitoring systems must be used to assess service delivery coverage, quality, and effectiveness for NCDs. These metrics guide improvement efforts and ensure that patients are not systematically missed.
B. Evaluation protocols must include both process (e.g., number of patients followed) and outcome indicators (e.g., blood pressure or glucose control rates). Without robust evaluation, there is no mechanism to identify system weaknesses or successes in NCD crisis care delivery.

***INFRASTRUCTURE AND EQUIPMENT***
A. Health facilities must be equipped with basic diagnostic and monitoring tools for NCDs, such as blood pressure monitors and glucometers. The absence of these tools prevents early detection of acute changes, leading to delayed care and higher mortality. For instance, an undiagnosed hypertensive crisis can result in a stroke within hours, making basic equipment essential.

***CONTINUITY OF CARE AND PATIENT FOLLOW-UP***
A. The guide advises the use of portable, patient-held records and harmonized treatment protocols to ensure uninterrupted care for displaced individuals with chronic diseases. Patients with NCDs often move between locations, and standardized documentation ensures that essential health information is preserved and used appropriately across different providers.
B. Case tracking systems must be developed to maintain follow-up for patients requiring continuous care. For example, a diabetic patient requiring insulin must be monitored regularly to adjust dosages and screen for complications; failure to maintain this follow-up leads to severe outcomes.

**37. Article Title:** *Noncommunicable diseases in emergencies*

**Author, Publication year:** WHO, 2016
The article discusses natural disasters, conflict, displacement, and other humanitarian emergencies as a combined category throughout, and does not stratify specific strategies or measures by disaster type. The article also does not stratify the impact or relevant considerations by disease type, instead addressing NCDs broadly or by acute management priority.

**List of the NCD diseases considered in this article:**
Hypertension, Diabetes Mellitus (Type 1 and Type 2), Heart Failure, Myocardial Infarction, Stroke, Cancer, Chronic Respiratory Disease (including Asthma and COPD), Epilepsy, Chronic Kidney Disease (Dialysis-dependent), Hyperlipidemia

**List all of the crises and disasters considered in this article (only non–inside health system crises):**
Earthquakes, Cyclones, Forest Fires, Floods, Heatwaves, Droughts, Epidemic and Pandemic Diseases, Transport Crashes, Building Fires, Chemical Hazards, Radiological Hazards, Food Insecurity, Armed Conflicts, Mass Gatherings, Forced Displacement, Humanitarian Emergencies

**ALL NCDs**

**ALL CRISES**

***DRUG SUPPLY AND ESSENTIAL MEDICATION ACCESS***
A. Ensure access to essential NCD medication, prioritizing the WHO Package of Essential Noncommunicable (PEN) disease interventions or the national essential medicines list.
When emergencies occur, access to critical NCD medications is often disrupted due to destroyed infrastructure, displacement, and breakdowns in supply chains. The article emphasizes that continued provision of medications is vital because interruption of treatment can lead to life-threatening exacerbations, especially in conditions such as diabetes mellitus, hypertension, and chronic heart disease. For instance, lack of insulin for type 1 diabetes or antihypertensives for severe hypertension can precipitate acute metabolic crises or hypertensive emergencies respectively. The WHO PEN package is designed to ensure availability of the most commonly required medications in such emergencies. These include insulin, metformin, ACE inhibitors, beta-blockers, and statins. The article recommends early review and adjustment of national essential medicines lists in affected countries to ensure alignment with emergency needs.

B. Avoid sudden discontinuation of treatment for NCDs, especially for patients requiring continuous medication such as insulin-dependent diabetics, transplant patients, and individuals on anticoagulation.
The article explains that sudden cessation of therapy, particularly in individuals with NCDs that are dependent on continuous treatment, can cause rapid deterioration. For example, transplant recipients are at risk of organ rejection, and anticoagulated patients with mechanical heart valves risk thromboembolic events if therapy is stopped abruptly. Insulin-dependent patients can enter diabetic ketoacidosis in the absence of insulin. These acute crises increase mortality and health system burden during emergencies. Therefore, medication continuity must be prioritized in disaster preparedness and response frameworks.

***SERVICE PROVISION***
A. Provide basic care at primary health-care level for physical symptoms of advanced NCDs, including pain management and respiratory distress relief.
Advanced presentations of NCDs such as end-stage cancer, chronic obstructive pulmonary disease (COPD), or congestive heart failure frequently involve distressing symptoms that worsen in crises. The article identifies dyspnea and pain as critical symptoms that must be managed even when definitive care is not feasible. Primary health-care services must be equipped to provide palliative interventions, including opioids, bronchodilators, and other supportive measures. This is crucial in humanitarian settings where hospital care is inaccessible.

B. Ensure clinical management or stabilization and appropriate referral for patients with acute exacerbations that cannot be managed at the primary care level.
Emergencies can precipitate acute cardiac events such as myocardial infarction and stroke, which require urgent intervention. The article notes that events such as heart attacks and strokes may be two to three times more common during emergencies than under normal conditions. This spike is attributed to stress, disruption of medication, and underlying vulnerabilities. Therefore, primary facilities must be prepared to stabilize such cases and refer them using predefined referral pathways to secondary and tertiary care centers, if accessible.

C. Ensure access to essential diagnostic equipment, core laboratory tests, and routine management resources for NCDs within primary health-care settings during protracted emergencies or recovery phases.
In long-term emergency settings or recovery phases, the article underlines the need to restore or establish routine diagnostic and monitoring services. Equipment such as glucometers, blood pressure monitors, and laboratory assays for serum creatinine and lipid profiles are essential for safe ongoing care of diabetes, hypertension, renal disease, and cardiovascular risk. Restoration of these services ensures continuity of care and detection of deteriorating conditions that may require escalation of treatment.

D. Maintain access to pre-emergency treatments as the mainstay of the health-sector response, including curative, preventive, and palliative services.
The article emphasizes that maintaining patients’ access to treatments they were receiving before the emergency is the most cost-effective and lifesaving strategy. This includes continuing medications, follow-up care, and symptom control. Disruption to these services leads to avoidable complications and mortality. For example, patients stable on antihypertensives or glucose-lowering drugs before the emergency may experience acute decompensation without these medications.

E. Identify patients with special needs for whom interruption of treatment could be fatal or critical (e.g., dialysis-dependent patients, type 1 diabetics), and ensure ongoing care.
Certain NCD patients depend on continuous, non-substitutable care (e.g., dialysis, insulin injections). The article notes that missing even a few sessions or doses can result in rapid clinical deterioration. For instance, dialysis patients risk fluid overload and electrolyte imbalance, leading to death. Type 1 diabetics risk severe ketoacidosis. Identifying and tracking these individuals allows for prioritization of scarce resources and tailored support plans.

***STRATEGIC AND OPERATIONAL PLANNING***
A. Incorporate the needs of people with NCDs into national disaster preparedness and emergency response plans, including the protection of health facilities and pre-positioning of critical supplies.
The article argues that most existing disaster plans inadequately consider the ongoing needs of NCD patients. To prevent treatment interruption, health systems must plan for scenarios that secure access to essential infrastructure (e.g., electricity for dialysis, refrigeration for insulin) and medicines. Facility protection strategies include reinforcement of buildings, planning for generator use, and ensuring functional supply lines.

B. Establish standard operating procedures (SOPs) for identifying patients in need of palliative care and symptom relief, and for referral to secondary and tertiary care facilities.
The article advises that all primary health-care facilities should have SOPs for triaging and managing patients with severe chronic NCDs. These SOPs should clearly define referral criteria and processes to access higher-level care or palliative services. This ensures that life-threatening symptoms are not overlooked and that suffering is minimized, especially when definitive treatment is not possible.

***STORAGE AND RESOURCE PREPOSITIONING***
A. Protect key health facilities from natural disasters and ensure stockpiling of key medicines and equipment required for NCD care, including dialysis and insulin.
According to the article, many patients with NCDs rely on time-sensitive medications and treatments. Insulin must be kept cold, dialysis requires complex machinery and water supplies, and pain medications must be available on demand. Prepositioning of such supplies in disaster-prone areas, along with facility-specific protection plans, can ensure service continuity even during severe disruptions.

***PATIENT-LEVEL EMERGENCY PLANNING***
A. Develop individual patient strategies for emergencies, including backup supply of medications and instructions for emergency care.
The article highlights the need for personalized emergency planning for individuals with chronic diseases. This includes ensuring patients have a 2–4 week backup of their essential medications and written instructions on what to do in case they are displaced or unable to reach their usual health provider. Such planning is critical for maintaining disease control when health services are overwhelmed.

B. Establish registries of NCD patients with critical needs, such as those on home ventilators, dialysis, or insulin-dependent.
The creation of NCD-specific registries helps responders rapidly identify and locate the most vulnerable individuals who need immediate and uninterrupted care. These registries can support targeted evacuation, prioritization for medication, or special referral pathways. The article states that this level of preparation can significantly reduce preventable deaths during disasters.

***INTER-SECTORAL COORDINATION AND SUPPORT PARTNERSHIPS***
A. Link emergency response systems with organizations that can support NCD care in crises, such as the Renal Disaster Relief Task Force of the International Society of Nephrology, the International Diabetes Federation, and the Insulin for Life charity.
The article recommends leveraging external partnerships to supplement national efforts. Specialized NGOs and international agencies can provide essential medications, technical expertise, and logistical support for NCD patients. For example, the Renal Disaster Relief Task Force provides dialysis support during emergencies, while the Insulin for Life network distributes insulin and diabetes supplies to areas in crisis.

***MONITORING AND EVALUATION (INDICATORS)***
A. Measure the percentage of primary health-care facilities with adequate medication to continue pre-emergency treatment, including pain relief, for individuals with NCDs.
This indicator provides a metric to evaluate the success of continuity strategies and highlights supply chain gaps in real time. The article notes this as critical for assessing quality of care during emergencies.

B. Monitor the number and percentage of functional health facilities providing NCD care and the number of basic health units capable of delivering such care per 10,000 population.
These indicators help ensure that sufficient service capacity exists within affected areas. They also inform resource reallocation and scaling decisions as emergencies evolve.

**Ref**

1.	Kishimoto M, Noda M. Diabetes care: After the Great East Japan Earthquake. J Diabetes Investig. 2013;4(1):97–102.

2.	Ruby A, Knight A, Perel P, Blanchet K, Roberts B. The Effectiveness of Interventions for Non-Communicable Diseases in Humanitarian Crises: A Systematic Review. PLoS One. 2015;10(9):e0138303.

3.	Ryan B, Franklin RC, Burkle FM, Jr., Aitken P, Smith E, Watt K, et al. Identifying and Describing the Impact of Cyclone, Storm and Flood Related Disasters on Treatment Management, Care and Exacerbations of Non-communicable Diseases and the Implications for Public Health. PLoS Curr. 2015;7(DISASTERS).

4.	Organization WH. Noncommunicable diseases in emergencies. World Health Organization; 2016.

5.	Tonelli M, Wiebe N, Nadler B, Darzi A, Rasheed S. Modifying the Interagency Emergency Health Kit to include treatment for non-communicable diseases in natural disasters and complex emergencies. BMJ Glob Health. 2016;1(3):e000128.

6.	Nakaya N, Nakamura T, Tsuchiya N, Narita A, Tsuji I, Hozawa A, et al. Psychological Distress and the Risk of Withdrawing From Hypertension Treatment After an Earthquake Disaster. Disaster Med Public Health Prep. 2017;11(2):179–82.

7.	ICRC. Managing Projects Addressing Non-Communicable Diseases: Operational Guidelines for Field Staff. 2018.

8.	IFRC. INSPIRATION AND ASPIRATION: Advocating for NCD care in humanitarian settings – Outcomes of a Bootcamp. 2018.

9.	IFRC. NCD care in humanitarian settings: a clarion call to civil society. 2018.

10.	Man RX, Lack DA, Wyatt CE, Murray V. The effect of natural disasters on cancer care: a systematic review. Lancet Oncol. 2018;19(9):e482–e99.

11.	Boulle P, Kehlenbrink S, Smith J, Beran D, Jobanputra K. Challenges associated with providing diabetes care in humanitarian settings. Lancet Diabetes Endocrinol. 2019;7(8):648–56.

12.	Kehlenbrink S, Smith J, Ansbro E, Fuhr DC, Cheung A, Ratnayake R, et al. The burden of diabetes and use of diabetes care in humanitarian crises in low-income and middle-income countries. Lancet Diabetes Endocrinol. 2019;7(8):638–47.

13.	UNHCR. Integrating NCD Care in Humanitarian Settings – An Operational Guide. 2020.

14.	Gyawali B, Harasym MC, Hassan S, Cooper K, Boschma A, Bird M, et al. Not an 'either/or': Integrating mental health and psychosocial support within non-communicable disease prevention and care in humanitarian response. J Glob Health. 2021;11:03119.

15.	Jaung MS, Willis R, Sharma P, Aebischer Perone S, Frederiksen S, Truppa C, et al. Models of care for patients with hypertension and diabetes in humanitarian crises: a systematic review. Health Policy Plan. 2021;36(4):509–32.

16.	Karak FE, Rawadi E, Sawan J, Haddad FG. The impact of disasters on cancer care in Lebanon. Future Oncol. 2021;17(6):629–31.

17.	Ansbro E, Issa R, Willis R, Blanchet K, Perel P, Roberts B. Chronic NCD care in crises: A qualitative study of global experts' perspectives on models of care for hypertension and diabetes in humanitarian settings. J Migr Health. 2022;5:100094.

18.	Kehlenbrink S, Ansbro E, Besancon S, Hassan S, Roberts B, Jobanputra K. Strengthening Diabetes Care in Humanitarian Crises in Low- and Middle-income Settings. J Clin Endocrinol Metab. 2022;107(9):e3553–e61.

19.	Leff R, Selvam A, Bernstein R, Wallace L, Hayward A, Agrawal P, et al. A review of interventions for noncommunicable diseases in humanitarian emergencies in low- and middle-income countries. Am J Disaster Med. 2022;16(4):297–311.

20.	Ngaruiya C, Bernstein R, Leff R, Wallace L, Agrawal P, Selvam A, et al. Systematic review on chronic non-communicable disease in disaster settings. BMC Public Health. 2022;22(1):1234.

21.	Odhaib SA, Masood SN, Belkhadir J, Sandid M, Shaikh Z, Farooq FN, et al. Diabetes Care during Humanitarian Crises due to Floodings and Earthquakes in IDF-MENA Region. Journal of Diabetology. 2022;13(Suppl 1):S62–S7.

22.	Taylor P, Berg C, Thompson J, Dean K, Yuan T, Nallamshetty S, et al. Effective Access to Care in a Crisis Period: Hypertension Control During the COVID-19 Pandemic by Telemedicine. Mayo Clin Proc Innov Qual Outcomes. 2022;6(1):19–26.

23.	Al-Nimer MS. Barriers to Diabetes Care during Humanitarian Crisis during 2013–2022 in Five Arabian Countries: A Systematic Review. Clinical Diabetology. 2023;12(2):123–34.

24.	Ide-Okochi A, He M, Murayama H, Samiso T, Yoshinaga N. Noncompliance with Hypertension Treatment and Related Factors among Kumamoto Earthquake Victims Who Experienced the COVID-19 Pandemic during Postearthquake Recovery Period. Int J Environ Res Public Health. 2023;20(6).

25.	Kehlenbrink S, Jobanputra K, Reddy A, Boulle P, Gomber A, Nugent R, et al. Diabetes Care in Humanitarian Settings. Endocrinol Metab Clin North Am. 2023;52(4):603–15.

26.	Moslehi S, Shirazi FB. Challenges of providing health services to patients with cardiovascular diseases during disasters in Iran: A qualitative study. J Educ Health Promot. 2023;12(1):25.

27.	Pitayarangsarit S, Bhagaman N, Yodmai K, Thangsirikul N, Tipayamongkholgul M. The resiliency of noncommunicable diseases services during the public health crisis: a lesson from Bangkok, Thailand. BMC Health Serv Res. 2023;23(1):409.

28.	Willis R, Akik C, El-Dirani Z, Truppa C, Zmeter C, Fleri F, et al. Patient experiences of diabetes and hypertension care during an evolving humanitarian crisis in Lebanon: A qualitative study. PLOS Glob Public Health. 2023;3(12):e0001383.

29.	Bhanu Pratap IG. Vision and Framework 2030: Non-Communicable Diseases: NCD prevention, control, management, care and access to services for everyone everywhere at all times in all contexts. 2024.

30.	Organization WH. Addressing noncommunicable diseases in emergencies: a regional framework for action. World Health Organization. Regional Office for the Eastern Mediterranean; 2024.

31.	Organization WH. Operational manual on noncommunicable diseases in all-hazards emergency preparedness and response. 2024.

32.	Sert G, Gozderesi Y, Kesen Yurtcanli Yurtcanl CH, Baris M, Hidiroglu S. Cancer Care After Turkiye-Syrian Earthquakes: A Qualitative Study. Disaster Med Public Health Prep. 2024;18:e141.

33.	Vijayasingham L, Ansbro E, Zmeter C, Abbas LA, Schmid B, Sanga L, et al. Implementing and evaluating integrated care models for non-communicable diseases in fragile and humanitarian settings. J Migr Health. 2024;9:100228.

34.	Alliance N. Strengthening the health system response to NCDs in humanitarian settings (Case study). 2025.

35.	IFRC. NCD in Humanitarian Settings: Key Advocacy Asks 2025.

36.	Organization WH. Strengthening noncommunicable disease integration in all-hazards emergency preparedness and response: policy brief: World Health Organization; 2025.

37.	UN.Secretary-General. Progress on the prevention and control of non-communicablediseases and the promotion of mental health and well-being. 2025.
