# Supplementary material for: Health-system challenges, resilience strategies, and actionable priorities for non-communicable disease care in humanitarian crises: A scoping review
Source: PLOS Glob Public Health. 2026 Aug 18;6(8):e0006035. doi: 10.1371/journal.pgph.0006035 (PMC13485051; doi:10.1371/journal.pgph.0006035)
Supplement: Supplementary file 3 [file pgph.0006035.s003.docx]

**Table S3. Evidence profile and directness of evidence across operational domains**

| Operational domain | Total contributing sources, n | Empirical primary studies, n: sources | Patient/clinical outcome evidence, n: sources | Service/system or implementation evidence, n: sources | Descriptive or recommendation-based evidence, n: sources | Sources with explicit economic/cost data, n: sources | Principal evidence gap |
| --- | --- | --- | --- | --- | --- | --- | --- |
| Strategic and operational planning | 30 sources | 3 sources: Ansbro, 2022; Moslehi, 2023; Sert, 2024 | 5 sources: Ruby, 2015; Ryan, 2015; Man, 2018; Jaung, 2021; Leff, 2022 | 11 sources: Kishimoto, 2013; Tonelli, 2016; Boulle, 2019; El Karak, 2021; Ansbro, 2022; Odhaib, 2022; Al-Nimer, 2023; Moslehi, 2023; Sert, 2024; Cooper, 2018; NCD Alliance, 2025 | 14 sources: Gyawali, 2021; Kehlenbrink, 2022; Aebischer Perone, 2018; WHO, 2024; Cooper, 2018; IFRC, 2025; UN Secretary-General, 2025; Pratap, 2024; Vijayasingham, 2024; Kehlenbrink, 2023; WHO, 2024; WHO, 2025; UNHCR, 2020; WHO, 2016 | 1sources: Tonelli, 2016 | Predominantly guidance-based; few evaluations of national or organizational plan implementation and limited linkage to patient outcomes. |
| Service delivery | 36 sources | 7: Nakaya, 2017 ; Ansbro, 2022; Taylor, 2022; Ide-Okochi, 2023; Pitayarangsarit, 2023; Willis, 2023; Sert, 2024 | 9: Ruby, 2015; Ryan, 2015; Nakaya, 2017; Man, 2018; Kehlenbrink, 2019; Taylor, 2022; Ide-Okochi, 2023; Pitayarangsarit, 2023; Ngaruiya, 2022 | 13: Kishimoto, 2013; Tonelli, 2016; Boulle, 2019; Jaung, 2021; El Karak, 2021; Ansbro, 2022; Odhaib, 2022; Al-Nimer, 2023; Moslehi, 2023; Willis, 2023; Sert, 2024; Leff, 2022; NCD Alliance, 2025 | 14: Gyawali, 2021; Kehlenbrink, 2022; Aebischer Perone, 2018; WHO, 2024; Cooper, 2018; UN Secretary-General, 2025; Pratap, 2024; Vijayasingham, 2024; Kehlenbrink, 2023; Cooper, 2018; WHO, 2024; WHO, 2025; UNHCR, 2020; WHO, 2016 | 3: Tonelli, 2016; Ansbro, 2022; Al-Nimer, 2023 | Largest evidence base, but interventions were heterogeneous, context-specific, and rarely evaluated for long-term effectiveness or scalability. |
| Medicines, supplies, and logistics | 32 sources | 4: Ansbro, 2022; Moslehi, 2023; Pitayarangsarit, 2023; Willis, 2023 | 7: Ruby, 2015; Ryan, 2015; Man, 2018; Kehlenbrink, 2019; Jaung, 2021; Pitayarangsarit, 2023; Ngaruiya, 2022 | 12: Kishimoto, 2013; Tonelli, 2016; Boulle, 2019; El Karak, 2021; Ansbro, 2022; Odhaib, 2022; Al-Nimer, 2023; Moslehi, 2023; Willis, 2023; Sert, 2024; Leff, 2022; Cooper, 2018 | 13: Kehlenbrink, 2022; Aebischer Perone, 2018; WHO, 2024; Cooper, 2018; IFRC, 2025; UN Secretary-General, 2025; Pratap, 2024; Kehlenbrink, 2023; NCD Alliance, 2025; WHO, 2024; WHO, 2025; UNHCR, 2020; WHO, 2016 | 2: Tonelli, 2016; Ansbro, 2022 | Frequent documentation of shortages, but few evaluated supply-chain, buffer-stock, redistribution, or cold-chain interventions. |
| Training and capacity building | 29 sources | 3: Ansbro, 2022; Moslehi, 2023; Sert, 2024 | 6: Ruby, 2015; Ryan, 2015; Man, 2018; Jaung, 2021; Leff, 2022; Ngaruiya, 2022 | 10: Kishimoto, 2013; Boulle, 2019; Ansbro, 2022; Odhaib, 2022; Al-Nimer, 2023; Moslehi, 2023; Sert, 2024; Cooper, 2018; NCD Alliance, 2025; Vijayasingham, 2024 | 13: Gyawali, 2021; Kehlenbrink, 2022; Aebischer Perone, 2018; WHO, 2024; Cooper, 2018; IFRC, 2025; UN Secretary-General, 2025; Pratap, 2024; Kehlenbrink, 2023; WHO, 2024; WHO, 2025; UNHCR, 2020; WHO, 2016 | 0: None identified | Training was usually embedded within multicomponent programmes; competency, retention, and independent effects on outcomes were seldom assessed. |
| Financing and budget planning | 22 sources | 2: Ide-Okochi, 2023; Willis, 2023 | 3: Ruby, 2015; Man, 2018; Ide-Okochi, 2023 | 6: Tonelli, 2016; El Karak, 2021; Ansbro, 2022; Al-Nimer, 2023; Willis, 2023; Leff, 2022 | 13: Ryan, 2015; Boulle, 2019; Gyawali, 2021; Kehlenbrink, 2022; Aebischer Perone, 2018; WHO, 2024; Cooper, 2018; IFRC, 2025; UN Secretary-General, 2025; Pratap, 2024; Vijayasingham, 2024; Kehlenbrink, 2023; Cooper, 2018 | 4: Tonelli, 2016; Ansbro, 2022; Al-Nimer, 2023; Ide-Okochi, 2023 | Evidence was mainly descriptive or normative; only limited costing, affordability, or expenditure data were available and no full comparative economic evaluation was identified. |
| Community engagement and support | 30 sources | 6: Nakaya, 2017; Ansbro, 2022; Ide-Okochi, 2023; Moslehi, 2023; Willis, 2023; Sert, 2024 | 7: Ruby, 2015; Nakaya, 2017; Man, 2018; Kehlenbrink, 2019; Jaung, 2021; Ide-Okochi, 2023; Ngaruiya, 2022 | 10: Kishimoto, 2013; Boulle, 2019; Ansbro, 2022; Odhaib, 2022; Al-Nimer, 2023; Moslehi, 2023; Willis, 2023; Sert, 2024; Cooper, 2018; NCD Alliance, 2025 | 13: Ryan, 2015; Gyawali, 2021; Kehlenbrink, 2022; Aebischer Perone, 2018; WHO, 2024; Leff, 2022; Cooper, 2018; IFRC, 2025; UN Secretary-General, 2025; Pratap, 2024; Kehlenbrink, 2023; WHO, 2024; UNHCR, 2020 | 0: None identified | Limited co-design with patients and practitioners, and few formal evaluations of peer support, outreach, feedback, or self-management interventions. |
| Intersectoral coordination and partnerships | 24 sources | 3: Ansbro, 2022; Willis, 2023; Sert, 2024 | 3: Ryan, 2015; Man, 2018; Jaung, 2021 | 8: Boulle, 2019; El Karak, 2021; Ansbro, 2022; Odhaib, 2022; Al-Nimer, 2023; Willis, 2023; Sert, 2024; Cooper, 2018 | 13: Gyawali, 2021; Kehlenbrink, 2022; Aebischer Perone, 2018; WHO, 2024; Leff, 2022; Cooper, 2018; IFRC, 2025; UN Secretary-General, 2025; Pratap, 2024; Vijayasingham, 2024; NCD Alliance, 2025; WHO, 2024; UNHCR, 2020 | 0: None identified | Mostly supported by frameworks and recommendations; standardized coordination indicators and evidence of effects on continuity or outcomes were lacking. |
| Monitoring, evaluation, and learning | 18 sources | 4: Nakaya, 2017; Taylor, 2022; Ide-Okochi, 2023; Pitayarangsarit, 2023 | 7: Ruby, 2015; Nakaya, 2017; Man, 2018; Jaung, 2021; Taylor, 2022; Ide-Okochi, 2023; Pitayarangsarit, 2023 | 2: Kehlenbrink, 2019; NCD Alliance, 2025 | 9: Ryan, 2015; Tonelli, 2016; Aebischer Perone, 2018; WHO, 2024; Leff, 2022; Vijayasingham, 2024; WHO, 2024; Ngaruiya, 2022; UNHCR, 2020 | 0: None identified | Few standardized indicators, inconsistent denominators and outcome definitions, and limited longitudinal assessment of programme adaptation. |
| Health information systems and digital technologies | 25 sources | 4: Ansbro, 2022; Taylor, 2022; Pitayarangsarit, 2023; Willis, 2023 | 7: Ruby, 2015; Ryan, 2015; Man, 2018; Kehlenbrink, 2019; Jaung, 2021; Taylor, 2022; NCD Alliance, 2025 | 8: Kishimoto, 2013; Boulle, 2019; Ansbro, 2022; Al-Nimer, 2023; Willis, 2023; Leff, 2022; Cooper, 2018; Vijayasingham, 2024 | 10: Kehlenbrink, 2022; Aebischer Perone, 2018; WHO, 2024; Cooper, 2018; IFRC, 2025; UN Secretary-General, 2025; Pratap, 2024; WHO, 2024; WHO, 2025; UNHCR, 2020 | 0: None identified | Promising EMR and telemedicine examples were reported, but interoperability, equity, low-connectivity feasibility, privacy, and sustained effectiveness remain uncertain. |
