# Supplementary material for: Health-system challenges, resilience strategies, and actionable priorities for non-communicable disease care in humanitarian crises: A scoping review
Source: PLOS Glob Public Health. 2026 Aug 18;6(8):e0006035. doi: 10.1371/journal.pgph.0006035 (PMC13485051; doi:10.1371/journal.pgph.0006035)
Supplement: Supplementary file 4 [file pgph.0006035.s004.docx]

**Table S4. Key findings and statistics underpinning the evidence profile across operational domains**

| Operational domain | Findings from empirical primary studies | Patient/clinical outcome findings | Service/system or implementation findings | Descriptive or recommendation-based findings | Economic/cost findings |
| --- | --- | --- | --- | --- | --- |
| Strategic and operational planning | • Ansbro et al. found that global experts considered crisis-specific protocols, phased models of care, and advance identification of insulin-dependent patients essential; insulin expenditure could consume up to 50% of some programme budgets.  • Moslehi and Shirazi identified advance medicine-distribution plans, patient medicine bags, referral pathways, workforce preparation, and psychosocial support as central requirements for cardiovascular continuity.  • Sert et al. interviewed 15 cancer-association volunteers and identified three themes and 11 subthemes concerning earthquake-related disruption, access to care, and future continuity concerns. | • In the programmes reviewed by Ruby et al., a structured heart-failure programme increased ejection fraction by 4.1 percentage points and reduced hospital admissions by 52.5% and emergency visits by 40.7% (all p<0.001).  • After hurricane-action planning in paediatric oncology, 78% of families had evacuation plans, 71% stored two weeks of medicines, 43% carried treatment plans, and 92% rated the plan as extremely useful.  • Intervention reviews nevertheless found sparse, heterogeneous evidence and limited ability to attribute patient benefits specifically to preparedness planning. | • Tonelli et al. modelled a 90-day NCD extension to the Interagency Emergency Health Kit, linking medicine quantities to country-specific burden and acute versus stable-care scenarios.  • Operational examples included mobile and chronic-care clinics, patient registries, simplified protocols, referral planning, and disaster-specific oncology or diabetes plans.  • Qualitative studies consistently reported that the absence of unified protocols and continuity plans increased reliance on improvised, patient-led navigation. | • Guidelines and frameworks consistently recommend integrating NCDs into national and humanitarian plans, establishing standard operating procedures, identifying high-risk patients, maintaining registries, and defining referral and evacuation pathways.  • The recommendations are operationally coherent but largely normative; most sources did not evaluate whether adoption of these planning components improved service continuity or patient outcomes. | • Tonelli et al. estimated a total Scenario 2 kit cost of US$22,208 for a 90-day population package; projected hypertension medicines cost less than US$160 in total. This was a costing model, not a comparative cost-effectiveness analysis. |
| Service delivery | • Taylor et al. evaluated 569 telemedicine patients: 62.4% initially had systolic BP >140 mmHg, 55.7% of initially uncontrolled patients achieved control, 77% improved systolic or diastolic BP, and 84% received a new antihypertensive prescription.  • Ide-Okochi et al. found 31.2% hypertension-treatment noncompliance during earthquake recovery and COVID-19; pandemic-related income reduction was associated with noncompliance (adjusted OR 3.23, 95% CI 2.27–4.58).  • In Bangkok, fewer than 25% of facilities maintained essential diabetes services for >95% of the target population; follow-up coverage fell from 18.4% to 12.0%, and home-visit coverage from 6.5% to 4.1% across pandemic waves. | • Ruby et al. reported 74% BP control among monitored hypertensive patients and 65% postprandial glucose, 63% cholesterol, and 87% BP target attainment among diabetes cohorts; however, complication screening remained incomplete.  • Severe psychological distress was associated with fourfold higher odds of hypertension-treatment withdrawal after the Great East Japan earthquake (OR 4.0, 95% CI 1.3–10.6; Nakaya et al.).  • Disaster reviews documented delayed stroke presentation, elevated cardiovascular events, poor glycaemic control, and interrupted cancer treatment, but findings were largely observational and heterogeneous. | • UNRWA and humanitarian clinic models using standardized pathways, periodic follow-up, and cohort monitoring achieved high patient retention: 76% among 4,130 hypertensive patients and 78% among 12,550 diabetes patients in programmes summarized by Ruby et al..  • A doorstep/teleconsultation programme in Pakistan reached approximately 1,800 patients with diabetes, illustrating adaptive delivery during disrupted access.  • Qualitative evidence showed that fragmented provider networks, repeated out-of-pocket visits, and absent case coordination shifted substantial navigation work to patients and families. | • Recommended delivery models include integrated primary care, mobile and outreach clinics, telemedicine, task-sharing, simplified protocols, structured referral, palliative care, and multi-month dispensing.  • Most guidance does not specify comparative effectiveness, minimum staffing, sustainable coverage thresholds, or which delivery adaptations perform best in different crisis phases. | • Cost-related findings were limited. Tonelli et al. modelled medicine-package costs; Ansbro et al. reported that insulin could consume up to 50% of some programme budgets; Al-Nimer reported financial hardship as the main access barrier for 31.6% of 1,363 Syrian refugees with diabetes. |
| Medicines, supplies, and logistics | • Experts and providers repeatedly described insulin shortages, cold-chain fragility, medicine rationing, and reliance on informal procurement as immediate threats to continuity.  • During Bangkok's second COVID-19 wave, 77.8% of facilities reported partial or complete redeployment of diabetes staff, coinciding with deterioration in medicine counselling and follow-up functions. | • Among Syrian refugees with type 1 diabetes in Iraq, 63.6% reported symptomatic hypoglycaemia and 36.6% at least one episode of diabetic ketoacidosis; mean HbA1c was 9.3%, with 81.8% above 8.0%.  • Only 5.5% of hypertensive patients in a conflict-affected Kosovo population were receiving regular treatment; after the Sichuan earthquake, 43.4% of 762 assessed patients had hypertensive readings.  • Cancer-care reviews documented destroyed oncology inventories, pharmacy closure, treatment delays, and displacement of more than 100 paediatric oncology patients after Hurricane Katrina. | • The proposed 90-day emergency kit included acute and chronic medicines for hypertension, cardiovascular disease, diabetes, asthma, and COPD, with quantities derived from population burden.  • Operational sources described pre-positioned kits, mobile pharmacy support, visual medication catalogues, alternative insulin-storage practices, and medicine delivery through clinics or households.  • Evidence was mainly feasibility-oriented; few sources evaluated stock-out duration, redistribution performance, cold-chain failure rates, or patient outcomes before and after a logistics intervention. | • Guidance recommends essential NCD medicine lists, two- to four-week patient backups, multi-month dispensing, buffer stocks, cold-chain contingency, real-time inventory monitoring, and procurement/redistribution arrangements.  • These recommendations are consistent across frameworks, but implementation requirements and supply-chain performance indicators are rarely standardized. | • Tonelli et al. provided unit and total medicine costs, including amlodipine at US$0.006/tablet, hydrochlorothiazide at US$0.0026/tablet, and a total 90-day Scenario 2 package cost of US$22,208. Ansbro et al. reported that insulin could account for up to 50% of programme budgets. |
| Training and capacity building | • Global experts identified inconsistent provider competence, absent or variably applied protocols, and high staff turnover as barriers to safe hypertension and diabetes management.  • Iranian providers emphasized advance training in cardiovascular triage, medicine management, referral, and psychosocial support (Moslehi and Shirazi); cancer-association volunteers similarly identified preparedness and continuity skills as post-earthquake needs. | • Training was generally embedded in multicomponent programmes. In the heart-failure programme reviewed by Ruby et al., physician training accompanied a 52.5% reduction in admissions and 40.7% reduction in emergency visits, but the independent training effect could not be isolated.  • Following Typhoon Haiyan, one-third of surgical procedures were performed in patients with type 2 diabetes because of sepsis from foot injuries, illustrating preparedness and clinical-skills gaps.  • Reviews consistently linked inadequate recognition and management capacity to delayed or unsafe care, but rarely measured provider competency before and after training. | • In Lebanon, 70% of 235 hospitalized patients with hyperglycaemia were reportedly mismanaged by nursing staff, mainly through incorrect insulin administration, supporting targeted protocol-based training (Al-Nimer).  • Programmes used physician training, train-the-trainer models, task-sharing, simplified algorithms, and community-health-worker education; however, most evaluations reported overall programme outcomes rather than training fidelity or skill retention. | • Operational guidance recommends pre-deployment and refresher training in acute NCD presentations, syndromic management, referral, medicine logistics, digital systems, psychological first aid, and supportive supervision.  • No common competency framework, minimum training dose, or cross-context evaluation standard was identified. | • No explicit numerical economic or cost evaluation of training interventions was identified in sources mapped to this domain. |
| Financing and budget planning | • Ide-Okochi et al. found that reduced income during COVID-19 was associated with treatment noncompliance (adjusted OR 3.23, 95% CI 2.27–4.58); 31.2% of respondents were noncompliant overall.  • Patients in Lebanon described choosing between food, consultations, laboratory tests, and medicines, sometimes selling belongings or relying on relatives to obtain insulin. | • Cost barriers contributed to delayed or interrupted treatment across hypertension, diabetes, and cancer. In the Kumamoto cohort, poor self-rated health (adjusted OR 2.49, 95% CI 1.72–3.61) and unstable housing were also associated with noncompliance.  • Cancer-care disruptions after major disasters included service closure, long delays, and displacement; financial and infrastructure constraints compounded clinical risk.  • Free or subsidized programmes achieved measurable retention and control in some humanitarian cohorts, but financing was rarely evaluated as an independent determinant of outcomes. | • Insulin consumed up to 50% of some programme budgets; 31.6% of 1,363 Syrian refugees with diabetes identified financial hardship as their main access barrier (Al-Nimer).  • In a humanitarian funding mechanism reviewed by Leff et al., 15% of 20 brain-tumour applications were only partially funded, receiving on average 39% of requested support; condition-specific denial rates were also substantial.  • Lebanon's economic crisis, with unemployment reported at 40% in 2020, made basic cancer diagnosis and treatment increasingly unaffordable. | • Frameworks recommend dedicated NCD budget lines, protected allocations during emergency reprogramming, pooled financing, subsidies or fee waivers, and funding that extends beyond acute relief.  • Most financing recommendations were not accompanied by budget-impact estimates, affordability thresholds, or comparative economic evaluation. | • Tonelli et al. estimated the 90-day expanded kit at US$22,208. Other evidence consisted of programme-budget burden (insulin up to 50%), patient-reported affordability barriers, and service-funding shortfalls rather than formal cost-effectiveness, cost-utility, or cost-benefit analyses. |
| Community engagement and support | • Nakaya et al. demonstrated that severe psychological distress was associated with treatment withdrawal (OR 4.0, 95% CI 1.3–10.6), supporting community identification and psychosocial outreach.  • Qualitative studies described families locating medicines, financing care, transporting patients, and transferring information between providers, effectively compensating for fragmented systems.  • Sert et al. showed that cancer-patient associations could identify access and future-care concerns after earthquakes, but patients themselves were not directly involved in designing the review's priorities. | • A paediatric oncology hurricane plan was rated extremely useful by 92% of families; 78% had evacuation plans and 71% had two-week medicine supplies after implementation.  • Housing instability after the Kumamoto earthquake showed a graded association with treatment noncompliance: adjusted ORs were 1.92 for rental housing, 2.47 for public housing, and 4.12 for restoration public housing.  • Evidence suggests that social and psychological vulnerability materially affects adherence and continuity, but formal trials of peer or community-support interventions were scarce. | • Community and home-based adaptations included household surveys, peer or family support, community-health-worker outreach, patient education, and doorstep diabetes services reaching approximately 1,800 patients in Pakistan.  • In humanitarian diabetes settings, trust and satisfaction with providers were associated with better adherence, while 42.1% of 380 Palestinian patients reported low adherence linked to beliefs that medicines were ineffective (Al-Nimer).  • Most programmes reported reach or implementation experience rather than comparative effects of community engagement on clinical outcomes. | • Guidance recommends involving people with NCDs in planning, formal feedback channels, culturally adapted communication, peer groups, self-management training, patient-held records, and trusted community actors.  • The evidence base contains limited documented co-design with patients or frontline humanitarian practitioners and few standardized measures of acceptability, participation, or equity. | • No explicit numerical economic or cost evaluation of community-engagement interventions was identified in sources mapped to this domain. |
| Intersectoral coordination and partnerships | • Ansbro et al., Willis et al., and Sert et al. consistently described fragmented referrals, absent information sharing, and unclear responsibilities across providers and organizations.  • Patients in Lebanon had to manually carry results and prescriptions between facilities, increasing workload, duplication, and risk of error. | • Cancer-care reviews documented transfer of more than 100 paediatric patients, disruption of radiotherapy, and treatment delays after Hurricane Katrina, demonstrating the clinical consequences of uncoordinated regional capacity.  • Disaster and care-model reviews linked fragmented coordination to delayed follow-up, medicine interruption, and reduced access, although few studies tested coordination mechanisms directly. | • Implemented responses included online emergency communication platforms, oncologist-to-oncologist handovers, service transfers, interagency NCD groups, and coordination among health, logistics, and community teams.  • These examples demonstrated feasibility but rarely reported coordination coverage, referral completion, duplication avoided, or comparative service outcomes. | • Frameworks recommend multisector working groups, standardized referral and treatment protocols, shared information systems, alignment with health clusters and national plans, and partnerships with specialist organizations and civil society.  • Evidence remains predominantly normative, with limited specification of governance authority, accountability, or measurable coordination indicators. | • No explicit numerical economic or cost evaluation of intersectoral coordination mechanisms was identified in sources mapped to this domain. |
| Monitoring, evaluation, and learning | • Taylor et al. used repeated home BP readings and telemedicine follow-up to demonstrate improvement in 77% of 569 patients and control in 55.7% of those initially uncontrolled.  • Ide-Okochi et al. quantified treatment noncompliance and socioeconomic predictors, while Nakaya et al. used the validated Kessler-6 scale to identify a strong association between distress and withdrawal from care.  • Pitayarangsarit et al. documented declining diabetes-service coverage between pandemic waves, including follow-up coverage of 18.4% then 12.0% and home-visit coverage of 6.5% then 4.1%. | • Patient-level monitoring identified poor control and major complications: among Syrian refugees with type 1 diabetes, 63.6% reported symptomatic hypoglycaemia and 36.6% diabetic ketoacidosis.  • UNRWA programme data showed measurable target attainment, while disaster reviews documented delayed presentation and treatment interruption; however, outcome definitions and denominators varied substantially. | • Electronic cohort monitoring improved documentation in UNRWA diabetes clinics: postprandial glucose recording increased from 42% of 2,851 patients to 99% of 9,691 patients one year later, while the proportion controlled increased from 50% to 65%.  • Case-study programmes used screening and follow-up data to identify patients and adjust services, but few reported sustained learning cycles or prespecified implementation outcomes. | • Operational sources recommend NCD indicators in emergency dashboards, disaggregation by age, sex, disease, and vulnerability, real-time surveillance, mixed-method assessments, and feedback loops linking data to resource allocation.  • No common minimum indicator set or standardized reporting schedule was consistently applied across the empirical literature. | • No explicit numerical economic or cost evaluation of monitoring and evaluation systems was identified in sources mapped to this domain. |
| Health information systems and digital technologies | • Taylor et al. showed that a video-visit model with home BP monitoring maintained care for 569 patients and produced clinically meaningful BP improvement; patient ratings were high, with 96.6% of respondents rating visits 4 or 5.  • Ansbro et al. and Willis et al. identified fragmented or non-portable records as major continuity barriers; shared records and patient-held information were proposed as practical adaptations.  • Pitayarangsarit et al. documented virtual consultations and alternative medicine pickup processes during COVID-19, but digital coverage and comparative effectiveness were not comprehensively reported. | • EMR-supported cohorts summarized by Ruby et al. retained 76% of 4,130 hypertensive patients and 78% of 12,550 diabetes patients in care, with measurable clinical-target attainment.  • Telemedicine reduced mean systolic BP by 9.7 mmHg and diastolic BP by 6.8 mmHg in Taylor et al..  • The evidence suggests potential clinical benefit, but most studies combined digital tools with medications, protocols, and active follow-up, preventing isolation of the digital component. | • UNRWA eHealth monitoring increased glucose documentation from 42% to 99% and control from 50% to 65% across successive cohorts.  • Other applications included EMR registries, eHealth notebooks for screening and referral, teleconsultation, online emergency communication, and patient-held records.  • Interoperability, low-connectivity performance, privacy, maintenance, and cross-border portability were rarely evaluated. | • Guidance recommends interoperable EMRs, digital or paper health passports, mHealth follow-up, telemedicine, SMS feedback, and backup communication systems, while retaining low-technology alternatives where connectivity is unreliable.  • Most recommendations do not include implementation costs, data-governance standards, or equity assessments for digitally excluded populations. | • No explicit numerical economic or cost evaluation of digital-health or information-system interventions was identified in sources mapped to this domain. |
