# Supplementary material for: Health-system challenges, resilience strategies, and actionable priorities for non-communicable disease care in humanitarian crises: A scoping review
Source: PLOS Glob Public Health. 2026 Aug 18;6(8):e0006035. doi: 10.1371/journal.pgph.0006035 (PMC13485051; doi:10.1371/journal.pgph.0006035)
Supplement: Supplementary file 5 [file pgph.0006035.s005.docx]

**S1 PRISMA Checklist**

**Health-System Challenges, Resilience Strategies, and Actionable Priorities for Non-Communicable Disease Care in Humanitarian Crises: A Scoping Review**

| **Section** | **Item** | **Checklist item** | **PRISMA-ScR requirement** | **Status** | **Reported on page(s)** | **Location/notes for this manuscript** |
| --- | --- | --- | --- | --- | --- | --- |
| TITLE | 1 | Title | Identify the report as a scoping review. | Reported | Page 1 | Title includes “A Scoping Review”. |
| ABSTRACT | 2 | Structured summary | Provide a structured summary including background, objectives, eligibility criteria, sources of evidence, charting methods, results, and conclusions. | Reported | Page 2 | Abstract summarizes rationale, objective, databases, eligibility, evidence synthesis, main findings, and implications. |
| INTRODUCTION | 3 | Rationale | Describe the rationale for the review in the context of what is already known and why a scoping review is needed. | Reported | Pages 3–4 | Introduction describes the NCD burden in humanitarian crises, fragmentation of existing evidence, and the need to map challenges, strategies, evidence directness, and actionable priorities. |
| INTRODUCTION | 4 | Objectives | Provide an explicit statement of the questions and objectives being addressed with reference to participants, concepts, and context. | Reported | Page 4; page 5 | End of Introduction states the review aims; Methods states the primary review question and target NCDs, humanitarian settings, challenges, interventions, and recommended strategies. |
| METHODS | 5 | Protocol and registration | Indicate whether a review protocol exists and whether it is available; provide registration information if available. | Not reported | Not reported | The manuscript does not state that a protocol was registered or published. If applicable, add a brief statement such as “The review was not prospectively registered and no protocol was published.” |
| METHODS | 6 | Eligibility criteria | Specify characteristics of sources of evidence used as eligibility criteria and provide rationale. | Reported | Pages 6–7 | Eligibility criteria define publication period, language, NCD groups, humanitarian/crisis settings, eligible concepts/actions, source types, and exclusion criteria. |
| METHODS | 7 | Information sources | Describe all information sources in the search and the date the most recent search was executed. | Partly reported | Pages 5–6; Appendix Table S1, page 1 | Databases and supplementary organizational/hand searches are reported. The publication period is stated. The exact date the final search was run should be confirmed if required by the journal. |
| METHODS | 8 | Search | Present the full electronic search strategy for at least one database, including any limits used. | Reported | Appendix Table S1, page 1 | Complete PubMed/MEDLINE, Scopus, and Web of Science strategies with limits and identified records are provided in Supplementary Table S1. |
| METHODS | 9 | Selection of sources of evidence | State the process for selecting sources of evidence included in the scoping review. | Reported | Page 7; Fig 1 | Two independent reviewers screened titles/abstracts and full texts, with disagreements resolved by discussion and third-reviewer consultation. PRISMA-ScR-compatible flow diagram is provided. |
| METHODS | 10 | Data charting process | Describe methods of charting data from included sources and any calibration process. | Reported | Pages 7–8 | Data were extracted by one reviewer using a piloted standardized Excel form and independently verified by a second reviewer; discrepancies were resolved by discussion or a third reviewer. |
| METHODS | 11 | Data items | List and define all variables for which data were sought and any assumptions or simplifications. | Reported | Pages 7–8 | Data items included source characteristics, crisis type, NCD group, target population, challenges, actions/strategies, continuity of care, preparedness, medicines, technologies, equipment, implementation, service, and patient outcomes. |
| METHODS | 12 | Critical appraisal of individual sources of evidence | If done, provide rationale and methods for critical appraisal of included sources. | Not applicable / not performed | Page 9; page 25 | No formal critical appraisal or certainty assessment was undertaken; evidence was characterized by type and directness rather than quality or certainty. |
| METHODS | 13 | Synthesis of results | Describe methods of handling and summarizing the data that were charted. | Reported | Pages 8–9 | Narrative and thematic synthesis is described, including inductive coding, deductive comparison with health-system concepts, nine operational domains, non-mutually exclusive categories, and characterization of evidence directness and economic evidence. |
| RESULTS | 14 | Selection of sources of evidence | Give numbers of sources screened, assessed for eligibility, and included, with reasons for exclusion at each stage, ideally using a flow diagram. | Reported | Page 9; Fig 1 | Results report 774 database records, 62 duplicates, 712 screened, 496 excluded, 216 full texts assessed, 179 excluded with reasons, and 37 included sources. Fig 1 provides the flow diagram. |
| RESULTS | 15 | Characteristics of sources of evidence | For each source, present characteristics for which data were charted and provide citations. | Reported | Pages 9–10; Appendix Table S2, pages 3–7 | Main text summarizes publication period, source type, geography, crisis context, and NCD focus. Table S2 provides source-level characteristics and principal contributions for 37 included sources. |
| RESULTS | 16 | Critical appraisal within sources of evidence | If done, present data on critical appraisal of included sources. | Not applicable / not performed | Page 9; page 25 | No formal critical appraisal was performed. The manuscript reports an evidence profile based on evidence type and directness, not methodological quality. |
| RESULTS | 17 | Results of individual sources of evidence | For each included source, present relevant data that were charted. | Reported | Appendix Table S2, pages 3–7; Appendix details from page 8 onward; Supplementary Tables S3–S4 | Source-level characteristics and key contributions are presented in Table S2; detailed extracted information and domain-level findings are provided in the Appendix tables. |
| RESULTS | 18 | Synthesis of results | Summarize and/or present the charting results as they relate to the review questions and objectives. | Reported | Pages 10–21; Supplementary Tables S3–S4 | Results synthesize evidence across operational domains, challenges, implemented strategies, recommended unevaluated strategies, resilience functions, evidence gaps, and cross-cutting priorities. |
| DISCUSSION | 19 | Summary of evidence | Summarize the main results, including how they relate to the review questions and objectives. | Reported | Pages 22–26 | Discussion interprets the evidence map, distinguishes policy consensus from evaluated effectiveness, discusses resilience, implementation evidence, financing, transferability, and implications. |
| DISCUSSION | 20 | Limitations | Discuss limitations of the scoping review process. | Reported | Page 25 | Limitations include English-language restriction, geographic concentration, lack of formal quality/certainty assessment, heterogeneity, non-mutually exclusive domains, no systematic feasibility or equity comparison, no co-design, and narrative priority synthesis. |
| DISCUSSION | 21 | Conclusions | Provide a general interpretation of results with implications for future research, policy, or practice. | Reported | Page 26 | Conclusion identifies the gap between policy consensus and evaluated implementation, and recommends testing three measurable priorities using standardized implementation, clinical, equity, and economic indicators. |
| FUNDING | 22 | Funding | Describe sources of funding for included sources and the scoping review; describe role of funders. | Reported | Page 27 | Manuscript states that the authors received no specific funding for this work. |
