# Supplementary material for: Health-system challenges, resilience strategies, and actionable priorities for non-communicable disease care in humanitarian crises: A scoping review
Source: PLOS Glob Public Health. 2026 Aug 18;6(8):e0006035. doi: 10.1371/journal.pgph.0006035 (PMC13485051; doi:10.1371/journal.pgph.0006035)
Supplement: Supplementary file 6 [file pgph.0006035.s007.docx]

**Dear Editor,**

We sincerely thank you and the three reviewers for the time, expertise, and constructive attention devoted to our manuscript. We are grateful for the careful and insightful comments, which were highly helpful in identifying the main limitations of the initial version and in guiding a substantial revision of the manuscript.

The reviewers’ perspectives were particularly valuable because they encouraged us to move beyond a largely descriptive presentation of challenges and to clarify the analytical contribution of the review. In response, we revised the title, abstract, methods, results, discussion, and conclusion; reorganized the results to distinguish reported challenges, implemented strategies, recommended but unevaluated strategies, resilience functions, evidence gaps, and measurable priorities; and added several supplementary tables to improve transparency and traceability.

In our view, the revised manuscript is now substantially more analytical and useful for readers. It more clearly distinguishes implemented evidence from policy recommendations, characterizes the directness of evidence across operational domains, acknowledges the limits of causal inference and economic evidence, and presents three testable, cross-cutting priorities for strengthening NCD care in humanitarian settings. We hope that the revised version satisfactorily addresses the concerns raised by the reviewers and meets the expectations of the Editor and the journal.

Below, we provide a point-by-point response to each comment. Reviewer comments are reproduced in the left column, followed by our response and the corresponding changes made in the revised manuscript.

**Point-by-point responses**

| **Reviewer comment** | **Authors’ response** | **Changes made in the revised manuscript** |
| --- | --- | --- |
| Reviewer #1: The manuscript appears overly generalized and does not adequately reflect the actual state of preparedness suggested by the title. The title, abstract, and specific findings are not sufficiently aligned.  Reviewer #2: The authors focused primarily on identifying challenges rather than actions and should revise the title. Resilience terminology could also be incorporated into the title. | We agree with the reviewers. The initial title and framing could be read as implying that the review measured the actual preparedness status of countries or humanitarian organizations, whereas the study was designed to map the available evidence on challenges, strategies, and priorities. We therefore revised the title and reframed the manuscript to avoid overstatement and to align the title, abstract, objective, results, discussion, and conclusion. We also incorporated resilience terminology explicitly and reorganized the results around challenges, implemented strategies, recommended strategies, resilience functions, evidence gaps, and actionable priorities. | The title was revised to: “Health-System Challenges, Resilience Strategies, and Actionable Priorities for Non-Communicable Disease Care in Humanitarian Crises: A Scoping Review.” The Introduction now clarifies that the review does not seek to directly measure or rank preparedness. The Abstract and Results now emphasize health-system challenges, resilience strategies, evidence gaps, and actionable priorities rather than implying a direct preparedness assessment. |
| Reviewer #1: The Abstract does not clearly define the central problem, explain how it was addressed, or identify the proposed solutions.  Reviewer #2: The Abstract should include the inclusion and exclusion criteria and explain how the findings were organized into nine categories and whether an existing framework was used. | We revised the abstract to define the central problem more clearly, describe the scoping review approach, specify the eligible source types and disease scope, and summarize how the evidence was synthesized. We also added the key findings regarding evidence directness, implemented strategies, resilience functions, absence of comparative economic evaluation, and the three cross-cutting priorities emerging from the synthesis. | The Abstract now states that PubMed/MEDLINE, Scopus, Web of Science, relevant organizational websites, and reference lists were searched; identifies eligible English-language empirical studies, reviews, programme reports, guidelines, policy documents, and operational frameworks; describes synthesis across nine operational domains; and reports the three specific priorities: a minimum emergency NCD service and medicine package, continuity mechanisms for high-risk patients, and simplified integrated NCD care within emergency and primary-care services. |
| Reviewer #2: PRISMA is intended for systematic reviews, and the authors should have used the PRISMA extension for scoping reviews, PRISMA-ScR. The exclusion criteria following “Studies were excluded if they” should also be properly indented. | We agree. The manuscript has been revised throughout to report the review in accordance with PRISMA-ScR rather than generic PRISMA. We also corrected the formatting of the exclusion criteria for readability and consistency. | The Methods section now explicitly states that the review is reported in accordance with the Preferred Reporting Items for Systematic Reviews and Meta-Analyses extension for Scoping Reviews (PRISMA-ScR). The study-selection process is described using a PRISMA-ScR-compatible flow diagram, and the completed PRISMA-ScR checklist is provided as a supplementary submission file. The exclusion criteria are now presented as a properly indented list. |
| Reviewer #2: The manuscript states that inductive coding was used, although the nine domains resemble the WHO health-system building blocks. The authors should clarify whether a combined inductive and deductive approach was used.  Reviewer #3: The nine-domain framework is descriptive and does not appear to be novel because similar taxonomies exist in WHO and UNHCR guidance. | We agree and have clarified the analytic approach. The original coding was predominantly inductive, but the resulting codes were subsequently compared with established health-system functions and organized using relevant health-system concepts. We no longer present the nine domains as a novel taxonomy. Instead, they are described as an operational synthesis used to organize a heterogeneous evidence base. | The Methods section now states that the analysis was predominantly inductive, followed by deductive comparison and organization using established health-system concepts. The Introduction and Discussion explicitly state that the nine domains overlap with existing health-system and humanitarian frameworks and should not be interpreted as an entirely new taxonomy. Supplementary Table S3 presents the evidence profile across these operational domains. |
| Reviewer #2: The findings should be presented using resilience strategies such as absorption, adaptation, and transformation. | We agree. We revised the Results and Discussion to incorporate a resilience lens based on absorptive, adaptive, and transformative functions. This reframing helped distinguish immediate continuity-preserving measures from service-delivery adaptations and longer-term system reforms. | A new Results subsection, “Contribution of reported strategies to health-system resilience,” has been added. It classifies reported strategies as absorptive, adaptive, or transformative and clarifies that empirical evidence was concentrated mainly on absorptive and adaptive actions, whereas transformative strategies were identified mostly in guidance and policy documents and were rarely evaluated. |
| Reviewer #1: The statistical analysis was not performed appropriately or rigorously.  Reviewer #3: The appropriateness of the statistical analysis was unclear. | We appreciate this concern and have revised the manuscript to avoid any implication that inferential statistical analysis or formal quantitative evidence grading was performed. As a scoping review, the study was intended to map and characterize heterogeneous evidence rather than estimate pooled effects. We therefore clarified that no meta-analysis or inferential statistical testing was undertaken. Counts are used descriptively to summarize the type and directness of evidence across domains. | The Methods now state that the synthesis was narrative and thematic because of heterogeneity in source type, crisis context, disease group, interventions, and outcomes, and that no meta-analysis or inferential statistical testing was undertaken. The Results and table notes clarify that domain counts are descriptive and non-mutually exclusive and should not be interpreted as effect estimates, quality grades, or certainty ratings. |
| Reviewer #3: The review does not critically assess whether included interventions improved patient outcomes, and most of the evidence appears to be process-based. The manuscript should include a table indicating the strength of evidence in each domain, including the number of studies reporting direct outcome data. | We addressed this comment by adding an evidence-profile approach. Rather than assigning a formal certainty grade, which is not typically required for a scoping review and would not be appropriate for the heterogeneous sources included, we characterized each domain by the type and directness of supporting evidence. We distinguished patient or clinical outcomes, service/system or implementation outcomes, descriptive or recommendation-based evidence, empirical primary studies, and sources with economic/cost information. | Supplementary Table S3 now reports, for each operational domain, the number of contributing sources, empirical primary studies, sources with patient/clinical outcomes, sources with service/system or implementation outcomes, descriptive or recommendation-based sources, and sources with explicit economic or cost data. The Results subsection “Evidence profile and directness across operational domains” summarizes these findings and emphasizes that service delivery had the strongest outcome-based profile, whereas several enabling domains were supported mainly by indirect or recommendation-based evidence. |
| Reviewer #3: The manuscript does not prioritize domains or provide analytical synthesis beyond a descriptive taxonomy. It also does not offer causal modeling. | We revised the synthesis to be more analytical while avoiding claims that the design cannot support. Because this is a scoping review of heterogeneous evidence, causal modelling was not feasible or methodologically appropriate. Instead, we added an evidence-informed prioritization section based on recurrence across disease groups and crisis settings, linkage to documented continuity failures, and measurability through implementation indicators. | The Results now end with “Cross-cutting evidence-informed priorities,” which presents three specific priorities and suggested indicators. The Discussion clarifies that these priorities are not a formal ranking, Delphi consensus, or causal model, but an evidence-informed narrative synthesis intended to guide implementation and future evaluation. |
| Reviewer #3: The manuscript should explicitly identify which domains contain cost-effectiveness evidence. | We agree. We re-examined the included sources for economic information and revised the manuscript to distinguish costing, affordability, expenditure, and resource-use information from full comparative economic evaluations. We found some cost-related evidence but no full comparative cost-effectiveness, cost-utility, or cost-benefit evaluation. | The Methods now describe the assessment of economic information. Supplementary Table S3 includes a column for explicit economic/cost data by domain. The Results state that cost-related information was identified in selected domains, but that no full comparative economic evaluation was found. The Discussion includes a dedicated subsection on financing and economic evidence and explains that the review cannot determine whether any specific intervention or domain was cost-effective. |
| Reviewer #1: The manuscript does not clearly explain what solutions are proposed.  Reviewer #2: The review focuses more on identifying challenges than on actions.  Reviewer #3: Vague recommendations for “integration” should be replaced with three specific and testable policy actions. | We agree and substantially revised the Results, Discussion, and Conclusion to distinguish actions that were implemented from strategies that were recommended but unevaluated. We also replaced broad calls for integration with three measurable, cross-cutting priorities that emerged from the synthesis. | The Results now include separate subsections on implemented strategies and evaluated outcomes, recommended strategies without evaluated implementation, and cross-cutting evidence-informed priorities. The three priorities are: (1) establishing a minimum emergency NCD service and medicine package; (2) establishing continuity mechanisms for patients at highest risk of harm from treatment interruption; and (3) integrating simplified NCD care into emergency and primary-care delivery. Suggested indicators are provided for each priority. |
| Reviewer #2: The included evidence is heavily concentrated in the Middle East and Asia, which may indicate geographic bias and should be acknowledged as a limitation.  Reviewer #3: The review does not distinguish feasibility between low- and high-income humanitarian settings. | We agree. We revised the Results to describe the geographic distribution of country-specific empirical evidence and revised the Discussion and limitations to address transferability. We also clarified that the review did not systematically compare feasibility across lower- and higher-resource settings. | The Results now state that directly observed country-level evidence was concentrated predominantly in Asia and the Middle East, with relatively little dedicated empirical research from Africa and Latin America. The Discussion includes a subsection on transferability and recommends distinguishing a minimum core package from context-dependent extensions. The limitations explicitly acknowledge the absence of systematic comparison by income level or resource setting. |
| Reviewer #3: The review does not include patient or humanitarian practitioner co-design. | We agree. The review did not include formal co-design with patients, caregivers, or humanitarian practitioners. We have acknowledged this limitation explicitly and emphasized that future implementation and prioritization work should involve affected communities and frontline practitioners. | The Discussion and limitations now state that patients and practitioners were not involved in co-design or priority setting for this review. The Conclusion also emphasizes that future implementation research should be conducted with affected communities and frontline practitioners. |
| Reviewer #3: Many included references predate 2020, and a substantial proportion of the NCD-humanitarian guidance is older. | We addressed this concern by reporting the publication-year distribution and acknowledging that some empirical and operational evidence predates recent developments in digital health, humanitarian coordination, and all-hazards NCD guidance. At the same time, we note that recurrent continuity problems were also evident in recent sources. | The Results now report that the included sources were published between 2013 and 2025 and that 21 sources were published between 2022 and 2025. The limitations acknowledge that part of the evidence predates recent developments and that applicability to current response systems may vary. |
| Reviewer #3: The review does not compare NCDs with other chronic conditions, such as mental health disorders or dialysis-dependent disease, to determine whether the identified challenges are specific to the four major NCD groups. | We agree that this is an important issue. However, the review was intentionally limited to cardiovascular diseases, diabetes, cancer, and chronic respiratory diseases. We therefore did not conduct a systematic comparison with other chronic conditions. We have clarified this scope and added a limitation noting that cross-condition transferability should be examined directly rather than inferred from this review. | The Methods now state that mental health conditions, dialysis-dependent kidney disease, and other chronic conditions were not treated as independent target conditions unless discussed within eligible sources addressing at least one of the four prespecified NCD groups. The Discussion and limitations now acknowledge that the review cannot determine whether identified challenges and strategies are specific to the four included NCD groups or reflect broader continuity requirements for chronic care. |
| Reviewer #2: The authors should ensure that all 37 included articles are correctly cited in the Results. | We agree. We revised the Results and supplementary material to ensure traceability of all included sources. Because the included evidence comprised studies, reviews, reports, guidelines, and policy documents, we refer to them as “sources” rather than as articles or studies throughout the revised manuscript. | The Results now cite the 37 included sources collectively and individually where relevant. Supplementary Table S2 lists all 37 included sources with source characteristics, context, NCD focus, evidence/outcome type, and principal contribution. Supplementary Table S3 and Supplementary Table S4 further link sources to operational domains and key findings. |
| Reviewer #3: The stated limitations do not sufficiently address the absence of direct outcome evidence, setting-specific feasibility, lack of stakeholder co-design, age of the evidence, and absence of comparison with other chronic conditions. | We agree and expanded the limitations accordingly. The revised limitations now address the lack of direct outcome evidence in many domains, the predominance of descriptive and recommendation-based evidence, geographic concentration, absence of formal co-design, limited economic evidence, heterogeneity of settings and outcomes, and the lack of systematic comparison with other chronic conditions. | The Strengths and limitations section has been substantially revised. It now states that the evidence profile reflects evidence type and directness rather than methodological quality or certainty; that heterogeneity prevented quantitative comparison of effects; that the review did not systematically compare feasibility across lower- and higher-resource settings; that patients and practitioners were not involved in co-design or priority setting; and that mental health, kidney disease, and other chronic conditions were not independently mapped. |
| Reviewers #1, #2, and #3: The manuscript was considered clearly written in standard English. | We thank the reviewers for this positive assessment. Although the manuscript was considered clear, we nevertheless revised the wording extensively to improve precision, consistency, and alignment with the revised structure. | The manuscript was edited for clarity, consistency, and terminology. We standardized the use of terms such as “included sources,” “operational domains,” “resilience strategies,” and “evidence directness.” |
| Reviewers #1, #2, and #3: The data underlying the review findings were considered available. | We thank the reviewers for confirming the availability of the underlying review data. In the revised manuscript, we further improved transparency by adding detailed supplementary tables documenting the search strategy, included sources, domain-level evidence profile, and key findings supporting the synthesis. | The supplementary appendix now includes database-specific search strategies, a characteristics table for all 37 included sources, an evidence-profile table across operational domains, and a key-findings table underpinning the evidence profile. These additions improve reproducibility and make the basis for the narrative synthesis more transparent. |

We again thank the Editor and reviewers for their careful and constructive assessment of our manuscript. We believe that the revised version is substantially stronger, more transparent, and more analytically useful as a result of these comments. We hope that the revisions have adequately addressed the reviewers’ concerns and meet the expectations of the journal.

Sincerely,

Ahad Bakhtiari
On behalf of all authors
