# Supplementary material for: Health-system challenges, resilience strategies, and actionable priorities for non-communicable disease care in humanitarian crises: A scoping review
Source: PLOS Glob Public Health. 2026 Aug 18;6(8):e0006035. doi: 10.1371/journal.pgph.0006035 (PMC13485051; doi:10.1371/journal.pgph.0006035)
Supplement: Supplementary file 7 [file pgph.0006035.s008.docx]

**Response to the Editor and Reviewers**

**Manuscript ID: PGPH-D-26-00504R1**

**Dear Dr. Azeb Gebresilassie Tesema,**

We sincerely thank you and the reviewers for the careful evaluation of our revised manuscript. We are especially grateful to Reviewer #2 and Reviewer #3 for recognizing the substantial improvements made during the previous revision and for confirming that the major concerns had been addressed.

The remaining comments were focused, constructive, and helpful. In response, we further clarified the method used to derive the three cross-cutting priorities, added an explicit discussion of trade-offs between NCD continuity and other immediate humanitarian health priorities, and expanded the limitations concerning the evidence-directness classification and differences across crisis phases. These revisions are highlighted in green in the marked manuscript.

Below, we provide a point-by-point response. Reviewer comments are reproduced in italicized text, followed by our responses and the corresponding changes made in the manuscript.

**Reviewer #2**

***Comment:*** *All comments have been addressed. The authors significantly improved the paper and my concerns are well addressed.*

**Response:** We thank Reviewer #2 . We are grateful for this assessment and for the reviewer's earlier comments, which helped us strengthen the analytical structure, methodological transparency, and practical relevance of the review.

**Reviewer #3**

***Comment:*** *Specify in Methods how the three priorities were derived—was there a threshold of recurrence? Did two reviewers independently identify them?*

**Response:** We agree that the derivation of the three priorities required a more explicit and reproducible description. We have added a new Methods subsection entitled “Derivation of cross-cutting priorities.” The revised text explains that two reviewers (AB and SK) independently identified candidate actions from the source-level extraction and evidence-profile tables, compared their candidate lists, and consolidated them through discussion, with consultation of a third reviewer (MB) when needed. We also clarify that no fixed numerical recurrence threshold, formal thematic-saturation rule, or inter-coder reliability statistic was applied because source types and domain assignments were heterogeneous and non-mutually exclusive. Instead, candidate actions were retained when they addressed recurrent continuity failures, were supported by more than one form of evidence, were relevant across multiple NCD or crisis contexts, and could be expressed using measurable implementation indicators.

**Change in the revised manuscript:** A new subsection, “Derivation of cross-cutting priorities,” has been added to the Methods (page 9). The opening paragraph of Results section 9 has also been revised to state that application of the predefined synthesis criteria yielded the three priorities and that they were not formally scored or ranked (page 21).

***Comment:*** *Add a brief sentence in Discussion on trade-offs: does prioritizing NCDs divert resources from trauma/infectious disease, and how should this be managed?*

**Response:** We agree that this trade-off should be addressed explicitly. We have added a paragraph explaining that continuity of NCD care should not be framed as displacing immediate life-saving responses for trauma, communicable diseases, nutrition, or maternal and child health. The revision proposes phased and context-sensitive allocation: protecting a minimum set of NCD functions during the acute phase to prevent avoidable decompensation and additional emergency demand, while expanding chronic-care services as response capacity stabilizes. It also identifies local disease burden, crisis phase, delivery capacity, equity, and opportunity costs as relevant allocation considerations.

**Change in the revised manuscript:** A new trade-off paragraph has been added to the Discussion immediately before the three operational priorities (page 25).

***Comment:*** *In Limitations, acknowledge that 'evidence directness' classification is based on author claims, not independent quality appraisal.*

**Response:** We agree. The revised limitations now state explicitly that the evidence-directness classification was based on outcomes and descriptions reported by the included sources and was not accompanied by independent verification or formal appraisal of methodological robustness. We further clarify that classification as patient or clinical outcome evidence identifies the type of outcome reported and should not be interpreted as evidence of high methodological quality, causal validity, or certainty.

**Change in the revised manuscript:** This clarification has been added to the Strengths and limitations subsection (page 26).

***Comment:*** *The review does not distinguish between crisis phases (acute versus protracted), despite potentially different feasibility.*

**Response:** We appreciate this observation. Although this issue was not repeated among the final three recommended edits, we agreed that it should be addressed. We have added a limitation stating that acute-onset, recovery, and protracted crisis settings were combined without systematic stratification and that the feasibility, relative priority, and expected effects of individual strategies may therefore differ across phases of an emergency.

**Change in the revised manuscript:** The crisis-phase limitation has been added to the Strengths and limitations subsection (page 26).

We again thank the Academic Editor and both reviewers for their careful and constructive evaluation. Their comments have helped us improve the transparency and interpretive balance of the manuscript. We hope that the revised version now fully addresses the remaining concerns and is suitable for publication in PLOS Global Public Health.

**Sincerely,**
Ahad Bakhtiari
On behalf of all authors
