# Supplementary material for: Health-system challenges, resilience strategies, and actionable priorities for non-communicable disease care in humanitarian crises: A scoping review
Source: PLOS Glob Public Health. 2026 Aug 18;6(8):e0006035. doi: 10.1371/journal.pgph.0006035 (PMC13485051; doi:10.1371/journal.pgph.0006035)
Supplement: Peer review history

# Peer review: Decision Letter 0

**DOI:** 10.1371/journal.pgph.0006035.r001

**Authors:** Azeb Gebresilassie Tesema

8 Jun 2026

PGPH-D-26-00504

Strengthening Preparedness for Non-Communicable Diseases in Humanitarian Crises: A Review of Actions for Countries and Humanitarian Organizations

PLOS Global Public Health

Dear Dr Ahad Bakhtiari

Thank you for submitting your manuscript to PLOS Global Public Health. After careful consideration, we feel that it has merit but does not fully meet PLOS Global Public Health’s publication criteria as it currently stands. Therefore, we invite you to submit a revised version of the manuscript that addresses the points raised during the review process.

Please ensure that all reviewer comments have been fully addressed. In particular, as highlighted by two of the  reviewers, the manuscript would benefit from a more detailed discussion of actionable priorities and policy recommendations arising from the study findings.

Please submit your revised manuscript by 22 July 2026. If you will need more time than this to complete your revisions, please reply to this message or contact the journal office at globalpubhealth@plos.org. When you're ready to submit your revision, log on to https://www.editorialmanager.com/pgph/ and select the 'Submissions Needing Revision' folder to locate your manuscript file.

Please include the following items when submitting your revised manuscript:

- A letter that responds to each point raised by the editor and reviewer(s). You should upload this letter as a separate file labeled 'Response to Reviewers'.
- A marked-up copy of your manuscript that highlights changes made to the original version. You should upload this as a separate file labeled 'Revised Manuscript with Track Changes'.
- An unmarked version of your revised paper without tracked changes. You should upload this as a separate file labeled 'Manuscript'.

Guidelines for resubmitting your figure files are available below the reviewer comments at the end of this letter.

As the corresponding author, your ORCID iD is verified in the submission system and will appear in the published article. PLOS supports the use of ORCID, and we encourage all coauthors to register for an ORCID iD and use it as well. Please encourage your coauthors to verify their ORCID iD within the submission system before final acceptance, as unverified ORCID iDs will not appear in the published article. *Only*  the individual author can complete the verification step; PLOS staff *cannot*  verify ORCID iDs on behalf of authors.

We look forward to receiving your revised manuscript.

Kind regards,

Azeb Gebresilassie Tesema, Ph.D.

Academic Editor

PLOS Global Public Health

Journal Requirements:

1. Please provide separate figure files in .tif or .eps format.

For more information about figure files please see our guidelines:

https://journals.plos.org/globalpublichealth/s/figures

https://journals.plos.org/globalpublichealth/s/figures#loc-file-requirements

If the reviewer comments include a recommendation to cite specific previously published works, please review and evaluate these publications to determine whether they are relevant and should be cited. There is no requirement to cite these works unless the editor has indicated otherwise.

Additional Editor Comments (if provided):

[Note: HTML markup is below. Please do not edit.]

Reviewers' comments:

Reviewer's Responses to Questions

**Comments to the Author**

1. Does this manuscript meet PLOS Global Public Health’s publication criteria?

Reviewer #1: Partly

Reviewer #2: Yes

Reviewer #3: Yes

**********

2. Has the statistical analysis been performed appropriately and rigorously?

Reviewer #1: No

Reviewer #2: N/A

Reviewer #3: I don't know

**********

3. Have the authors made all data underlying the findings in their manuscript fully available (please refer to the Data Availability Statement at the start of the manuscript PDF file)?

The PLOS Data policy

Reviewer #1: Yes

Reviewer #2: Yes

Reviewer #3: Yes

**********

4. Is the manuscript presented in an intelligible fashion and written in standard English?

Reviewer #1: Yes

Reviewer #2: Yes

Reviewer #3: Yes

**********

Reviewer #1: The manuscript appears overly generalized and does not adequately reflect the actual state of preparedness as suggested by the title. Additionally, the abstract lacks clarity in defining the core problem and does not clearly outline how the issue is addressed or what solutions are proposed. Strengthening the alignment between the title, abstract, and the study’s specific findings would improve the overall clarity and impact of the paper.

Reviewer #2: Title:

• The topic is highly relevant and timely, as the world is facing an unprecedented polycrisis. However, the authors could add resilience vocabulary to the title

Abstract:

• Add inclusion and exclusion criteria.

• They have to tell how they thematized the findings into nine categories. Did they use existing frameworks?

• They focused on challenge identification, not actions. I would recommend they change their title

Introduction:

• Well-written introduction

• Research gap well crafted

Methods

• In the exclusion criteria, the points following “Studies were excluded if they:” should be indented.

• PRISMA is used for systematic reviews. They should have used PRISMA-ScR (extension of PRISMA for scoping review).

• They stated that they used inductive coding for data analysis. However, the components of the WHO's six building blocks are clearly seen in the domains. The authors should clarify whether they used an inductive and deductive coding approach (a hybrid one).

Results

• Your studies are heavily concentrated in the Middle East and Asia. This may show a geographic bias. Therefore, putting this as a limitation may strengthen your study.

Discussions

• Well discussed

Citations

• Make sure the 37 selected articles in the results section have been correctly cited

General comments

• I would appreciate it if the authors wrote their findings based on resilience strategies, such as absorption, adaptation, and transformation

Reviewer #3: SUMMARY:

This scoping review synthesizes 37 studies on preparedness for NCDs (cardiovascular disease, diabetes, cancer, chronic respiratory diseases) in humanitarian crises, organizing findings into nine domains.

It concludes that systemic gaps in planning, financing, supply chains, training, and community engagement persist.

WHAT’S ACTUALLY NEW:

Little. The nine-domain framework is descriptive, not novel.

Similar taxonomies already exist in WHO (2016, 2024) and UNHCR (2020) guidance.

The review confirms known problems without offering new analytical synthesis, causal modeling, or prioritization among domains.

The claim that challenges are “systemic rather than disease-specific” is unsurprising and unquantified.

STATED LIMITATIONS:

English-only, no quality appraisal of included studies (scoping review nature), possible publication bias, heterogeneous settings limiting generalizability.

UNSTATED LIMITATIONS:

(1) No critical assessment of whether any included intervention actually improved patient outcomes; most evidence is process-based.

(2) No distinction between feasibility in low- vs. high-income humanitarian settings.

(3) Authors include no patient or humanitarian practitioner co-design.

(4) Search ended in 2025 but many included references predate 2020; nearly half of cited NCD-humanitarian guidance is pre-2021.

(5) No comparison with non-NCD chronic conditions (e.g., mental health, dialysis) to test specificity.

IMPORTANCE:

Moderate. The topic is undeniably important, but the review adds primarily descriptive weight, not actionable prioritisation or implementation science insights.

RECOMMENDATION:

must: (1) Add a table rating strength of evidence per domain (e.g., number of studies with direct outcome data).

(2) Explicitly state which domains have cost-effectiveness data.

(3) Replace vague calls for “integration” with three specific, testable policy actions.

**********

what does this mean?). If published, this will include your full peer review and any attached files.

**Do you want your identity to be public for this peer review?**  If you choose “no”, your identity will remain anonymous but your review may still be made public.

For information about this choice, including consent withdrawal, please see our Privacy Policy

Reviewer #1: **Yes:**  Immaculate Wandera

Reviewer #2: **Yes:**  Mengistu Hagazi Tequare

Assistant Professor of Health Systems

Mekelle University, Ethiopia

Reviewer #3: No

**********

[NOTE: If reviewer comments were submitted as an attachment file, they will be attached to this email and accessible via the submission site. Please log into your account, locate the manuscript record, and check for the action link "View Attachments". If this link does not appear, there are no attachment files.]

# Author comment: Author response to Decision Letter 1

**DOI:** 10.1371/journal.pgph.0006035.r002

26 Jun 2026

Attachment Submitted filename: Response.docx

# Peer review: Decision Letter 1

**DOI:** 10.1371/journal.pgph.0006035.r003

**Authors:** Azeb Gebresilassie Tesema

17 Jul 2026

PGPH-D-26-00504R1

Health-System Challenges, Resilience Strategies, and Actionable Priorities for Non-Communicable Disease Care in Humanitarian Crises: A Scoping Review

PLOS Global Public Health

Thank you for submitting your manuscript to PLOS Global Public Health. After careful consideration, we feel that it has merit but does not fully meet PLOS Global Public Health’s publication criteria as it currently stands. Therefore, we invite you to submit a revised version of the manuscript that addresses the points raised during the review process.

Please submit your revised manuscript by 30 August 2026. If you will need more time than this to complete your revisions, please reply to this message or contact the journal office at globalpubhealth@plos.org. When you're ready to submit your revision, log on to https://www.editorialmanager.com/pgph/ and select the 'Submissions Needing Revision' folder to locate your manuscript file.

Please include the following items when submitting your revised manuscript:

- A letter that responds to each point raised by the editor and reviewer(s). You should upload this letter as a separate file labeled 'Response to Reviewers'.
- A marked-up copy of your manuscript that highlights changes made to the original version. You should upload this as a separate file labeled 'Revised Manuscript with Track Changes'.
- An unmarked version of your revised paper without tracked changes. You should upload this as a separate file labeled 'Manuscript'.

Guidelines for resubmitting your figure files are available below the reviewer comments at the end of this letter.

As the corresponding author, your ORCID iD is verified in the submission system and will appear in the published article. PLOS supports the use of ORCID, and we encourage all coauthors to register for an ORCID iD and use it as well. Please encourage your coauthors to verify their ORCID iD within the submission system before final acceptance, as unverified ORCID iDs will not appear in the published article. *Only*  the individual author can complete the verification step; PLOS staff *cannot*  verify ORCID iDs on behalf of authors.

We look forward to receiving your revised manuscript.

Kind regards,

Azeb Gebresilassie Tesema, Ph.D.

Academic Editor

PLOS Global Public Health

Journal Requirements:

If the reviewer comments include a recommendation to cite specific previously published works, please review and evaluate these publications to determine whether they are relevant and should be cited. There is no requirement to cite these works unless the editor has indicated otherwise.

Please review your reference list to ensure that it is complete and correct. If you have cited papers that have been retracted, please include the rationale for doing so in the manuscript text, or remove these references and replace them with relevant current references. Any changes to the reference list should be mentioned in the rebuttal letter that accompanies your revised manuscript. If you need to cite a retracted article, indicate the article’s retracted status in the References list and also include a citation and full reference for the retraction notice.

Additional Editor Comments (if provided):

[Note: HTML markup is below. Please do not edit.]

Reviewers' comments:

Reviewer's Responses to Questions

**Comments to the Author**

Reviewer #2: All comments have been addressed

Reviewer #3: All comments have been addressed

**********

publication criteria?

Reviewer #2: Yes

Reviewer #3: Partly

**********

3. Has the statistical analysis been performed appropriately and rigorously?

Reviewer #2: N/A

Reviewer #3: I don't know

**********

4. Have the authors made all data underlying the findings in their manuscript fully available (please refer to the Data Availability Statement at the start of the manuscript PDF file)?

The PLOS Data policy

Reviewer #2: Yes

Reviewer #3: Yes

**********

5. Is the manuscript presented in an intelligible fashion and written in standard English?

Reviewer #2: Yes

Reviewer #3: Yes

**********

Reviewer #2: The authors significantly improved the paper and my concerns are well addressed.

Reviewer #3: SUMMARY:

This revised scoping review maps health-system challenges, implemented and recommended strategies, and evidence gaps for NCD care (CVD, diabetes, cancer, chronic respiratory diseases) in humanitarian crises. It synthesizes 37 sources across nine operational domains, characterizes evidence directness (patient outcomes vs. service outcomes vs. descriptive/recommendation-only), applies an absorptive-adaptive-transformative resilience lens, and proposes three actionable priorities: (1) minimum emergency NCD service/medicine package; (2) continuity mechanisms for high-risk patients; (3) simplified integrated NCD care.

WHAT'S ACTUALLY NEW:

Modest but meaningful improvements over original.

The evidence-directness characterization (distinguishing patient/clinical outcomes from service/implementation from descriptive/recommendation) is a useful analytical contribution.

The resilience framing (absorptive vs. adaptive vs. transformative) provides interpretive clarity, though not novel per se.

The three concrete priorities with suggested indicators represent genuine operational translation beyond vague "integration" calls.

However, the nine-domain framework remains derivative of existing WHO/UNHCR taxonomies, explicitly acknowledged.

STATED LIMITATIONS:

English-only; geographic concentration in Asia/Middle East; no formal risk-of-bias or certainty assessment; heterogeneity prevents quantitative synthesis; no systematic feasibility comparison across resource settings; no patient/practitioner co-design; no independent mapping of mental health/kidney disease; some evidence predates recent digital health developments.

UNSTATED LIMITATIONS:

(1) The evidence-classification relies on source-level reporting, not independent verification—a source claiming "patient outcomes" may have weak methodology.

(2) No distinction between crisis phases (acute vs. protracted) despite very different feasibility.

(3) The three priorities are presented as "emerging" but the synthesis method for deriving them is under-specified—no formal thematic saturation or inter-coder reliability reported.

(4) The review does not address whether NCD preparedness competes with or complements other humanitarian health priorities (trade-offs).

IMPORTANCE:

Moderate-high. The revision substantially addresses prior criticisms.

The paper now offers a clearer evidence map, distinguishes recommendation from implementation, and translates broad policy consensus into testable operational priorities.

This is useful for policymakers, humanitarian actors, and implementation researchers.

It does not, however, advance primary evidence or causal inference.

RECOMMENDATION:

Accept with minor revisions.

The revision is responsive and substantially improved.

Recommended minor edits:

Specify in Methods how the three priorities were derived—was there a threshold of recurrence?

Did two reviewers independently identify them?

Add a brief sentence in Discussion on trade-offs: does prioritizing NCDs divert resources from trauma/infectious disease, and how should this be managed?

In Limitations, acknowledge that "evidence directness" classification is based on author claims, not independent quality appraisal.

**********

what does this mean?). If published, this will include your full peer review and any attached files.

**Do you want your identity to be public for this peer review?**  If you choose “no”, your identity will remain anonymous but your review may still be made public.

For information about this choice, including consent withdrawal, please see our Privacy Policy

Reviewer #2: No

Reviewer #3: No

**********

[NOTE: If reviewer comments were submitted as an attachment file, they will be attached to this email and accessible via the submission site. Please log into your account, locate the manuscript record, and check for the action link "View Attachments". If this link does not appear, there are no attachment files.]

# Author comment: Author response to Decision Letter 2

**DOI:** 10.1371/journal.pgph.0006035.r004

20 Jul 2026

Attachment Submitted filename: Response 2.docx

# Editor report: Decision Letter 2

**DOI:** 10.1371/journal.pgph.0006035.r005

**Authors:** Azeb Gebresilassie Tesema

22 Jul 2026

Health-System Challenges, Resilience Strategies, and Actionable Priorities for Non-Communicable Disease Care in Humanitarian Crises: A Scoping Review

PGPH-D-26-00504R2

Dr Ahad Bakhtiari

We are pleased to inform you that your manuscript 'Health-System Challenges, Resilience Strategies, and Actionable Priorities for Non-Communicable Disease Care in Humanitarian Crises: A Scoping Review' has been provisionally accepted for publication in PLOS Global Public Health.

Before your manuscript can be formally accepted you will need to complete some formatting changes, which you will receive in a follow up email. A member of our team will be in touch with a set of requests.

Please note that your manuscript will not be scheduled for publication until you have made the required changes, so a swift response is appreciated.

IMPORTANT: The editorial review process is now complete. PLOS will only permit corrections to spelling, formatting or significant scientific errors from this point onwards. Requests for major changes, or any which affect the scientific understanding of your work, will cause delays to the publication date of your manuscript.

If your institution or institutions have a press office, please notify them about your upcoming paper to help maximize its impact. If they'll be preparing press materials, please inform our press team as soon as possible -- no later than 48 hours after receiving the formal acceptance. Your manuscript will remain under strict press embargo until 2 pm Eastern Time on the date of publication. For more information, please contact globalpubhealth@plos.org.

Thank you again for supporting Open Access publishing; we are looking forward to publishing your work in PLOS Global Public Health.

Best regards,

Azeb Gebresilassie Tesema, Ph.D.

Academic Editor

PLOS Global Public Health

***********************************************************

Reviewer Comments (if any, and for reference):
